# rTMS for the treatment of psychiatric disorders: a review about training courses and materials and the presentation of the training materials of the German Society for Brain Stimulation in Psychiatry

**DOI:** 10.3389/fpsyt.2025.1490039

**Published:** 2025-08-08

**Authors:** Martin Schecklmann, Wolfgang Strube, Katrin Sakreida, Ulrike Vogelmann, Christiane Licht, Andreas Reissmann, Stefanie Dierkes-Möller, Roberto Goya-Maldonado, Bernhard Kis, Michael Landgrebe, Carlos Schönfeldt-Lecuona, Berthold Langguth, Tobias Hebel

**Affiliations:** ^1^ Department of Psychiatry and Psychotherapy, University of Regensburg, Regensburg, Germany; ^2^ Department of Psychiatry, Psychotherapy and Psychosomatics, Medical Faculty, University of Augsburg, Augsburg, Germany; ^3^ Deutsches Zentrum für Psychische Gesundheit (DZPG, German Center for Mental Health), Augsburg, Germany; ^4^ Department of Psychiatry, Psychotherapy and Psychosomatics, Faculty of Medicine, Rheinisch-Westfälische Technische Hochschule (RWTH) Aachen University, Aachen, Germany; ^5^ Deutsches Zentrum für Psychische Gesundheit (DZPG, German Center for Mental Health), Munich, Germany; ^6^ Department of Psychiatry and Psychotherapy, Technical University of Munich (TUM) School of Medicine and Health, Klinikum rechts der Isar, Technical University of Munich, Munich, Germany; ^7^ Neurologie im Vest, Recklinghausen, Germany; ^8^ Laboratory of Systems Neuroscience and Imaging in Psychiatry (SNIP-Lab), Department of Psychiatry and Psychotherapy, University Medical Center Göttingen (UMG), Göttingen, Germany; ^9^ Department of Psychiatry, Psychotherapy and Psychosomatics, Catholic Hospitals Ruhrhalbinsel, Hattingen, Germany; ^10^ Department of Psychiatry, Psychosomatics and Psychotherapy, kbo-Lech-Mangfall-Klinik Agatharied, Hausham, Germany; ^11^ Department of Psychiatry and Psychotherapy III, University of Ulm, Ulm, Germany; ^12^ CuraMed day-clinic for Psychiatry, Psychosomatic Medicine & Psychotherapy, Neu-Ulm, Germany

**Keywords:** manual, hands-on, training, transcranial magnetic stimulation, review

## Abstract

There is an increasing interest in the treatment of mental disorders such as depression using repetitive transcranial magnetic stimulation (rTMS). Treatment guidelines for the application of rTMS are available, but they focus primarily on scientific evidence for efficacy and tolerability. Other aspects that are relevant for the training of practitioners, such as implementation in the national health system as well as organizational, formal, and practical aspects, are not covered in detail in these guidelines. Here, we present an overview of German rTMS courses and present training materials for the hands-on workshop of the German Society for Brain Stimulation in Psychiatry [Deutsche Gesellschaft für Hirnstimulation in der Psychiatrie (DGHP) e.V.] in German and English as an example. Publishing national standard operating procedures is important in order to harmonize rTMS practice all over the world.

## 1 Introduction

Interest in repetitive transcranial magnetic stimulation (rTMS) for the treatment of depression and other mental diseases is growing worldwide. The basis for this development is scientific evidence that rTMS is a safe and effective treatment for depression (1). Boosters for this development are an increasing number of approvals by national authorities such as the US Food and Drug Administration (2). However, despite the scientific evidence, the availability of rTMS treatment is still limited. In Germany, boosters for rTMS are the recommendations in national clinical guidelines and the reimbursability for inpatients since 2021. Therefore, interest is also increasing in Germany. This interest leads to an increased need for training of rTMS practitioners and to the development of standard operating procedures. In this context, it is important to provide guidance about the essential information necessary for the effective and safe clinical application of rTMS out of the huge amount of scientific data in the rTMS field. Furthermore, it is important that rTMS users apply this stimulation technique at high- quality standards. Thus, it is essential to have not only paper-based guidelines but also practical standard procedures integrated into hands-on workshops, including the different aspects such as the handling and positioning of the coil and the TMS machine, informed consent issues, documentation, treatment organization, and reimbursement.

While there are published recommendations regarding the contents of training in the application of non-invasive brain stimulation techniques published by the International Federation of Clinical Neurophysiology (3), there is limited availability of material that addresses the practical, organizational, and administrative aspects of rTMS implementation in clinical routine. As a result, interested clinicians often lack concrete, applicable guidance on how to initiate and integrate rTMS into their daily practice regarding topics such as motor threshold determination, documentation standards, billing procedures, team coordination, or standard operating procedures. This practical gap can hinder the broader dissemination of TMS despite its proven efficacy.

Here, we aim to review German training courses for rTMS in psychiatry and provide the material of a standard German workshop of the German Society for Brain Stimulation in Psychiatry (DGHP) as an example. The material includes a hands-on manual that covers not only the clinical but also organizational and procedural aspects of rTMS. We believe that the actual and free availability of such material may serve as a practically oriented reference for beginners as well as advanced users of rTMS in the clinical field and support their implementation of evidence-based rTMS treatment. Moreover, we believe that the availability of such materials helps to harmonize and promote the training and application of scientifically sound rTMS procedures at both the national and international levels.

## 2 Training materials in Germany

### 2.1 Overview

First, we screened PubMed ^®^ (National Library of Medicine, NIH, USA) for relevant publications. Literature research with the key words “training”, “hands-on”, “practice*”, “guide*”, “manual” in combination with “transcranial magnetic”, “TMS”, and “rTMS” resulted in international (1, 4) or national guidelines (5–7). For Germany, we found one guideline (8). However, most of these publications do not include practical training materials or recommendations regarding training (but see 3). Thus, guidelines remain at a more academic or theoretical level. At least for Germany, we found no training material. Thus, we also searched the homepages of TMS companies, hospitals, and specialized societies for workshops or rTMS training procedures.

The German Society for Brain Stimulation in Psychiatry (www.dghp-online.de) and the Department of Transcranial and Deep Brain Stimulation of the Section Brain Stimulation Methods of the German Association for Psychiatry, Psychotherapy and Psychosomatics (www.dgppn.de) offer hands-on workshops associated with their annual society meeting. The Neurocare Group (neurocare group AG 2024) promotes an rTMS practical course (https://magandmore.com/de/tms-praxiskurs-mit-dr-wolfgang-strube) headed by a psychiatrist (author WS). The course “Transcranial Brain Stimulation: from Basics to Advanced Applications” of the University of Mainz (https://www.unimedizin-mainz.de/transmed/training-program/training-in-scientific-skills/transcranial-brain-stimulation-from-basics-to-advanced-applications.html) is available on demand and includes other stimulation methods such as transcranial ultrasound stimulation and transcranial electrical stimulation. The same is true for the workshop of the Noninvasive Brain Stimulation Lab of the Department of Neurology of the University Medical Center Göttingen. The German Society for Clinical Neurophysiology and Functional Imaging (www.dgkn.de) also offers a TMS workshop. However, it is not specialized for treatments in psychiatry.

None of these workshops provides free access to the training material. In the present publication, we want to make the material of the DGHP freely available in German and English.

### 2.2 The hands-on workshop of the DGHP

The DGHP (www.dghp-online.de) is a non-profit organization established in 2011. Professionals from different disciplines working in university hospitals, psychiatric hospitals, psychiatric departments, or psychiatric outpatient practices are members of the society. In order to ensure the application of brain stimulation methods in psychiatry in Germany at a high quality and on a scientifically sound level, the DGHP has developed standard operating procedures. These pertain to the application of rTMS in the adult age range and focus on psychiatric indications (i.e., excluding diagnosis groups more typically encountered in neurological departments, such as dementias, or movement disorders, such as Parkinson’s disease or post-stroke syndromes). The society awards a certificate titled “non-invasive and non-convulsive transcranial brain stimulation methods for therapy of mental disorders” for psychiatrists and psychotherapists who fulfill the following specific criteria: i) continuous use of non-invasive brain stimulation and ii) theoretical knowledge and practical expertise in this field. Theoretical knowledge and practical experience in brain stimulation methods have recently also been introduced as a requirement to become a specialist in psychiatry and psychotherapy in Germany.

The DGHP holds an annual meeting at different locations within German-speaking countries, consisting of a scientific program and a hands-on workshop on rTMS. Since 2023, this workshop has consisted of 3 hours of theoretical knowledge transfer and 3 hours of practical training. The members of the DGHP were invited to contribute to writing a handbook for users. Several rounds of discussions among the authors MS, TH, WS, KS, UV, CL, SDM, RGM, BK, ML, CSL, and BL led to the present version of the handbook. In 2024, a smaller group (MS, WS, KS, UV, AR, and CL) developed additional material for didactic purposes. In detail, this material encompasses i) a poster with instructions for treatment with rTMS, ii) questions for controlling learning objectives, and iii) presentation slides. All authors are practitioners of rTMS, and most of them are scientifically active.

For all materials, state-of-the- art reviews, expert opinions, and meta-analyses were included (1, 4, 8–13). Special consideration was given to the German guideline for rTMS in depression (8), which was developed by the DGHP together with the sections Clinical Brain Stimulation and Experimental Brain Stimulation of the German Association for Psychiatry, Psychotherapy and Psychosomatics (DGPPN).

The presented material (handbook, presentation slides, instruction poster, and exam questions) has been prepared for the annual hands-on workshop of the DGHP as part of the quality assurance measures of the DGHP. The material presented is intended to help clinicians and researchers carry out rTMS in accordance with our proposed good practice recommendations and high- quality standards. The material is provided in German and English in the **Supplementary Material**. We are aware that scientific development in the future will require regular updates of the proposed educational material. It should be a “living document” that must be updated every year or at least every 2 years.

## 3 Discussion

The scientific literature about the efficacy and safety of rTMS for the treatment of various psychiatric disorders is summarized in meta-analyses and systematic reviews, and their results are condensed in published guidelines for the use of rTMS in psychiatry. However, these guidelines do not cover all aspects that are relevant for training to effectively and safely apply TMS, as well as other aspects of the practical implementation of rTMS. Therefore, we present educational and training materials that have been developed to cover relevant practical aspects such as indication and contraindication for treatment, practical application, standard operating procedures, operation of stimulation devices, use of helpful tools, trouble shooting, emergency management, informed consent, documentation of treatment, and reimbursement aspects. Some of these aspects are health system- and country- specific but can be used as an example and a basis for the international harmonization of the application of rTMS in psychiatry. As the presented guidelines and training materials focus on the adult range, a current limitation is the lack of coverage regarding the use of rTMS in child and adolescent psychiatric populations. Given the increasing scientific and clinical interest in rTMS as an adjunct or alternative treatment in these age groups (14), increased acknowledgement of these aspects may become relevant for future revisions of rTMS workshop materials and guidelines.
