# Supplementary material for: rTMS for the treatment of psychiatric disorders: a review about training courses and materials and the presentation of the training materials of the German Society for Brain Stimulation in Psychiatry
Source: Front Psychiatry. 2025 Aug 8;16:1490039. doi: 10.3389/fpsyt.2025.1490039 (PMC12371536; doi:10.3389/fpsyt.2025.1490039)
Supplement: Supplementary file 1 [file SupplementaryFile1.zip › Exam Questions (English).PDF]

Transcranial Magnetic Stimulation

German Society for Brain Stimulation in Psychiatry (DGHP; registered society)

Hands-On Workshop Beginner June 2024

Deutsche Gesellschaft für
Hirnstimulation
in der Psychiatrie e. V.

Exam questions (correct answer = bold)

1. How does rTMS work physiologically?

A) Cortical neurons are influenced by the positive effect of magnetic shock waves.

B) The vibration of magnetic pulses leads to increased release of neurotrophic growth factors.

C) Under a magnetic coil and through the physical principle of electromagnetic induction, cortical neurons are depolarized by an electrical current passing through conductive brain tissue.

D) During stimulation, the left hemisphere is momentarily magnetized. Free radicals are thus magnetically attracted and subsequently metabolized more efficiently.

2. How does rTMS work therapeutically?

A) It can only induce short-term effects, limited to the duration of the therapy sessions.

B) It works neuroplastically and can only have an excitatory effect on neuronal networks.

C) It can alter brain function over the course of weeks and months.

D) A 1Hz protocol is considered exclusively inhibitory and a 10Hz protocol is considered exclusively excitatory.

3. Which statement regarding the penetration depth of rTMS pulses is correct?

A) Since rTMS magnetic fields are not "stopped" by bones or other structures, they can penetrate unhindered into the brainstem.

B) The magnetic field has a direct stimulating effect on the cerebral cortex.

C) Certain coils allow for greater penetration depth. With these specific coils, any effects on brain areas between said deeper target regions and the coil are irrelevant.

D) The motor threshold is a measure of the excitability for the motor cortex and other cortical brain areas.

4. Why is an electromyography (EMG) recommended for the accurate determination of the RMT (resting motor threshold)?

A) Patients feel better with more technical support.

B) EMG helps to more accurately determine the individual risk of an induced seizure and thus avoid it.

C) Determining the motor threshold with EMG may be more precise because even non-visible muscle activity can be measured.

D) EMG ensures that TMS pulses are transmitted to the small hand muscles.

5. Which statement about risk management is correct?

A) An EEG should be performed before starting treatment for better risk screening, as induced seizures are frequently observed under rTMS.

B) Syncope as a side effect is completely unproblematic because it only occurs under high tension and can always be easily avoided by relaxation strategies.

C) Pregnancy is an absolute contraindication for rTMS.

D) Hearing protection should be recommended during treatment.

6. Which statement regarding pre-treatment consultation is correct?

A) A signed written consent document is sufficient.

B) A pre-treatment consultation is to be given verbally and documented.

C) Treatment alternatives do not need to be mentioned.

D) Since rTMS has so few side effects, it is not necessary to give the patient any time to consider.

7. Based on current evidence, which of the following treatment protocols for unipolar depressive disorder is the most effective?

A) Stimulation over the left DLPFC with a high-frequency protocol

B) Stimulation over the right DLPFC with a high-frequency protocol

C) Stimulation over the left DLPFC with a low-frequency protocol

D) Stimulation over the right DLPFC with a low-frequency protocol

8. Which statement about the treatment of depression with rTMS is correct?

A) Individuals with bipolar depression do not benefit from rTMS treatment according to recent study findings.

B) Due to its high risk, rTMS cannot be offered as an individual treatment attempt for peripartum depression.

C) In individuals with depression over the age of 65, neuroplastic therapies show no evidence of efficacy.

D) rTMS can be used in addition to most medications.

9. What is the average response rate to regular rTMS treatment in people with depression?

A) Approximately 90% of patients are in remission after four weeks.

B) Approximately 10-50% of patients respond to treatment.

C) In combination with Moclobemide, the remission rate can be increased to up to 70%.

D) The response rate after four weeks is 5%.

10. You currently have only one treatment slot available for rTMS. Which of the following patients would you most likely treat?

A) A 27-year-old pregnant patient, who does not want to take medication and has previously benefited from rTMS, currently on her third week of a second depressive episode.

B) A 68-year-old patient with treatment-resistant depression and somatic comorbidities, as well as numerous somatic and psychiatric medications.

C) A 41-year-old patient with depressive stupor.

D) A 25-year-old patient with borderline personality disorder, ADHD, and PTSD, currently suffering from a high degree of psychological strain, because many medications have not helped in the past.

11. What is the most effective way to determine stimulation intensity?

A) By asking the patients about the strength of any perceived pain during stimulation and adjusting the stimulation strength if necessary.

B) By using a group reference value from a scientific publication.

C) By determining the individual motor threshold as a measure of cortical excitability.

D) It is best to use 60% of the stimulator output.

12. Which statement about neuronavigation and its use in rTMS for treating depression in the DLPFC (dorso-lateral prefrontal cortex) is true?

A) The use of neuronavigation is always preferred over other methods because it consistently produces stronger effects.

B) Neuronavigation is a method used to precisely define brain areas for stimulation. However, localization using EEG points, such as the Beam-F3 method, leads to similar anatomical accuracy.

C) The 5cm rule remains the gold standard for rTMS in clinical practice to this day.

D) The accuracy of coil localization is particularly important when treating tinnitus, so neuronavigation is primarily used for this condition.
