# Supplementary material for: rTMS for the treatment of psychiatric disorders: a review about training courses and materials and the presentation of the training materials of the German Society for Brain Stimulation in Psychiatry
Source: Front Psychiatry. 2025 Aug 8;16:1490039. doi: 10.3389/fpsyt.2025.1490039 (PMC12371536; doi:10.3389/fpsyt.2025.1490039)

Transkranielle Magnetstimulation

Deutsche Gesellschaft für Hirnstimulation in der Psychiatrie e.V. (DGHP)

Hands-On-Workshop April 2025

Deutsche Gesellschaft für
Hirnstimulation
in der Psychiatrie e. V.

Transkranielle Magnetstimulation

Deutsche Gesellschaft für Hirnstimulation in der Psychiatrie e.V. (DGHP)

Hands-On-Workshop Beginner April 2025

Deutsche Gesellschaft für
Hirnstimulation
in der Psychiatrie e. V.

Übersicht (3h Theorie & 3h Praxis)

Deutsche Gesellschaft für Hirnstimulation in der Psychiatrie e. V.

Was?	Wo?	Wann?
Theorie: Basics, Spulenpositionierung – allgemein, Motorschwelle, Behandlung der Depression		2 Stunden
Hands-On		1 Stunde
Pause		1 Stunde
Hands-On		1 Stunde
Hands-On		1 Stunde
Theorie: Aufklärung, Kontraindikationen und Nebenwirkungen, Vergütung		1 Stunde
Abschluss		

Basics

Basics: Physik und Technik

Deutsche Gesellschaft für Hirnstimulation in der Psychiatrie e. V.

- Basis: elektromagnetische Induktion

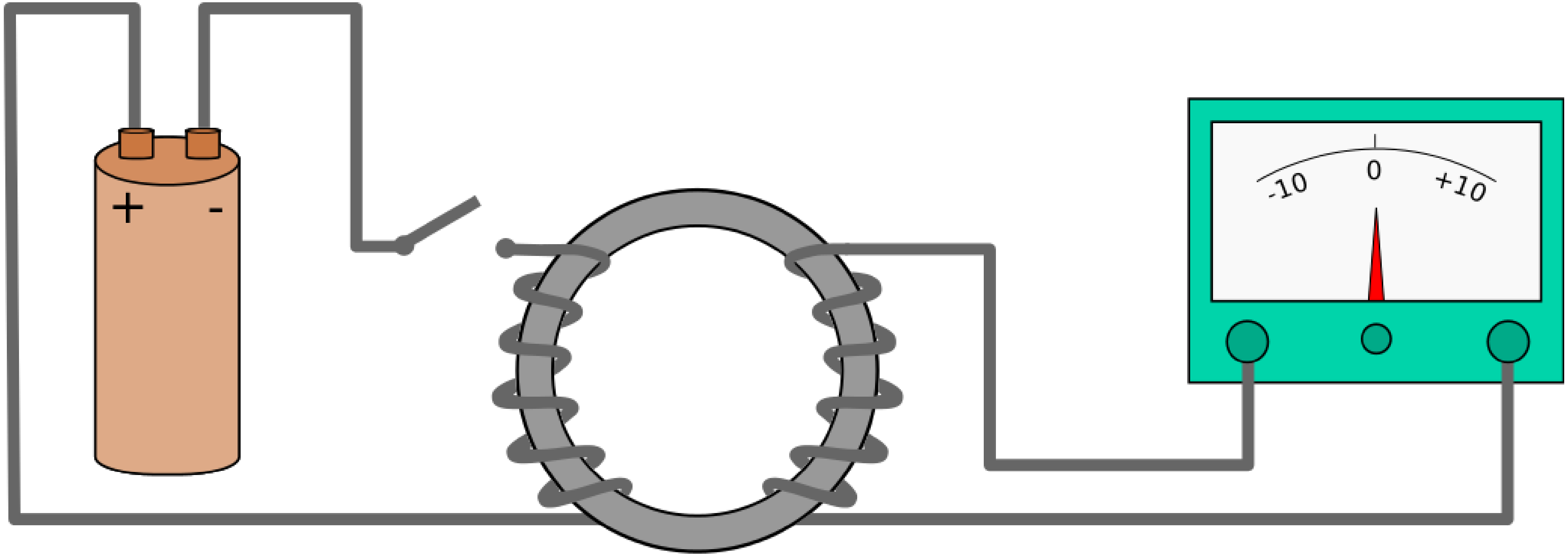

https://de.wikipedia.org/wiki/Elektromagnetische_Induktion

Basics: Physik und Technik

Deutsche Gesellschaft für Hirnstimulation in der Psychiatrie e. V.

- Basis: elektromagnetische Induktion
- Prinzip: Neuronen als elektrische Leiter

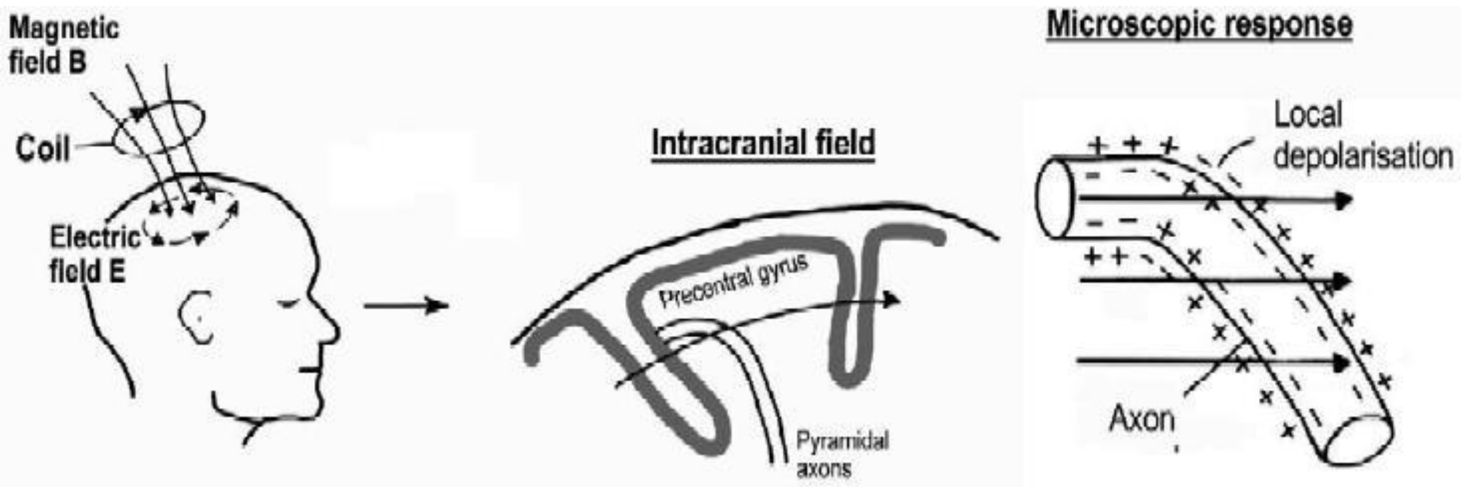

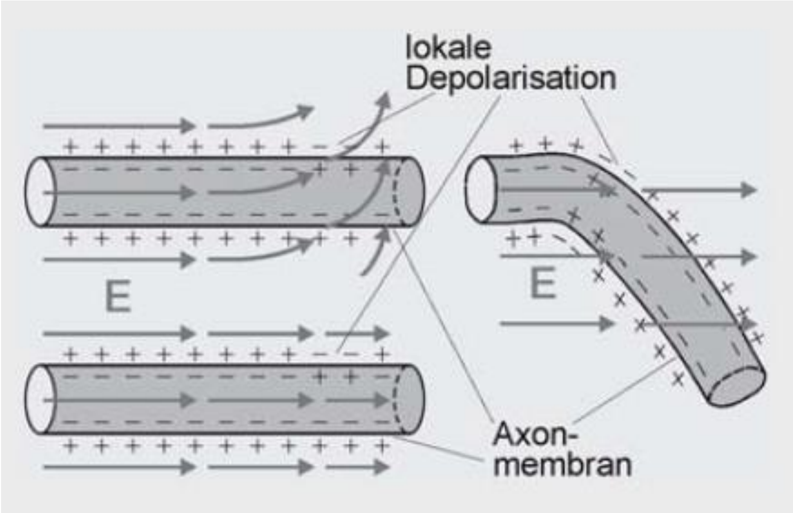

Siebner & Ziemann (2007) - doi: 10.1007/978-3-540-71905-2
Viesca et al. (2012) - doi: 10.1063/1.4764608

Basics: Physik und Technik

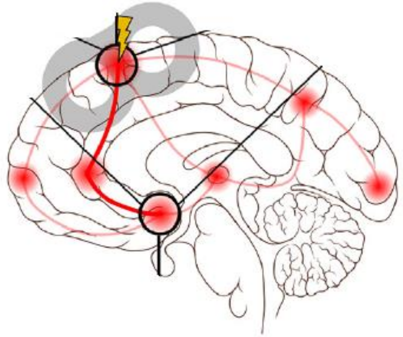

Deutsche Gesellschaft für
Hirnstimulation
in der Psychiatrie e. V.

- Basis: elektromagnetische Induktion
- Prinzip: Neuronen als elektrische Leiter
- induzierte elektrische Felder: kalotten-nah

Remote-Effekte sind wahrscheinlich!

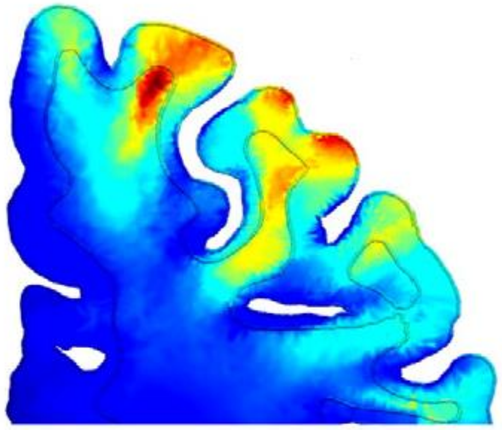

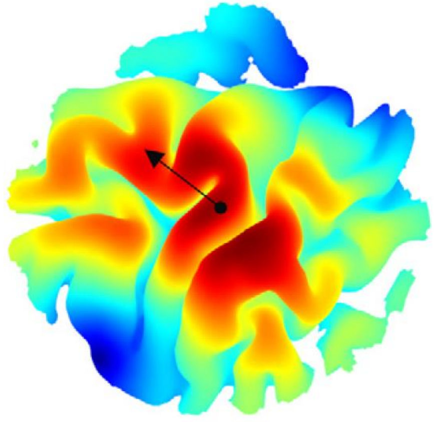

Erdmagnetfeld: 40µT
Hufeisenmagnet: 4mT
TMS/MRT: 1-3T

Optiz et al. (2011) - doi: 10.1016/j.neuroimage.2011.06.069
Mueller-Dahlhaus & Bergmann (2023) - doi: 10.1016/j.xcrm.2023.101086

Basics: Physik und Technik

- Basis: elektromagnetische Induktion
- Prinzip: Neuronen als elektrische Leiter
- induzierte elektrische Felder: kalotten-nah
- Spulengeometrie: Wicklung, Winklung, Durchmesser etc. sind relevant!

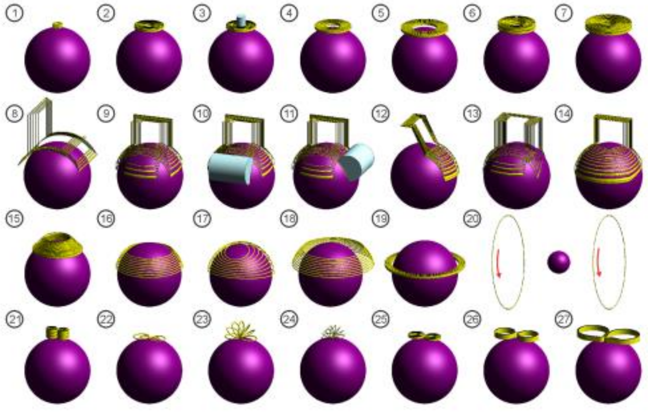

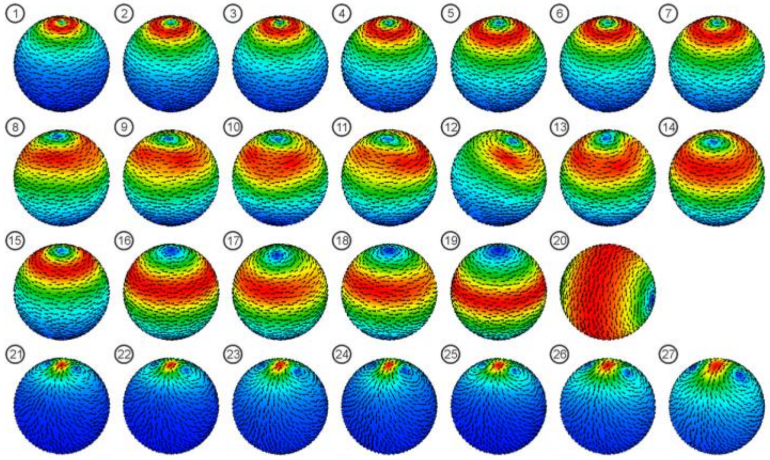

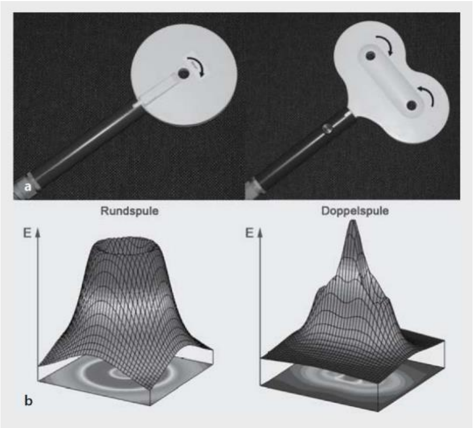

Kaum Untersuchungen mit Rund-Spule vorhanden!

Siebner & Ziemann (2007) - doi: 10.1007/978-3-540-71905-2
Deng et al. (2013) - doi:10.1016/j.brs.2012.02.005

Basics: Physik und Technik

- Basis: elektromagnetische Induktion
- Prinzip: Neuronen als elektrische Leiter
- induzierte elektrische Felder: kalotten-nah
- Spulengeometrie
- Pulsrichtung/Spulenrichtung („Wohin zeigt der Griff?“): Gerätehersteller mit unterschiedlichen Default-Einstellungen

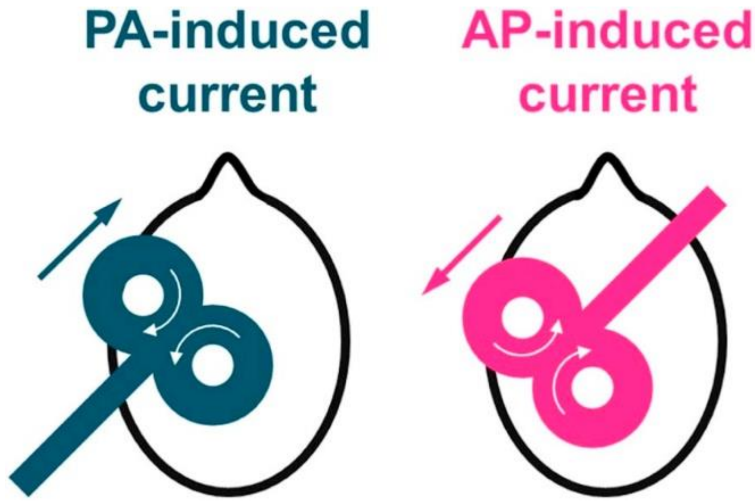

Hannah & Rothwell (2017) - doi: 10.1016/j.brs.2016.09.008
Schoisswohl et al. (2022) - doi: 10.1016/j.neulet.2022.137026

Basics: Stimulationsparameter

Deutsche Gesellschaft für Hirnstimulation in der Psychiatrie e. V.

- Intensität = Stärke der Stimulation: Einheiten
- Maßeinheiten:
  - Stimulator-Output (angegeben in Prozent der Leistung des Geräts, d.h. %MSO)
    - → CAVE: Nicht jede Spule und jeder Stimulator haben die gleiche Leistung!
  - Motorschwelle (angegeben in % Stimulator-Output)
  - Stimulationsstärke auf Basis der Motorschwelle (z.B. 110% Motorschwelle meint: bei einer Motorschwelle von 50% Stimulator-Output sind das 55% Stimulator-Output)
    - → CAVE: Es gibt auch Hinweise auf die Wirksamkeit der unterschwelligen Stimulation!

Basics: Stimulationsparameter

Deutsche Gesellschaft für
Hirnstimulation
in der Psychiatrie e. V.

- Intensität = Stärke der Stimulation
- Frequenz = Schnelligkeit oder Häufigkeit der Pulse in einem Zeitraum
  - ≤1Hz: gilt als inhibitorisch
  - >5Hz: gilt als exzitatorisch
  - Einteilung im psychiatrisch-therapeutischen Kontext

Basics: Stimulationsparameter

Deutsche Gesellschaft für Hirnstimulation in der Psychiatrie e. V.

- Intensität = Stärke der Stimulation
- Frequenz = Schnelligkeit oder Häufigkeit der Pulse in einem Zeitraum
- Train = Anzahl von Pulsen in einem Block
- Inter-Train-Intervall: Pause zwischen Trains

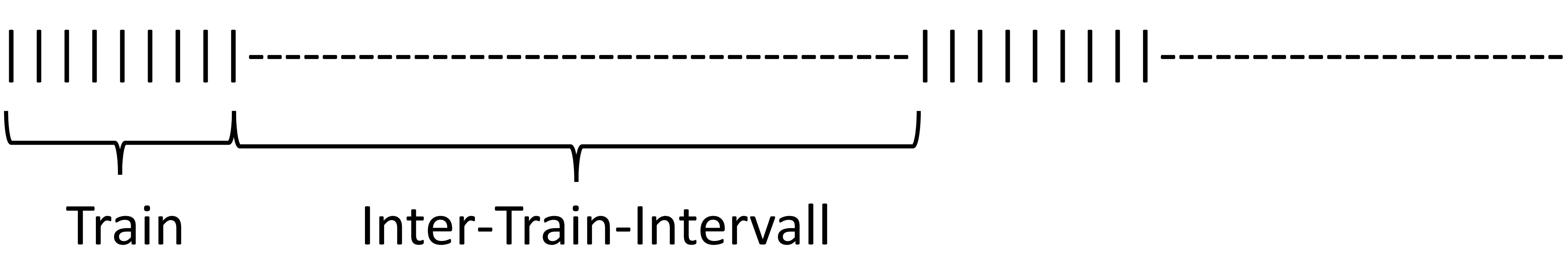

Basics: Stimulationsparameter

Deutsche Gesellschaft für Hirnstimulation in der Psychiatrie e. V.

- Theta-Burst-Stimulation (TBS): Tripletts von Pulsen (50Hz, alle 20ms) in 5Hz-Rhythmus (alle 200ms)
  - kontinuierliche TBS (engl. cTBS): gilt als inhibitorisch
  - intermittierende TBS (iTBS): gilt als exzitatorisch

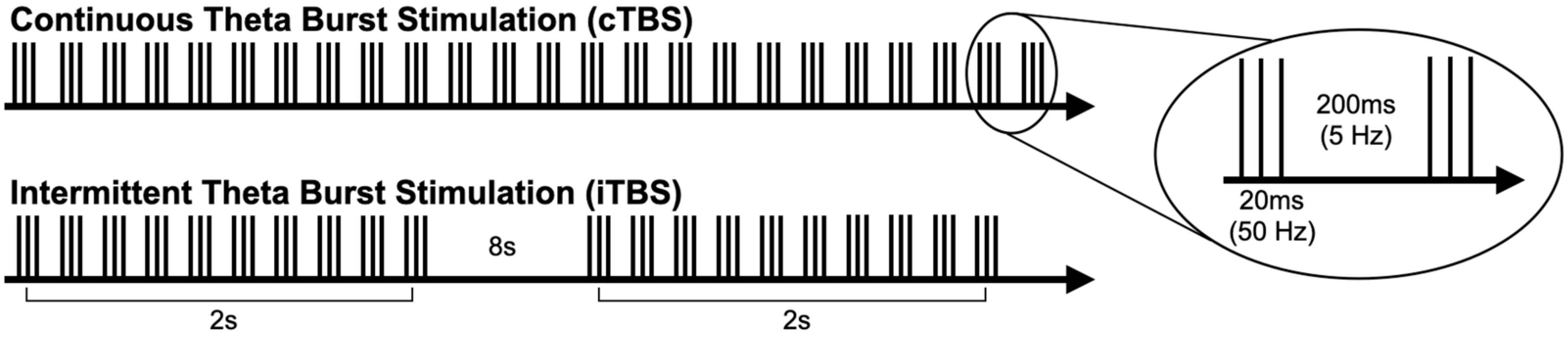

Huang et al. (2005) - doi: 10.1016/j.neuron.2004.12.033

Basics: neurowissenschaftliche Grundlagen

Deutsche Gesellschaft für
Hirnstimulation
in der Psychiatrie e. V.

- Kortikale Erregbarkeit (z.B. Motorschwelle) und Neuroplastizität (Langzeitpotenzierung und Langzeitdepression)

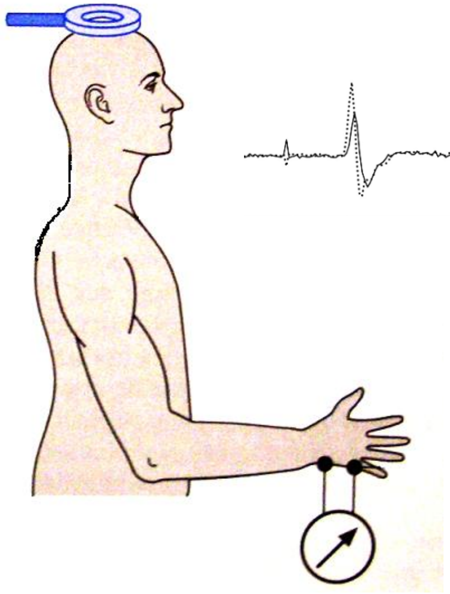

Einzelpulse =
Diagnostik

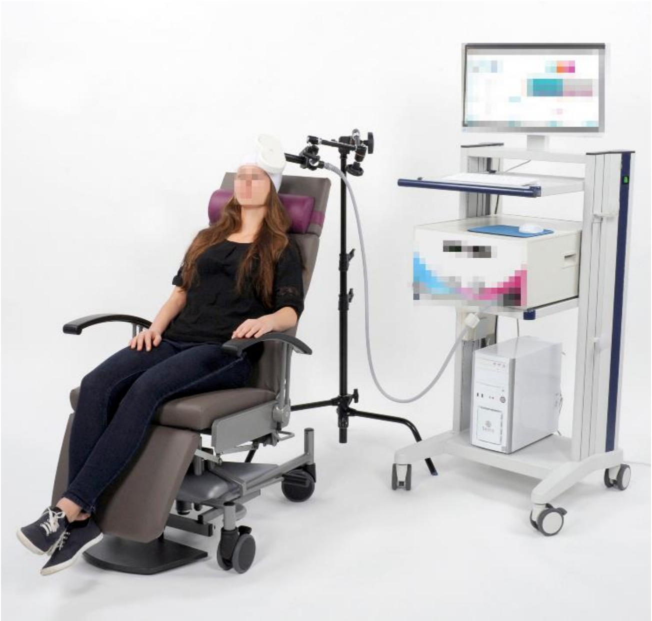

Pulsserien =
repetitive TMS =
Behandlung

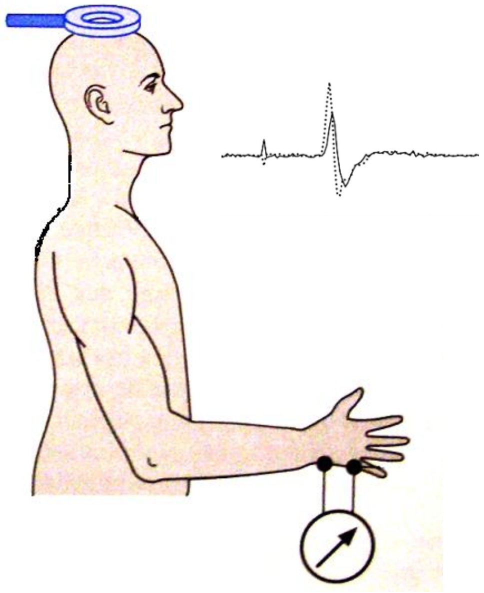

MEP-Veränderung =
Neuromodulatorischer Nacheffekt

Basics: neurowissenschaftliche Grundlagen

Deutsche Gesellschaft für
Hirnstimulation
in der Psychiatrie e. V.

- Kortikale Erregbarkeit (z.B. Motorschwelle) und Neuroplastizität (Langzeitpotenzierung und Langzeitdepression)
- Veränderungen messbar mit neurowissenschaftlichen Verfahren wie EEG und MRT, aber nicht tauglich als Biomarker

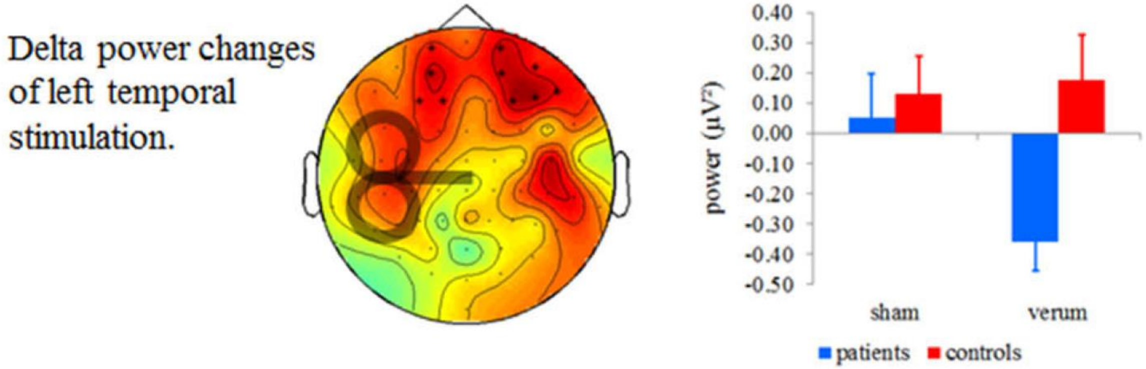

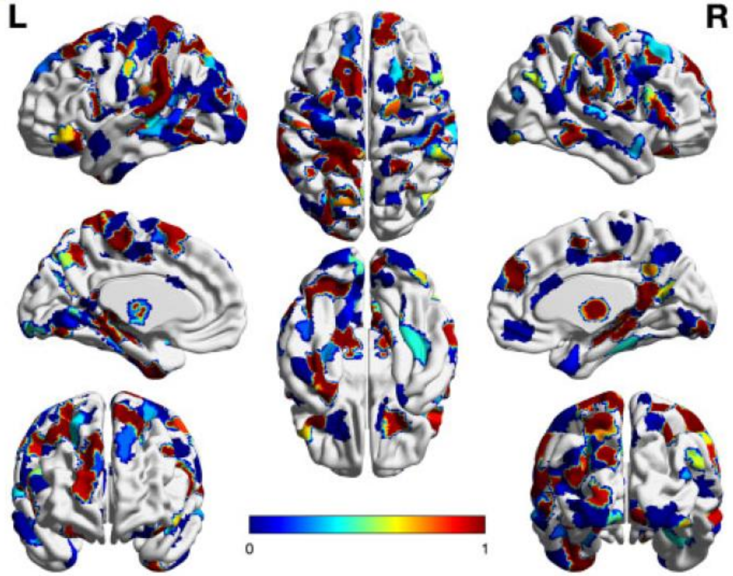

Schecklmann et al. (2015) - doi: 10.3389/fncel.2015.00421
Poeppl et al. (2021) - doi: 10.1093/braincomms/fcab115

Basics: neurowissenschaftliche Grundlagen

Deutsche Gesellschaft für
Hirnstimulation
in der Psychiatrie e. V.

- Kortikale Erregbarkeit (z.B. Motorschwelle) und Neuroplastizität (Langzeitpotenzierung und Langzeitdepression)
- Veränderungen messbar mit neurowissenschaftlichen Verfahren wie EEG und MRT, aber nicht tauglich als Biomarker
- möglicher Wirkmechanismus
  - Depression:
    Dysbalance-/Hypofrontalitäts-Modell

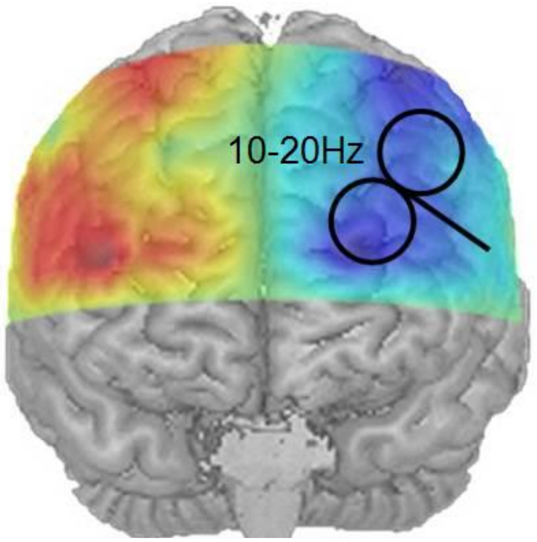

van der Finne et al. (2017) - doi: 10.1016/j.nicl.2017.07.006

Basics: neurowissenschaftliche Grundlagen

Deutsche Gesellschaft für
Hirnstimulation
in der Psychiatrie e. V.

- Kortikale Erregbarkeit (z.B. Motorschwelle) und Neuroplastizität (Langzeitpotenzierung und Langzeitdepression)
- Veränderungen messbar mit neurowissenschaftlichen Verfahren wie EEG und MRT, aber nicht tauglich als Biomarker
- möglicher Wirkmechanismus
  - Depression: Dysbalance-/Hypofrontalitäts-Modell
  - Phantomwahrnehmungen: Überaktivität des auditorischen Kortex

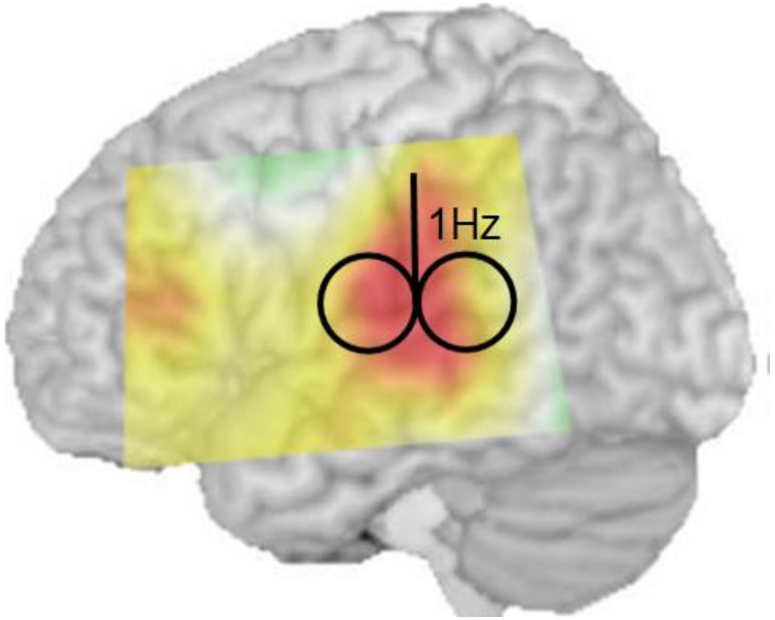

De Ridder et al. (2007) - doi: 10.1007/978-3-211-33081-4_52

Exkurs: Ablauf einer Behandlung

Deutsche Gesellschaft für Hirnstimulation in der Psychiatrie e. V.

- **Ärztliches** Aufklärungsgespräch
- kein EEG oder MRT nötig
- Motorschwelle (Bestimmung der Behandlungsstärke)
- Behandlung (durch Arzt delegierbar, wechselnde Behandler möglich)
- Abschluss-/Zwischengespräch
- ggf. Weiterbehandlung
- ggf. Nachuntersuchung
- ggf. Rückfallprophylaxe

Spulenpositionierung - allgemein

Deutsche Gesellschaft für
Hirnstimulation
in der Psychiatrie e. V.

- entspannte Position des Patienten bei der Behandlung
- Motorschwelle im Sitzen
- Verwendung einer Schmetterlingsspule
- CAVE: je nachdem wie man zum Patienten steht (seitlich stehend, dahinter stehend, oder auch Patient halb-liegend), wirkt die Spulenposition unterschiedlich

  → Abteilung sollte Positionierung des Behandlers zu PatientIn standardisieren (z.B. SOP-Dokument, Training)

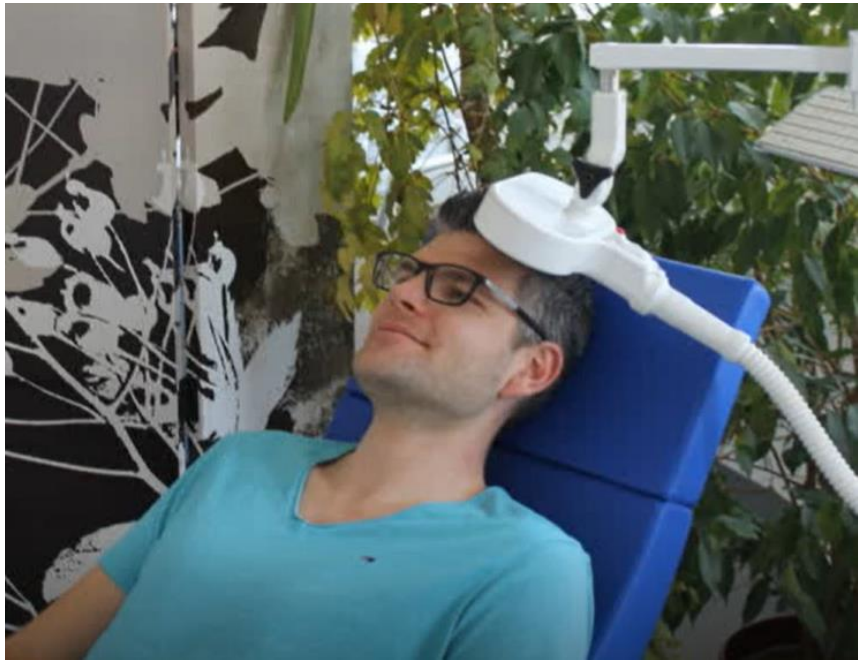

Spulenpositionierung - allgemein

Deutsche Gesellschaft für Hirnstimulation in der Psychiatrie e. V.

- Spule soll tangential zum Kopf orientiert sein
- Spulenmittelpunkt soll aufliegen
- Orientierung der Spule im 90-Grad-Winkel zum Gyrus
- Kontrolle der Spulenposition im Verlauf
- Spulengriff in Richtung Hinterkopf
- Spule mit **<u>minimalem</u>** Druck platzieren (Verletzungsrisiko)

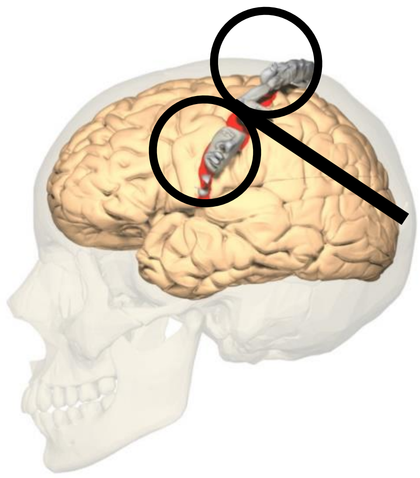

2021, StatPearls Publishing LLC.

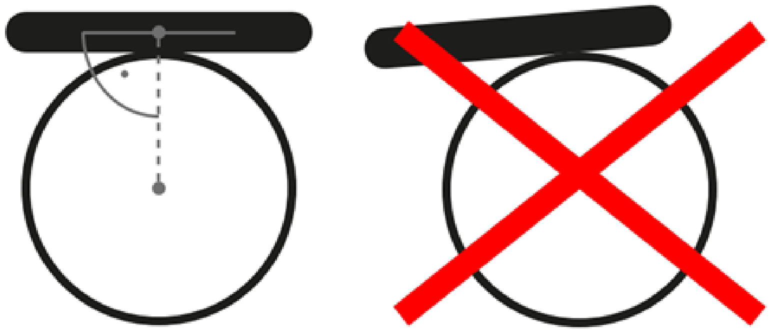

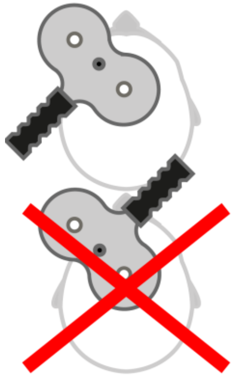

Spulenpositionierung

Deutsche Gesellschaft für
Hirnstimulation
in der Psychiatrie e. V.

Oberflächenbasierte Identifikation des Behandlungsorts

- Nutzt EEG-System Positionen

  (z.B. 10-20 EEG-System)

- Benötigtes Material:
  - Haube für den Patienten
  - Flexibles Geodreieck
  - Maßband
  - Marker-Stift
  - Für „Bequeme“: EEG-Hauben…

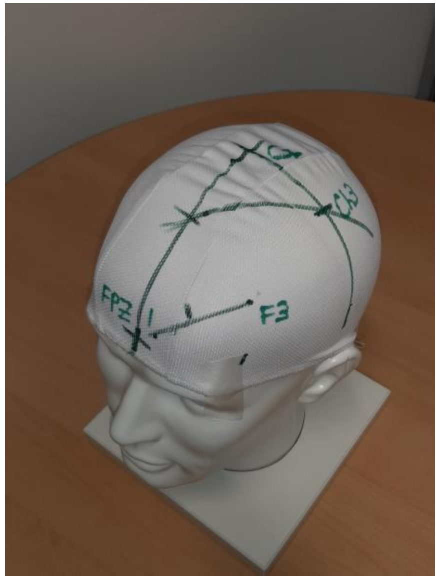

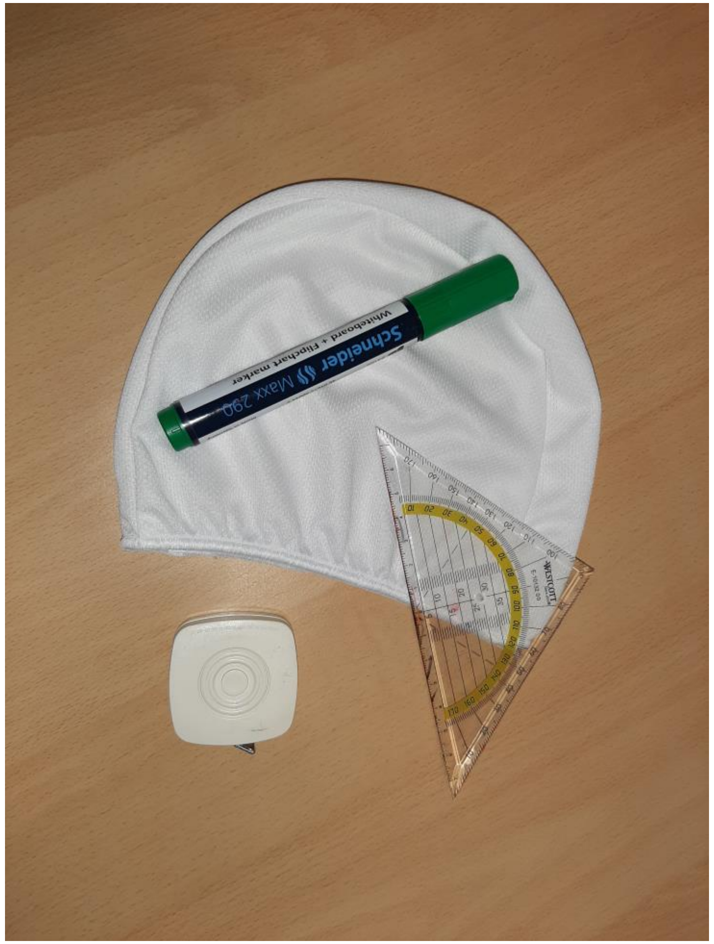

Exkurs: 10-20 EEG-System

Deutsche Gesellschaft für
Hirnstimulation
in der Psychiatrie e. V.

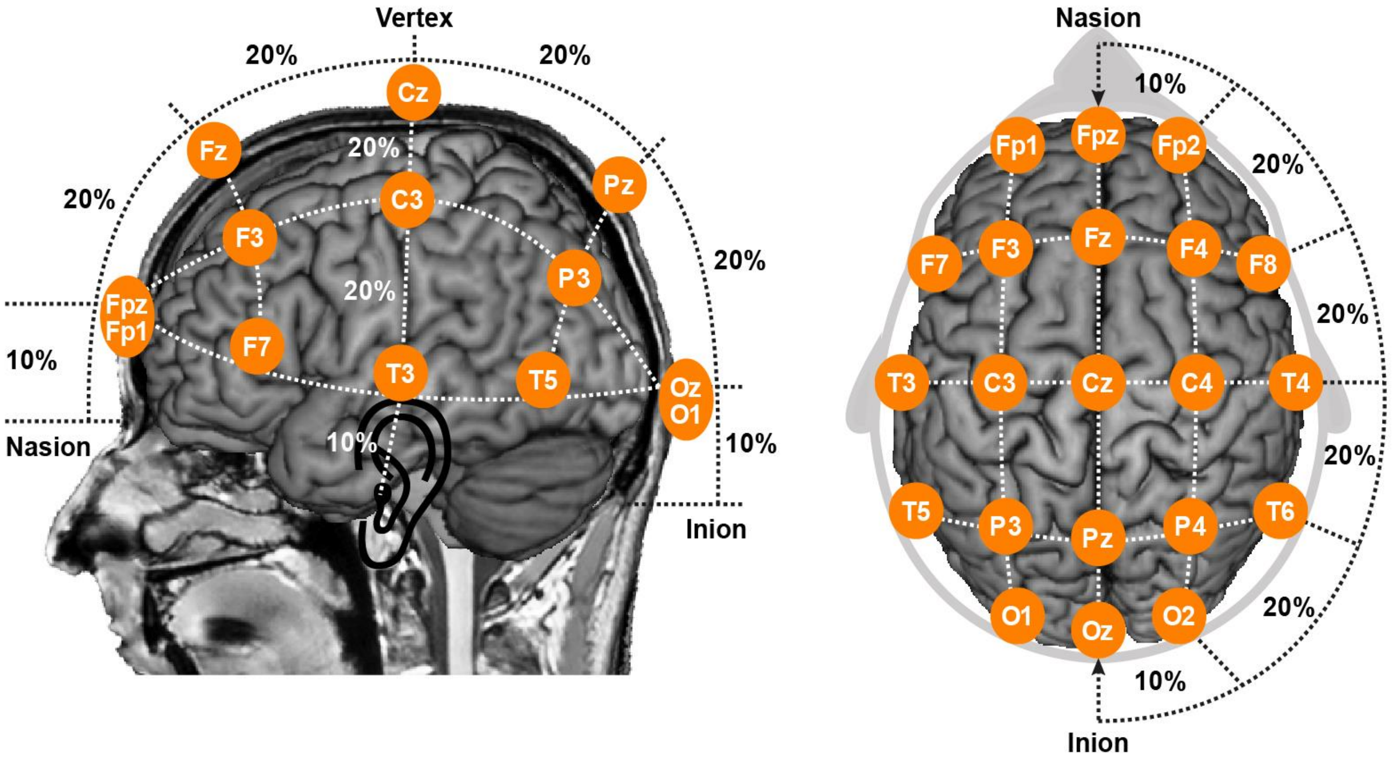

Deutsche Gesellschaft für Hirnstimulation in der Psychiatrie e. V.

Spulenpositionierung

oberflächenbasiert

- an Hand von EEG-Positionen
- praktischer
- evidenz-basiert
- nötig

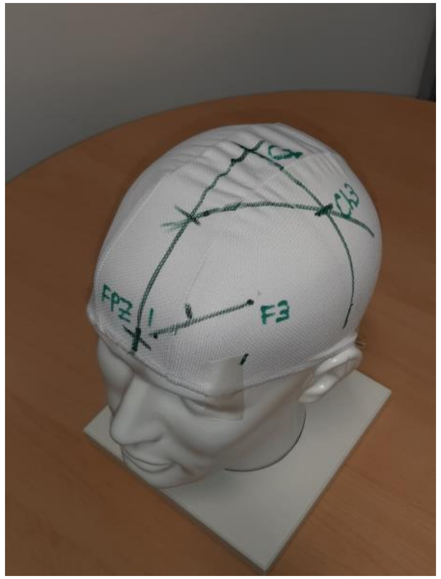

neuronavigiert

- an Hand von anatomischen oder funktionellen Hirnscans
- präziser, reliabler, aufwändiger
- zu wenige überlegene Studien vorhanden
- möglich

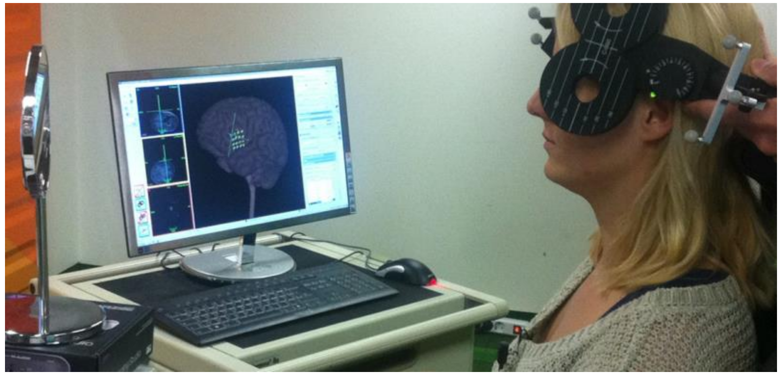

Motorschwelle

Motorschwelle

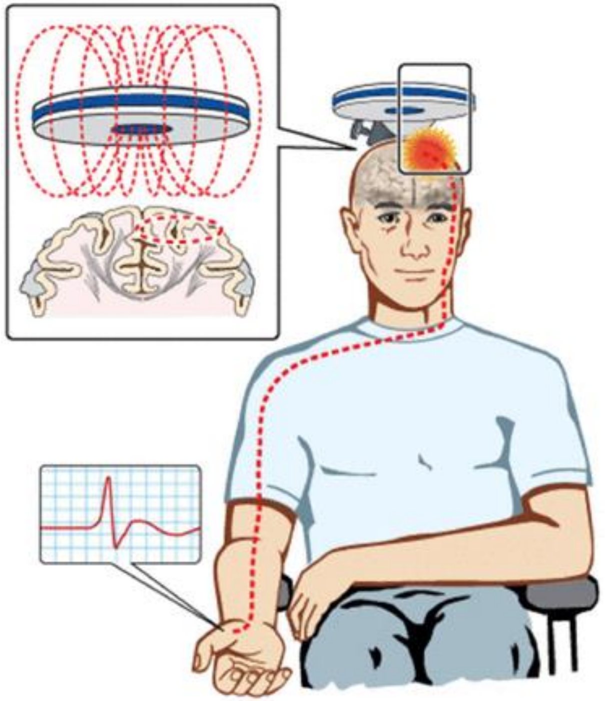

- 2 Schritte: Hot-Spot-Suche und Bestimmung der Schwellenintensität
- Standard: **Ruhemotorschwelle** (RMT) der entspannten Fingermuskeln
  - *(Kleinfingerspreizer, Daumenballen oder Zeigefinger)*
- Standard: Stimulation des Motorkortex auf der Behandlungsseite
  - *z.B. Depressionsbehandlung: linker Motorkortex (Fingermuskeln der rechten Hand)*

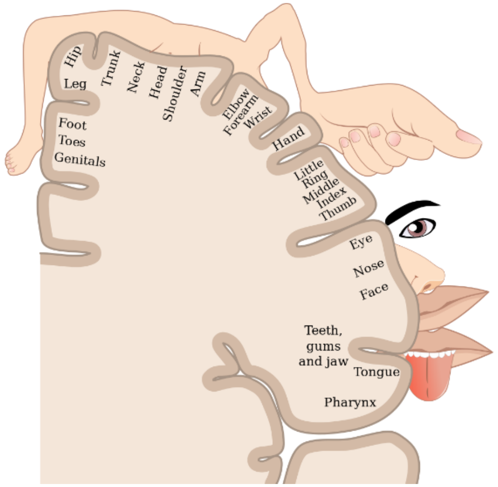

https://commons.wikimedia.org/wiki/File:Sensory_Homunculus-en.svg
Vucic et al. (2012) - doi: 10.1136/jnnp-2012-304019

Motorschwelle

Deutsche Gesellschaft für
Hirnstimulation
in der Psychiatrie e. V.

- Empfohlen: Ableitung des motorisch evozierten Potentials (MEPs) mittels Elektromyographie
  - (bei Nicht-Verfügbarkeit von EMG kann auch visuell Muskelzucken beurteilt werden, aber im Vgl. zur MEP-Detektion ca. 10% höherer Stimulatoroutput nötig)
- MEP: biphasische Welle 20-40 ms nach Puls
- Elektroden: belly-tendon-Montage
  - aktiv: Muskelbauch
  - passiv: Sehnenansatz
  - Erdung: zwischen Elektroden und Kopf (z.B. Styloidspitze der Ulna)

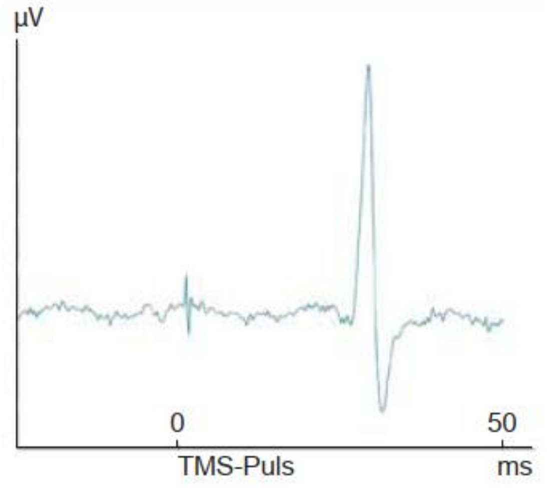

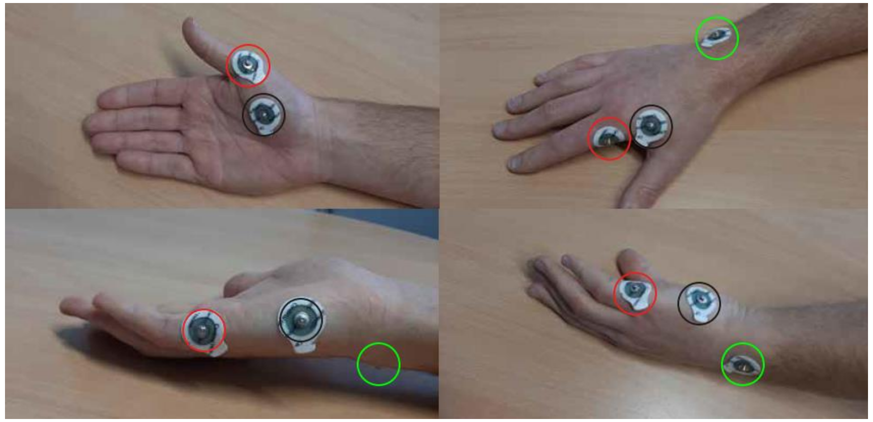

Motorschwelle

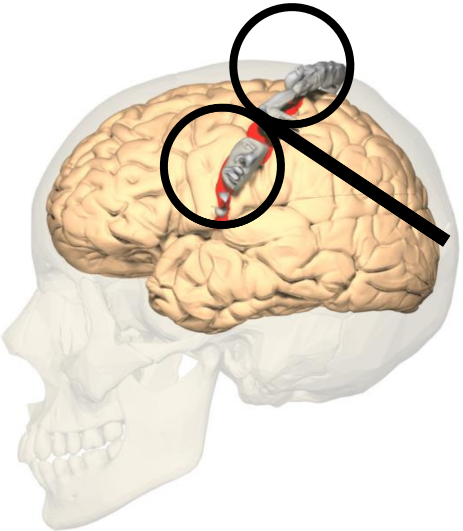

Deutsche Gesellschaft für
Hirnstimulation
in der Psychiatrie e. V.

- Startpunkt Hot-Spot-Suche: C3/C1
- funktionelle Bestimmung anhand der Muskelantwort
- Spulenorientierung im 45-Grad-Winkel zur Mittellinie
- Wie man den 45-Grad-Winkel bestimmt:
  - vom Vertex/Cz ausgehend 5 cm nach lateral (in Richtung Ohr) messen und Punkt markieren
  - vom Vertex/Cz ausgehend 5 cm nach anterior (in Richtung Nase) messen und Punkt markieren
  - Die beiden Punkte verbinden – die resultierende Linie bildet einen 45°-Winkel relativ zur Mittellinie

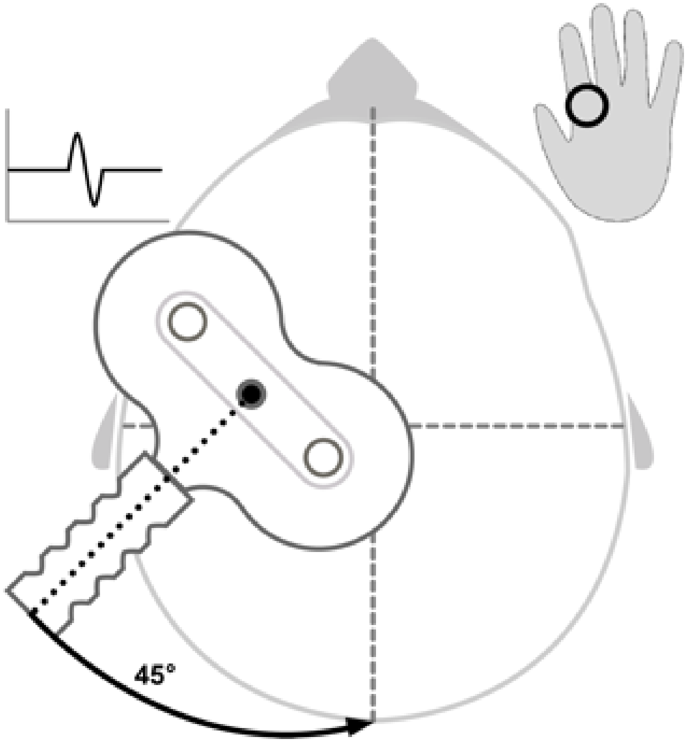

https://www.statpearls.com/ArticleLibrary/viewarticle/29224

Deutsche Gesellschaft für
Hirnstimulation
in der Psychiatrie e. V.

Motorschwelle

- Bestimmung einmalig vor der Behandlung
- Monitoring bei Einnahme relevanter psychotroper Medikation (z.B. Benzodiazepine)
- Hotspot-Suche mit überschwelliger Intensität
- Algorithmen zur Motorschwellenbestimmung:
  - pragmatisch: MEPs bei 4 aus 8 Pulsen (50µV oder Zucken)
  - „Schwellenjagd“ (Computer-gestützter Algorithmus)

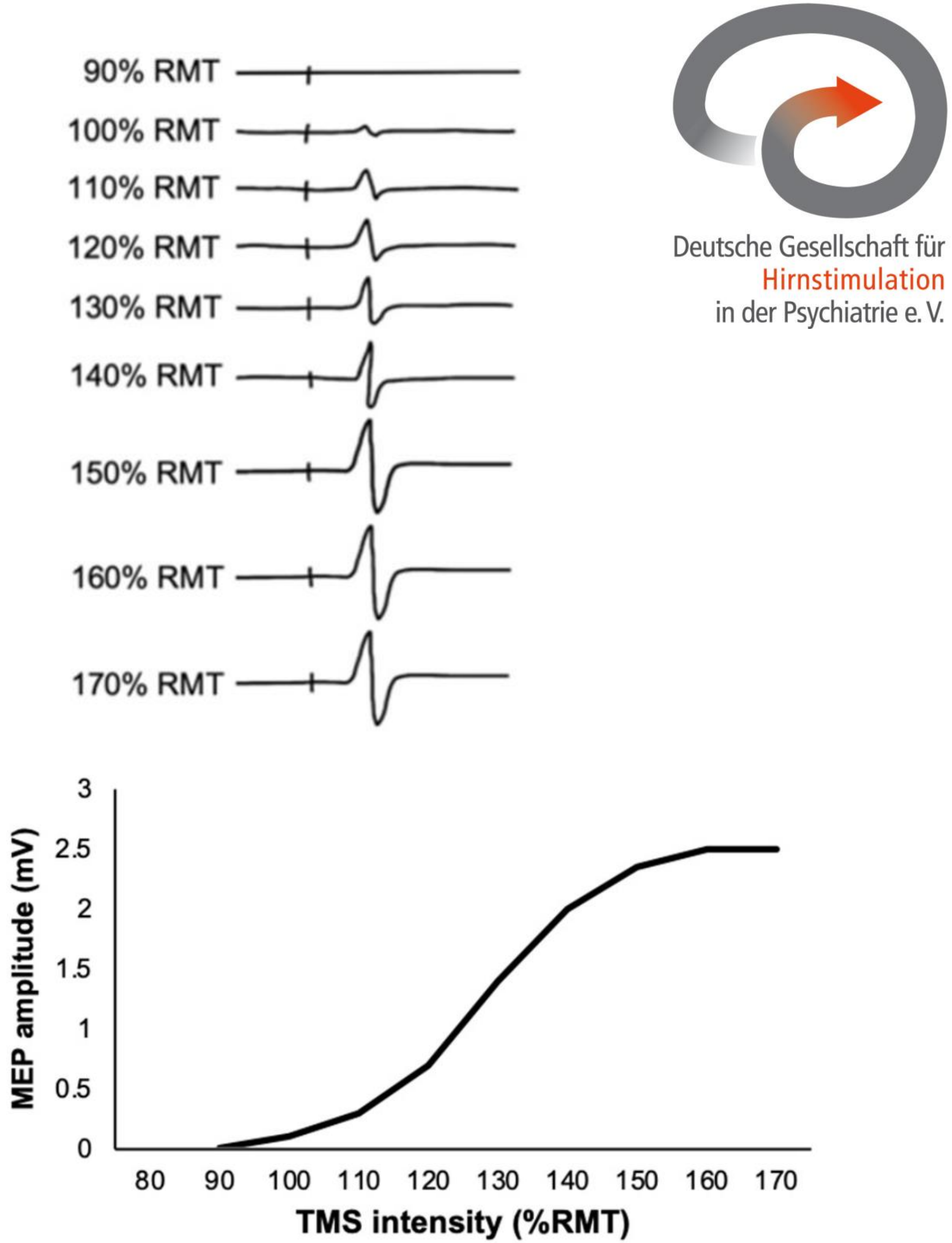

Turco & Nelson (2021) - doi: 10.3389/fnrgo.2021.679033

Motor threshold

Deutsche Gesellschaft für
Hirnstimulation
in der Psychiatrie e. V.

- **Aktive Motorschwelle** (AMT):
  - Eventuelle Alternative bei Patienten mit erhöhtem Muskeltonus (z.B. Tremor, Spastik, Dystonie, or Schlaganfall- bzw. Demenz-bezogene Phänomene)
  - Häufig genutzte Methode bei Theta-Burst-Stimulationsprotokollen
  - Typischerweise ist die AMT niedriger als die RMT (in %MSO)
  - Vorgehen:
    - Ähnlich RMT-Bestimmung, jedoch mit voraktivierten Muskeln (z.B. der Finger)
    - Anwendung eines Schwellen-Algorithmus (Schwelle von 200 µV oder Muskelzucken)
  - CAVE: Problem der Standardisierung des Vorgehens (z.B. ohne Dynamometer)

Sen et al. (2017) – doi: 10.1371/journal.pone.0186007
Turi et al. (2021) – doi: 10.1111/ejn.15195

Motor threshold

Deutsche Gesellschaft für Hirnstimulation in der Psychiatrie e. V.

- Weitere Hinweise und zu beachtende Aspekte:
  - Vermeidung von starkem Druck auf den Kopf während der Spulenplatzierung und -bewegung (z. B. bei der Hotspot-Suche)
  - empfehlenswert: Teammitglied mit Erfahrung in EMG-Messungen
    - Standardisierte Hautvorbereitung für die Platzierung der EMG-Elektroden
    - Überprüfung der EMG-Signalqualität in der Ausgangslage (Baseline-Check)
    - Probleme bei zuverlässigen EMG-Ableitungen unter bestimmten Bedingungen (z. B. Ulnarisrinnen-Syndrom, periphere Neuropathie, Radikulopathie etc.)

Behandlung der Depression

Deutsche Gesellschaft für
Hirnstimulation
in der Psychiatrie e. V.

Behandlung der Depression

- DLPFC: BEAM-F3 (früher 5/6cm-Regel)
- „adjusted“ BEAM-F3 wenig untersucht

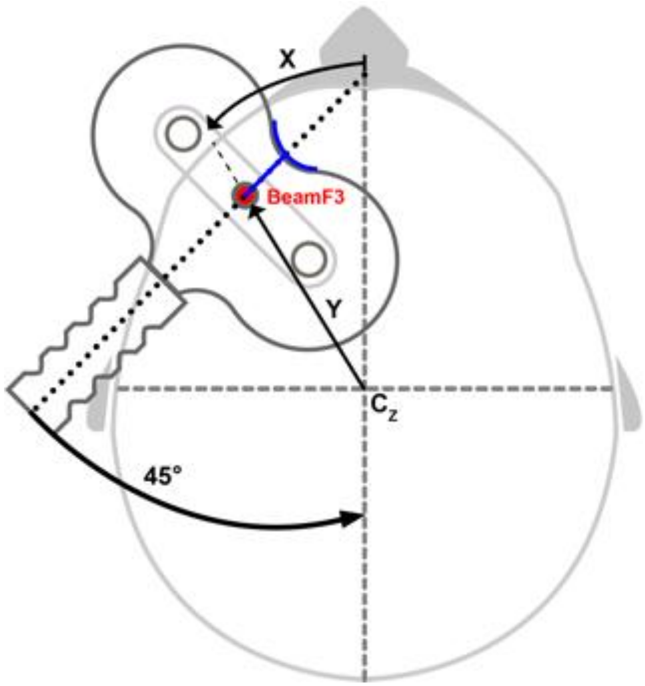

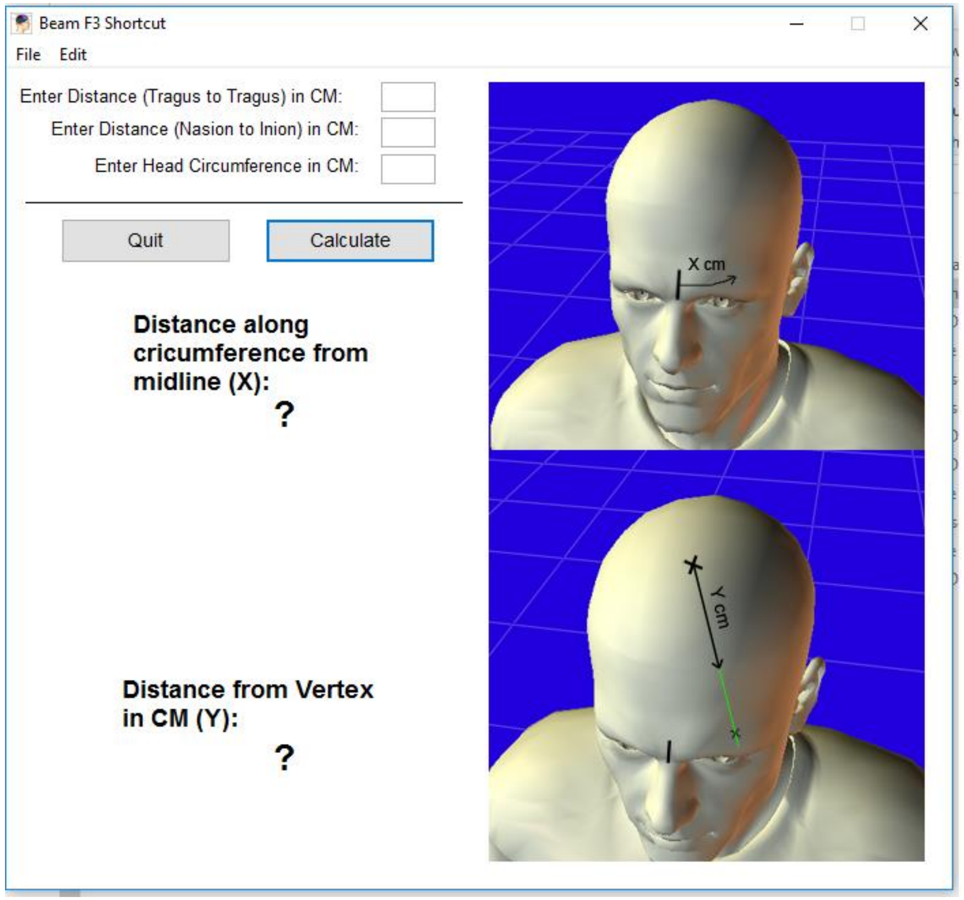

Beam et al. (2009) - doi: 10.1016/j.brs.2008.09.006

Behandlung der Depression

Deutsche Gesellschaft für Hirnstimulation in der Psychiatrie e. V.

	Behandlung	Deutsche Leitlinien	Europäische Leitlinie	Zulassungen	Studienlage
**Unipolare/ bipolare Depression**	F3, 10/20Hz, 100-120%, 1500-3000 Pulse, 20-30 Sitzungen (*alternativ: F3, iTBS, 80%, 600 Pulse, 20-30 Sitzungen*) (Hebel et al., 2022)	Sollte-Empfehlung bei Therapie-Resistenz<br>Kann-Empfehlung nach nicht erfolgreicher Monotherapie	definitiv wirksam	FDA, CE	zahlreiche positive Meta-Analysen vorhanden

Ansprechrate: ca. 10-50%!
Es werden auch Stimulationsintensitäten von 80% berichtet!

Behandlung der Depression

Deutsche Gesellschaft für Hirnstimulation in der Psychiatrie e. V.

NATIONALE VERSORGUNGS LEITLINIEN

Programm für Nationale VersorgungsLeitlinien

Repetive transkranielle Magnetstimulation

Empfehlung	Empfehlungs-grad
**7-16 | neu 2022**<br>Bei Patient*innen, die nicht auf eine Monotherapie mit Antidepressiva ansprechen, kann eine Augmentation mit repetitiver transkranieller Magnetstimulation (rTMS) angeboten werden.	⇔
**7-29 | modifiziert 2022**<br>Eine repetitive transkranielle Magnetstimulation (rTMS) sollte bei therapieresistenten depressiven Episoden angeboten werden.	⇑
**7-30 | neu 2022**<br>Die Auswahl der rTMS-Methode (Stimulationsort und -art) soll durch ein spezialisiertes Zentrum erfolgen.	⇑⇑

© äzq 2022

Behandlung der Depression

Deutsche Gesellschaft für
Hirnstimulation
in der Psychiatrie e. V.

- auch bei bipolarer Störung möglich
- niedrigfrequente rechtsfrontale Stimulation möglich
- bifrontale Stimulation möglich
- auch iTBS möglich
- Wirksamkeit unabhängig vom Alter

Aufklärung

Aufklärung

Deutsche Gesellschaft für Hirnstimulation in der Psychiatrie e. V.

- mündliche Aufklärung durch **ärztliches** Personal (schriftliches Aufklärungsdokument als Hilfsmittel)
- Dokumentation der Aufklärung
- Bedenkzeit einplanen (abhängig von Komplexität des Falls)
- Information nicht nur über Erfolgsaussichten, Ablauf, Risiken und Nebenwirkungen, sondern auch über mögliche Alternativen
- Indikationsstellung durch **fachärztliches** Personal (Psychiatrie, Nervenheilkunde mit entsprechender Qualifikation im Bereich der Gehirnstimulationsverfahren, entsprechend der Weiterbildungsordnung für Ärzte oder der Vorschläge der Fachgesellschaften DGPPN und/oder DGHP)

Aufklärung

Deutsche Gesellschaft für Hirnstimulation in der Psychiatrie e. V.

- im Falle fehlender Zulassung Dokumentation der Off-Label-Aufklärung
- bei Depression mit komorbiden Störungen sollte die Depression als Indikation betont werden und die Behandlung mit einem Depressionsprotokoll erfolgen
- Formulierungshilfe: *„Es erfolgte die Vorstellung zur rTMS-Behandlung bei einer vorbeschriebenen Depression und unzureichender Besserung unter der bisherigen multimodalen Therapie. Die/Der Patient/in wurde mündlich und schriftlich über Indikation, Wirkung und Nebenwirkungen aufgeklärt. Kontraindikationen liegen keine vor. Ziel der Behandlung ist die Besserung der depressiven Symptomatik. … Bedenkzeit …"*

Sicherheitsaspekte

Deutsche Gesellschaft für
Hirnstimulation
in der Psychiatrie e. V.

Konsenspapiere europäischer Experten zum Umgang mit TMS

- **generell:** Immer Risiko-Nutzen Abwägung notwendig (wie bei allen therapeutischen Entscheidungen)
- Dokumentation dieser Abwägung
- Patient über Risiken und erwarteten Nutzen informieren
- bei besonderen Patientengruppen verschiebt sich Risiko-Nutzen-Abwägung zugunsten Risiko, TMS meist dennoch möglich

Wassermann (1998) - doi: 10.1016/s0168-5597(97)00096-8
Rossi et al. (2009) - doi: 10.1016/j.clinph.2009.08.016
Rossi et al. (2021) - doi:10.1016/j.clinph.2020.10.003

Sicherheitsaspekte

- Kontraindikationen
- Nebenwirkungen
- besondere Settings
- sichere Behandlungsparameter

Kontraindikationen

Deutsche Gesellschaft für
Hirnstimulation
in der Psychiatrie e. V.

- metallische Implantate
- Elektrische Implantate (z.B. Herzschrittmacher, Insulinpumpe)
- Epilepsie und neurologische Erkrankungen

- absolute vs. relative Kontraindikationen

Implantierte Geräte

Deutsche Gesellschaft für Hirnstimulation in der Psychiatrie e. V.

- Geräte, die ferromagnetisch sind, elektrisch betrieben werden und/oder für ihre Aktivität auf Detektion/Generation elektrischer Pulse angewiesen sind
- individuelle Risikoabschätzung (Material, Abstand, Relevanz und Dringlichkeit der Indikation)
- metallhaltige Tätowierungen direkt unterhalb der Spule kritisch
- Zahnimplantate und Piercings unproblematisch
- MRT-Tauglichkeit lediglich als **Hinweis**
- Verantwortung bleibt beim Anwender!

Zerebrale Vorschädigung

Deutsche Gesellschaft für Hirnstimulation in der Psychiatrie e. V.

- grober, für die Praxis wenig hilfreicher Überbegriff
- Art, Ausmaß, Lokalisation entscheidend
- Problem 1: Anfallsrisiko
- Problem 2: verminderte Effektivität
- Einzelfallentscheidung!

Mögliche Nebenwirkungen

Deutsche Gesellschaft für Hirnstimulation in der Psychiatrie e. V.

nach Häufigkeit sortiert

- lokale sensorische Missempfindungen (Nerven in Kopfhaut und Kopfmuskulatur)
- Kopfschmerzen, Nackenschmerzen (i.d.R. selbstlimitierend, gutes Ansprechen auf Schmerzmittel)
- Motorische Entäußerungen des N. facialis (Auge/Stirn/Nase/Ohr)
- Schwindel

Mögliche Nebenwirkungen

Deutsche Gesellschaft für
Hirnstimulation
in der Psychiatrie e. V.

Problematischer, aber deutlich seltener:

- akustische Nebenwirkungen
- Switch in die Manie
- induzierte Anfälle und Synkopen

Induzierte Anfälle und Synkopen

Deutsche Gesellschaft für Hirnstimulation in der Psychiatrie e. V.

- induzierter Anfall: in der Praxis geringes Risiko (ca. 2-8/100.000 Behandlungen, nur 10% der Hintergrundinzidenz von ca. 50/100.000)
- wohl Bias durch Patientenauswahl und reporting bias
- in Risikopatienten: ca. 30/100.000 (>60% in erster Session)
- viele Ko-Faktoren: Provokationsfaktoren, Drogen, Medikamente(!), Schlaf usw.
- für Aufklärung entscheidend: **Folgen** eines Anfalles, Unterschied Epilepsie vs. induzierter Anfall

Lerner et al. (2019) - doi:10.1016/j.clinph.2019.03.016

Induzierte Anfälle und Synkopen

Deutsche Gesellschaft für Hirnstimulation in der Psychiatrie e. V.

- Kenntnisse im Erkennen und Erstbehandlung, Verfügbarkeit von Medikamenten und ärztlichem Personal
- viel häufiger, aber auch selten: (prä)synkopale Ereignisse - Unterschied erkennen (Reorientierungsphase)!
- „basic life support“
- Equipment: Benzodiazepine, Notfall-Alarmierungssystem

Lokale Nebenwirkungen

Deutsche Gesellschaft für Hirnstimulation in der Psychiatrie e. V.

- klinisch benigne, aber für die Praxis und Patientenbetreuung relevanteste Nebenwirkungen
  - lokale Kopfschmerzen (Spannungskopfschmerzen)
  - Behandlung über Kopfmuskulatur am unangenehmsten (Motorschwelle vs. frontale Stimulation)
  - bei hochindividueller Schmerzverarbeitung klinisches Fingerspitzengefühl notwendig …

Akustische und elektromagnetische Auswirkungen

Deutsche Gesellschaft für Hirnstimulation in der Psychiatrie e. V.

- offensichtliche akustische Effekte (bis zu maximal 125dB in 25cm, bis zu 139dB in 5cm Entfernung)
- Gehörschutz immer anbieten und Ablehnung entsprechend dokumentieren (nicht ausschließlich, aber vor allem: Tinnitusbehandlung)
- Langzeiteffekte elektromagnetischer Exposition: bisher keine Berichte dazu, relevant weniger für Patienten als für die Behandler

Switch-Risiko und Suizidalität

Deutsche Gesellschaft für
Hirnstimulation
in der Psychiatrie e. V.

- Switch in die Manie, Zunahme der Suizidalität oder allgemein Verschlechterung der Symptome kommt in der Praxis vor
- klinisch und forensisch relevante, praktisch gesehen aber TMS-unspezifische Effekte
- weiterer Grund für die Bedeutung der Indikationsstellung, Aufklärung und Abschlussbeurteilung(!) durch psychiatrisch geschultes ärztliches/psychologisches Fachpersonal

„TMS Dip“

Deutsche Gesellschaft für Hirnstimulation in der Psychiatrie e. V.

- „Verschlechterung“ der Symptomatik nach 2 Wochen (Mechanismus: evtl. Erwartungshaltung der Patienten und Beschäftigung mit Symptomatik)
- kein notwendiger Grund für Beendigung der Therapie, da vorgeschlagener Zeitraum mind. 3 Wochen sind

Kognitive Nebenwirkungen

Deutsche Gesellschaft für
Hirnstimulation
in der Psychiatrie e. V.

- relevant, da oft gefragt - Evidenz aber gering bis fehlend
- oft begriffliche Vermengung mit der EKT bei Patienten
- aus der Literatur keine Hinweise, in der Praxis nicht berichtet, grundsätzlich natürlich nicht auszuschließen
- viele Confounder (Medikamente, Depression an sich)
- Mehrheit der Studien beschreibt eine Verbesserung der Kognition (Rossi et al., 2021)!
- Autofahren möglich, keine Wartezeit nach Sitzung nötig

Rossi et al. (2021) - doi: 10.1016/j.clinph.2020.10.003

Besondere Settings

Deutsche Gesellschaft für
Hirnstimulation
in der Psychiatrie e. V.

- Schwangerschaft und Stillzeit
- Medikation

Schwangerschaft und Stillzeit

Deutsche Gesellschaft für Hirnstimulation in der Psychiatrie e. V.

- 3 kontrollierte Studien, mehrere Dutzend Fallberichte
- Einzelfallentscheidung
- physikalisch wenig Anhalt für Schädigungspotential (≠Medikamente)
- CAVE: publication bias
- CAVE: emotional besonders aufgeladene und forensisch relevante Situation, daher besondere Sorgfalt bei der Dokumentation erforderlich (insbesondere bei off-label Anwendung)
- CAVE: explizite Aufklärung bzgl. möglicheer Schädigung des Kinds im Falle eines induzierten epileptischen Anfalls

Ko-Medikation

- TMS gut mit anderen Therapien kombinierbar, keine besondere Rücksicht auf Medikation bis auf Benzodiazepine auf Grund der Änderung der Krampfschwelle
- Wunsch: Medikation stabil belassen
- im Fokus bisher insbesondere neurotrope Medikation
- Antikonvulsiva und Phasenprophylaktika: wenig Evidenz vorhanden
- Antipsychotika wohl mit abschwächendem Effekt
- Benzodiazepine wohl mit abschwächendem Effekt
- CAVE: methodische Schwierigkeiten der Studien, da kaum prospektiv randomisiert möglich, viele potentielle Confounder

Sichere Behandlungsparameter

Deutsche Gesellschaft für Hirnstimulation in der Psychiatrie e. V.

- Sicherheitsrichtlinien nach Wassermann et al. (1998) und Rossi et al. (2009)
- **CAVE: auf Herstellerangaben in den Handbüchern achten**
- Sicherheit abhängig von Intensität, Frequenz und Inter-Train-Intervallen
  - Ab 60% Stimulator-Output wird es schmerzhaft!
  - Bei hohen Frequenzen sind Pausen nötig!
  - Induzierte Anfälle werden sowohl bei Einzelpulsen, niedrigen Frequenzen sowie auch Gesunden berichtet und nicht nur bei vermeintlich gefährlichen hochfrequenten und TBS-Stimulationen!

Wassermann (1998) - doi: 10.1016/s0168-5597(97)00096-8
Rossi et al. (2009) - doi: 10.1016/j.clinph.2009.08.016

Deutsche Gesellschaft für
Hirnstimulation
in der Psychiatrie e. V.

Sichere Behandlungsparameter

- sichere Dauer eines Trains (außerhalb Motorkortex)

Maximum safe duration (expressed in seconds) of single trains of rTMS. Safety defined as absence of seizure, spread of excitation or afterdischarge of EMG activity. Numbers preceded by > are longest duration tested. Consensus has been reached for this table.

Frequency (Hz)	Intensity (% of MT)				
	90%	100%	110%	120%	130%
1	>1800[a]	>1800	>1800	>360	>50
5	>10	>10	>10	>10	>10
10	>5	>5	>5	4.2	2.9
20	2.05	2.05	1.6	1.0	0.55
25	1.28	1.28	0.84	0.4	0.24

[a] In Japan, up to 5000 pulses have been applied without safety problems (communication of Y. Ugawa).

Sichere Behandlungsparameter

Deutsche Gesellschaft für Hirnstimulation in der Psychiatrie e. V.

- sichere Mindestdauer der Inter-Train-Intervalle (Motorkortex)

Adapted from Table 4 (Part A) and Table 3 (part B) of Chen et al., 1997, with permission from the authors. Safety recommendations for inter-train intervals for 10 trains at <20 Hz. The maximum duration of pulses for individual rTMS trains at each stimulus intensity should not exceed those listed in the Part B of the table. A consensus has been reached in adopting this table at this point. However, there is a need to extend these investigations and provide more detailed guidelines that may apply also to non-motor areas.

Inter-train interval (ms)	Stimulus intensity (% of MT)			
	100%	105%	110%	120%
Part A				
5000	Safe	Safe	Safe	Insufficient data
1000	Unsafe (EMG spread after 3 trains)	Unsafe[a]	Unsafe (EMG spread after 2 trains)	Unsafe (EMG spread after 2 trains)
250	Unsafe[a]	Unsafe[a]	Unsafe (EMG spread after 2 trains)	Unsafe (EMG spread after 3 trains)

Frequency (Hz)	100%		110%		120%		130%	
	Duration (s)/pulses		Duration (s)/pulses		Duration (s)/pulses		Duration (s)/pulses	
Part B								
1	>270	>270	>270	>270	>180	>180	50	50
5	10	50	10	50	10	50	10	50
10	5	50	5	50	3.2	32	2.2	22
20	1.5	30	1.2	24	0.8	16	0.4	8
25	1.0	25	0.7	17	0.3	7	0.2	5

[a] These stimulus parameters are considered unsafe because adverse events occurred with stimulation of lower intensity or longer inter-train interval, but no adverse effects were observed with these parameters.

Sichere Behandlungsparameter

Deutsche Gesellschaft für
Hirnstimulation
in der Psychiatrie e. V.

Theta-Burst-Stimulation

Published TBS (biphasic pulses) and QPS (monophasic pulses) protocols on normal subjects. No significant side effects reported, apart vagal reactions after prefrontal cortex stimulation. Consensus reached for this table.

	Pulses in the burst	Total train pulses	Intensity	Stimulation site
"Standard" cTBS (following Huang et al. 2005)	3 at 50 Hz, repeated at 5 Hz	600 (40 s)	80% of active MT	Motor cortex, PFC[c]
Silvanto et al. 2007	8 at 40 Hz, repeated every 1.8 s	200	60% of the maximal stimulator output	Visual cortex
Nyffeler et al. 2006[a]	3 at 30 Hz, repeated at 10 Hz	200	80% of resting MT	Frontal eye fields
"Standard" iTBS protocols (following Huang et al. 2005)	3 at 50 Hz, repeated at 5 Hz for 2 s	600	80% of active MT	Motor cortex, PFC[c]
QPS [b] (following Hamda et al., 2008)	4 (ISI ranging 1.5 ms–1.25 s), repeated every 5 s	1440	90% of active MT	Motor cortex

[a] Also repeated TBS in the same session (at 5, 15, 60, 75 min).
[b] 2000 maximal total pulse number per day; highest intensity used resting MT (Y. Ugawa, personal communication).
[c] PFC = prefrontal cortex (Grossheinrich et al. 2009).

Sichere Behandlungsparameter

Deutsche Gesellschaft für Hirnstimulation in der Psychiatrie e. V.

ABER (Rossi et al., 2021)!

„Despite such variety, as reviewed for these guidelines, neither seizure occurrence nor other AEs emerged consistently, thus indicating that whatever the protocol of intervention, the technique can be considered basically safe. Therefore, we have decided not to provide a formal update of the previous safety tables, and that, instead, we propose ''operational guidelines''. Clearly, the parameters of stimulation used for MST [magnetic seizure therapy] should not be exceeded.

The usual lowest parameters of stimulation to induce seizures during MST are 100% of maximal stimulator output (at least for these commercially available devices), frequency of 25 Hz, delivered in a single train lasting up to 10 s. Therefore, every combination of intensity/frequency/duration of conventional rTMS treatment (when seizure induction is not the goal) must remain well below this combination of parameters.“

Rossi et al. (2021) - doi: 10.1016/j.clinph.2020.10.003

Vergütung

Deutsche Gesellschaft für
Hirnstimulation
in der Psychiatrie e. V.

Vergütung

Deutsche Gesellschaft für Hirnstimulation in der Psychiatrie e. V.

	GKV	PKV und Selbstzahlende
stationär	Zusatzentgelte<br>• Dokumentation, Indikation, Facharzt<br>• rTMS Grund- und Therapieleistung	Zusatzentgelte
ambulant	deutschlandweiter Fleckenteppich<br><br>Bayern: Ziffern für entsprechende Berufsgruppen und Geräte	GOÄ<br><br>Kostenvoranschlag bzw. Behandlungsvertrag

Zusatzentgelte (Stand: 2024)

Deutsche Gesellschaft für Hirnstimulation in der Psychiatrie e. V.

- rTMS-Grundleistung (ZP75.01, OPS: 8-632.0, 124.17 Euro): enthält Aufklärung, Motorschwelle und erste Behandlung, für den ersten Behandlungstag zu kodieren; Dokumentation und Abrechnung der Aufklärung separat (<25 Minuten)
- rTMS-Therapiesitzung (ZP75.02, OPS: 8-632.1; 74.05 Euro): evtl. mehrfach am Tag möglich
- Entgelte seit 2021 möglich, deshalb MDK-Prüfungen relevant, die regional unterschiedlich ausfallen können
- Wichtig bei der Dokumentation und bei Ausstellung von Kostenvoranschlägen: Therapieresistenz und Schwere der Depression benennen, auf Notwendigkeit der TMS explizit hinweisen

Zusatzentgelte

Deutsche Gesellschaft für Hirnstimulation in der Psychiatrie e. V.

ICD-Code OPS Suche

ICD OPS Impressum

OPS Suche

OPS-2024 Systematik online lesen

OPS-2024 > 8 > 8-63...8-66 > 8-63 > 8-632

8-63 Elektrostimulation des Nervensystems

8-632 Repetitive transkranielle Magnetstimulation [rTMS]

8-632.0 Grundleistung

Info: Zur Grundleistung gehören die fachärztliche Indikationsstellung, die Aufklärung, die Planung und die Durchführung der ersten Therapiesitzung mit repetitiver transkranieller Magnetstimulation.
Diese ist nicht gesondert zu kodieren
Dieser Kode ist nur einmal pro stationären Aufenthalt anzugeben

8-632.1 Therapiesitzung

Inkl.: Erhaltungs-rTMS

Info: Dieser Kode ist unabhängig von der Gesamtzahl der Stimulationen einmal pro Therapiesitzung anzugeben

8-632.y N.n.bez.

PKV und Selbstzahlende

Deutsche Gesellschaft für
Hirnstimulation
in der Psychiatrie e. V.

- Kostenvoranschlag oder Behandlungsvertrag
- Evidenzlage, Verweis auf Leitlinien und Nichtansprechen des Falls darstellen
- Abrechnung nach GOÄ: mögliche Ziffern für Motorschwelle und Behandlung
  - 839a (elektromyographische Untersuchung, 93.84 Euro)
  - 828 (evozierte Potentiale, 81.11 Euro)

Transkranielle Magnetstimulation

Deutsche Gesellschaft für Hirnstimulation in der Psychiatrie e.V. (DGHP)

Hands-On-Workshop Refresher April 2025

Deutsche Gesellschaft für
Hirnstimulation
in der Psychiatrie e. V.

Übersicht (1.5h Theorie & 1.5h Praxis)

Deutsche Gesellschaft für Hirnstimulation in der Psychiatrie e. V.

Was?	Wo?	Wann?
Theorie: Indikationen und Behandlungsprotokolle, Akzelerierte Behandlung, Aufrechterhaltung und Rückfallprophylaxe, Fragen		1.5 Stunden
Hands-On		1.5 Stunden
Abschluss		

Indikationen und Behandlungsprotokolle

Indikationen und Behandlungsprotokolle

Deutsche Gesellschaft für Hirnstimulation in der Psychiatrie e. V.

	Behandlung	Deutsche Leitlinien	Europä-ische Leitlinie	Zulass-ungen	Studienlage
**Unipolare/ bipolare Depression**	F3, 10/20Hz, 100-120%, 1500-3000 Pulse, 20-30 Sitzungen (*alternativ: F3, iTBS, 80%, 600 Pulse, 20-30 Sitzungen*) (Hebel et al., 2022)	Sollte-Empfehlung bei Therapie-Resistenz<br>Kann-Empfehlung nach nicht erfolgreicher Monotherapie	definitiv wirksam	FDA, CE	zahlreiche positive Meta-Analysen vorhanden

Ansprechrate: ca. 10-50%!
Es werden auch Stimulationsintensitäten von 80% berichtet!

Indikationen und Behandlungsprotokolle

Deutsche Gesellschaft für Hirnstimulation in der Psychiatrie e. V.

	Behandlung	Deutsche Leitlinien	Europä-ische Leitlinie	Zulass-ungen	Studienlage
**Negativ-symptomatik bei Schizophrenie**	Protokoll analog Depression (Lefaucheur et al., 2014; 2020)	Kann-Empfehlung bei Therapieresistenz im Rahmen eines Gesamt-behandlungsplans<br>„Die Patienten sollen über die hohe Rate an möglicher Non-Response aufgeklärt werden.“	möglich wirksam	nein	weitere Evidenz notwendig

Indikationen und Behandlungsprotokolle

Deutsche Gesellschaft für Hirnstimulation in der Psychiatrie e. V.

	Behandlung	Deutsche Leitlinien	Europä-ische Leitlinie	Zulass-ungen	Studienlage
**Akustische Halluzinationen bei Schizophrenie**	CP5, 1Hz, 80-100%, 1000/1200 Pulse, 10 Sitzungen<br>(oder CP5 **und** CP6, cTBS, 80%, je 600 Pulse, 15-20 Sitzungen)<br>(Lefaucheur et al., 2014; 2020; Plewnia et al., 2018; Ye et al., 2024)	Sollte-Empfehlung bei Therapieresistenz im Rahmen eines Gesamt-behandlungsplans	möglich wirksam	nein	weitere Evidenz notwendig

Indikationen und Behandlungsprotokolle

Deutsche Gesellschaft für Hirnstimulation in der Psychiatrie e. V.

	Behandlung	Deutsche Leitlinien	Europä-ische Leitlinie	Zulass-ungen	Studienlage
**Chronischer Tinnitus**	CP5, 1Hz, 110%, 2000 Pulse, 10 Sitzungen<br>(Folmer et al., 2015; Lefaucheur et al., 2014; 2020)	Sollte-Empfehlung **gegen** TMS<br>(Sondervotum der DGPPN: „kann erwogen werden“)	möglich wirksam	nein	weitere Evidenz notwendig

Indikationen und Behandlungsprotokolle

Deutsche Gesellschaft für Hirnstimulation in der Psychiatrie e. V.

	Behandlung	Deutsche Leitlinien	Europäische Leitlinie	Zulassungen	Studienlage
**Zwangsstörung**	SMA, 1Hz, 100% (Bein), mind. 1200 Pulse, 15-30 Sitzungen, ggf. mit gewinkelter 8er-Spule (oder F4, 1Hz, 110% RMT, mind. 1200 Pulse, 15-30 Sitzungen) (Fitzsimmons et al., 2022)	Kann-Empfehlung bei Therapieresistenz zur kurzfristigen Symptomlinderung	möglich wirksam	FDA (in Kombination mit Exposition), CE	Inkonsistenz der Protokolle, weitere Evidenz notwendig

Indikationen und Behandlungsprotokolle

Deutsche Gesellschaft für Hirnstimulation in der Psychiatrie e. V.

	Behandlung	Deutsche Leitlinien	Europä-ische Leitlinie	Zulass-ungen	Studienlage
**Abhängigkeit**	Behandlung analog Depression (Lefaucheur et al., 2014)	keine Empfehlung möglich	möglich wirksam bei Nikotin	FDA, CE für Sub-stanzen	weitere Evidenz nötig
**Posttraumatische Belastungs-störung**	F4, 1 vs. 20Hz, 80-120%, 100-4000 Pulse, 10-30 Sitzungen (Boggio et al., 2010)	TMS ist nicht erwähnt	wahr-scheinlich wirksam	nein	weitere Evidenz notwendig

Spulenpositionen - DLPFC (F3)

Deutsche Gesellschaft für Hirnstimulation in der Psychiatrie e. V.

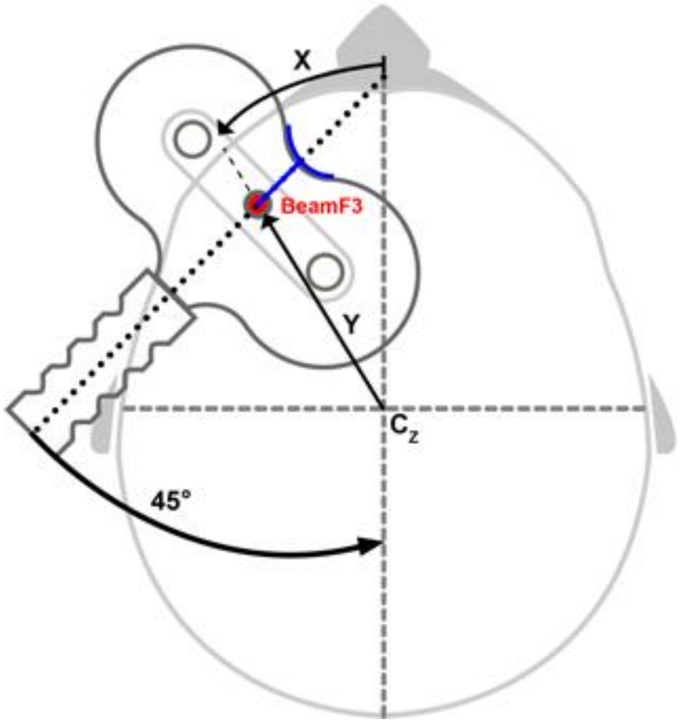

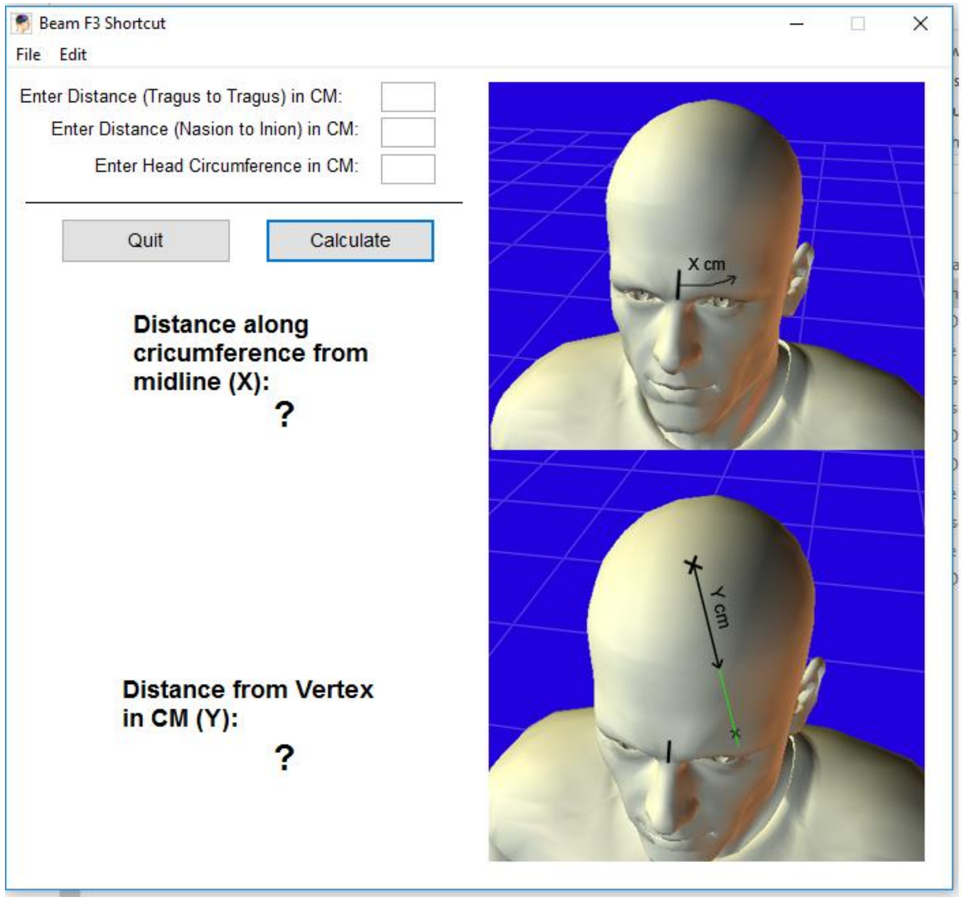

Beam et al. (2009) - doi: 10.1016/j.brs.2008.09.006

Spulenpositionen - TPJ (CP5)

Deutsche Gesellschaft für
Hirnstimulation
in der Psychiatrie e. V.

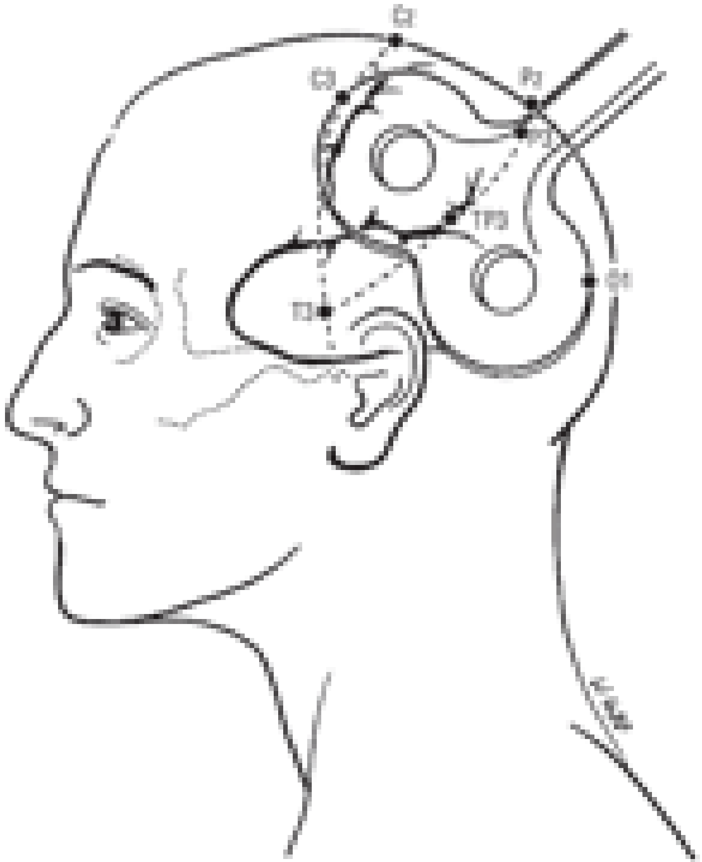

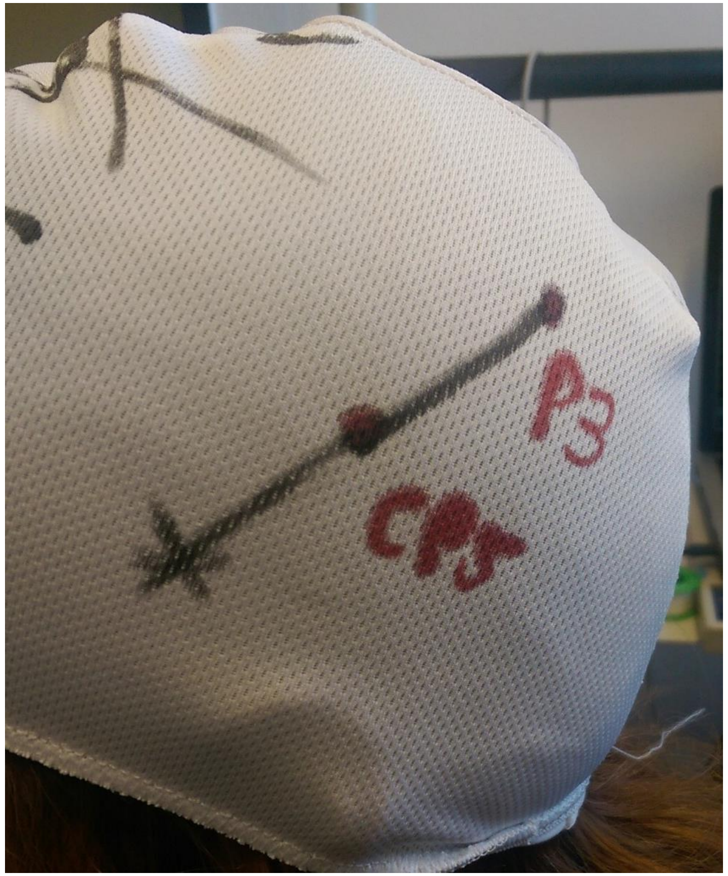

Hoffman et al. (2003) - doi: 10.1001/ARCHPSYC.60.1.49

Spulenpositionen - SMA (FFCz)

Deutsche Gesellschaft für Hirnstimulation in der Psychiatrie e. V.

- Ausgehend von Punkt Cz, 15% der Länge des Nasion-Inion-Abstandes auf der Mittellinie nach anterior (in Richtung Nase)
- Spulengriff zeigt nach dorsal

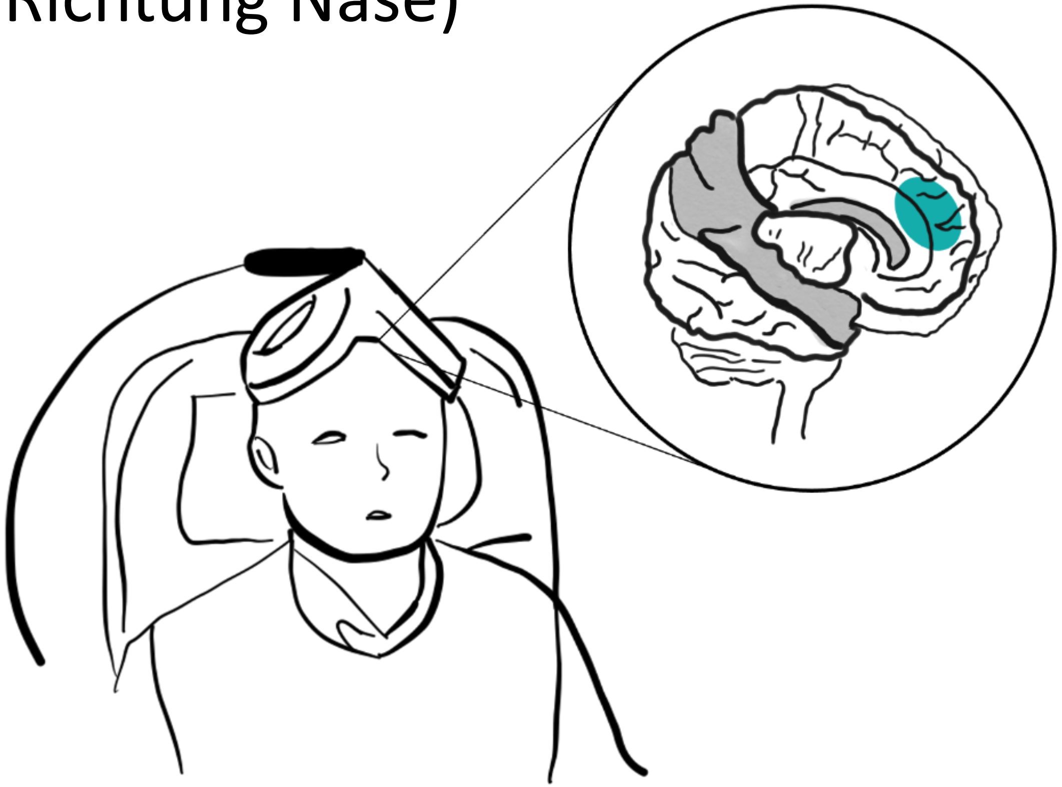

Akzelerierte Behandlung

Deutsche Gesellschaft für
Hirnstimulation
in der Psychiatrie e. V.

Akzelerierte Behandlung

Deutsche Gesellschaft für Hirnstimulation in der Psychiatrie e. V.

- schnelleres Ansprechen
- iTBS statt 10Hz (intermittierende Theta-Burst-Stimulation)
- mehrere Sitzungen am Tag (15-50min Pause)
- Abweichungen vom Standard begründen

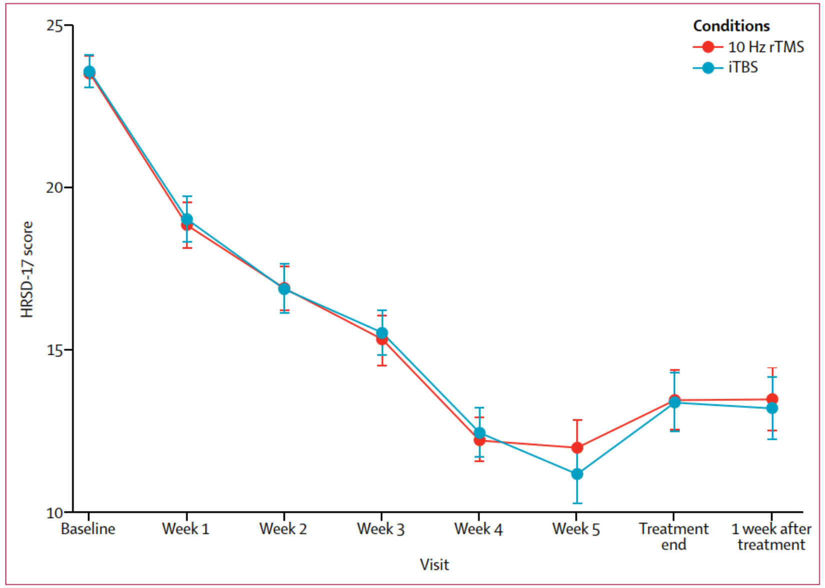

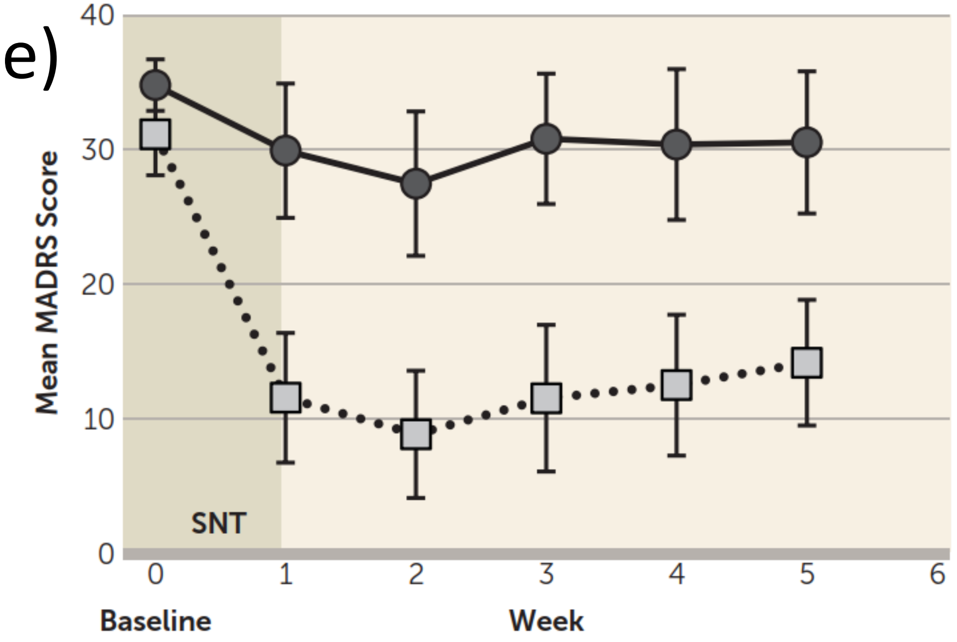

Blumberger et al. (2018) - doi: 10.1016/S0140-6736(18)30295-2
Cole et al. (2021) - doi: 10.1176/appi.ajp.2021.20101429

Akzelerierte Behandlung

Deutsche Gesellschaft für
Hirnstimulation
in der Psychiatrie e. V.

- schnelleres Ansprechen
- iTBS statt 10Hz (intermittierende Theta-Burst-Stimulation)
- mehrere Sitzungen am Tag (15-50min Pause)
- Abweichungen vom Standard begründen

- offene Fragen:
    - je mehr, desto besser? (Pulszahl, Sitzungszahl)
    - Neuronavigation nötig?
    - wenig placebo-kontrollierte positive Studien

Aufrechterhaltung und Rückfallprophylaxe

Deutsche Gesellschaft für
Hirnstimulation
in der Psychiatrie e. V.

- Verwendung nach erfolgreicher TMS (nach Remission/Response)
- etliche Daten zu effectiveness (naturalistisches Setting)
- wenige Daten zur efficacy (kontrollierte Studien)
- zwei Schemata möglich
  - Ausschleichen der Sitzungen
  - Booster-Sitzungen
  - Beispiel LMU

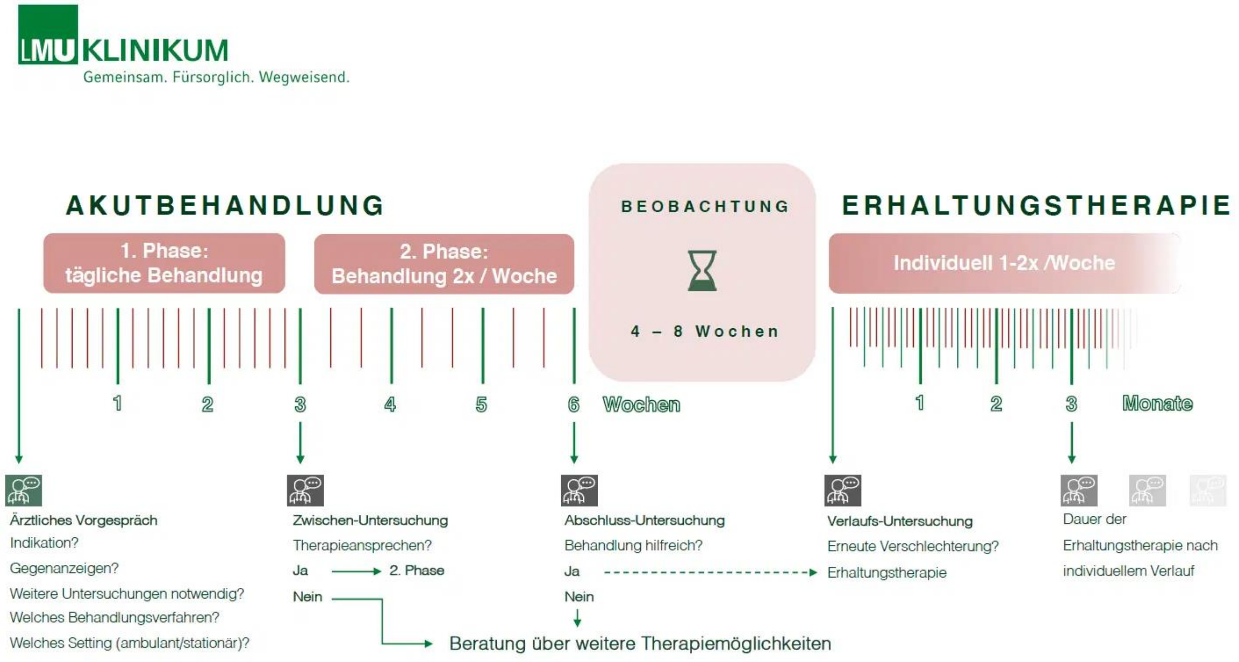

Supplement: Supplementary file 1 [file SupplementaryFile1.zip › Workshop (German).PDF]
