# Supplementary material for: rTMS for the treatment of psychiatric disorders: a review about training courses and materials and the presentation of the training materials of the German Society for Brain Stimulation in Psychiatry
Source: Front Psychiatry. 2025 Aug 8;16:1490039. doi: 10.3389/fpsyt.2025.1490039 (PMC12371536; doi:10.3389/fpsyt.2025.1490039)

Transcranial Magnetic Stimulation

German Society for Brain Stimulation in Psychiatry (DGHP; registered society)

Hands-On Workshop April 2025

Deutsche Gesellschaft für
Hirnstimulation
in der Psychiatrie e. V.

Transcranial Magnetic Stimulation

German Society for Brain Stimulation in Psychiatry (DGHP; registered society)

Beginner-Workshop April 2025

Deutsche Gesellschaft für
Hirnstimulation
in der Psychiatrie e. V.

Overview (3h theory & 3h hands-on)

Deutsche Gesellschaft für Hirnstimulation in der Psychiatrie e. V.

What?	Where?	How Long?
Theory: Basics, coil positioning, motor threshold, treatment of depression		2 Hours
Hands-On		1 Hour
Break		1 Hour
Hands-On		1 Hour
Hands-On		1 Hour
Theory: pre-treatment consultation, contraindications and side effects, billing		1 Hour
Conclusion		

Basics

Deutsche Gesellschaft für
Hirnstimulation
in der Psychiatrie e. V.

Basics: physics and mechanisms

Deutsche Gesellschaft für
Hirnstimulation
in der Psychiatrie e. V.

- Basic principle: electromagnetic induction

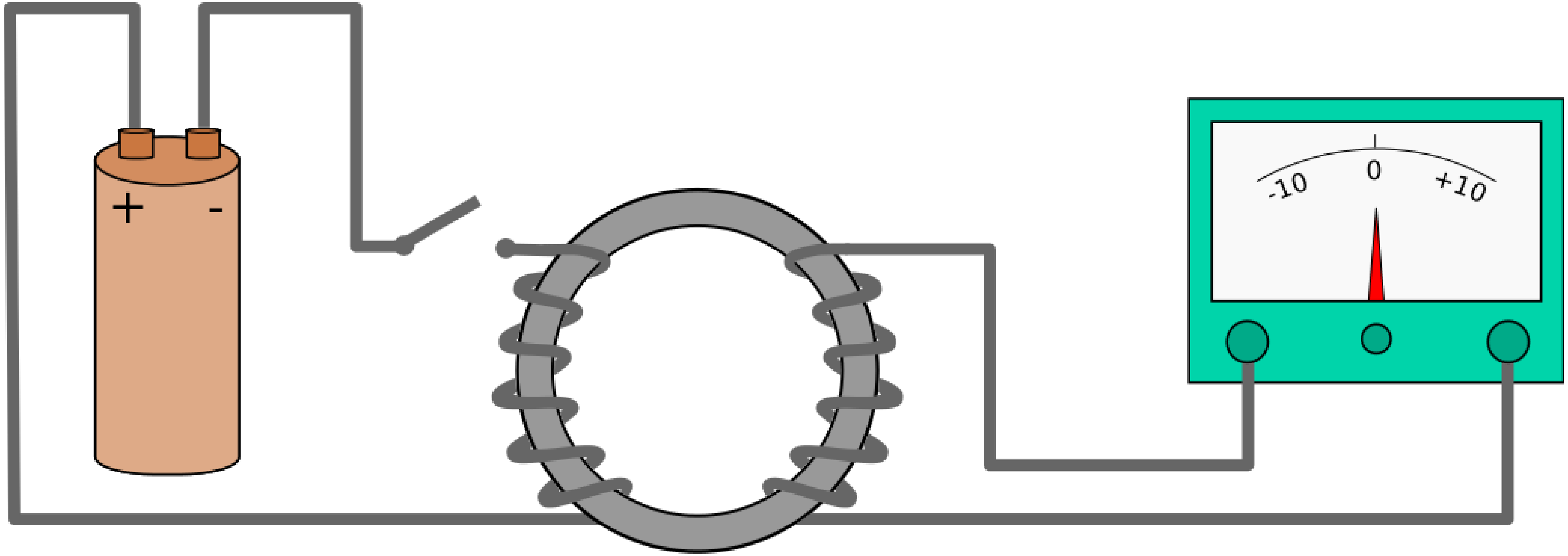

https://en.wikipedia.org/wiki/Electromagnetic_induction

Basics: physics and mechanisms

- Basic principle: electromagnetic induction
- Neurons as electrical conductors

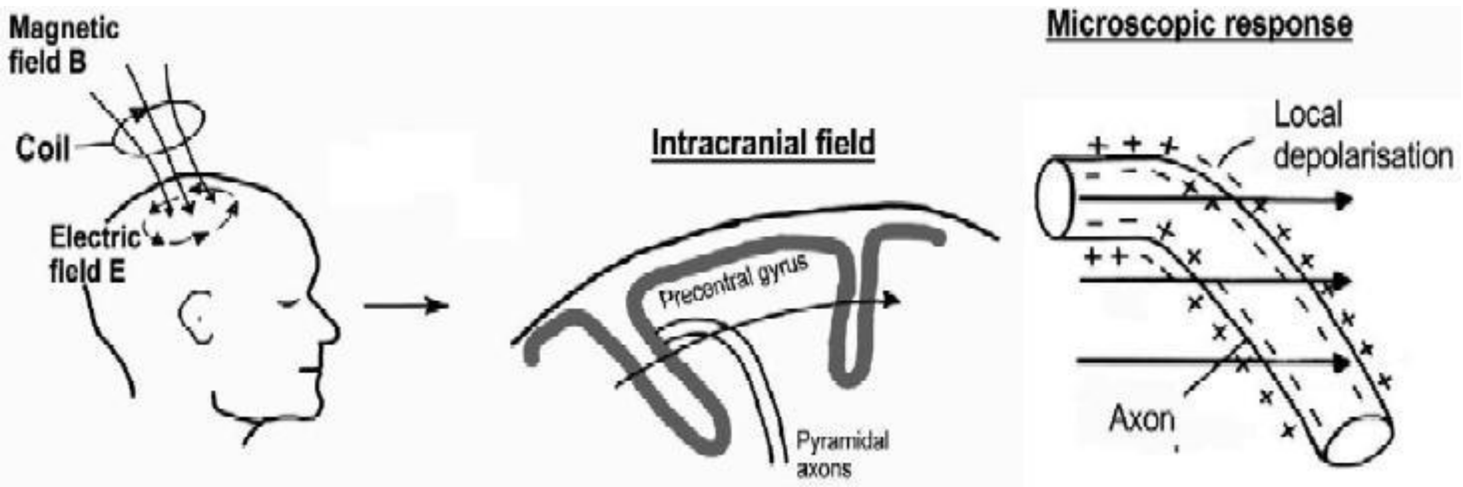

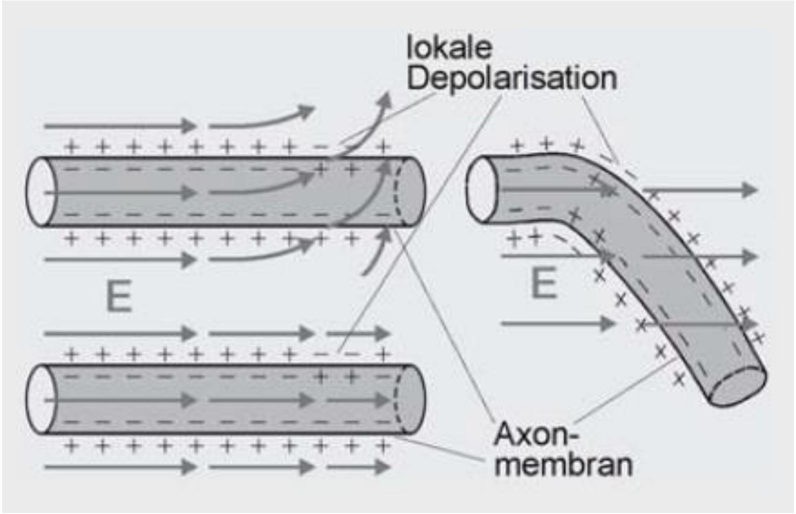

Siebner & Ziemann (2007) - doi: 10.1007/978-3-540-71905-2
Viesca et al. (2012) - doi: 10.1063/1.4764608

Basics: physics and mechanisms

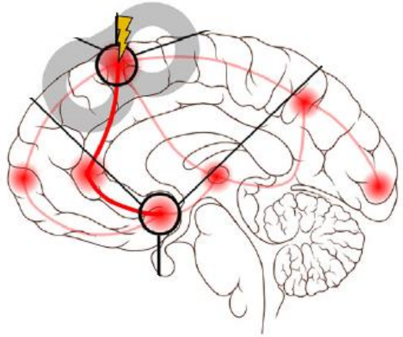

Deutsche Gesellschaft für Hirnstimulation in der Psychiatrie e. V.

- Basic principle: electromagnetic induction
- Neurons as electrical conductors
- Induced electric fields: close to the skull

Remote effects are likely!

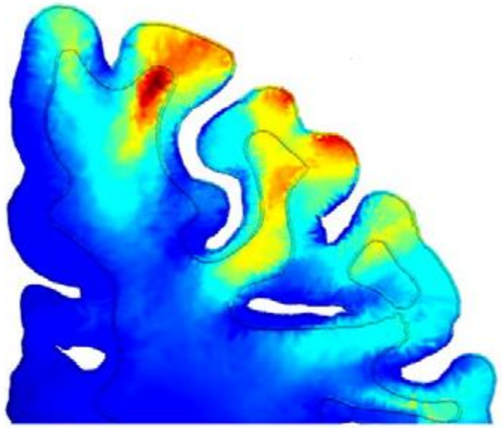

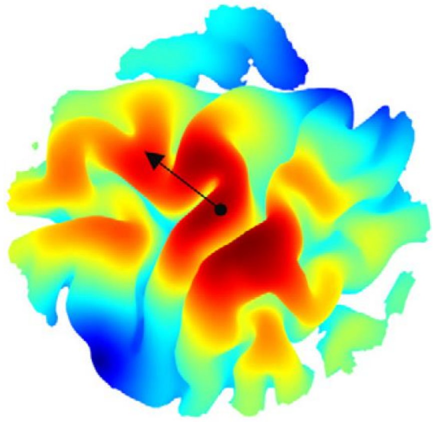

Earth‘s magnetic field: 40µT
Horseshoe magnet: 4mT
TMS/MRT: 1-3T

Optiz et al. (2011) - doi: 10.1016/j.neuroimage.2011.06.069
Mueller-Dahlhaus & Bergmann (2023) - doi: 10.1016/j.xcrm.2023.101086

Basics: physics and mechanisms

- Basis principle: electromagnetic induction
- Neurons as electrical conductors
- Induced electric fields: close to the skull
- Coil geometry: winding, angles, diameter etc. are relevant!

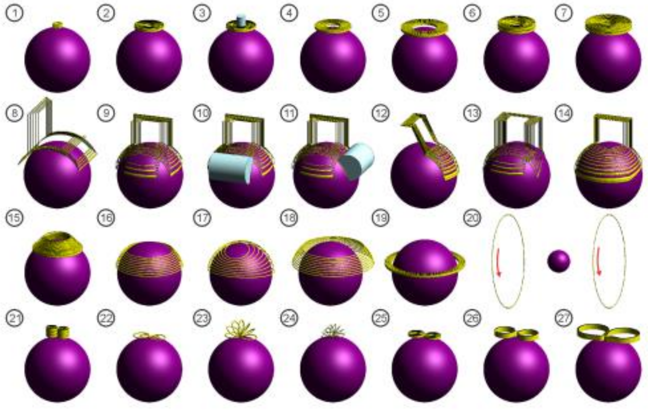

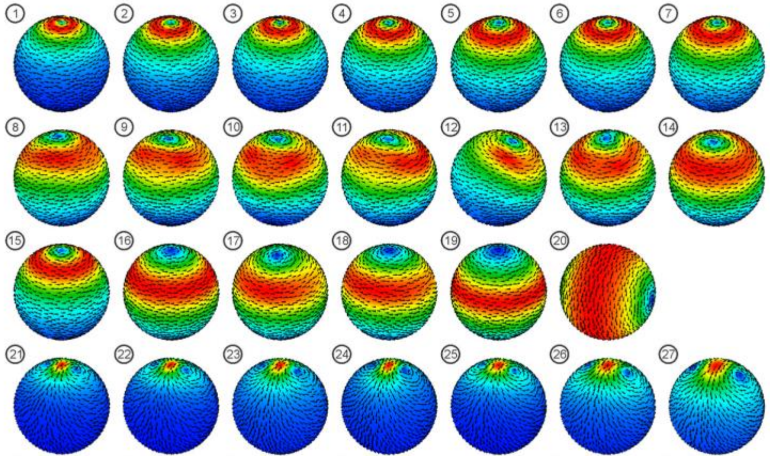

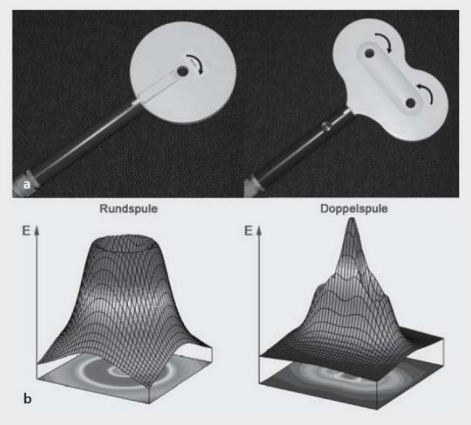

There are hardly any studies available with circular coils!

Siebner & Ziemann (2007) - doi: 10.1007/978-3-540-71905-2
Deng et al. (2013) - doi:10.1016/j.brs.2012.02.005

Basics: physics and mechanisms

Deutsche Gesellschaft für Hirnstimulation in der Psychiatrie e. V.

- Basic principle: electromagnetic induction
- Neurons as electrical conductors
- Induced electric fields: close to the skull
- Coil geometry
- Pulse direction/coil orientation ('Which way is the handle pointing?'): device manufacturers have different default settings

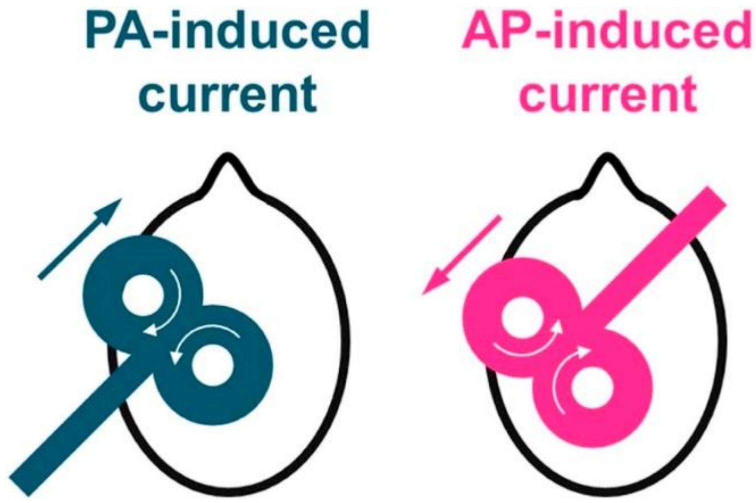

Hannah & Rothwell (2017) - doi: 10.1016/j.brs.2016.09.008
Schoisswohl et al. (2022) - doi: 10.1016/j.neulet.2022.137026

Basics: stimulation parameters

Deutsche Gesellschaft für
Hirnstimulation
in der Psychiatrie e. V.

- Intensity = stimulation strength
- Units of measurement:
  - Stimulator output (specified as a percentage of the device's power, i.e. %MSO)
    - → CAUTION: not every coil and stimulator have the same power!
  - Motor threshold (specified as %MSO)
  - Stimulation strength based on motor threshold (e.g., 110% motor threshold means: at a motor threshold of 50% stimulator output, we need 55% stimulator output)
    - → CAUTION: there is also evidence of the effectiveness of subthreshold stimulation!

Basics: stimulation parameters

Deutsche Gesellschaft für Hirnstimulation in der Psychiatrie e. V.

- Intensity = stimulation strength
- Frequency = speed or amount of pulses in a time period
    - ≤1Hz is considered inhibitory
    - >5Hz: is considered excitatory
    - classification in a psychiatric-therapeutic context

Deutsche Gesellschaft für
Hirnstimulation
in der Psychiatrie e. V.

Basics: stimulation parameters

- Intensity = stimulation strength
- Frequency = speed or amount of pulses in a time period
- Train = amount of pulses in a block
- Inter-train interval: break period between trains

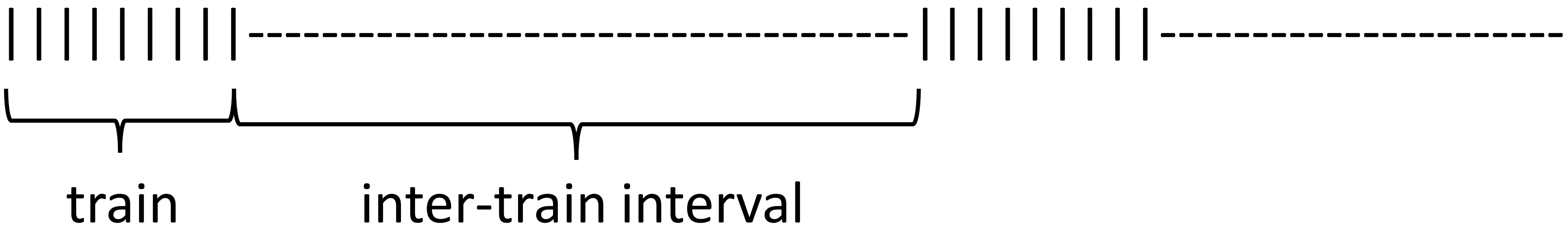

Basics: stimulation parameters

- Theta burst stimulation (TBS): pulse triplets (50Hz, every 20ms) in a 5Hz rhythm (every 200ms)
  - continuous TBS (cTBS): considered inhibitory
  - intermittent TBS (iTBS): considered excitatory

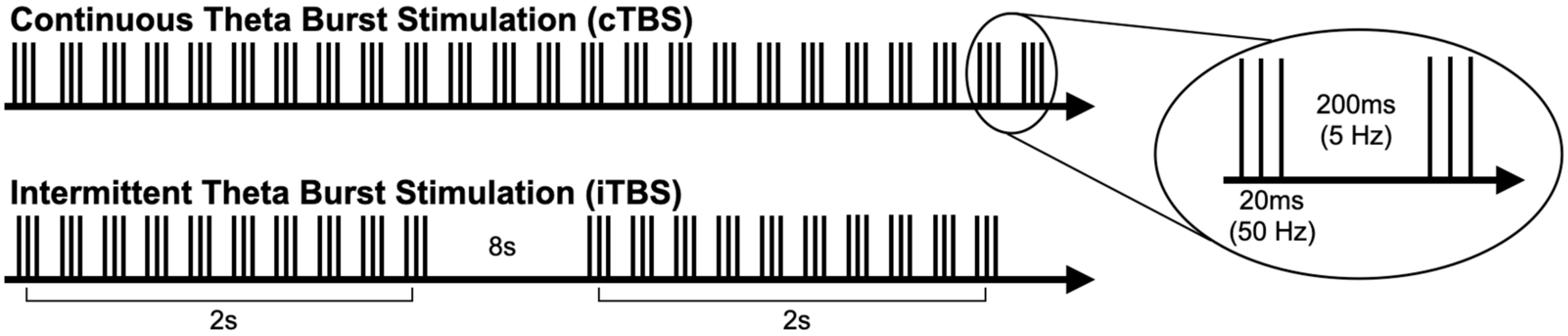

Huang et al. (2005) - doi: 10.1016/j.neuron.2004.12.033

Basics: neuroscientific principles

- Cortical excitability (e.g., motor threshold) and neuroplasticity (long-term potentiation and long-term depression)

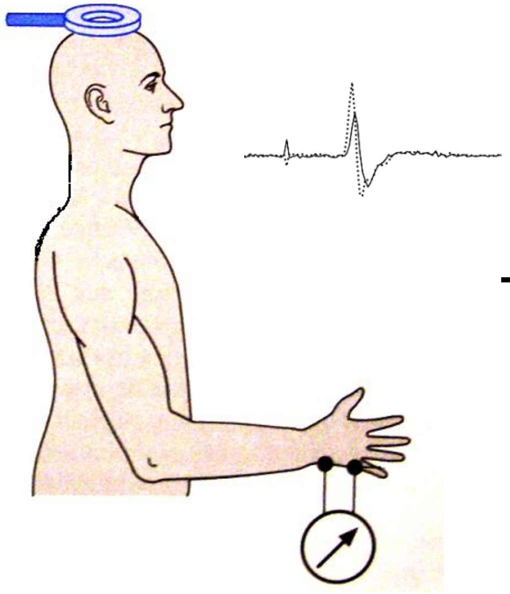

single pulses =
diagnostics

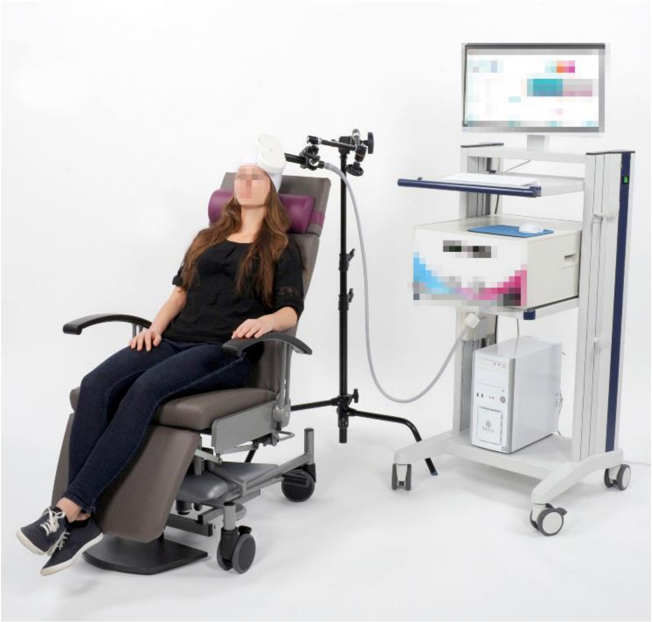

series of pulses =
repetitive TMS (rTMS) =
treatment

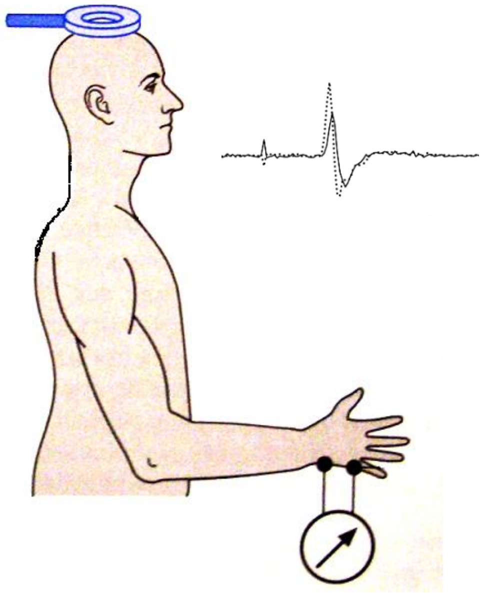

MEP change =
neuromodulatory aftereffect

Basics: neuroscientific principles

- Cortical excitability (e.g., motor threshold) and neuroplasticity (long-term potentiation and long-term depression)
- Changes measurable with neuroscientific methods such as EEG and MRI, but not suitable as biomarkers

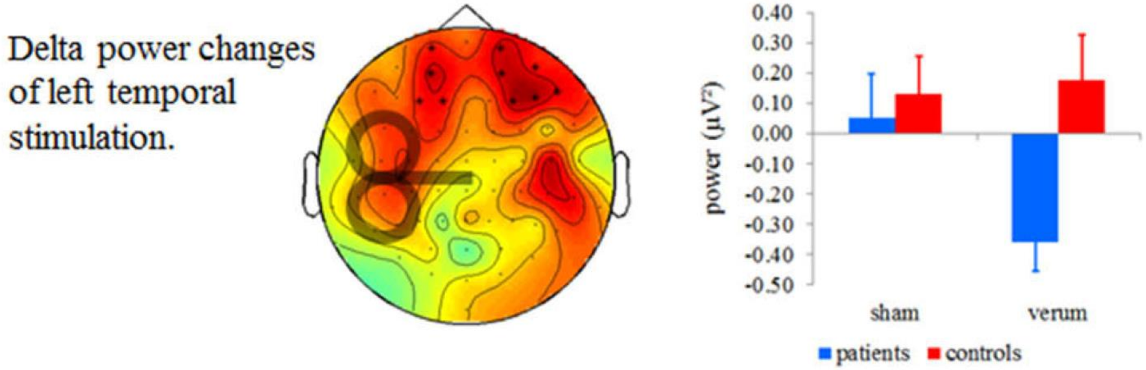

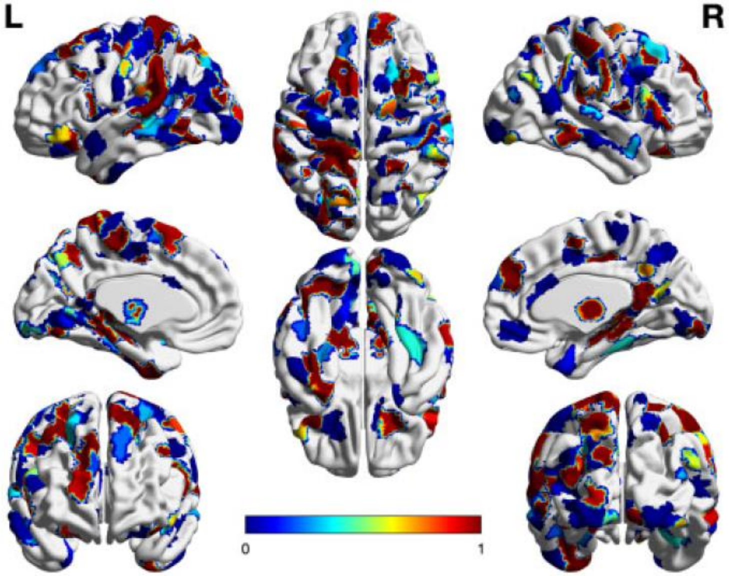

Schecklmann et al. (2015) - doi: 10.3389/fncel.2015.00421
Poeppl et al. (2021) - doi: 10.1093/braincomms/fcab115

Deutsche Gesellschaft für Hirnstimulation in der Psychiatrie e. V.

Basics: neuroscientific principles

Deutsche Gesellschaft für
Hirnstimulation
in der Psychiatrie e. V.

- Cortical excitability (e.g., motor threshold) and neuroplasticity (long-term potentiation and long-term depression)
- Changes measurable with neuroscientific methods such as EEG and MRI, but not suitable as biomarkers
- Possible mechanism of action
  - Depression: dysbalance/hypofrontality model

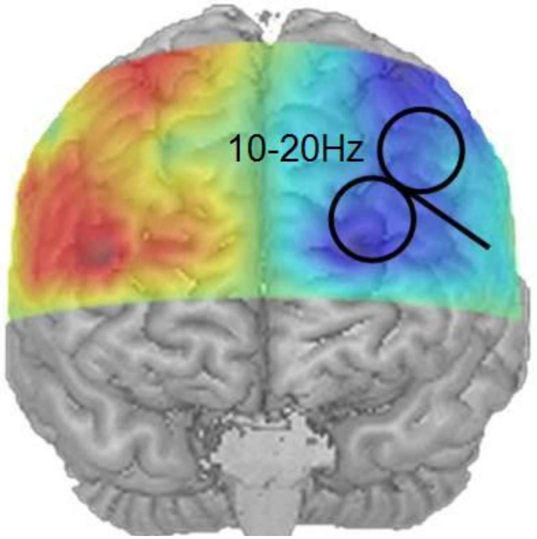

van der Finne et al. (2017) - doi: 10.1016/j.nicl.2017.07.006

Basics: neuroscientific principles

Deutsche Gesellschaft für
Hirnstimulation
in der Psychiatrie e. V.

- Cortical excitability (e.g., motor threshold) and neuroplasticity (long-term potentiation and long-term depression)
- Changes measurable with neuroscientific methods such as EEG and MRI, but not suitable as biomarkers
- Possible mechanism of action
  - Depression: dysbalance/hypofrontality model
  - Phantom perceptions: overactivity of the auditory cortex

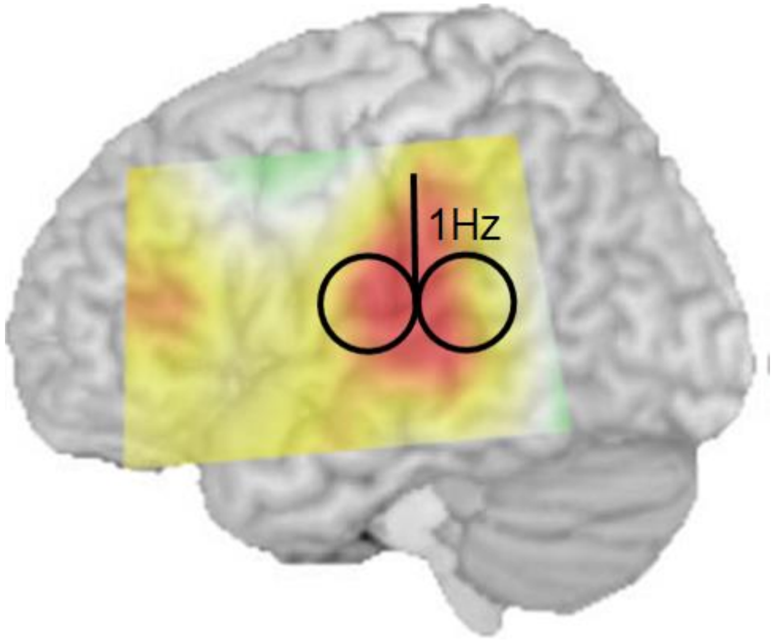

De Ridder et al. (2007) - doi: 10.1007/978-3-211-33081-4_52

Excursus: treatment process

Deutsche Gesellschaft für Hirnstimulation in der Psychiatrie e. V.

- Pre-treatment consultation with a **medical physician**
- No EEG or MRI required
- Motor threshold (to determine treatment intensity)
- Treatment (can be delegated by a physician and carried out by alternative practitioners)
- Interim evaluations and final assessment
- where appropriate: continued treatment
- where appropriate: follow-up examination
- where appropriate: relapse prevention

Coil positioning

Deutsche Gesellschaft für
Hirnstimulation
in der Psychiatrie e. V.

- Patient in a relaxed position during treatment
- Motor threshold is determined while seated
- Use of a figure-eight coil
- CAUTION: depending on the practitioner’s position relative to the patient (standing to the side or behind) and the degree of the patient reclination, coil position will vary

  → the treatment unit should standardize how patient and practitioner are positioned (e.g. SOP document, training)

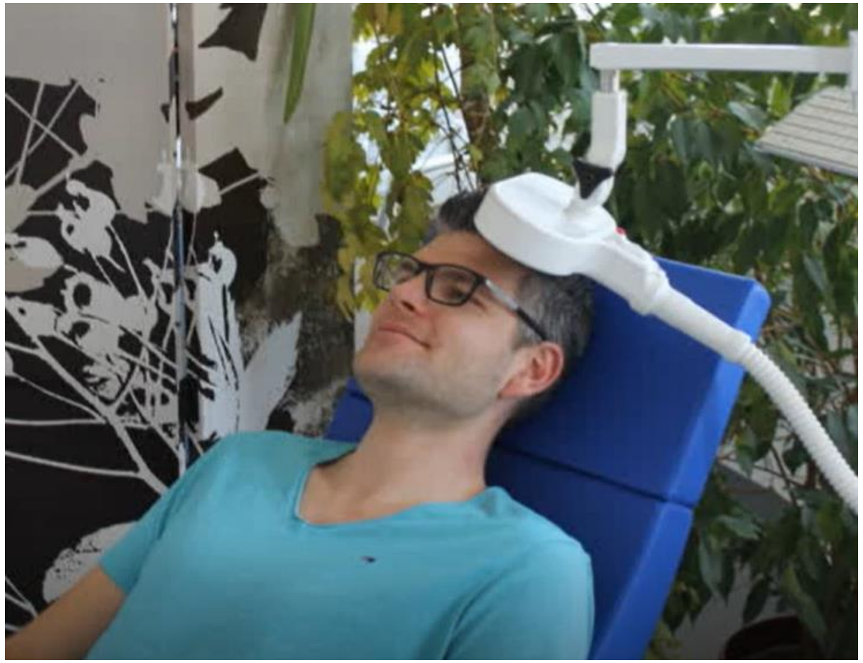

Coil positioning

Deutsche Gesellschaft für
Hirnstimulation
in der Psychiatrie e. V.

- The coil should lie tangentially on the head
- The coil center should rest against the head
- The coil orientation is at a 90-degree angle to the gyrus
- The coil position should be monitored during the procedure
- The coil handle should point towards the back of the head
- The coil should be placed with **minimal** pressure (risk of injury)

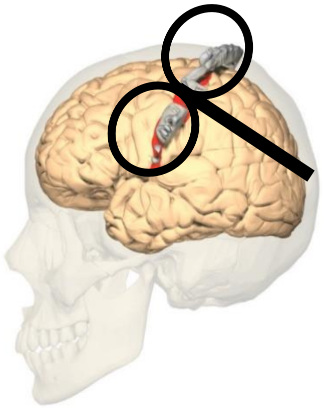

2021, StatPearls Publishing LLC.

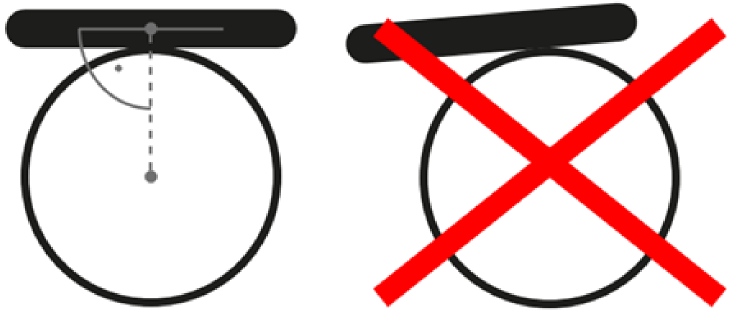

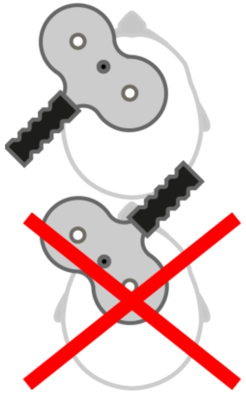

Coil positioning

Deutsche Gesellschaft für
Hirnstimulation
in der Psychiatrie e. V.

Surface-based treatment spot identification

- Uses EEG system positions

  (e.g. 10-20 EEG system)

- Requires equipment:
  - Patient cap
  - Triangle ruler
  - Measuring tape
  - Marker pen
  - For „lazy ones“: EEG caps…

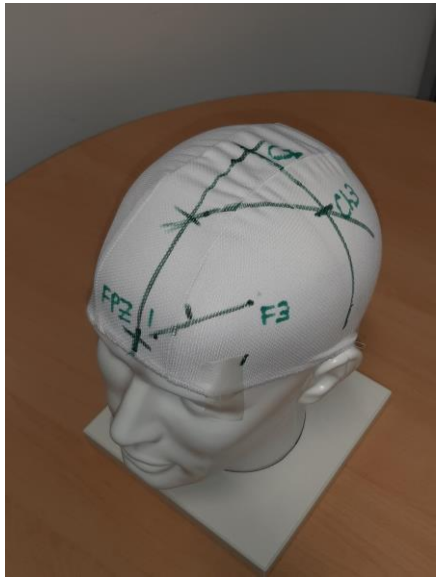

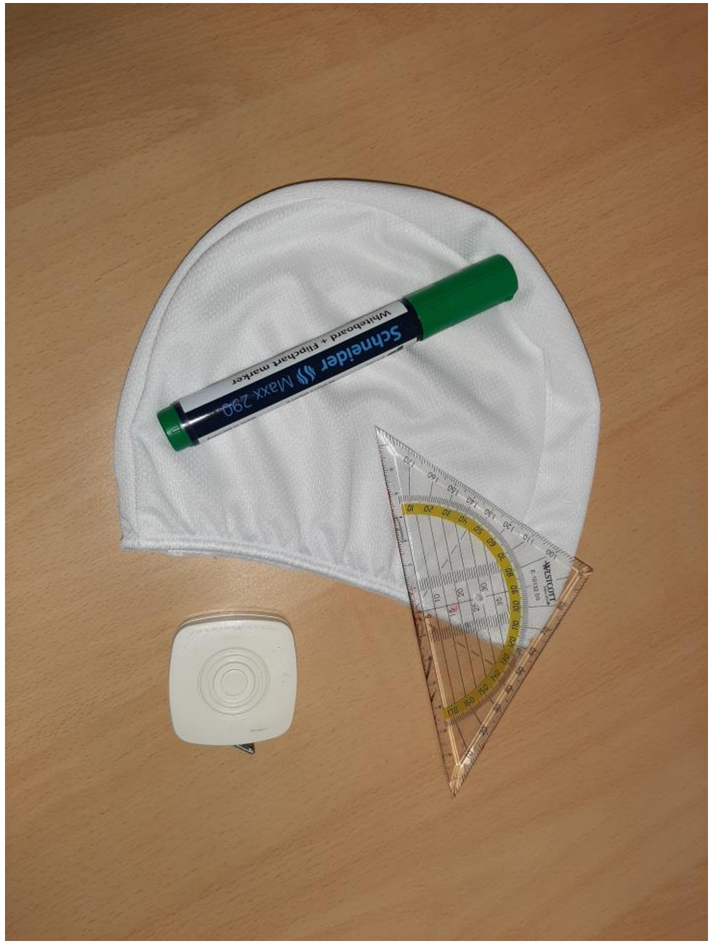

Excursus: 10-20 EEG system

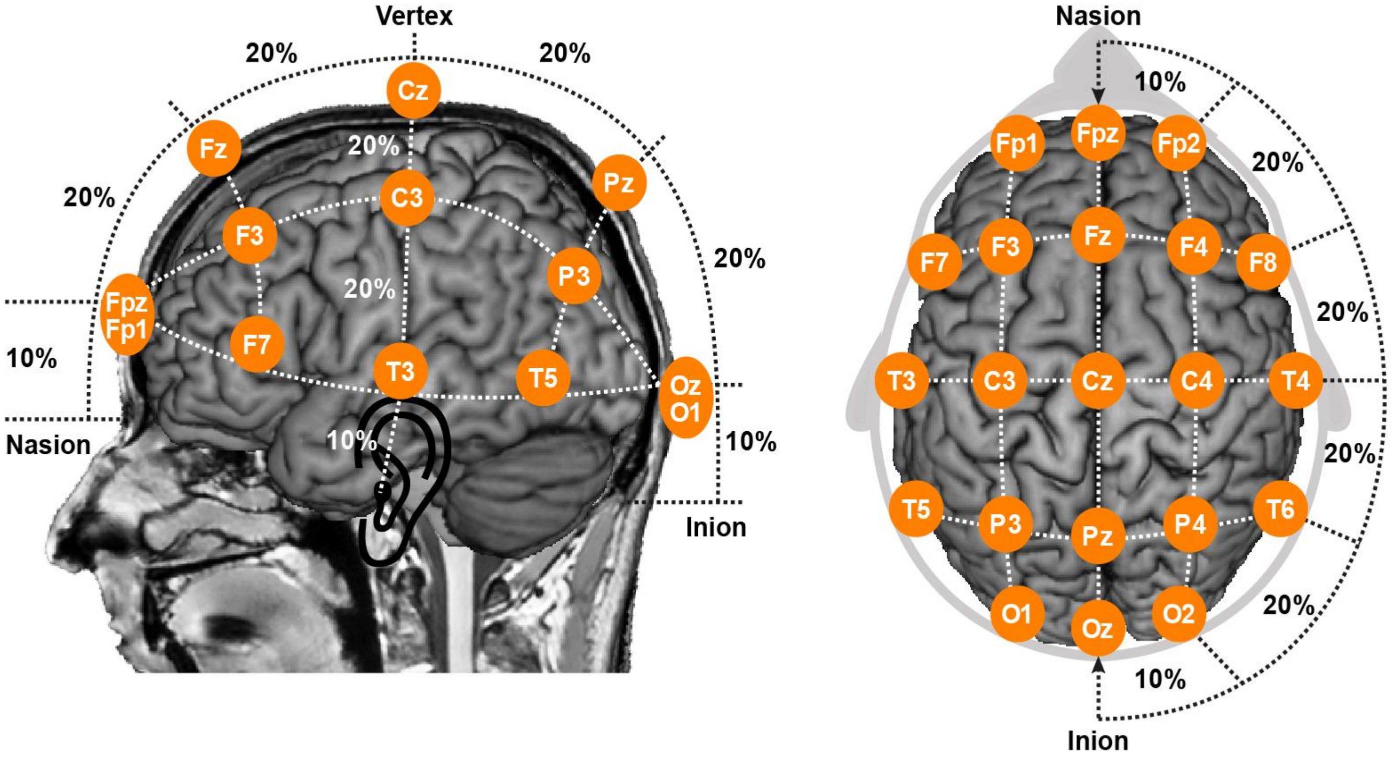

Deutsche Gesellschaft für Hirnstimulation in der Psychiatrie e. V.

Coil positioning

Deutsche Gesellschaft für
Hirnstimulation
in der Psychiatrie e. V.

Surface-based

- Uses EEG system positions
- Handy
- Evidence-based
- Necessary

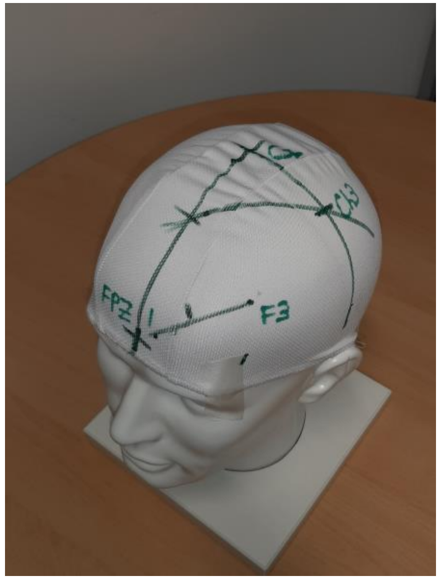

Neuronavigation

- Based on anatomical or functional brain scans
- More precise and reliable, but also more laborious
- Too few superiority trials available
- Possible

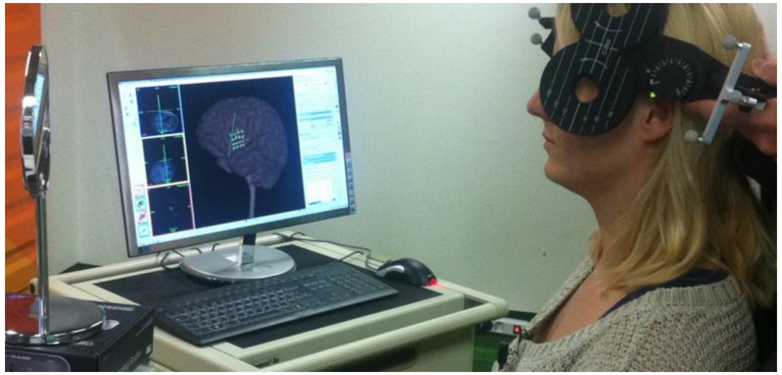

Motor threshold

Deutsche Gesellschaft für
Hirnstimulation
in der Psychiatrie e. V.

Motor threshold

Deutsche Gesellschaft für Hirnstimulation in der Psychiatrie e. V.

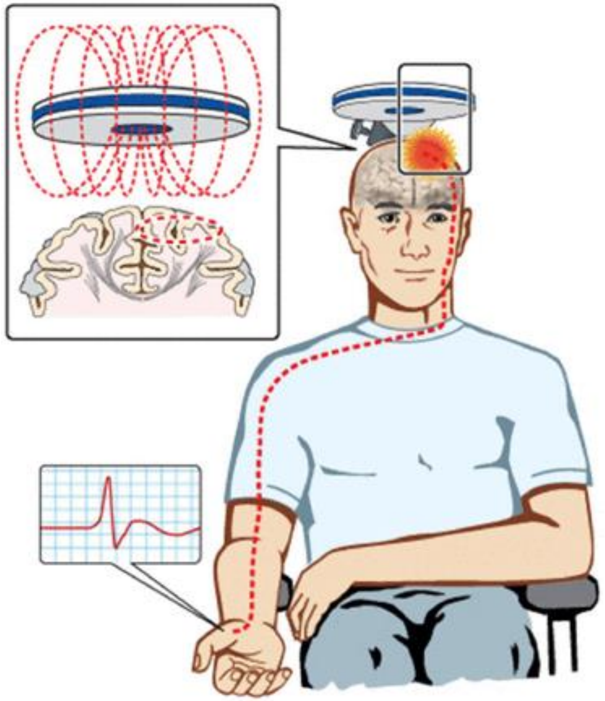

- 2-step procedure: hotspot localization and determination of threshold intensity
- Standard: determining the **resting motor threshold** (RMT) of relaxed finger muscles

  (abductor muscle of the pinky, ball of the thumb, or flexor of the index finger)
- Standard: stimulation of the motor cortex on the treatment side

  e.g. depression treatment: left motor cortex (finger muscles of the right hand)

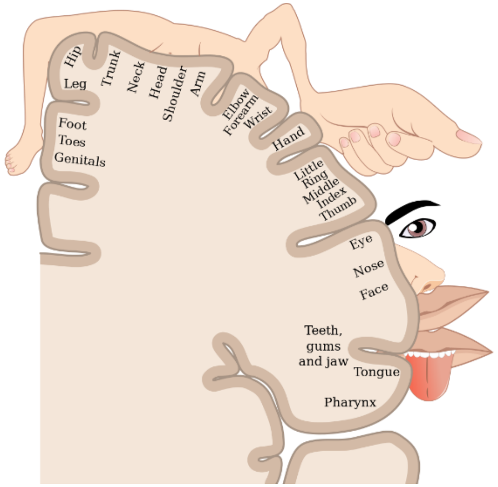

https://commons.wikimedia.org/wiki/File:Sensory_Homunculus-en.svg
Vucic et al. (2012) - doi: 10.1136/jnnp-2012-304019

Motor threshold

Deutsche Gesellschaft für
Hirnstimulation
in der Psychiatrie e. V.

- <u>Recommended</u>: derivation of motor-evoked potentials (MEPs) using electromyography
  - *(note: if no EMG is available, visual twitches can also be used, but* may only occur at approximately 10% higher stimulator output compared to MEP detection*)*
- MEP: biphasic wave 20-40 ms after pulse
- Electrodes: belly-tendon montage
  - <u>active</u>: muscle belly
  - <u>passive</u>: tendon insertion
  - <u>ground</u>: between electrodes and head (e.g. ulnar styloid process)

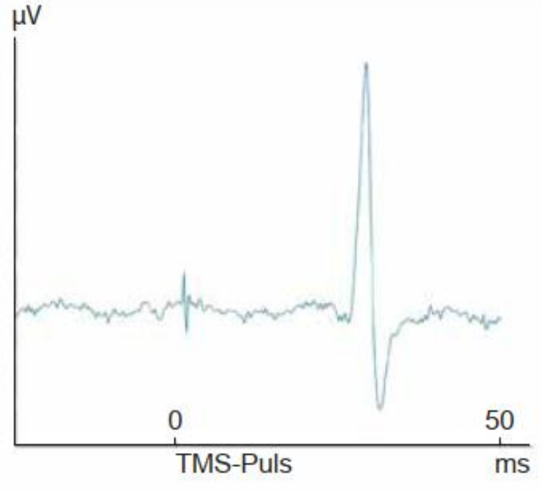

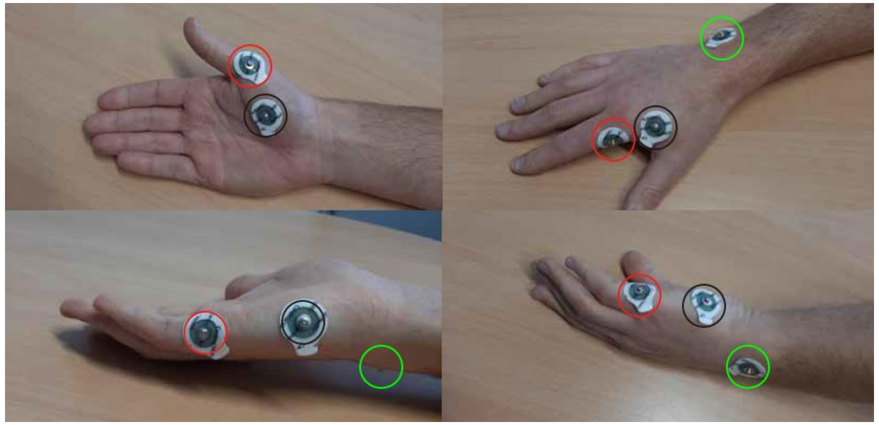

Deutsche Gesellschaft für Hirnstimulation in der Psychiatrie e. V.

Motor threshold

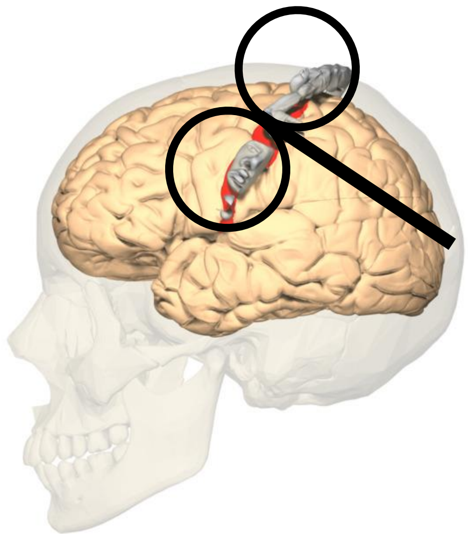

- Starting point for hot-spot search: C3/C1
- Functional determination based on muscle response
- Coil orientation at a 45-degree angle to the midline
- How to mark the 45° angle:
  - From Cz, measure 5 cm laterally (towards the ear) and mark this point
  - From Cz again, measure 5 cm anteriorly (towards the nose) and mark this point
  - Connect the two marks – the resulting line forms a 45° angle relative to the midline

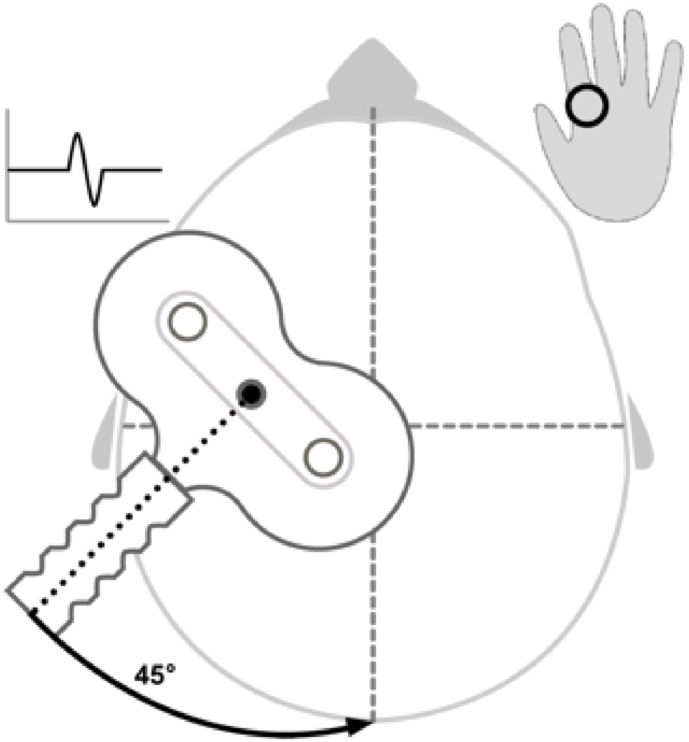

https://www.statpearls.com/ArticleLibrary/viewarticle/29224

Motor threshold

- Determined once before treatment
- Monitoring during intake of relevant psychotropic medication (e.g., benzodiazepines)
- Hotspot localization with stimulation intensities above threshold
- Motor thresholding algorithms:
  - pragmatically: MEP detected in 4 out of 8 stimulations (50µV or visual twitch)
  - via „threshold hunting“ (computer-based algorithm)

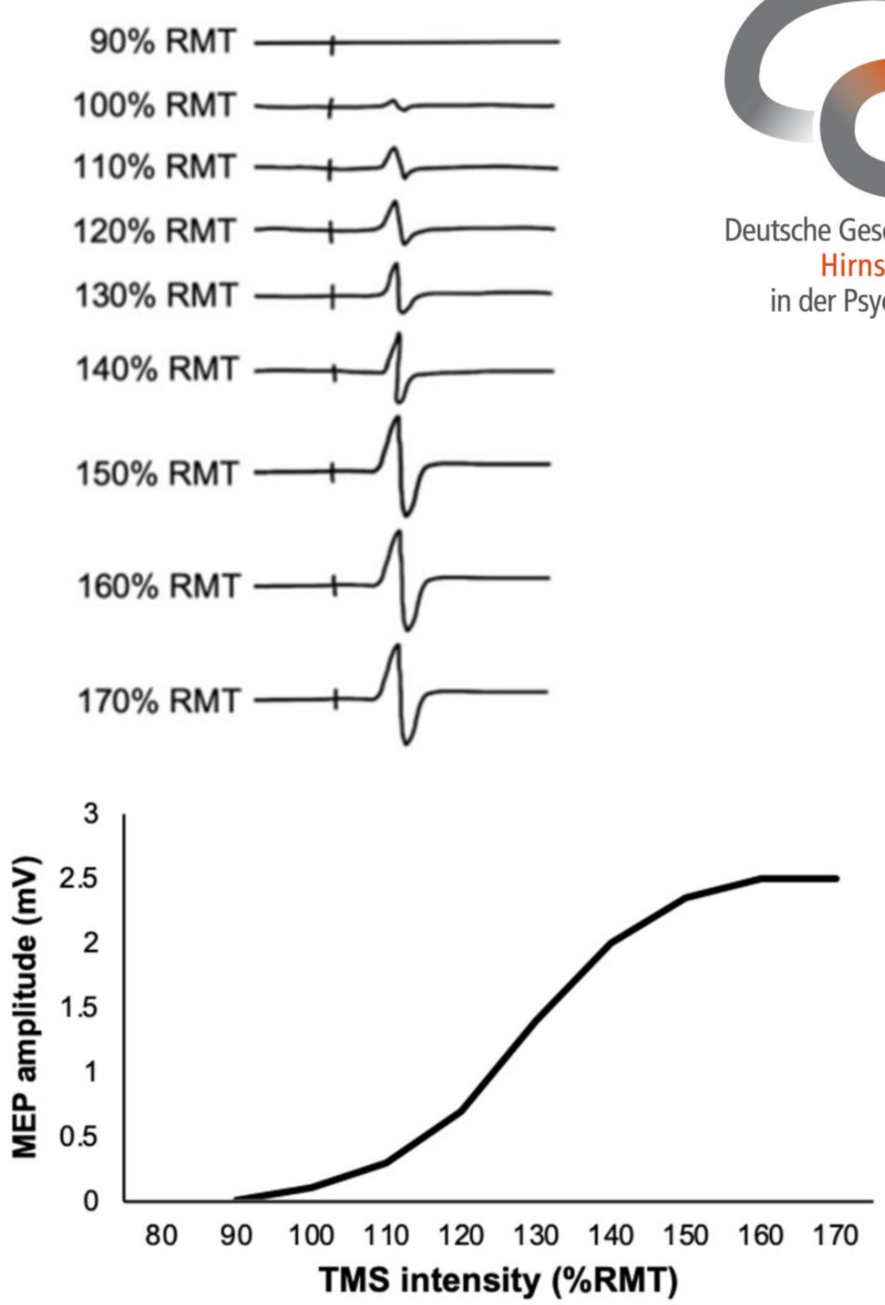

Deutsche Gesellschaft für Hirnstimulation in der Psychiatrie e. V.

Turco & Nelson (2021) - doi: 10.3389/fnrgo.2021.679033

Motor threshold

Deutsche Gesellschaft für
Hirnstimulation
in der Psychiatrie e. V.

- **Active motor threshold** (AMT):
  - Eventual alternative in patients with heightened muscle tone (e.g. tremor, spasticity, dystonia, or post-stroke or dementia-related phenomena)
  - Frequently used for theta-burst stimulation protocols
  - Typically, AMT is lower than RMT (in %MSO)
  - Procedure:
    - basically as in RMT assessment, however with preactivated target (finger) muscles
    - application of motor threshold algorithm (threshold of 200 µV or visual twitch)
  - CAUTION: problems with standardization of the procedure (e.g. without force grip sensor)

Sen et al. (2017) – doi: 10.1371/journal.pone.0186007
Turi et al. (2021) – doi: 10.1111/ejn.15195

Motor threshold

Deutsche Gesellschaft für
Hirnstimulation
in der Psychiatrie e. V.

- Additional considerations and caveats:
  - avoid high levels of pressure to the head during coil placement and movement (e.g. during hotspot search)
  - advisable: team member with proficiency in EMG measurements
    - standardized skin preparation procedures for EMG electrode placement
    - baseline check of EMG signal quality
    - problems of reliable EMG recordings in certain conditions (e.g. ulnar nerve compression neuropathy, peripheral neuropathy, radiculopathy etc.)

Treatment of depression

Deutsche Gesellschaft für
Hirnstimulation
in der Psychiatrie e. V.

Treatment of depression

- DLPFC: BEAM-F3 (formerly based on the 5/6cm rule)
- The „adjusted“ BEAM-F3 is less studied

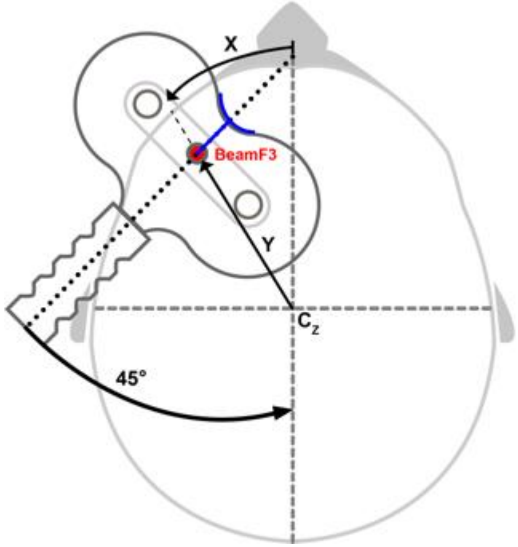

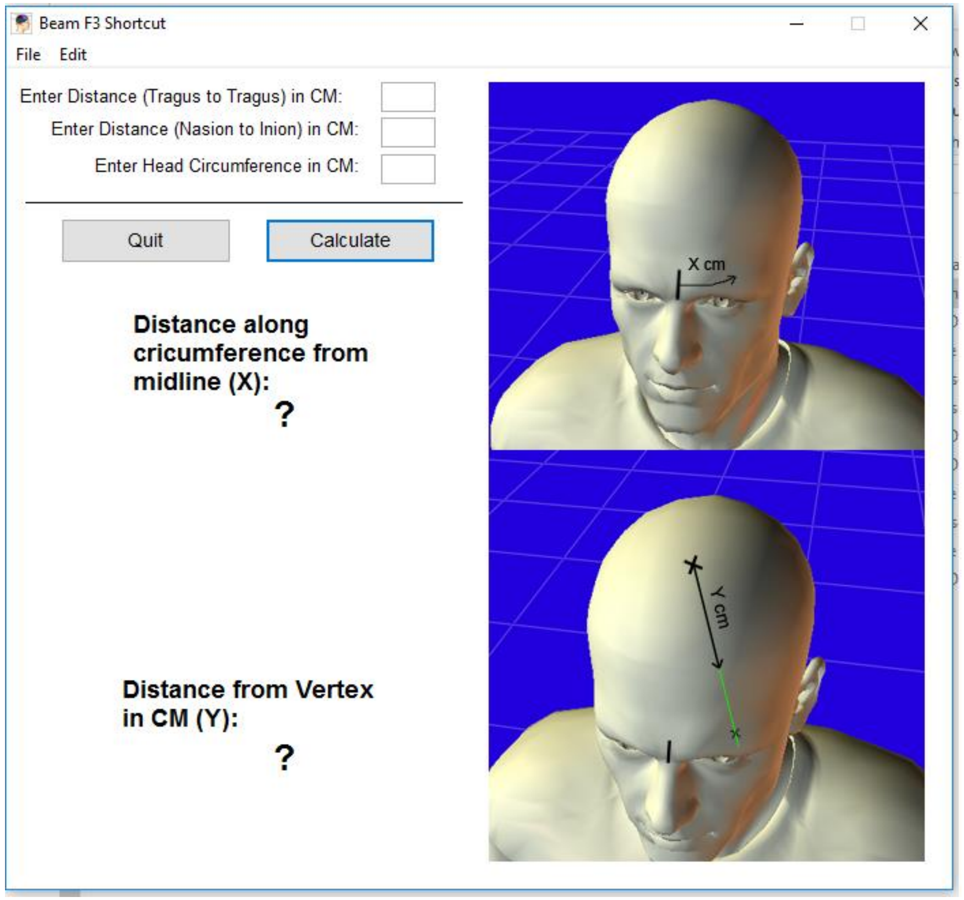

Beam et al. (2009) - doi: 10.1016/j.brs.2008.09.006

Treatment of depression

Deutsche Gesellschaft für Hirnstimulation in der Psychiatrie e. V.

	treatment	German guidelines	European guidelines	approvals	state of research
**Unipolar/ bipolar depression**	F3, 10/20Hz, 100-120%, 1500-3000 pulses, 20-30 sessions (*alternatively: F3, iTBS, 80%, 600 pulses, 20-30 sessions*) (Hebel et al., 2022)	"*should be offered*" recommendation in cases of therapy resistance "*can be offered*" recommendation after unsuccessful monotherapy	definitely effective	FDA, CE	numerous positive meta-analyses available

Response rate: approximately 10-50%!
80% stimulation intensities have also been reported!

German treatment guidelines

Deutsche Gesellschaft für Hirnstimulation in der Psychiatrie e. V.

Recommendation	Level of Recommendation
**7-16 | new 2022**<br>For patients who do not respond to monotherapy with antidepressants, augmentation with repetitive transcranial magnetic stimulation (rTMS) can be offered.	⇔
**7-29 | modified 2022**<br>Repetitive transcranial magnetic stimulation (rTMS) should be offered for treatment-resistant depressive episodes.	⇑
**7-30 | new 2022**<br>The selection of a rTMS method (stimulation site and type) should be done by a specialized center	⇑⇑

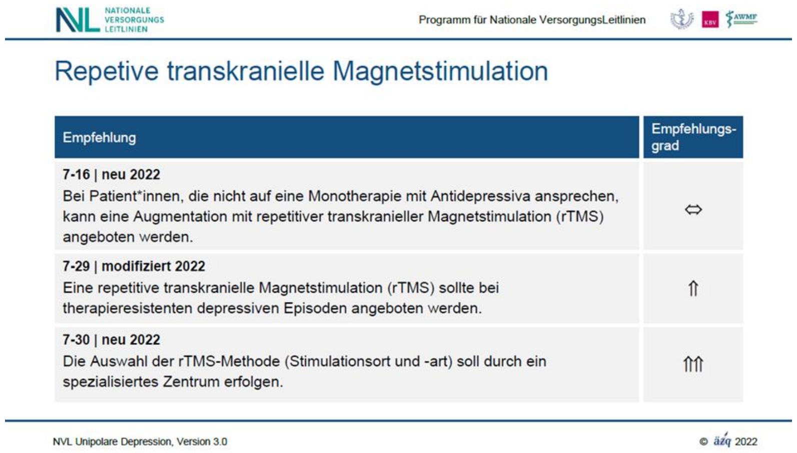
NATIONALE VERSORGUNGS LEITLINIEN

Programm für Nationale VersorgungsLeitlinien

Repetive transkranielle Magnetstimulation

Empfehlung	Empfehlungs-grad
**7-16 | neu 2022**<br>Bei Patient*innen, die nicht auf eine Monotherapie mit Antidepressiva ansprechen, kann eine Augmentation mit repetitiver transkranieller Magnetstimulation (rTMS) angeboten werden.	⇔
**7-29 | modifiziert 2022**<br>Eine repetitive transkranielle Magnetstimulation (rTMS) sollte bei therapieresistenten depressiven Episoden angeboten werden.	⇑
**7-30 | neu 2022**<br>Die Auswahl der rTMS-Methode (Stimulationsort und -art) soll durch ein spezialisiertes Zentrum erfolgen.	⇑⇑

NVL Unipolare Depression, Version 3.0

© äzq 2022

Treatment of depression

Deutsche Gesellschaft für Hirnstimulation in der Psychiatrie e. V.

- Also possible for bipolar disorder
- Low-frequency right frontal stimulation possible
- Bifrontal stimulation possible
- iTBS also possible
- Therapeutic effect independent of age

Pre-treatment consultation

Pre-treatment consultation

Deutsche Gesellschaft für
Hirnstimulation
in der Psychiatrie e. V.

- Verbal consultation via **medical staff** (a pre-treatment consultation sheet can be helpful)
- Documentation of the consultation
- Plan a consideration period (depending on the complexity of the case)
- Information must include not only chances of success, procedure, risks and side effects, but also possible treatment alternatives
- Indication should be determined by **specialized medical staff** (psychiatry/neurology with qualifications in brain stimulation procedures as per training regulations or medical societies such as DGPPN and/or DGHP)

Pre-treatment consultation

Deutsche Gesellschaft für Hirnstimulation in der Psychiatrie e. V.

- In the absence of FDA/CE approval, there should be documentation of off-label consultation
- In the case of depression with comorbid disorders, the emphasis should be on depression as an indication, with treatment following a depression protocol.
- Wording aid: „*The patient sought rTMS treatment following prior diagnosis of depression with inadequate improvement despite current multimodal therapy. The patient received comprehensive information, both verbally and in writing, about the treatment's indication, effects and side effects. There are no contraindications. The primary objective of the treatment is to alleviate depressive symptoms… time to consider…*"

Safety issues

Deutsche Gesellschaft für
Hirnstimulation
in der Psychiatrie e. V.

European experts' consensus (papers) on the use of TMS:

- **always:** risk-benefit analysis is necessary (as in any therapy decision)
- documentation of the analysis
- informing the patient about risks and expected benefits
- for special patient groups, the risk-benefit assessment may shift towards risk, but TMS is often still possible

Wassermann (1998) - doi: 10.1016/s0168-5597(97)00096-8
Rossi et al. (2009) - doi: 10.1016/j.clinph.2009.08.016
Rossi et al. (2021) - doi:10.1016/j.clinph.2020.10.003

Safety issues

Deutsche Gesellschaft für Hirnstimulation in der Psychiatrie e. V.

- Contraindications
- Side effects
- Special settings
- Secure treatment parameters

Contraindications

- Metal implants
- Electrical implants (e.g. pacemaker, insulin pump)
- Epilepsy and neurological conditions

- Absolute vs. relative contraindications

Implants

- Devices that are ferromagnetic, electrically operated, and/or reliant on detecting/generating electrical pulses for their activity
- Individual risk assessment (material, distance, relevance, and urgency of the indication)
- Metallic tattoos directly under the coil are problematic
- Dental implants and piercings are unproblematic
- MRI compatibility is merely a **guideline**
- Responsibility remains with the operator!

Pre-exisiting cerebral damage

- Broad term, not very helpful
- Type, extent, and location are crucial
- Issue no. 1: seizure risk
- Issue no. 2: reduced effectiveness
- Case-by-case decision!

Possible side effects

In order of frequency

- Local sensory paresthesia (nerves in the scalp and head muscles)
- Headaches, neck pain (usually self-limiting, respond well to pain relievers)
- Motor manifestations of the facial nerve (eye/forehead/nose/ear)
- Dizziness

Possible side effects

Deutsche Gesellschaft für
Hirnstimulation
in der Psychiatrie e. V.

More problematic, but much less common:

- Acoustic side effects
- Manic switch
- Induced seizures and syncope

Induced seizures and syncopes

Deutsche Gesellschaft für
Hirnstimulation
in der Psychiatrie e. V.

- Induced seizure: in practice, low risk (approximately 2-8 per 100,000 treatments) (in comparison background incidence in base population 50 per 100,000)
- Likely biased by patient selection and reporting bias
- In high-risk patients: approximately 30 per 100,000 (>60% in the first session)
- Many co-factors: triggers, drugs, medications(!), sleep, etc.
- Critical for evaluation: **consequences** of the seizure, differentiating between epilepsy and an induced seizure

Lerner et al. (2019) - doi:10.1016/j.clinph.2019.03.016

Induced seizures and syncopes

Deutsche Gesellschaft für
Hirnstimulation
in der Psychiatrie e. V.

- Knowledge in detection and first response, availability of medication and medical personnel
- Much more common, but also rare: (pre)syncopal events - recognizing the difference (recovery phase)!
- “Basic life support"
- Equipment: benzodiazepines, emergency alert system

Local side effects

- Clinically benign, but most relevant side effect during treatment:
  - local headaches (tension headaches)
  - treatment through the scalp muscles is the most uncomfortable (motor threshold vs. frontal stimulation)
  - every patient experiences pain differently, so clinical intuition is a must

Noise levels and electromagnetic field exposure

Deutsche Gesellschaft für Hirnstimulation in der Psychiatrie e. V.

- High noise levels (up to a maximum of 125dB at 25cm, up to 139dB at a 5cm distance)
- Always offer hearing protection and document refusal accordingly (highly relevant for tinnitus treatment)
- Long-term effects of electromagnetic exposure: no reports so far, more relevant for operators than patients

Switch into mania and suicide risk

Deutsche Gesellschaft für Hirnstimulation in der Psychiatrie e. V.

- A switch into mania, increased suicidality, or overall symptom worsening can occur
- These aspects are clinically and legally relevant, however they are not specific to TMS
- This underscores the importance of indication, informed consent, and a final assessment (!) by psychiatrists or psychologically trained medical professionals

„TMS dip“

- “Exacerbation" of symptoms after 2 weeks (possible reason: patient’s expectations and symptom fixation)
- Does not necessitate a termination of the treatment, as the suggested therapy duration is at least 3 weeks

Cognitive side effects

Deutsche Gesellschaft für
Hirnstimulation
in der Psychiatrie e. V.

- Relevant, as patients often ask - evidence is however limited
- TMS often conceptually confused with ECT by patients
- No evidence in literature nor practice, but not completely ruled out
- Many confounders (medication, depression itself)
- Majority of studies describe cognitive improvement (Rossi et al., 2021)!
- Driving is possible, no waiting period needed after session

Rossi et al. (2021) - doi: 10.1016/j.clinph.2020.10.003

Special patient groups

Deutsche Gesellschaft für Hirnstimulation in der Psychiatrie e. V.

- Pregnancy and breastfeeding
- Medication

Pregnancy and breastfeeding

Deutsche Gesellschaft für Hirnstimulation in der Psychiatrie e. V.

- 3 controlled studies and several dozen case reports
- Case-by-case assessment
- From physical standpoint, TMS shows minimal potential for harm – unlike pharmacological treatments
- CAUTION: risk of publication bias
- CAUTION: given the emotionally charged and legally relevant nature of the situation, special care must be taken during documentation (particularly for off-label use)
- CAUTION: explicit consultation of potential harm to the child in case of epileptic seizure

Co-medication

Deutsche Gesellschaft für Hirnstimulation in der Psychiatrie e. V.

- rTMS is highly compatible with other therapies and has no special pharmaceutical considerations - **except** for benzodiazepines due to their impact on the seizure threshold.
- Goal: maintaining a stabile medication plan
- Neurotropic medications have been the focus thus far
- Anticonvulsants and mood stabilizers: limited evidence
- Antipsychotics may diminish treatment success
- Benzodiazepines may also diminish treatment success
- CAUTION: trials face methodological challenges, as prospective randomization is difficult and numerous potential confounders exist

Treatment safety guidelines

Deutsche Gesellschaft für Hirnstimulation in der Psychiatrie e. V.

- Safety guidelines according to Wassermann et al. (1998) and Rossi et al. (2009)
- **CAUTION: pay attention to manufacturer specifications and manuals**
- Safety depends on intensity, frequency, and inter-train intervals
  - painful above 60% of the stimulator output!
  - at high frequencies breaks are necessary!
  - induced seizures have been reported with single pulses, low frequencies and in healthy individuals. Not just with supposedly dangerous theta-burst and high-frequency stimulations!

Wassermann (1998) - doi: 10.1016/s0168-5597(97)00096-8
Rossi et al. (2009) - doi: 10.1016/j.clinph.2009.08.016

Treatment safety guidelines

Deutsche Gesellschaft für Hirnstimulation in der Psychiatrie e. V.

- maximum safe duration of a train (outside of the motor cortex)

Maximum safe duration (expressed in seconds) of single trains of rTMS. Safety defined as absence of seizure, spread of excitation or afterdischarge of EMG activity. Numbers preceded by > are longest duration tested. Consensus has been reached for this table.

Frequency (Hz)	Intensity (% of MT)				
	90%	100%	110%	120%	130%
1	>1800[a]	>1800	>1800	>360	>50
5	>10	>10	>10	>10	>10
10	>5	>5	>5	4.2	2.9
20	2.05	2.05	1.6	1.0	0.55
25	1.28	1.28	0.84	0.4	0.24

[a] In Japan, up to 5000 pulses have been applied without safety problems (communication of Y. Ugawa).

Treatment safety guidelines

Deutsche Gesellschaft für
Hirnstimulation
in der Psychiatrie e. V.

- minimum safe duration of an inter-train interval (motor cortex)

Adapted from Table 4 (Part A) and Table 3 (part B) of Chen et al., 1997, with permission from the authors. Safety recommendations for inter-train intervals for 10 trains at <20 Hz. The maximum duration of pulses for individual rTMS trains at each stimulus intensity should not exceed those listed in the Part B of the table. A consensus has been reached in adopting this table at this point. However, there is a need to extend these investigations and provide more detailed guidelines that may apply also to non-motor areas.

Inter-train interval (ms)	Stimulus intensity (% of MT)			
	100%	105%	110%	120%
Part A				
5000	Safe	Safe	Safe	Insufficient data
1000	Unsafe (EMG spread after 3 trains)	Unsafe[a]	Unsafe (EMG spread after 2 trains)	Unsafe (EMG spread after 2 trains)
250	Unsafe[a]	Unsafe[a]	Unsafe (EMG spread after 2 trains)	Unsafe (EMG spread after 3 trains)

Frequency (Hz)	100%		110%		120%		130%	
	Duration (s)/pulses		Duration (s)/pulses		Duration (s)/pulses		Duration (s)/pulses	
Part B								
1	>270	>270	>270	>270	>180	>180	50	50
5	10	50	10	50	10	50	10	50
10	5	50	5	50	3.2	32	2.2	22
20	1.5	30	1.2	24	0.8	16	0.4	8
25	1.0	25	0.7	17	0.3	7	0.2	5

[a] These stimulus parameters are considered unsafe because adverse events occurred with stimulation of lower intensity or longer inter-train interval, but no adverse effects were observed with these parameters.

Treatment safety guidelines

Deutsche Gesellschaft für
Hirnstimulation
in der Psychiatrie e. V.

theta burst stimulation

Published TBS (biphasic pulses) and QPS (monophasic pulses) protocols on normal subjects. No significant side effects reported, apart vagal reactions after prefrontal cortex stimulation. Consensus reached for this table.

	Pulses in the burst	Total train pulses	Intensity	Stimulation site
"Standard" cTBS (following Huang et al. 2005)	3 at 50 Hz, repeated at 5 Hz	600 (40 s)	80% of active MT	Motor cortex, PFC[c]
Silvanto et al. 2007	8 at 40 Hz, repeated every 1.8 s	200	60% of the maximal stimulator output	Visual cortex
Nyffeler et al. 2006[a]	3 at 30 Hz, repeated at 10 Hz	200	80% of resting MT	Frontal eye fields
"Standard" iTBS protocols (following Huang et al. 2005)	3 at 50 Hz, repeated at 5 Hz for 2 s	600	80% of active MT	Motor cortex, PFC[c]
QPS [b] (following Hamda et al., 2008)	4 (ISI ranging 1.5 ms–1.25 s), repeated every 5 s	1440	90% of active MT	Motor cortex

[a] Also repeated TBS in the same session (at 5, 15, 60, 75 min).
[b] 2000 maximal total pulse number per day; highest intensity used resting MT (Y. Ugawa, personal communication).
[c] PFC = prefrontal cortex (Grossheinrich et al. 2009).

Treatment safety guidelines

Deutsche Gesellschaft für
Hirnstimulation
in der Psychiatrie e. V.

- HOWEVER (Rossi et al., 2021)!

„Despite such variety, as reviewed for these guidelines, neither seizure occurrence nor other AEs emerged consistently, thus indicating that whatever the protocol of intervention, the technique can be considered basically safe. Therefore, we have decided not to provide a formal update of the previous safety tables, and that, instead, we propose ''operational guidelines''. Clearly, the parameters of stimulation used for MST [magnetic seizure therapy] should not be exceeded.

The usual lowest parameters of stimulation to induce seizures during MST are 100% of maximal stimulator output (at least for these commercially available devices), frequency of 25 Hz, delivered in a single train lasting up to 10 s. Therefore, every combination of intensity/frequency/duration of conventional rTMS treatment (when seizure induction is not the goal) must remain well below this combination of parameters.“

Rossi et al. (2021) - doi: 10.1016/j.clinph.2020.10.003

Billing

Deutsche Gesellschaft für
Hirnstimulation
in der Psychiatrie e. V.

Billing

	national health insurance (GKV)	private health insurance (PKV) and self-payers
in-patient	additional charges<br>• documentation, indication, medical specialist<br>• rTMS basic and therapy service	additional charges
out-patient	no uniform system in Germany<br><br>Bavaria: coding based on respective professional groups and devices	German fee catalog for physicians (GOÄ)<br><br>cost estimate or treatment contract

Additional charges (as of 2024)

Deutsche Gesellschaft für Hirnstimulation in der Psychiatrie e. V.

- the basic rTMS service (ZP75.01, OPS: 8-632.0, EUR 124.17) includes consultation, motor threshold assessment, and the first treatment; the pre-treatment consultation needs to be documented separately (<25 Minuten) and is billed separately.
- rTMS therapy session (ZP75.02, OPS: 8-632.1; 77.54 Euro) may take place multiple times a day
- as these additional charges are new (2021), regional German health insurance medical service (MDK) inspections will reveal further aspects to consider over time
- when documenting and providing cost estimates, it is important to mention therapy resistance, the severity of depression, and the necessity of TMS

Deutsche Gesellschaft für
Hirnstimulation
in der Psychiatrie e. V.

Additional charges - ICD codes

- 8-63 Electrostimulation of the nervous system
- 8-632 Repetitive transcranial magnetic stimulation (rTMS)
- 8-632.0 Basis service
  - basic service covers the following: indication, pre-treatment consultation, planning, and carrying out the first therapy session with repetitive transcranial magnetic stimulation
  - don‘t code this extra
  - code to be used only once per stationary visit
- 8-632.1 Therapy session
  - incudes maintenance rTMS
  - this code is to be documented once per therapy session regardless of the amount of stimulations

ICD-Code
OPS Suche

ICD OPS Impressum

OPS Suche

OPS-2024 Systematik online lesen

OPS-2024 > 8 > 8-63...8-66 > 8-63 > 8-632

8-63 Elektrostimulation des Nervensystems

8-632 Repetitive transkranielle Magnetstimulation [rTMS]

8-632.0 Grundleistung

Info: Zur Grundleistung gehören die fachärztliche Indikationsstellung, die Aufklärung, die Planung und die Durchführung der ersten Therapiesitzung mit repetitiver transkranieller Magnetstimulation.
Diese ist nicht gesondert zu kodieren
Dieser Kode ist nur einmal pro stationären Aufenthalt anzugeben

8-632.1 Therapiesitzung

Inkl.: Erhaltungs-rTMS

Info: Dieser Kode ist unabhängig von der Gesamtzahl der Stimulationen einmal pro Therapiesitzung anzugeben

8-632.y N.n.bez.

Private health insurance and self-payers

- cost estimate or treatment contract
- present evidence, medical guidelines, and the non-responder nature of the case
- billing according to the German fee catalog for physicians (GOÄ) - possible codes for motor threshold determination and treatment
  - 839a (electromyographic examination, 93.84 euros)
  - 828 (evoked potentials, 81.11 euros)

Transcranial magnetic stimulation

German Society for Brain Stimulation in Psychiatry (DGHP; registered society)

Hands-On Workshop Refresher April 2025

Deutsche Gesellschaft für
Hirnstimulation
in der Psychiatrie e. V.

Overview (1.5h theory & 1.5h hands-on)

Deutsche Gesellschaft für Hirnstimulation in der Psychiatrie e. V.

What?	Where?	How Long?
Theory: Indications and Treatment Protocols, Accelerated Treatment, Maintenance and Relapse Prevention, Questions		1.5 hours
Hands-On		1.5 hours
Conclusion		

Indications and treatment protocols

Deutsche Gesellschaft für
Hirnstimulation
in der Psychiatrie e. V.

Indications and treatment protocols

Deutsche Gesellschaft für
Hirnstimulation
in der Psychiatrie e. V.

	treatment	German guidelines	European guidelines	approvals	state of research
**Unipolar/ bipolar depression**	F3, 10/20Hz, 100-120%, 1500-3000 pulses, 20-30 sessions (*alternatively: F3, iTBS, 80%, 600 pulses, 20-30 sessions*) (Hebel et al., 2022)	"*should be offered*" recommendation in cases of therapy resistance "*can be offered*" recommendation after unsuccessful monotherapy	definitely effective	FDA, CE	numerous positive meta-analyses available

Response rate: approximately 10-50%!
80% stimulation intensities have also been reported!

Indications and treatment protocols

Deutsche Gesellschaft für Hirnstimulation in der Psychiatrie e. V.

	treatment	German guidelines	European guidelines	approvals	state of research
**Negative symptoms in schizophrenia**	Protocol analogous to depression treatments<br><br>(Lefaucheur et al., 2014; 2020)	"*can be offered*" recommendation in cases of therapy resistance as part of an overall treatment plan<br><br>"patients should be informed about the high rate of potential non-response."	possibly effective	no	further evidence required

Indications and treatment protocols

Deutsche Gesellschaft für Hirnstimulation in der Psychiatrie e. V.

	treatment	German guidelines	European guidelines	approvals	state of research
**auditory hallucinations in schizophrenia**	CP5, 1Hz, 80-100%, 1000/1200 pulses, 10 sessions<br>(or CP5 and CP6, cTBS, 80%, 600 pulses each, 15-20 sessions)<br>(Lefaucheur et al., 2014; 2020; Plewnia et al., 2018; Ye et al., 2024)	"should be offered" recommendation in cases of therapy resistance as part of an overall treatment plan	possibly effective	no	further evidence required

Indications and treatment protocols

Deutsche Gesellschaft für
Hirnstimulation
in der Psychiatrie e. V.

	treatment	German guidelines	European guidelines	approvals	state of research
**chronic tinnitus**	CP5, 1Hz, 110%, 2000 pulses, 10 sessions<br>(Folmer et al., 2015; Lefaucheur et al., 2014; 2020)	"*should **NOT** be offered*" recommendation against TMS (dissenting opinion of the DGPPN: "*may be considered*")	possibly effective	no	further evidence required

Indications and treatment protocols

	treatment	German guidelines	European guidelines	approvals	state of research
**obsessive-compulsive disorder**	SMA, 1Hz, 100% (leg), min. 1200 pulses, 15-30 sessions, with double cone coil if necessary<br><br>(or F4, 1Hz, 110% MRI, at least 1200 pulses, 15-30 sessions)<br><br>(Fitzsimmons et al., 2022)	"*can be offered*" recommendation for short-term symptom relief in cases of therapy resistance	possibly effective	FDA (in com-bination with exposi-tion), CE	protocol inconsistency, further evidence required

Indications and treatment protocols

Deutsche Gesellschaft für Hirnstimulation in der Psychiatrie e. V.

	treatment	German guidelines	European guidelines	approvals	state of research
**addiction**	treatment analogous to depression (Lefaucheur et al., 2014)	no recommendation possible	possibly effective with nicotine	FDA, CE for sub-stances	further evidence needed
**post-traumatic stress disorder**	F4, 1 vs. 20Hz, 80-120%, 100-4000 pulses, 10-30 sessions (Boggio et al., 2010)	rTMS is not mentioned	*probably* effective	no	further evidence required

Coil cositioning - DLPFC (F3)

Deutsche Gesellschaft für Hirnstimulation in der Psychiatrie e. V.

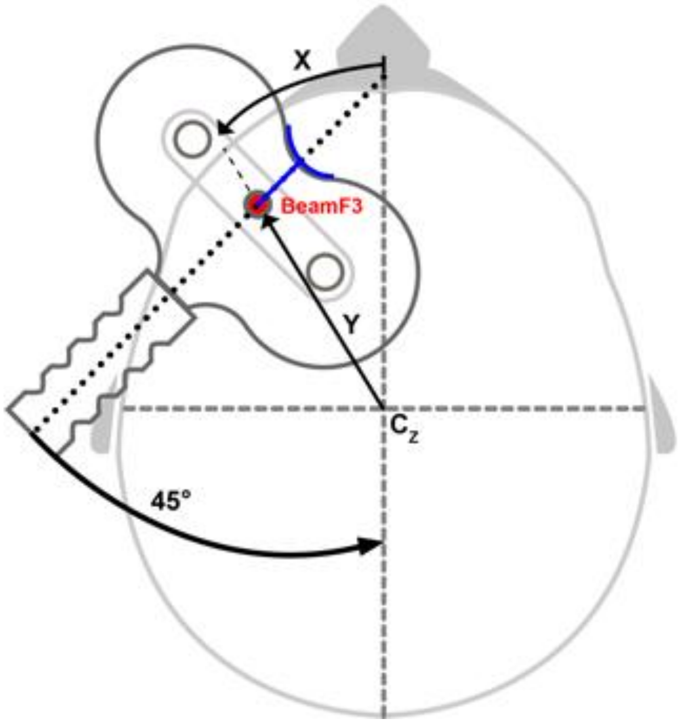

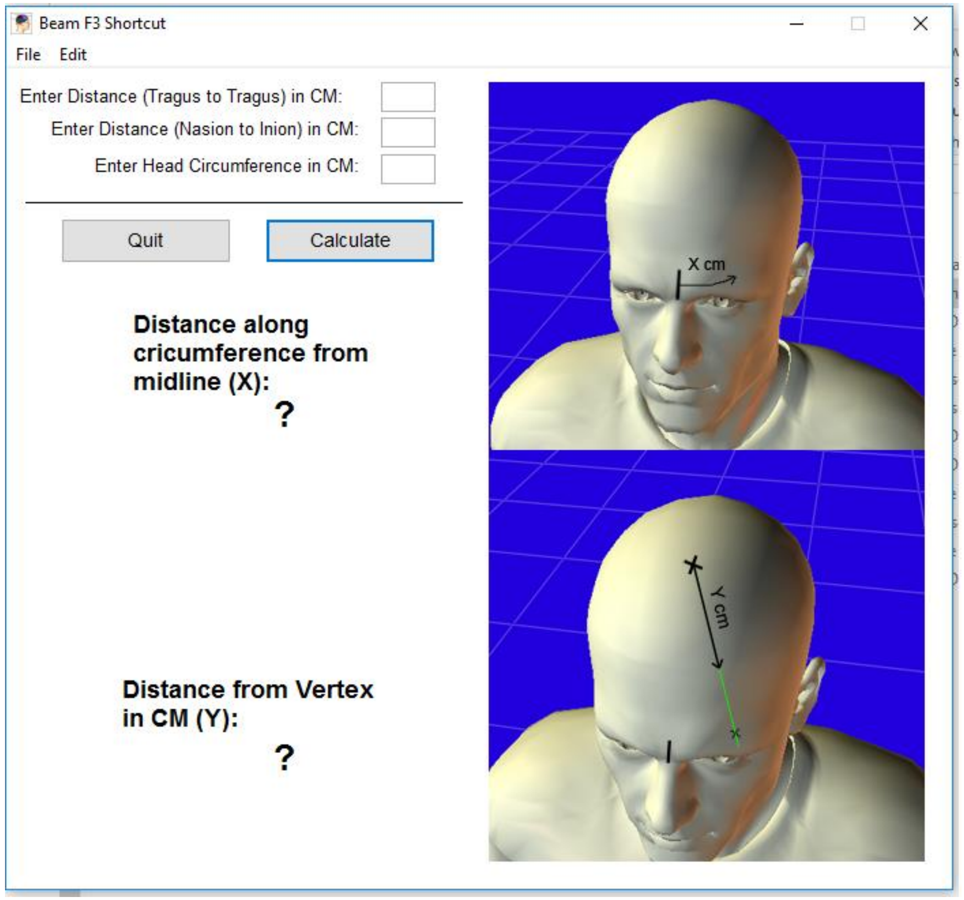

Beam et al. (2009) - doi: 10.1016/j.brs.2008.09.006

Coil positioning - TPJ (CP5)

Deutsche Gesellschaft für
Hirnstimulation
in der Psychiatrie e. V.

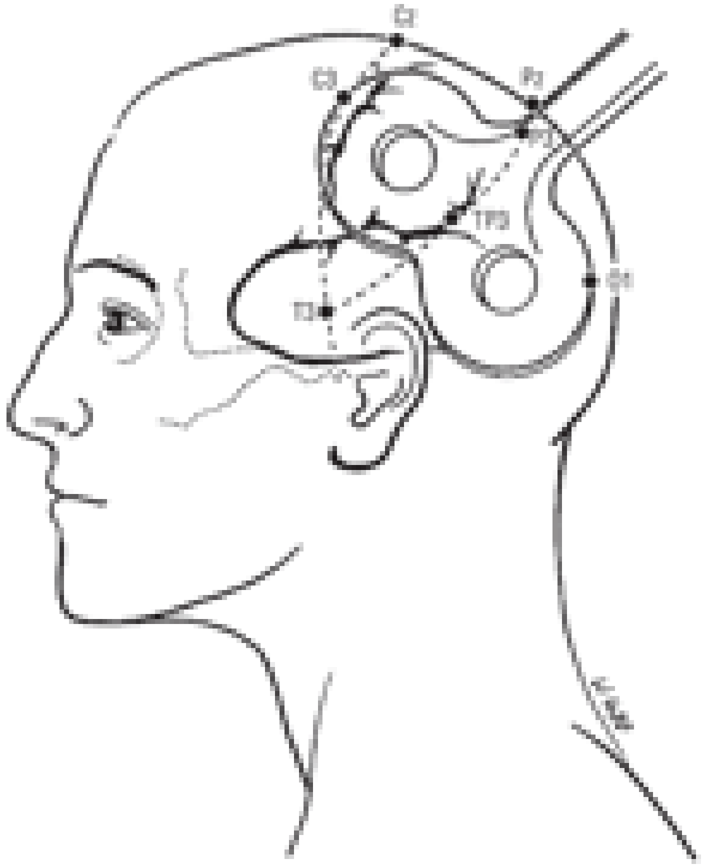

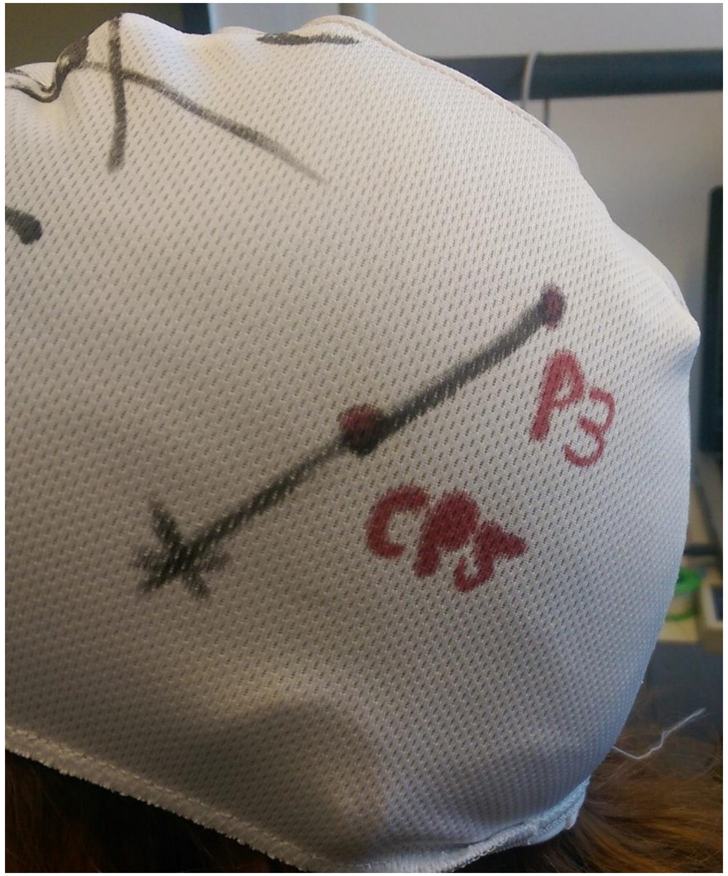

Hoffman et al. (2003) - doi: 10.1001/ARCHPSYC.60.1.49

Coil positioning - SMA (FFCz)

Deutsche Gesellschaft für
Hirnstimulation
in der Psychiatrie e. V.

- starting at Cz, move 15% of the length of the nasion-inion distance forward, while staying on the midline
- the coil handle should point backwards

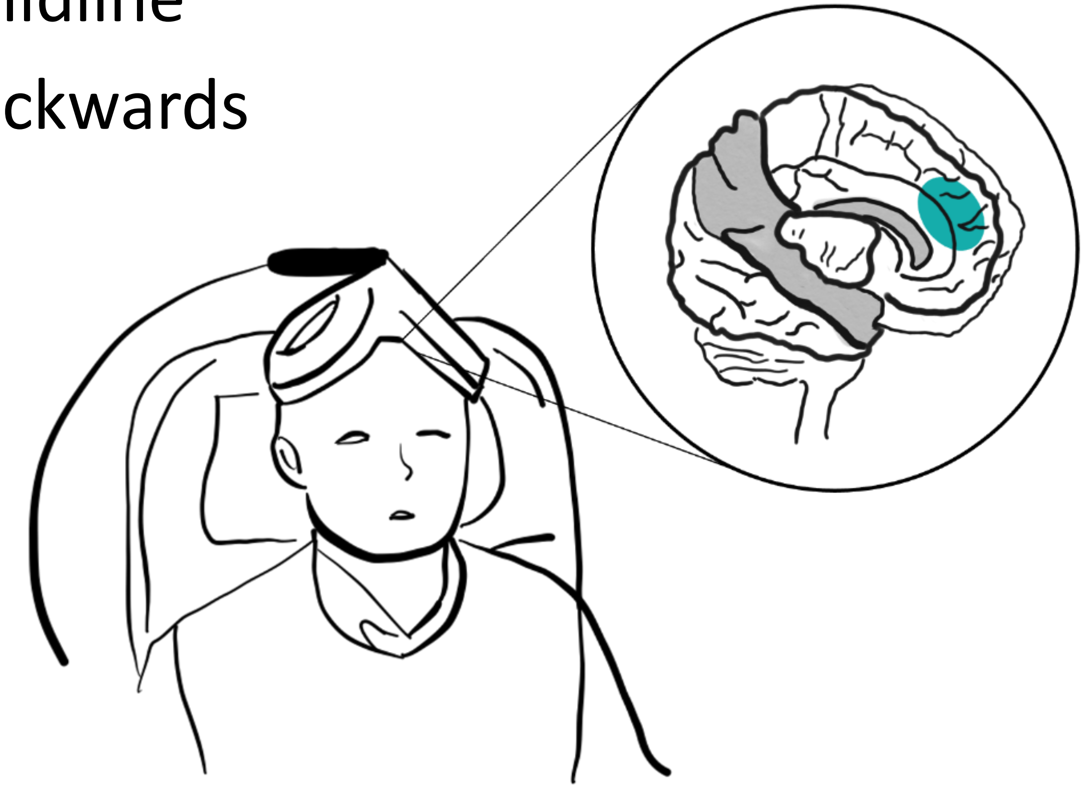

Accelerated treatment

Deutsche Gesellschaft für
Hirnstimulation
in der Psychiatrie e. V.

Accelerated treatment

- faster response
- iTBS instead of 10Hz (intermittent theta-burst stimulation)
- multiple sessions per day (15-50 minutes apart)
- justify deviations from the standard

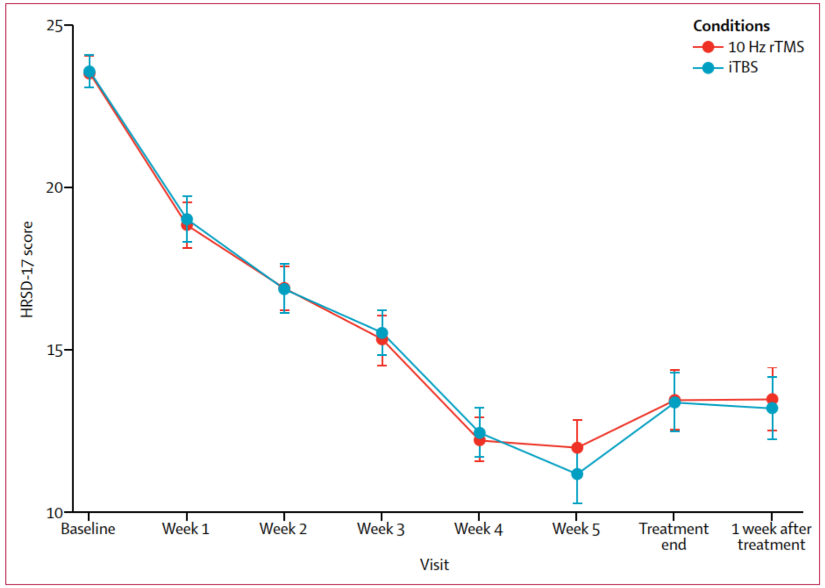

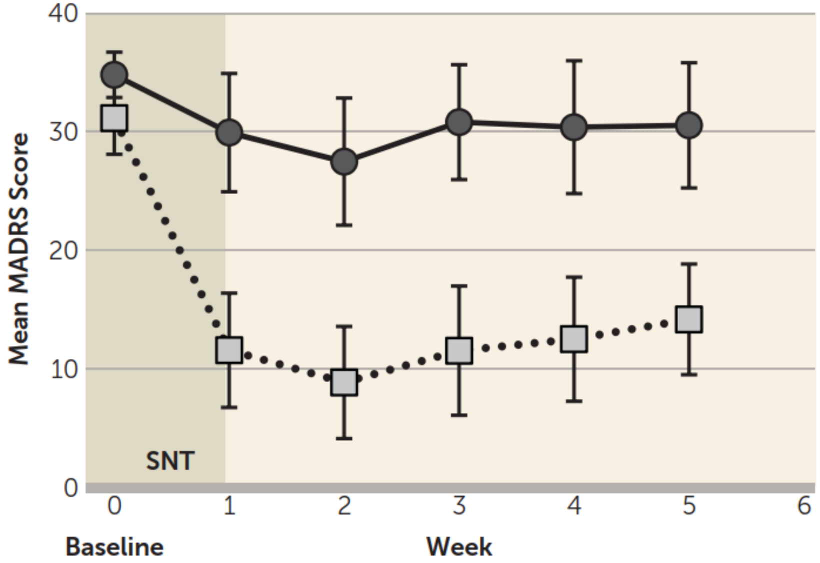

Blumberger et al. (2018) - doi: 10.1016/S0140-6736(18)30295-2
Cole et al. (2021) - doi: 10.1176/appi.ajp.2021.20101429

Accelerated treatment

Deutsche Gesellschaft für
Hirnstimulation
in der Psychiatrie e. V.

- faster response
- iTBS instead of 10Hz (intermittent theta-burst stimulation)
- multiple sessions per day (15-50 minutes apart)
- justify deviations from the standard

- open questions:
  - is more better? (number of pulses/sessions)
  - is neuronavigation necessary?
  - few positive placebo-controlled studies

Maintenance and relapse prevention

Deutsche Gesellschaft für
Hirnstimulation
in der Psychiatrie e. V.

- application after successful rTMS (remission/response)
- plenty of data on effectiveness (naturalistic setting)
- little data on efficacy (controlled studies)
- two possible models
  - tapering of treatment sessions
  - booster sessions
  - example: LMU

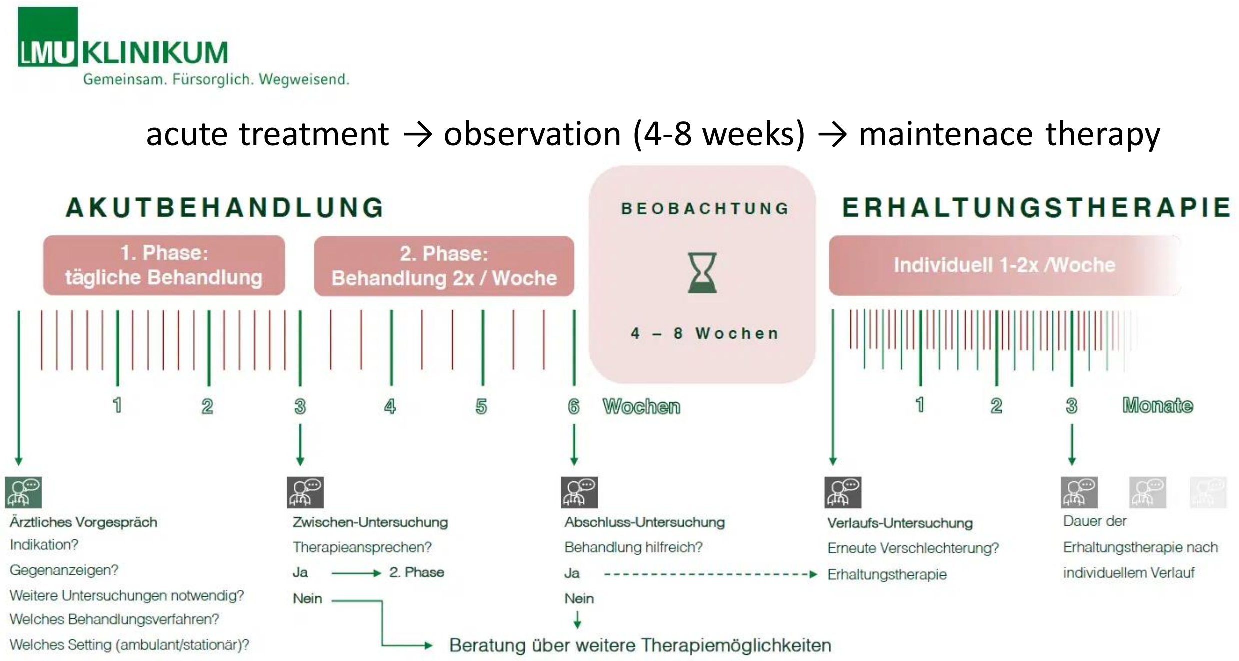

Supplement: Supplementary file 1 [file SupplementaryFile1.zip › Workshop (English).PDF]
