# Supplementary material for: rTMS for the treatment of psychiatric disorders: a review about training courses and materials and the presentation of the training materials of the German Society for Brain Stimulation in Psychiatry
Source: Front Psychiatry. 2025 Aug 8;16:1490039. doi: 10.3389/fpsyt.2025.1490039 (PMC12371536; doi:10.3389/fpsyt.2025.1490039)
Supplement: Supplementary file 1 [file SupplementaryFile1.zip › Practical Guideline (German).PDF]

Deutsche Gesellschaft für Hirnstimulation in der Psychiatrie e. V.

# Praktische Anleitung zur Vorbereitung der Transkraniellen Magnetstimulation in der Psychiatrie

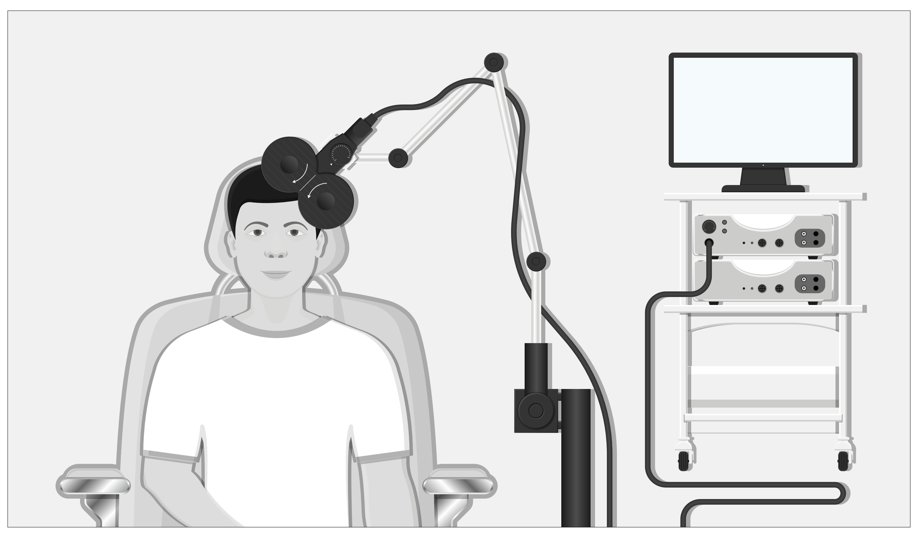

## Material und Basics

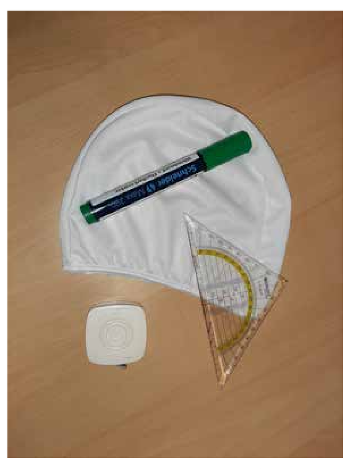
Markierungs-Material

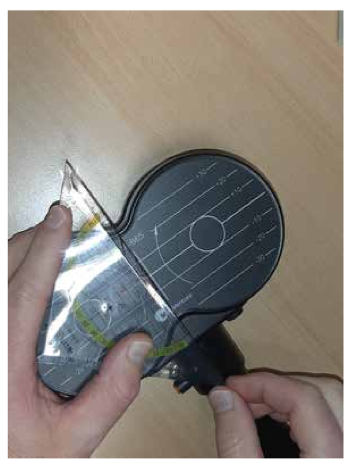
Abstand Spulenmitte zu -spitze der Behandlungsspule notieren

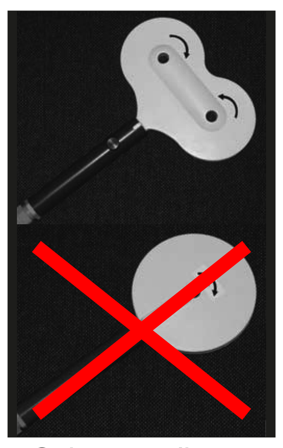
Schmetterlings-spule verwenden

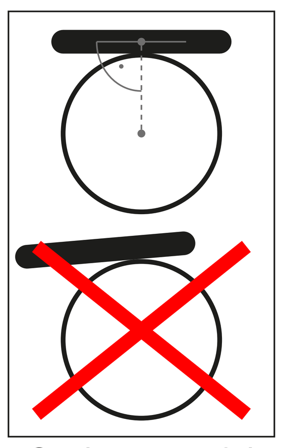
Spule tangential und mittig auflegen

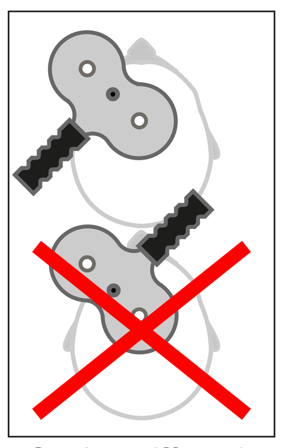
Spulengriff nach hinten orientieren

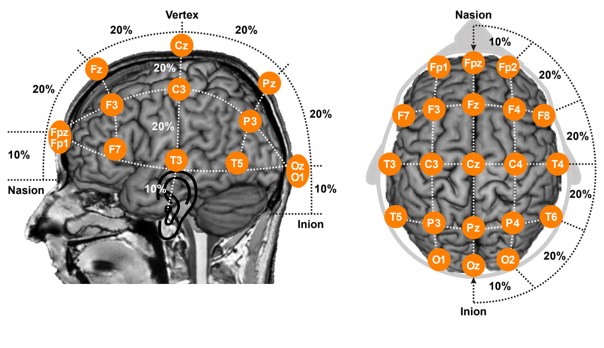

EEG–10–20-System als Basis für die Markierungen

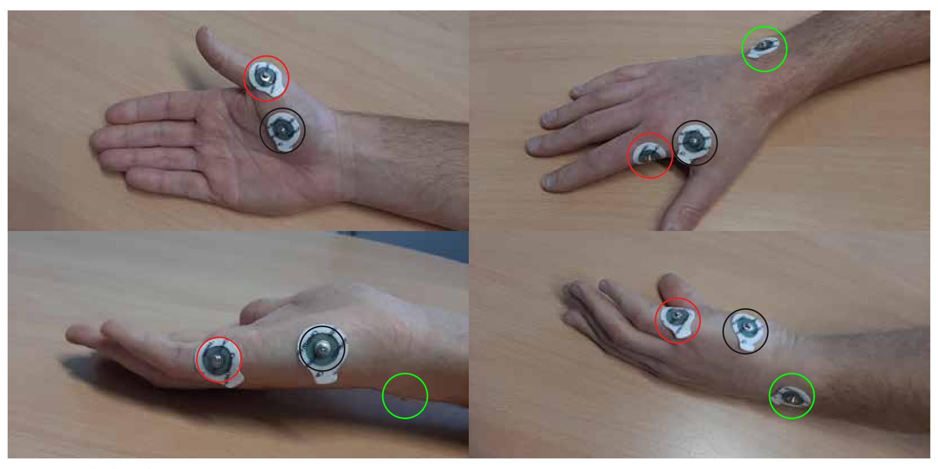
EMG vorbereiten mit Elektroden am Muskelbauch und Sehnenansatz (*belly-tendon* Montage) des rechten Daumenballens, Zeigefinges oder Kleinfingers

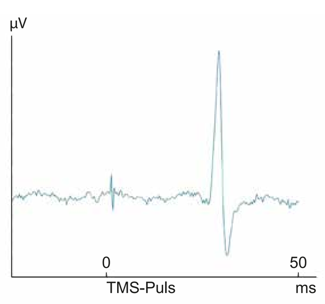

Motorisch Evoziertes Potential

## Vorbereitung

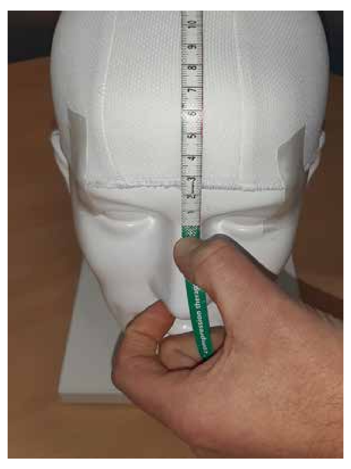
Abstand Nasion–Haubenrand messen und notieren

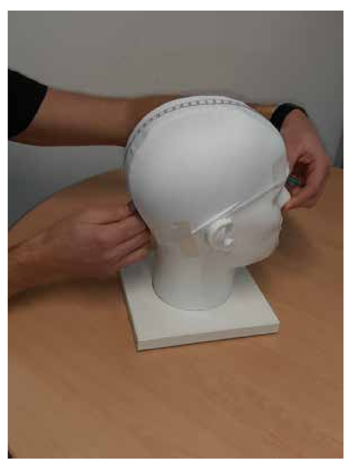
Abstand Nasion–Inion messen und notieren

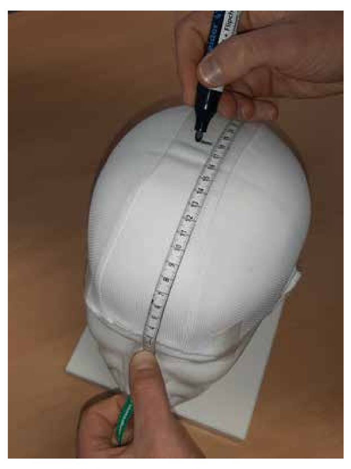
50% der Strecke Nasion–Inion einzeichnen

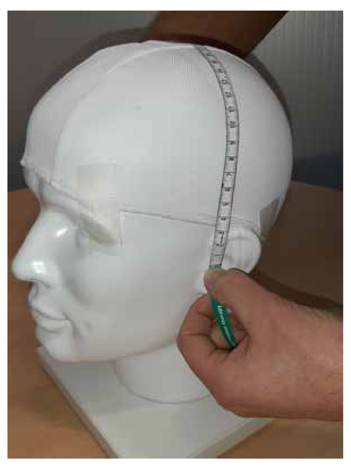
Abstand Tragus–Tragus messen und notieren

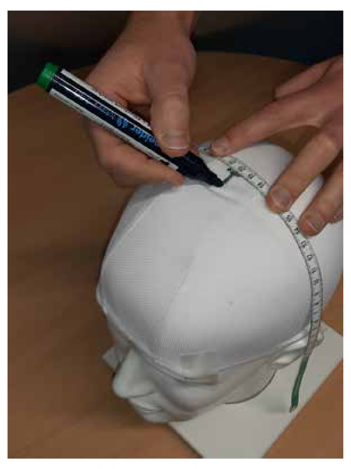
50% der Strecke Tragus–Tragus einzeichnen

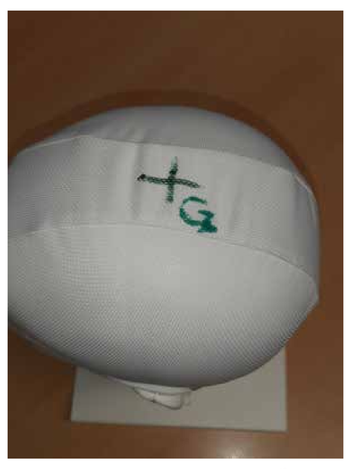
Kreuzung ergibt Cz

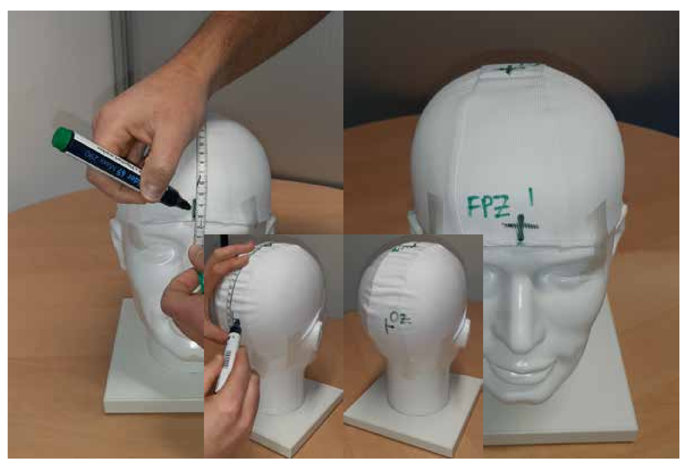
10% der Strecke Nasion–Inion vom Nasion (Fpz) und vom Inion (Oz) einzeichnen

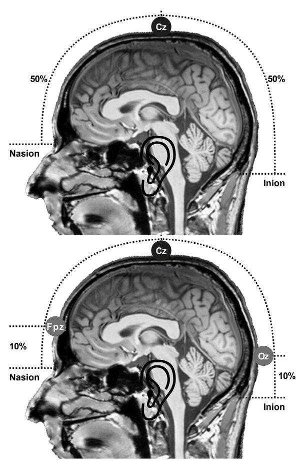
Schematische Darstellung Cz, Fpz und Oz

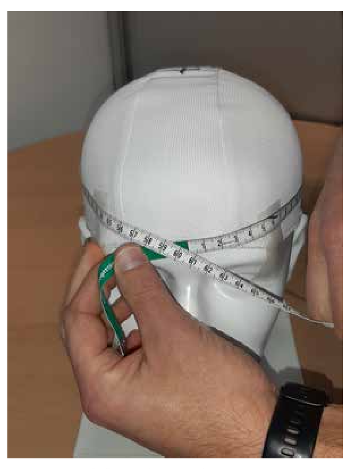
Kopfumfang durch Fpz und Oz messen und notieren

## Motorischen Hotspot ausmessen

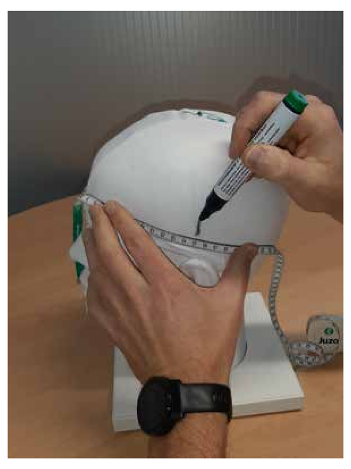
50% des halben Kopfumfangs einzeichnen

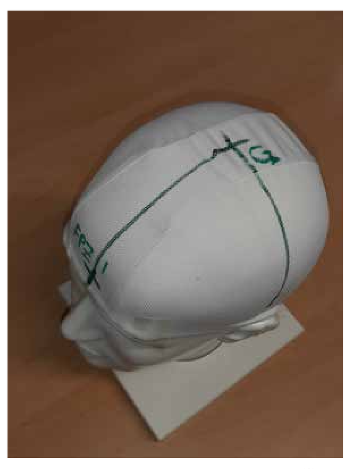
Verbindungslinie zu Cz und Verbindungslinie Fpz–Cz einzeichnen

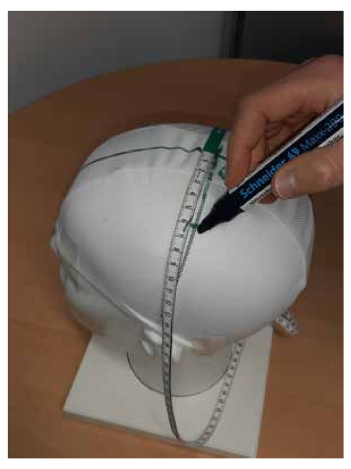
15% der Strecke Tragus–Tragus nach links lateral einzeichnen

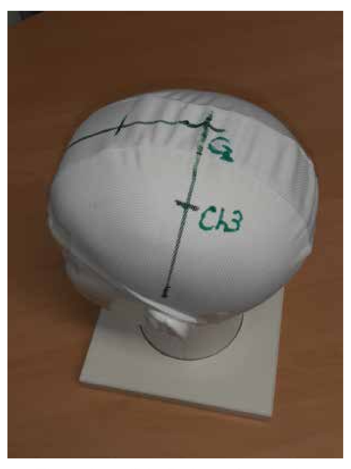
Punkt Ch3 ist (mittig zwischen C1 und C3) optimaler Startpunkt

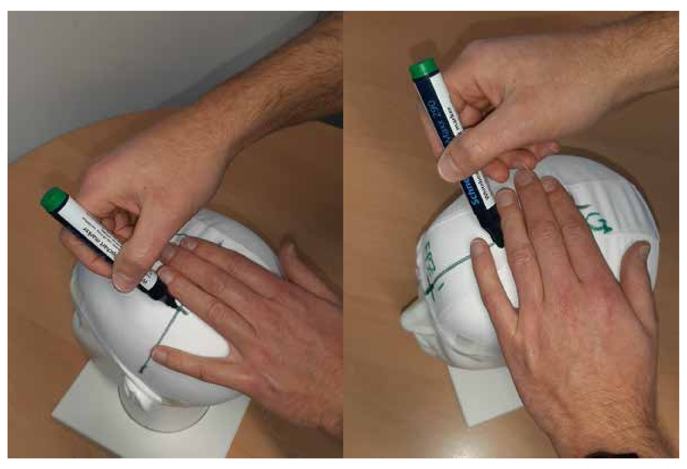
Alternativ von Cz ausgehend 5 cm nach lateral und auch 5 cm nach anterior messen oder 2/3 Fingerbreiten zur Seite und nach vorne messen

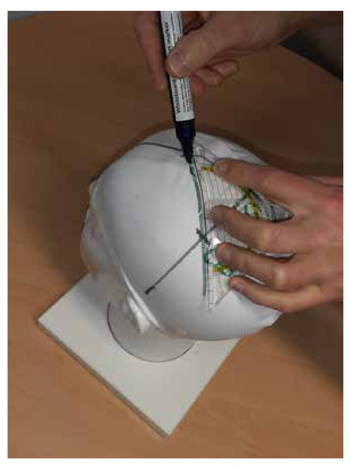
Verbindungslinie der Markierungen einzeichnen

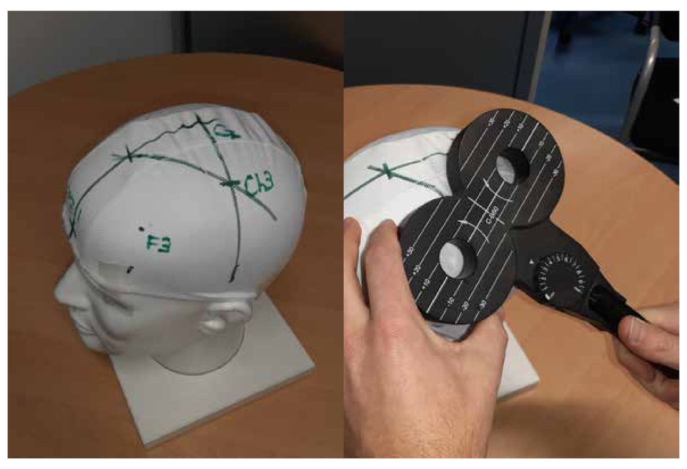
Spulenorientierung entlang der Verbindungslinie

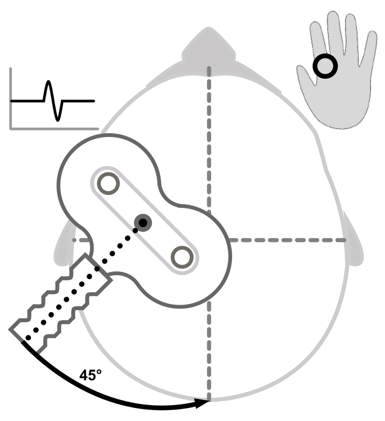

Schematische Darstellung der Bestimmung der Motorschwelle

## F3 im linken DLPFC ausmessen

http://clinicalresearcher.org/F3/calculate.php

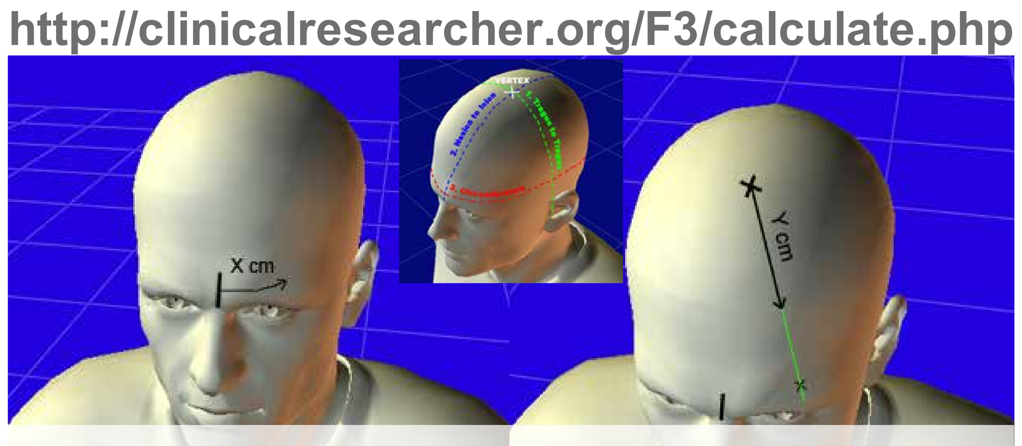

Eingabe der Maße Tragus–Tragus, Nasion–Inion, Kopfumfang und Ablesen der Ausgabemaße X und Y

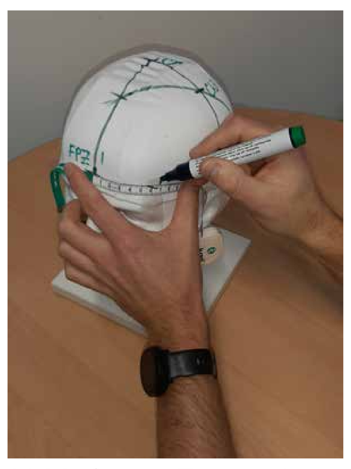
X-Maß von Fpz entlang des Kopfumfangs einzeichnen

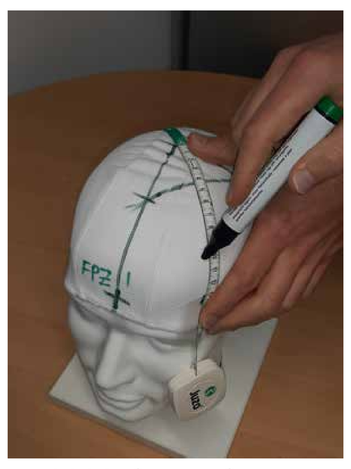
Y-Maß von Cz zu Markierung aus X einzeichnen

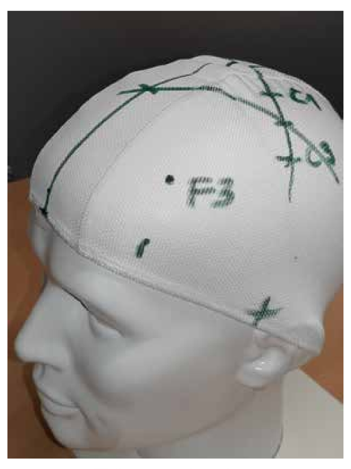
F3 nach der Beam-Methode ausgemessen

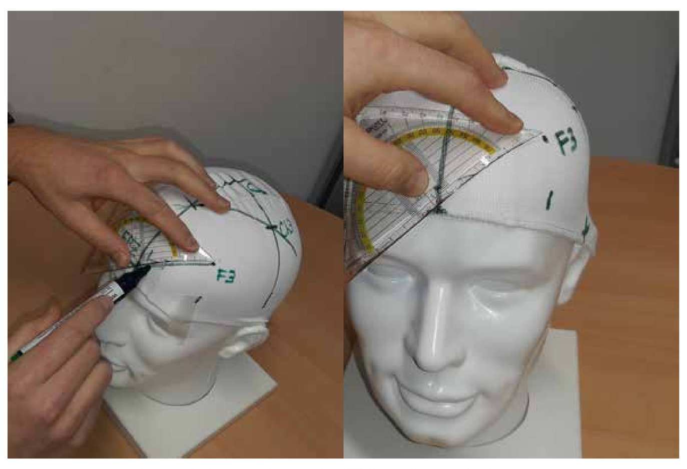
45°-Line durch F3 einzeichnen

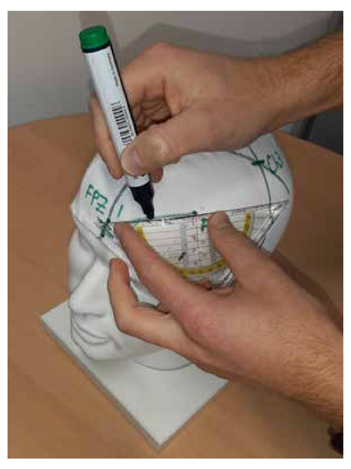
Spulenabstand von F3 an der 45°-Line einzeichnen

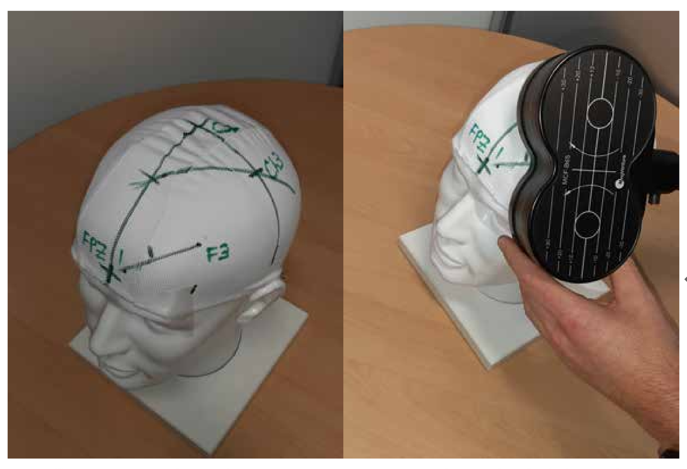
Spulenorientierung entlang der 45°-Line in Richtung Fpz oder Nasion

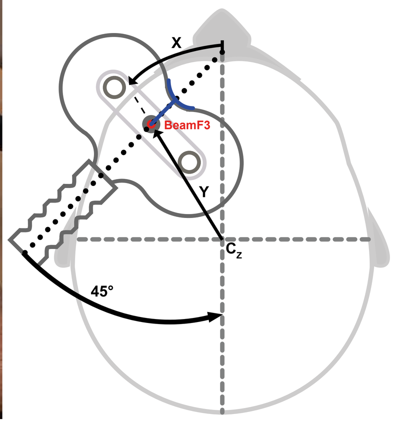

Schematische Darstellung der Beam F3-Lokalisation und Spulenorientierung

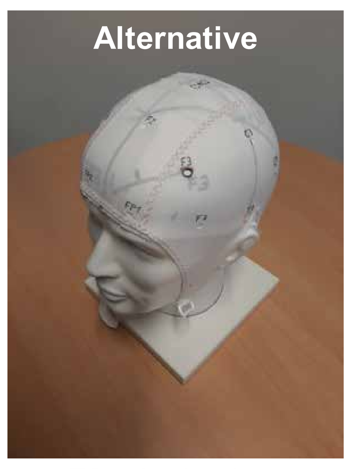

F3 mittels einer EEG-Leerhaube markiert

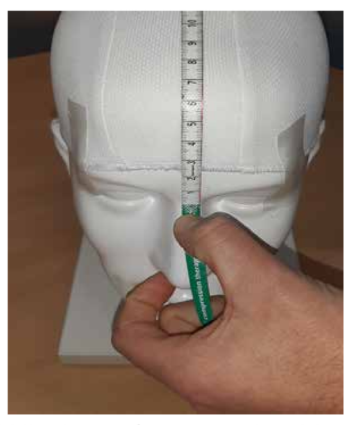
Beim Wiederaufsetzen der Haube an Haubennaht und Cz orientieren und auf Abstand Nasion–Haubenrand achten

## Andere Stimulationsziele

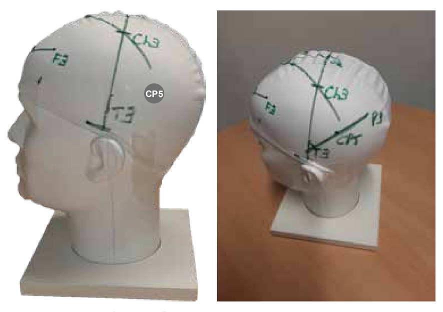
Position für Stimulation des linken temporoparietalen Cortex bei akustischen Phantomwahrnehmungen

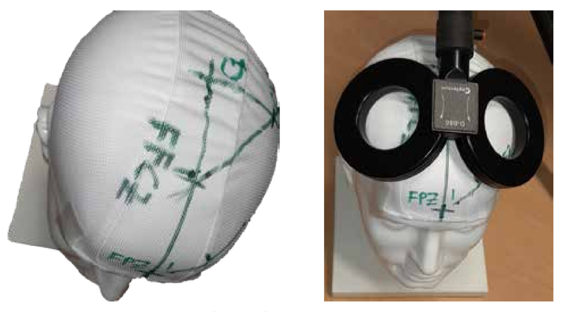
Position für Stimulation des supplementär-motorischen Areals bei Zwangsstörungen

## Platz für Notizen

## Autoren

Fotos: Martin Schecklmann
Layout: Katrin Sakreida
Beratung: Wolfgang Strube, Christiane Licht, Ulrike Vogelmann
2024

Anfrage für pdf-Datei an ksakreida@ukaachen.de
