# Supplementary material for: rTMS for the treatment of psychiatric disorders: a review about training courses and materials and the presentation of the training materials of the German Society for Brain Stimulation in Psychiatry
Source: Front Psychiatry. 2025 Aug 8;16:1490039. doi: 10.3389/fpsyt.2025.1490039 (PMC12371536; doi:10.3389/fpsyt.2025.1490039)
Supplement: Supplementary file 1 [file SupplementaryFile1.zip › Practical Guideline (English).PDF]

Deutsche Gesellschaft für Hirnstimulation in der Psychiatrie e. V.

Practical guideline for the preparation of transcranial magnetic stimulation in psychiatry

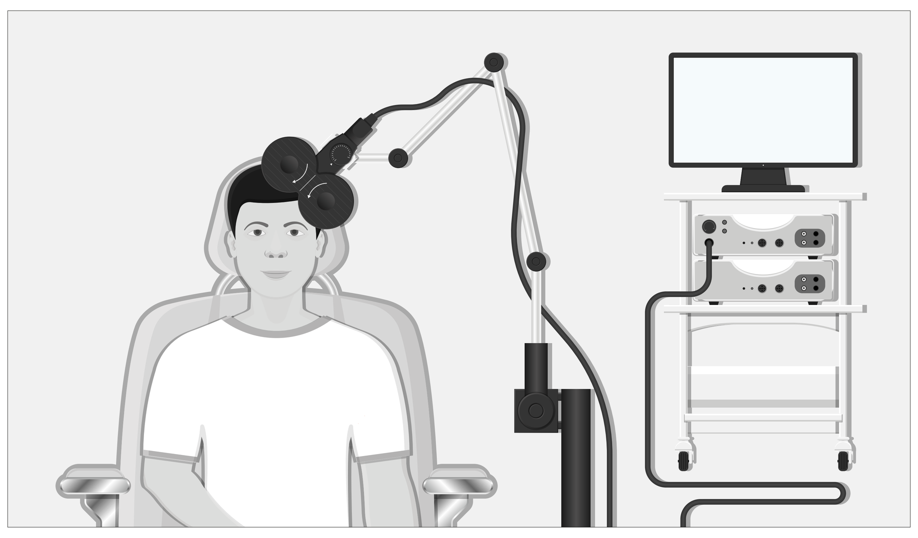

Material and Basics

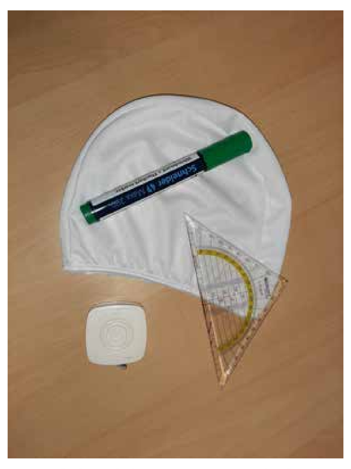
Marking material

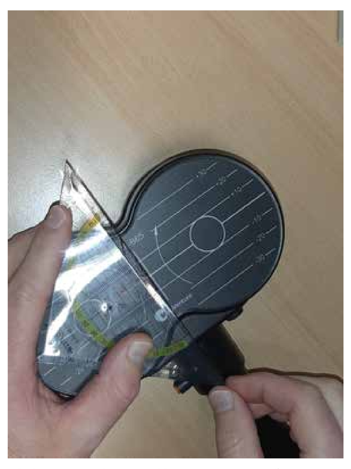
Note the distance from the centre to the tip of the treatment coil

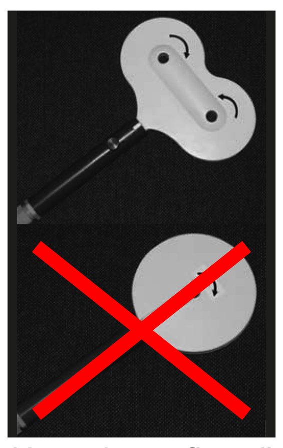
Use a butterfly coil

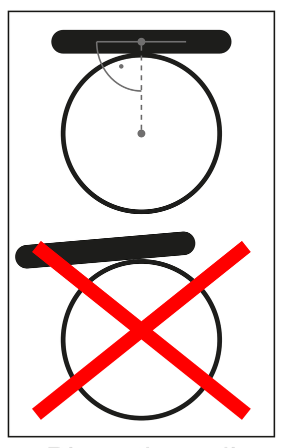
Place the coil tangentially and with centre point

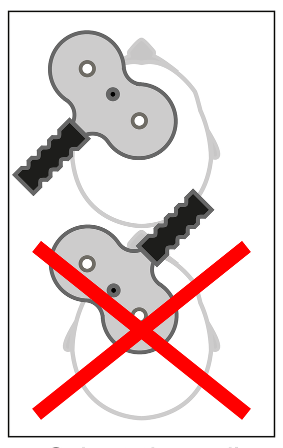
Orient the coil handle backwards

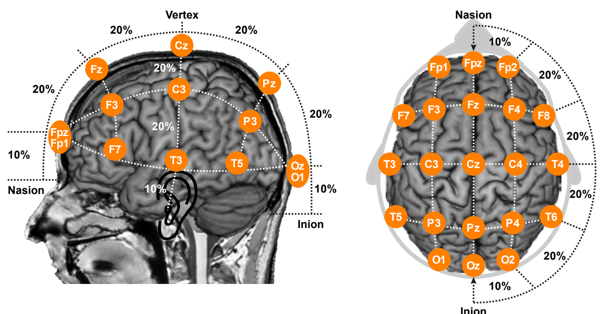
EEG–10–20 system as the basis for the markings

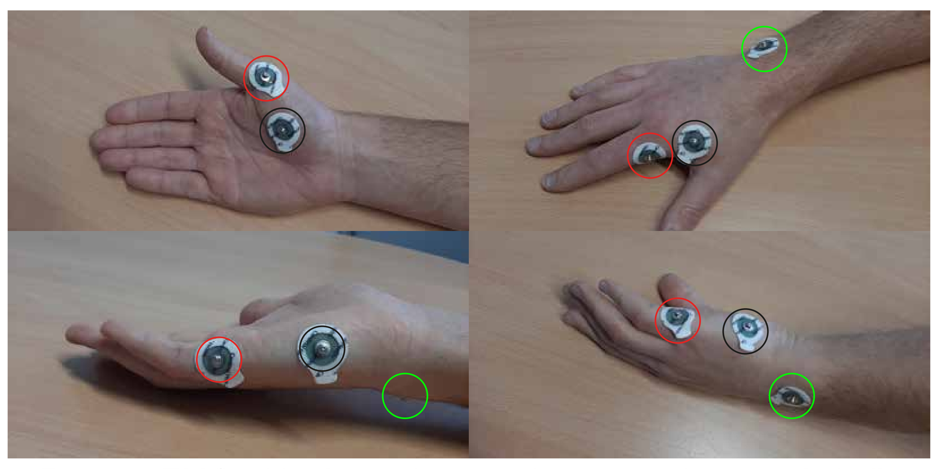
Prepare EMG with electrodes attachment on the muscle belly and tendon (*belly-tendon* montage) of the right thumb ball, index finger, or little finger

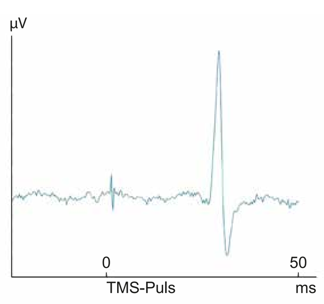

Motor evoked potential

Preparation

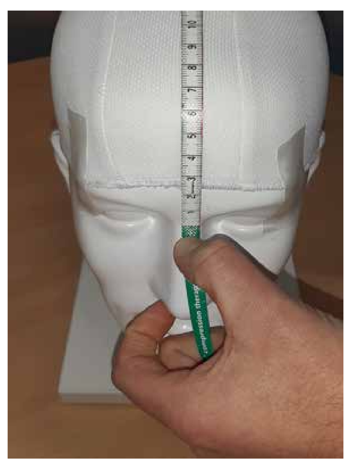
Measure and note the distance nasion–edge of the cap

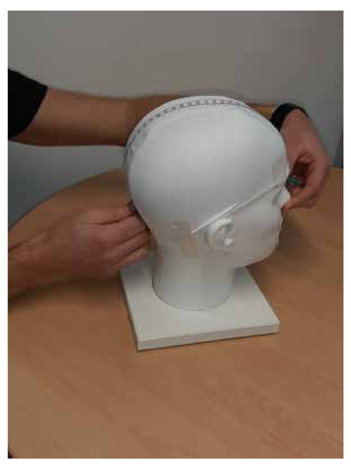
Measure and note the distance nasion–inion

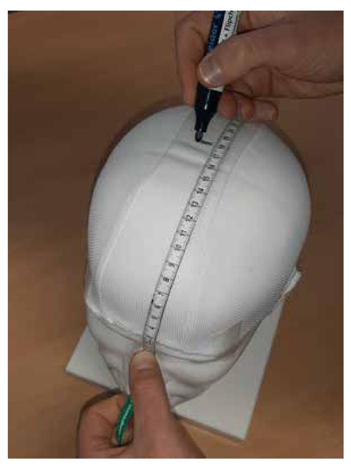
Mark 50% of the distance nasion–inion

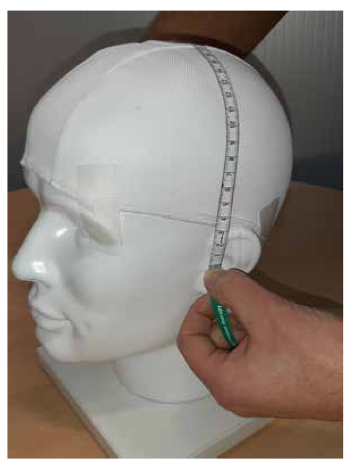
Measure and note the distance tragus–tragus

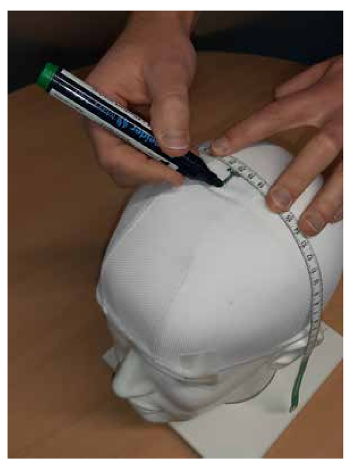
Mark 50% of the distance tragus–tragus

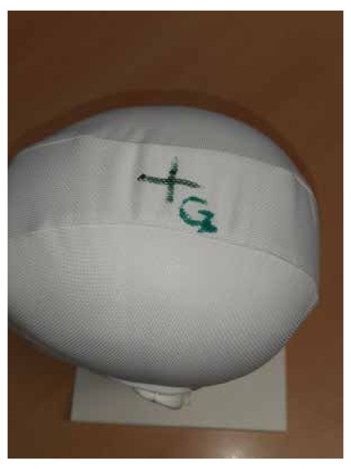
Crossing results in Cz

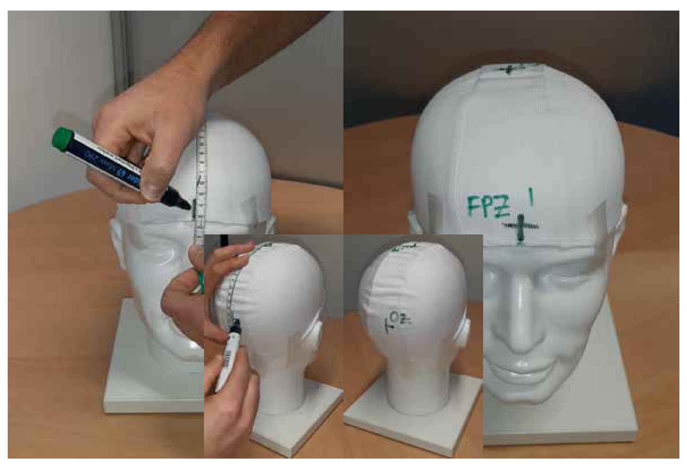
Mark 10% of the distance nasion–inion from nasion (Fpz) and from inion (Oz)

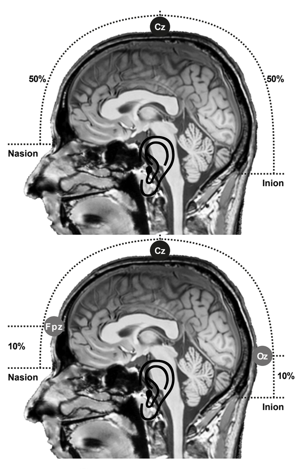
Schematic illustration Cz, Fpz, and Oz

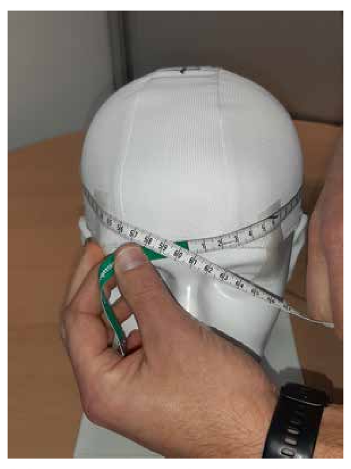
Measure and note head circumference through durch Fpz and Oz

Measure motor hotspot

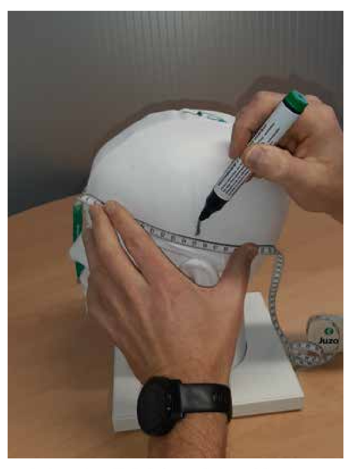
Mark 50% of half the head circumference

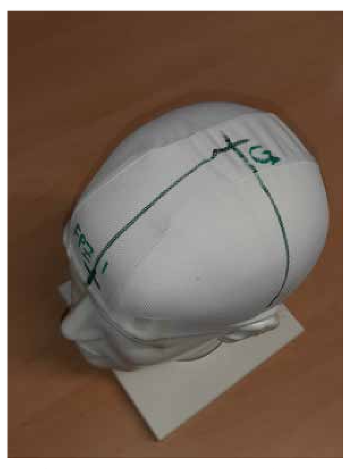
Draw the connecting line to Cz and connecting line Fpz–Cz

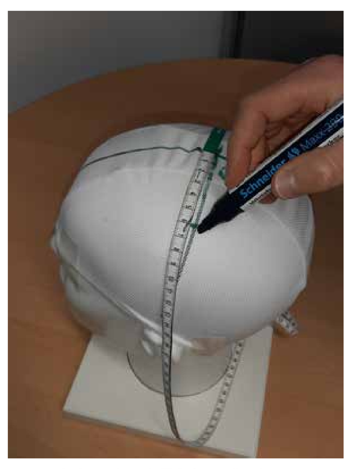
Mark 15% of the tragus–tragus distance to the left lateral side

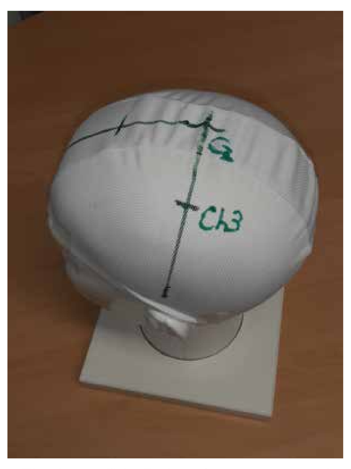
Point Ch3 is (centered between C1 and C3) optimal starting point

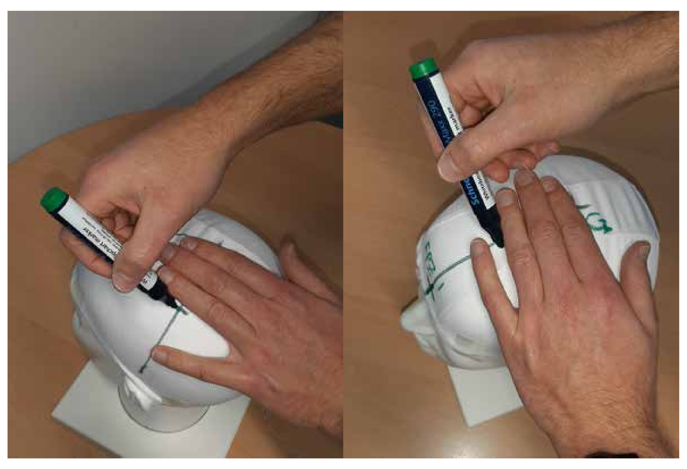
Alternatively, measure starting from Cz 5 cm laterally and also 5 cm anteriorly or measure 2/3 finger widths to the side and to the front

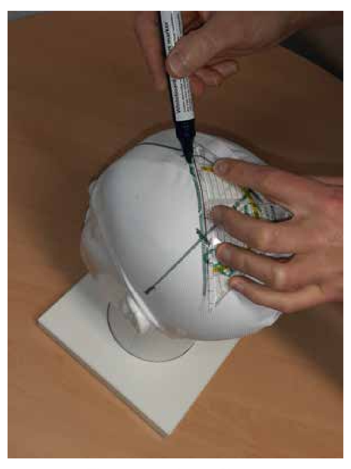
Draw the line connecting the markings

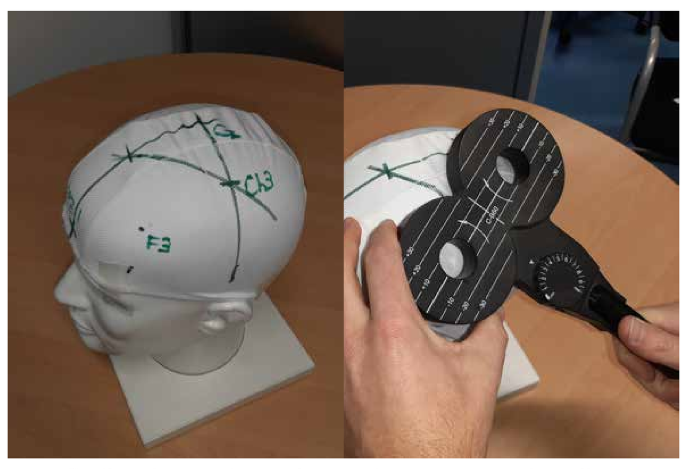
Coil orientation along the connecting line

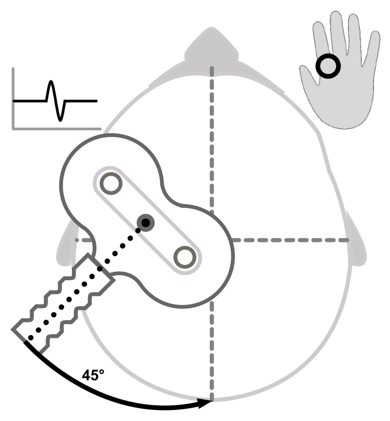

Schematic illustration of the motor threshold determination

Measure F3 in left DLPFC

http://clinicalresearcher.org/F3/calculate.php

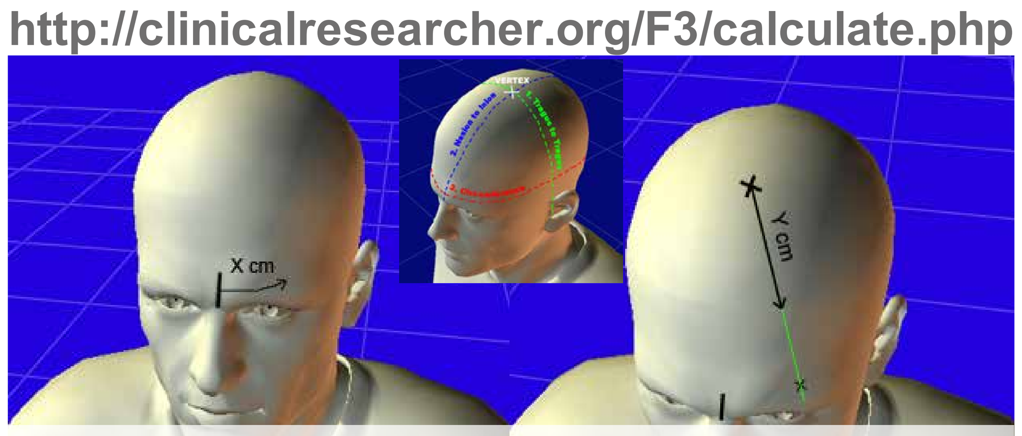

Enter the measurements tragus–tragus, nasion–inion, head circumference and read the output measurements X and Y

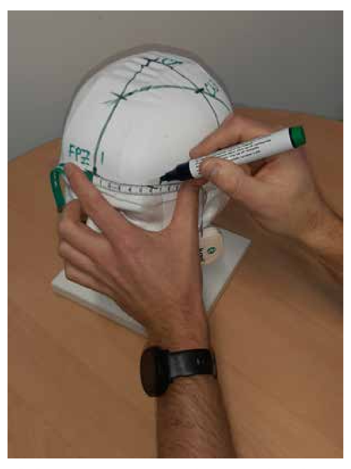
Measure and mark the X value from Fpz along the head circumference

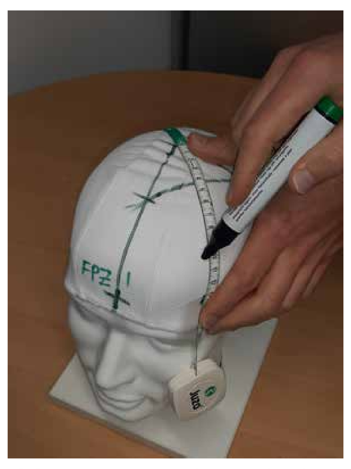
Measure and mark the Y value from Cz to the marking from X

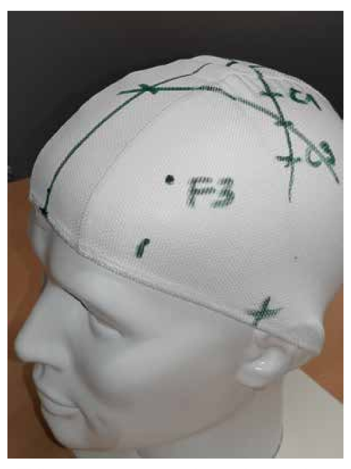
F3 measured using the Beam method

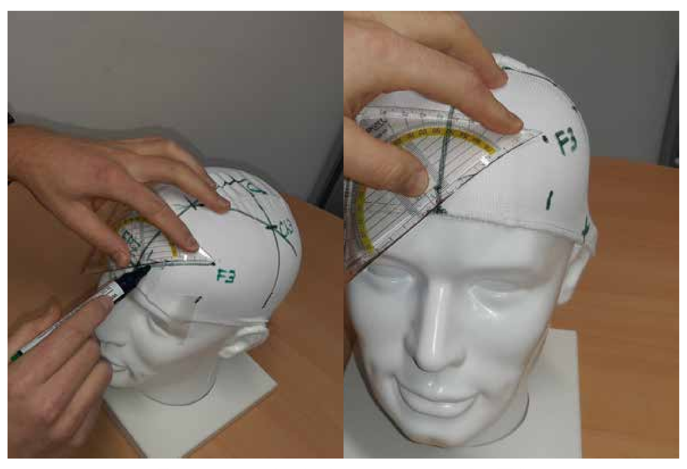
Draw a 45° line through F3

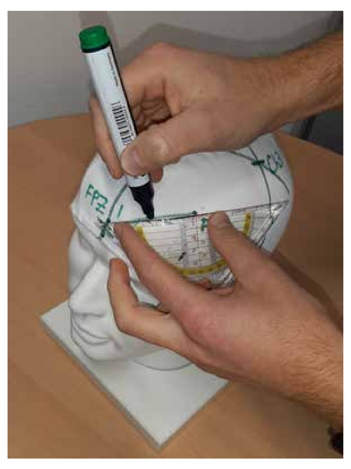
Mark the coil distance of F3 on the 45° line

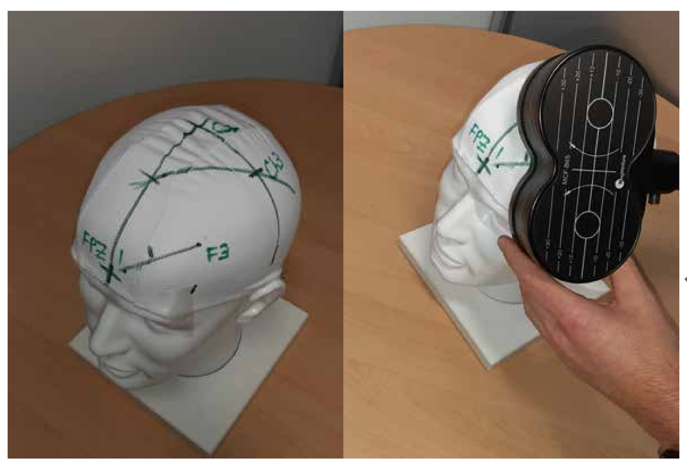
Coil orientation along the 45° line in the direction of Fpz or nasion

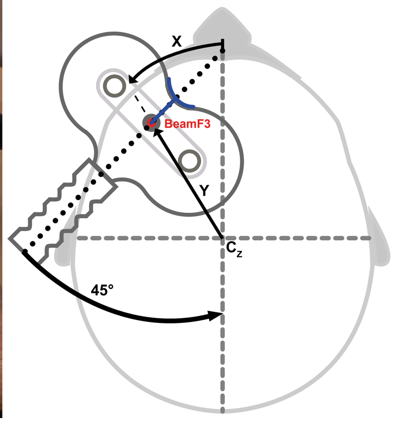

Schematic illustration of the Beam F3 localization and coil orientation

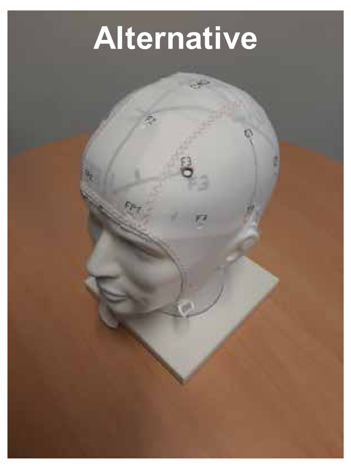

F3 marked using an EEG blank cap

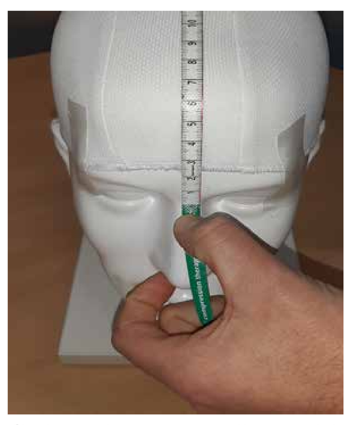
When refitting the cap, align with the cap seam and Cz and pay attention to the distance nasion–edge of the cap

Other stimulation targets

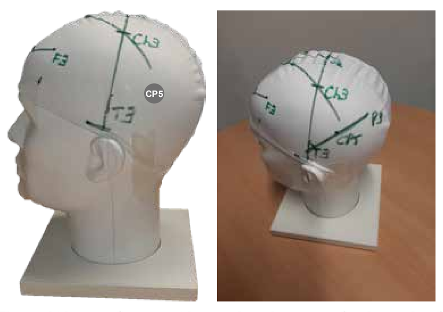
Position for stimulation of the left temporoparietal cortex in acoustic phantom perceptions

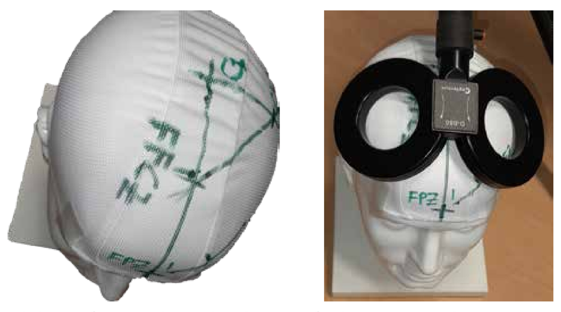
Position for stimulation of the supplementary motor area in obsessive-compulsive disorders

Space for notes

Authors

Photos: Martin Schecklmann
Layout: Katrin Sakreida
Consulting: Wolfgang Strube, Christiane Licht, Ulrike Vogelmann
2024

Request for pdf file to ksakreida@ukaachen.de
