# Supplementary material for: rTMS for the treatment of psychiatric disorders: a review about training courses and materials and the presentation of the training materials of the German Society for Brain Stimulation in Psychiatry
Source: Front Psychiatry. 2025 Aug 8;16:1490039. doi: 10.3389/fpsyt.2025.1490039 (PMC12371536; doi:10.3389/fpsyt.2025.1490039)
Supplement: Supplementary file 1 [file SupplementaryFile1.zip › Handbook (German).pdf]

Deutsche Gesellschaft für Hirnstimulation in der Psychiatrie e. V.

Repetitive Transkranielle Magnetstimulation (rTMS)

Ein Handbuch der Deutschen Gesellschaft

für Hirnstimulation in der Psychiatrie e.V. (DGHP)

Version vom April 2025

vorgelegt von Christiane Licht, Andreas Reissmann, Katrin Sakreida, Wolfgang Strube und Ulrike Vogelmann

(DGHP-Arbeitsgruppe „Klinischer Einsatz, Qualitätssicherung und Zertifizierung“)

unter Mitarbeit des Vorstands der DGHP sowie von Martin Schecklmann,

Stefanie Dierkes-Möller, Roberto Goya-Maldonado, Bernhard Kis, Michael Landgrebe,

Berthold Langguth, Tobias Hebel und

Carlos Schönfeldt-Lecuona

Dieses Manual erhebt keinen Anspruch auf Vollständigkeit

und drückt die Meinung der Autoren aus.

Relevante Abkürzungen

- DGHP = Deutsche Gesellschaft für Hirnstimulation in der Psychiatrie
- DLPFC = dorsolateraler präfrontaler Kortex (engl. dorsolateral prefrontal cortex)
- EEG = Elektroenzephalographie
- EMG = Elektromyographie
- GCP = gute klinische Praxis (engl. good clinical practice)
- GOÄ = Gebührenordnung der Ärzte
- MDK = Medizinischer Dienst der Krankenkassen
- MEP = motorisch evoziertes Potential
- MRT = Magnetresonanztomographie
- OPS = Operationen- und Prozedurenschlüssel
- PEPP = Pauschalisierte Entgelte für Psychiatrie und Psychosomatik
- RMT = Ruhemotorschwelle (engl. resting motor threshold)
- AMT = active Motorschwelle (engl. active motor threshold)
- SMA = supplementär-motorisches Areal
- rTMS = repetitive Transkranielle Magnetstimulation
- TMS = Transkranielle Magnetstimulation
- TPJ = temporoparietaler Übergangskortex (engl. temporoparietal junction)
- ZP = Zusatzentgelte

Inhaltsverzeichnis

1. Zusammenfassung

2. Rahmenbedingungen

3. Indikationen und Behandlungsprotokolle

Exkurs: Theta-Burst-Stimulation (TBS)

Exkurs: Beschleunigung der Behandlung

Exkurs: Aufrechterhaltungs-rTMS

Exkurs: rTMS-Indikationen jenseits der Depression

Tabelle: Indikationen der rTMS in der Psychiatrie abhängig von verschiedenen Evidenzleveln

4. Aufklärung, Kontraindikationen, Nebenwirkungen und besondere Personengruppen

4.1. Inhalte der Aufklärung

4.2. Kontraindikationen und besondere Personengruppen

4.3. Nebenwirkungen der rTMS-Behandlung

Exkurs: Risiken für Anwenderinnen und Anwender

5. Dokumentation und Vergütung

6. Spulenpositionierung und Spulentyp

Exkurs: EEG-Koordinaten

7. Relevante Spulenpositionen

7.1. Motorkortex inkl. Motorschwellenbestimmung

7.2. Dorsolateraler präfrontaler Kortex

7.3. Temporoparietaler Übergangskortex

7.4. Supplementär-motorisches Areal

8. Literaturverzeichnis

Anhang A: Textvorschlag Aufklärungsbogen zur Behandlung

Anhang B: Textvorschlag Abfrage von Nebenwirkungen

1. Zusammenfassung

Die repetitive Transkranielle Magnetstimulation (rTMS)…

- … ist eine nicht-invasive Hirnstimulationsbehandlung - beruhend auf gepulsten Magnetfeldern - zur Anregung neuroplastischer Prozesse
- … ist per se eine ärztliche Leistung, aber delegierbar
- … kann (teil)stationär oder ambulant durchgeführt werden
- … hat typischerweise eine ein- bis mehrwöchige Behandlungsdauer mit wiederholten, werktäglich stattfindenden Sitzungen
- … wird vor Beginn jeder Behandlungsserie flankiert durch die diagnostische Bestimmung der motorischen Ruheschwelle (als Basis zur Festlegung der Stimulationsintensität in den rTMS-Behandlungssitzungen)
- … kann bei zahlreichen Indikationen und unter Verwendung verschiedener Behandlungsprotokolle klinisch eingesetzt werden:
    - hochfrequente Behandlung des linken DLPFC bei der Depression (klare und beste Evidenz) und der Negativsymptomatik bei Schizophrenie (Kann-Empfehlung)
    - niederfrequente Stimulation des linken temporoparietalen Kortex bei akustischen Phantomwahrnehmungen und des supplementär-motorischen Areals bei Zwangsstörungen (Kann-Empfehlung)
- … hat (potenzielle) Nebenwirkungen:
    - Sensationen an der Stimulationsstelle und Lautstärke (Tragen von Gehörschutz)
    - gelegentlich Kopfschmerzen
    - äußerst selten: Krampfanfall oder Synkope
- … sollte bei (relativen) Kontraindikationen ggf. nicht zum Einsatz kommen:
    - kaum absolute Kontraindikationen
    - individuelle Risiko-Nutzen-Abschätzung
    - besondere Umsicht bei elektromagnetischen Implantaten und neurologischen Vorerkrankungen

2. Rahmenbedingungen

Die repetitive Transkranielle Magnetstimulation (rTMS) ist eine auf starken Magnetfeldern beruhende nicht-invasive Hirnstimulationsbehandlung, die im (teil-)stationären oder ambulanten Kontext durchgeführt werden kann. Die Behandlung besteht aus wiederholten Sitzungen, die über mehrere Tage bis Wochen hinweg durchgeführt wird. Typischerweise erfolgt die Stimulation an Werktagen, eine Stimulation am Wochenende ist nicht nötig. Vor jeder Behandlungsserie wird die Stimulationsintensität anhand der sogenannten Motorischen Ruheschwelle (RMT; engl. resting motor threshold) bestimmt. Die rTMS ist per se eine ärztliche Leistung, die an nicht-ärztliches Personal delegiert werden kann. Rahmenbedingung für die Anwendung und Abrechnung geben u.a. die Vereinbarung über die Delegation ärztlicher Leistungen an nichtärztliches Personal in der ambulanten vertragsärztlichen Versorgung gemäß § 28 Abs. 1 S. 3 SGB V, die Handbücher und Manuale der Gerätehersteller, die Nationalen Versorgungs-Leitlinien, die Experten-Konsensus-Artikel, im Rahmen von Studien die Voten der zuständigen Ethikkommissionen inkl. Einhaltung Guter Klinischer Praxis (GCP), die Empfehlungen entsprechender Fachgesellschaften, der Operationen- und Prozedurenschlüssel (OPS) sowie die Gebührenordnung für Ärzte (GOÄ) vor.

Neben diesen rechtlichen Rahmenbedingungen sind im klinischen Alltag folgende Qualitätssicherungsmaßnahmen zu empfehlen:

- regelmäßige Schulungen des Personals (mind. einmal im Jahr)
- Standardisierung der Prozeduren
- Notfallplan zum Umgang mit Komplikationen wie einer Synkope oder einem konvulsiven Anfall
- Fallbesprechungen
- standardisierte Regeln zur Dokumentation und Abrechnung
- Einhaltung der Maßnahmen zur Schweigepflicht und zum Datenschutz

3. Indikationen und Behandlungsprotokolle

Es gibt verschiedene Möglichkeiten, die Evidenz der einzelnen rTMS-Protokolle bei verschiedenen Erkrankungen zu beurteilen. Lefaucheur und Kollegen verfassten einen umfassenden Überblick über die Evidenz der rTMS bei psychiatrischen und neurologischen Indikationen, bei dem internationale/europäische Experten der entsprechenden Indikationen auf Basis bestimmter Qualitätslevel die Studienlage bewerteten (Lefaucheur et al., 2014, 2020). Folgende Evidenzklassen wurden definiert:

a) *„Definitiv wirksame Protokolle"* sind entsprechend definiert über das Vorhandensein von mindestens zwei Studien entsprechend Klasse I (verblindete randomisierte placebo-kontrollierte klinische Studie; n≥25) oder von mindestens einer nach Klasse I und zwei nach Klasse II (entsprechend Klasse I mit n<25).
b) *„Wahrscheinlich wirksame Protokolle"* = zwei überzeugende Studien der Klasse II oder eine Studie der Klasse II und zwei der Klasse III (andere kontrollierte Studien).
c) *„Möglich wirksame Protokolle"* = eine Studie der Klasse II oder zwei der Klasse III.

Im Rahmen der evidenz-basierten Medizin sind Meta-Analysen randomisierter placebo-kontrollierter Studien die höchste Stufe der Qualität. Die deutschen Versorgungs-Leitlinien orientieren sich an dem Vorhandensein entsprechender Meta-Analysen, berücksichtigen aber auch neben der Evidenz ethische Aspekte, klinische Relevanz und Umsetzbarkeit. „Soll" entspricht dabei einer starken positiven, „sollte" einer abgeschwächten positiven, und „kann" einer offenen Empfehlung. In der Tabelle werden ausschließlich S3-Leitlinien (https://www.awmf.org/leitlinien) der entsprechenden Erkrankungen (Unipolare/ bipolare Depression, Schizophrenie, Chronischer Tinnitus, Zwangsstörungen, Abhängigkeitserkrankungen, Posttraumatische Belastungsstörung) aufgelistet. Daneben gibt es Zulassungen für die Anwendung von bestimmten Geräten bei definierten Krankheiten, im amerikanischen Raum durch die Food and Drug Administration (FDA) oder im europäischen Raum durch die Conformité Européenne (CE-Zertifizierung), welche auch nach bestimmten Kriterien (u.a. Sicherheit und Leistungsfähigkeit) erfolgen. Die Zulassungen finden sich in den entsprechenden Dokumenten der Gerätehersteller. Die

folgende Tabelle gibt einen Überblick über die Evidenzlage der rTMS bei psychiatrischen Erkrankungen. Nicht erwähnte Erkrankungen fehlen deshalb, weil derzeit noch keine Aussagen auf Grund bislang zu weniger vorliegender Studien zu tätigen sind, was also kein Beweis für eine fehlende Wirksamkeit bei diesen Erkrankungen ist.

Die rTMS wird bei verschiedenen Erkrankungen angewandt - am häufigsten und mit der besten und einer klaren Evidenz bei **Depressionen**. Die Depression wird im deutschsprachigen Raum meist mit einem hoch-frequenten Protokoll mit einer Schmetterlingsspule über dem linken dorsolateralen präfrontalen Kortex (DLPFC) behandelt. Ein deutscher Konsensus-Artikel entsprechender Fachgesellschaften empfiehlt folgende Parameter (Hebel et al., 2022): eine **Frequenz** von 10Hz oder 20Hz, eine **Stimulationsintensität** von 100-120% relativ zur individuell gemessenen Ruhemotorschwelle (RMT), eine Pulszahl pro **Einzel-Sitzung** von 1500-3000 Pulsen pro Sitzung und eine **Behandlungsdauer** von 15-30 Sitzungen (entsprechend 3-6 Wochen Therapie). Dies fasst Empfehlungen zur Anwendung der rTMS als second line und third line Therapieansatz entsprechend der nationalen Versorgungsleitlinie (NVL) zur Behandlung zusammen. Zudem werden die gleichen Protokolle zur Behandlung der therapieresistenten Depression (TRD) – nach neuerer Evidenz in Kombination mit Psychotherapie – empfohlen. Der Wortlaut der S3-Leitlinie der Behandlung mit TMS lautet:

- 7-16 (neu): „Bei Patient*innen, die nicht auf eine Monotherapie mit Antidepressiva ansprechen, kann eine Augmentation mit repetitiver transkranieller Magnetstimulation (rTMS) angeboten werden."
- 7-29 (modifiziert): „Eine repetitive transkranielle Magnetstimulation (rTMS) sollte bei therapieresistenten depressiven Episoden angeboten werden."
- 7-30 (neu): „Die Auswahl der rTMS-Methode (Stimulationsort und -art) soll durch ein spezialisiertes Zentrum erfolgen."

In der Europäischen Leitlinie (Lefaucheur et al., 2020; Lefaucheur et al., 2014) wird zudem davon ausgegangen, dass eine rechtsfrontale niedrig-frequente oder eine bi-frontale Stimulation (auch mit Theta-Burst-Protokollen) wahrscheinlich wirksam ist. Diese Protokolle sind möglicherweise gleich effektiv wie die Standardbehandlung. Eine rechtsfrontale Stimulation mit 1Hz ist v.a. bei Unverträglichkeit oder relativen neurologischen Kontraindikationen in Erwägung zu ziehen. Die Behandlungsprotokolle sind

recht unterschiedlich (120-1600 Pulse pro Sitzung, 10-20 Sitzungen). Was auffällt, ist die vergleichsweise geringe Pulsanzahl und Dauer bei üblicher Stimulationsintensität.
Neben der unipolaren Depression „kann“ (S3-Leitlinie zur Diagnostik und Therapie Bipolarer Störungen) das genannte Behandlungsschema auch zur Behandlung einer depressiven Episode im Rahmen einer bipolaren Störung (insbesondere von Bipolar-II-Störungen) verwendet werden, wobei das individuelle Switch-Risiko erwogen werden sollte. Dabei ist insgesamt eine geringere Evidenzlage aufgrund weniger Publikationen für die bipolare Depression vorliegend. Die Effekte scheinen altersunabhängig zu sein.

Exkurs: Theta-Burst-Stimulation (TBS)

Im Gegensatz zur klassischen TMS basiert TBS auf kurzen, hochfrequenten Impulsserien, die in einem bestimmten rhythmischen Muster verabreicht werden und dabei die natürliche Theta-Frequenz des Gehirns (~5 Hz) nachahmen (Huang et al. 2005). Die Stimulation erfolgt in sogenannten Bursts (Pulspaketen), die jeweils aus drei TMS-Pulsen mit einer Frequenz von 50 Hz bestehen. Diese Bursts werden dann mit einer Theta-Frequenz von 5 Hz wiederholt.
Als Hauptformen von TBS werden die intermittierende TBS (iTBS) und die kontinuierliche TBS (cTBS) unterschieden. Bei der iTBS werden Serien von Bursts für typischerweise 2 Sekunden (sog. on-Phase) verabreicht und nachfolgend von kurzen Pausen von typischerweise 8 Sekunden (sog. off. Phase) unterbrochen. Durch die kurzen Pausen sollen exzitatorische neuroplastische Effekte gefördert werden. Bei der cTBS werden alle Bursts in einer durchgehenden Serie ohne Pausen über einen bestimmten Zeitraum hinweg (typischerweise 40 Sekunden) verabreicht. Die fehlenden Pausen führen in Modellversuchen zu hemmenden (inhibitorischen) Wirkungen auf die neuronale Aktivität des stimulierten Hirnareals, weshalb die cTBS beispielsweise zur Dämpfung überaktiver neuronaler Netzwerke bei persistierenden akustischen Halluzinationen zum Einsatz kommt. Etwas komplexer ist der Einsatz am rechten DLPFC zu betrachten. Hier soll durch die hemmende Wirkung ein antidepressiver Effekt induziert werden, der auf der sog. Lateralitätshypothese der Depression fußt.
Als Vorteile der TBS gegenüber der klassischen TMS werden häufig kürzere Behandlungszeiten angeführt, da TBS meist in nur wenigen Minuten durchgeführt werden kann, während klassische TMS-Sitzungen zwischen 17 und 40 Minuten dauern. Neuere

Studien werfen zudem die Frage auf, ob aus der kürzeren Behandlungsdauer auch resultieren könnte, dass TBS von PatientInnen besser toleriert wird als die klassische TMS Therapie. Dies könnte auch mit der verwendeten Stimulationsintensität in Zusammenhang stehen, die typischerweise bei nur 80% der Ruhemotorschwelle liegt.

Durch die erfolgreiche Evaluation der TBS bei der Depressionsbehandlung durch den Nachweis zumindest gleichwertiger antidepressiver Effekte im Vergleich zur konventionellen rTMS (Blumberger et al. 2018) hat diese Stimulationsart sich international in der klinischen Anwendung zunehmender Beliebtheit erfreut. In der Nicht-Unterlegenheits-Studie konnte gezeigt werden, dass eine mit 120% RMT-Intensität applizierte iTBS einer konventionellen 10 Hz rTMS nicht unterlegen war und zu ebenso starkem Rückgang depressiver Beschwerden bei den behandelten PatientInnen führte (Blumberger et al., 2018).

Durch die Anwendung von intermittierender Theta-Burst-Stimulation (iTBS) ist bei Applikation des 600 Pulse umfassenden Standardprotokolls eine Verkürzung der täglichen Behandlungsdauer (um den Faktor 6-7) für die jeweiligen Behandlungssitzungen möglich. Die iTBS erfolgt im Vergleich zu konventioneller rTMS typischerweise mit etwas niedrigerer Intensität bei ebenso langer Behandlungsdauer (Tage) (Kishi et al., 2024). Strittig ist nach wie vor die pro Behandlungssitzung zu applizierende Anzahl an Pulsen (600, 1200, 1800). Populärer wurde die iTBS in Zeiten der pandemie-bedingten Kontaktbeschränkungen durch die enorme Zeitersparnis (Anwendungsdauer ca. 3.5 Minuten im Vergleich zu ca. 20 Minuten pro Sitzung im Fall konventioneller rTMS), in Zeiten ökonomischen Drucks im klinischen Bereich ist sie aber von bleibend großem Interesse.

Exkurs: Beschleunigung der Behandlung

Eine in den vergangenen Jahren zumindest im wissenschaftlichen Bereich stark diskutierte Anpassung antidepressiver TMS-Protokolle bezieht sich auf die Beschleunigung der Behandlung durch eine Massierung von rTMS/TBS-Behandlungssitzungen innerhalb eines kurzen Zeitraums. Hierbei wird mit der Durchführung mehrerer Behandlungssitzungen pro Tag (akzelerierte TMS, aTMS) die Idee einer schnelleren antidepressiven Response und einer Verkürzung der Gesamtbehandlungsdauer verfolgt.

Die **Anwendung der aTMS** hat durch die Publikation des sogenannten und in ersten Studien sehr wirksamen SAINT-/SNT-Protokolls (Cole et al., 2022; Cole et al., 2020) deutlich

zugenommen und wissenschaftliches Interesse auf sich gezogen. Allerdings ist unklar, welche Spezifika des SAINT-/SNT-Protokolls entscheidend sind: die Verwendung einer Neuronavigation (basierend auf Gegenkorrelation des Stimulationsortes mit dem subgenualen anterioren Zingulum), die hohe Anzahl von 10 Sitzungen pro Tag oder die Anzahl von 1800 Pulsen in Form prolongierter iTBS pro Behandlungssitzung (Gesamtanzahl applizierter Pulse pro Person über fünf Tage hinweg: 90000). Die Umsetzung des SAINT-/SNT-Protokolls als akzelerierte iTBS-Behandlung (aiTBS) im klinischen Alltag erscheint auf Grund des hohen Aufwands im Moment kaum außerhalb hochspezialisierter Zentren realisierbar. Eine aktuelle Meta-Analyse (Cai et al., 2023) fasst jedoch die Ergebnisse von aTMS-Protokollen über die SAINT-Ergebnisse hinausgehend als positiv hinsichtlich der antidepressiven Wirksamkeit zusammen. Bedenken Sie für den Einsatz aTMS, dass flexiblere Protokolle als das SAINT-/SNT-Protokoll denkbar sind und entsprechend ein Protokoll mit weniger Sitzungen pro Tag (z.B. 4-5 Sitzungen) einfacher zu implementieren ist. Beispielsweise konnten Duprat et al. (2016) bei einer nur vier Tage dauernden aTMS-Behandlung mit fünf iTBS-Sitzungen pro Tag (pro Sitzung 1620 Pulse über dem linken dlPFC) und einem Intersession-Intervall von 15 Minuten einen positiven therapeutischen Effekt nachweisen, der teilweise auch erst zeitlich verzögert in den zwei Wochen nach Behandlungsende zur Gänze auftrat. Wichtig bei der Planung ist die Einhaltung eines Intersession-Intervalls zwischen den Behandlungsprotokollen innerhalb eines Tages: Hierbei sollte die zeitliche Pause zwischen Sitzungen mindestens 15 Minuten, idealerweise jedoch ca. 50 Minuten betragen (siehe auch Cai et al., 2023). Wird dies nicht beachtet, kommt es unter Umständen nicht zur intendierten Häufung neuroplastischer TMS-Effekte, und das Ergebnis mehrerer Sitzungen am Tag unterscheidet sich potenziell nicht von dem einer einzelnen Sitzung. Aufgrund potenziell nachgelagerter therapeutischer Effekte sollte bei der Aufklärung zur aTMS auf diese Möglichkeit hingewiesen werden und es sollten idealerweise auch nachgelagerte Termine zur Zustandsbeurteilung (z.B. 2-4 Wochen nach Behandlungsende) vereinbart werden.

Jede Abweichung von den Standardprotokollen muss gut begründet werden. Es muss zudem berücksichtigt werden, dass weniger Daten zur Verträglichkeit vorliegen. Eine adäquate Abwägung von Nutzen und Risiken erfordert umfassende Erfahrung mit der TMS und ein besonders sorgfältiges Sicherheitsmanagement. Insgesamt ist zur Wirksamkeit und Sicherheit der schnelleren Protokolle noch Forschung nötig.

Exkurs: Erhaltungs-rTMS

Zur Rückfallprophylaxe in der Depressionstherapie und zur Erhaltungstherapie mit rTMS bei PatientInnen mit positiver initialer Response gibt es kaum kontrollierte Studien (Baeken et al., 2019; Chang et al., 2020; Haesebaert et al., 2018; Wilson et al., 2022). **Auf Basis klinischer Erfahrung hat sich gezeigt,** dass das positive Ansprechen auf eine rTMS Behandlung das künftige Ansprechen auf eine erneute Behandlung prädiziert. Somit lässt sich als grundsätzliche Empfehlung festhalten, dass PatientInnen, die auf rTMS angesprochen haben, empfohlen werden kann, sich bei einer erneuten depressiven Episode wieder einer rTMS Behandlung zu unterziehen.

Bezüglich einer fortgesetzten Erhaltungsbehandlung mit rTMS nach Abschluss einer effektiven antidepressiven rTMS-Behandlung ist die vorliegende Evidenz auf Basis vorwiegend offener Studien zwar schwach, jedoch überwiegend positiv. Die Befunde weisen klar darauf hin, dass die Erhaltungsbehandlung mit rTMS sicher und effektiv ist. Allerdings gibt es bislang keinen Standard bzw. kein Protokoll-Schema, das besonders häufig untersucht und publiziert wurde. Eine aktuelle systematische Übersichtsarbeit zum Thema von d'Andrea et al. (2023) konnte zeigen, dass mit einer zu geringen Anwendung von rTMS-Sitzungen (2 oder weniger pro Monat) kein nennenswerter Erhaltungseffekt erzielt werden konnte. Zudem lassen die vorliegenden Studien den vorsichtigen Schluss zu, dass ein zeitlicher Abstand von vier Wochen zwischen Abschluss der Akutbehandlung und Beginn der Erhaltungsbehandlung mit rTMS liegen kann, ohne damit das Rückfallrisiko zu erhöhen (d'Andrea et al., 2023). Dies sollte in Anbetracht der zur Verfügung stehenden Ressourcen also ebenfalls in die Überlegungen über ein passendes Erhaltungs-TMS-Protokoll einbezogen werden.

Letztlich sind aus klinischer Sicht bei PatientInnen mit rezidivierenden depressiven Episoden mehrere Varianten der Erhaltungsbehandlung mit rTMS denkbar:

- Variante (a): Allmähliches Ausschleichen der rTMS-Sitzungen über die Wochen hinweg. Es wird also die Zahl der wöchentlichen Sitzungen im Laufe der Zeit einem Reduktionsschema folgend verringert. Gestartet wird dabei üblicherweise mit 1 oder 2 Sitzungen pro Woche. Diese Form der Erhaltungsbehandlung beginnt ggf. direkt im Anschluss an die Akutbehandlungsphase.

- Variante (b): Anwendung von Booster-Behandlungswochen (also eine Woche mit täglichen rTMS-Behandlungen) mit einem Abstand von mehreren Wochen (zunächst beginnend mit einem Intervall von 3-6 Wochen), ggf. mit allmählicher Ausweitung behandlungsfreier Intervalle.

Es wird empfohlen, im Einzelfall mit betreffenden PatientInnen die rezidivierende Symptomatik ihrer Depression zu besprechen und die Aufrechterhaltungs-rTMS entsprechend einzelfallbezogen zu planen und anzupassen.

Exkurs: rTMS-Indikationen jenseits der Depression

Die **Negativsymptomatik** der Schizophrenie wird vergleichbar zur Depression behandelt. Die Evidenz ist aber geringer, so dass nur von „kann“ und „möglich wirksam“ gesprochen wird. Dies deckt sich mit den in den veröffentlichten Meta-Analysen berichteten Befunden, die auf einer relativ geringen Anzahl an Studien basieren. Zudem ist die Anzahl an verfügbaren Meta-Analysen geringer, sie sind teilweise relativ alt und befassen sich nicht ausschließlich mit der rTMS bei Negativsymptomatik (Aleman et al., 2018; Dougall et al., 2015; Hyde et al., 2022; Osoegawa et al., 2018; Tseng et al., 2022). Die S3-Leitlinie betont, dass PatientInnen über die zu erwartende geringe Response-Rate zu informieren sind.

Etwas positiver stellt sich die Einschätzung der Wirksamkeit der niedrig-frequenten rTMS der therapie-resistenten **akustischen Halluzinationen** bei Schizophrenie im Rahmen der konsensus-basierten Richtlinien dar, wenngleich hier auch negative Meta-Analysen vorgelegt wurden (He et al., 2017). Nach aktueller S3-Leitlinie „sollte“ eine rTMS-Behandlung bei Vorliegen therapieresistenter akustischer Halluzinationen im Rahmen einer schizophrenen Psychose durchgeführt werden, da trotz der Heterogenität vorliegender Befunde ein zumindest geringfügiger positiver Therapieeffekt als wahrscheinlich anzunehmen ist. Zusammengefasst empfiehlt sich bei Therapie-Resistenz ein Behandlungsversuch mit niederfrequenter (hemmender) 1Hz über dem linken temporoparietalen Übergangskortex (TPJ, Stimulationsintensität: 80-100% RMT, 10 Sessions, 1000 oder 1200 Pulse).

Bei **chronischem Tinnitus** wird durch die aktuelle S3-Leitlinie eine Behandlung mit rTMS explizit nicht empfohlen, auch wenn eine Reihe positiver Meta-Analysen publiziert wurde und die europäische Leitlinie eine Behandlung mit rTMS als möglich wirksam erachtet

(Lefaucheur et al., 2020; Lefaucheur et al., 2014). Die Behandlung ist analog zu der von akustischen Halluzinationen.

Bei **Zwangsstörungen** kann bei PatientInnen im Falle eines unzureichenden Ansprechens auf Verfahren der ersten Wahl laut S3-Leitlinie eine rTMS-Behandlung durchgeführt werden (Empfehlungsgrad 0). Es gibt hierbei eine Reihe verschiedener Stimulationsprotokolle (Fitzsimmons et al., 2022), die über verschiedenen Regionen zum Einsatz kommen (dorsolateraler präfrontaler Kortex, supplementär-motorisches Areal, medialer präfrontaler Kortex/ anteriorer cingulärer Kortex, orbitofrontaler Kortex). Trotz des Vorliegens von Zulassungen (z. B. FDA-zugelassene tiefe rTMS mit H1-Spule, NeuroStar-System) und Leitlinienempfehlungen bleibt die Evidenz heterogen. Insbesondere fehlen systematische Untersuchungen zur Differenzierung zwischen verschiedenen syndromalen Subtypen (z. B. primär Zwangsgedanken vs. Zwangshandlungen), sodass aktuell keine eindeutige Präferenz für ein spezifisches Protokoll ausgesprochen werden kann. Eine neuere Entwicklung stellt die Anwendung einer tiefen rTMS dar, die durch spezielle Spulengeometrien eine höhere Eindringtiefe erreicht (anvisiertes Ziel: medialer PFC, anteriorer cingulärer Kortex). Die zur FDA-Zulassung führenden Studien haben hierbei die typischerweise auf sechs Wochen angelegte tiefe rTMS-Behandlung mit einer verhaltenstherapeutischen Expositionsintervention kombiniert, was in der Planung und Ausgestaltung dieser Behandlungsform zu berücksichtigen ist.

Für **Abhängigkeitserkrankungen und Craving** ist die Evidenz sehr heterogen. Leitlinien und Zulassungen stimmen kaum überein. Empfohlen wird – auch auf Basis neurobiologischer Erkenntnisse – eine rTMS-Behandlung über dem linken dlPFC (hochfrequentes Stimulationsprotokoll) mit dem Ziel der Stärkung kognitiver Kontrollnetzwerke und der Abschwächung von Craving (Gay et al., 2022; Zhang et al., 2019). Die Wahl des Stimulationsprotokolls könnte hierbei von der spezifischen Substanzabhängigkeit und dem Behandlungsziel abhängig gemacht werden und es sollte angesichts des diesbezüglich bestehenden Forschungsbedarfs und fortlaufender Studien in diesem Bereich vor Festlegung eine aktuelle Literaturrecherche durchgeführt werden.

Für die **Posttraumatische Belastungsstörung** ist die hochfrequente Behandlung des rechten DLPFC wahrscheinlich wirksam, auch wenn für verschieden TMS-Behandlungsformen bislang nur kurzzeitige Behandlungseffekte aufgezeigt werden

konnten (Liu et al., 2024). Zu beachten ist allerdings, dass die TMS in den Leitlinien keine Erwähnung findet und keine Zulassungen existieren.

Die Tabelle auf der nachfolgenden Seite fasst nochmals die derzeitige Evidenz und die gängigsten rTMS-Protokolle für die Behandlung der besprochenen psychischen Störungen zusammen.

Tabelle: Psychiatrische Indikationen der rTMS, prominente Protokolle und Evidenzgrade

	Behandlungsprotokoll	Deutsche Leitlinien	Europäische Leitlinie[a]	Zulassungen	Studienlage
Uni-/ Bipolare Depression	F3, 10/20Hz, 100-120%, 1500-3000 Pulse, 20-30 Sitzungen (oder F3, iTBS, 80%, 600 Pulse, 20-30 Sitzungen) (Hebel et al., 2022)	Sollte-Empfehlung bei Therapie-Resistenz<br>Kann-Empfehlung nach erfolgloser Monotherapie und bei bipolarer Depression	definitiv wirksam	FDA, CE	zahlreiche positive Meta- Analysen vorhanden
Negativ-symptomatik bei Schizophrenie	Protokoll analog Depression (Lefaucheur et al., 2014; 2020)	Kann-Empfehlung bei Therapieresistenz im Rahmen eines Gesamt-behandlungsplan<br>Aufklärung über niedrige Ansprechrate	möglich wirksam	nein	weitere Evidenz notwendig
Akustische Halluzinationen bei Schizophrenie	CP5, 1Hz, 80-100%, 1000/1200 Pulse, 10 Sitzungen (oder CP5 **und** CP6, cTBS, 80%, je 600 Pulse, 15-20 Sitzungen) (Lefaucheur et al., 2014; 2020; Plewnia et al., 2018; Ye et al., 2024)	Sollte-Empfehlung bei Therapieresistenz im Rahmen eines Gesamt-behandlungsplans	möglich wirksam	nein	weitere Evidenz notwendig
Chronischer Tinnitus	CP5, 1Hz, 110%, 2000 Pulse, 10 Sitzungen (Folmer et al., 2015; Lefaucheur et al., 2014; 2020)	Sollte-Empfehlung **gegen** TMS (Sondervotum der DGPPN: *„kann erwogen werden“*)	möglich wirksam	nein	weitere Evidenz notwendig
Zwang	SMA, 1Hz, 100% (Bein), mind. 1200 Pulse, 15-30 Sitzungen, ggf. mit gewinkelter 8er-Spule<br>(oder F4, 1Hz, 110%, mind. 1200 Pulse, 15-30 Sitzungen) (Fitzsimmons et al., 2022)	Kann-Empfehlung bei Therapieresistenz zur kurzfristigen Symptomlinderung	möglich wirksam	FDA, CE	Inkonsistenz der Protokolle, weitere Evidenz notwendig
Abhängigkeit	Protokoll analog Depression (Lefaucheur et al., 2014; 2020)	keine Empfehlung möglich	möglich wirksam bei Nikotin	FDA, CE für Substanzen	weitere Evidenz notwendig
PTBS	F4, 1 vs. 20Hz, 80-120%, 100-4000 Pulse, 10-30 Sitzungen (McGirr et al., 2021)	TMS ist nicht erwähnt	wahrsch. wirksam	nein	weitere Evidenz notwendig

Anmerkungen: F3-entspricht dem linken dlPFC (10-20 EEG-Koordinate), F4-entspricht dem rechten dlPFC (10-20 EEG-Koordinate), CP5/CP6-entspricht dem linken/ rechten temporoparietalen Übergang (TPJ, 10-20 EEG-Koordinate), SMA-supplementär-motorisches Areal; [a] bezieht sich auf Lefaucheur et al. (2014; 2020)

4. Aufklärung, Kontraindikationen, Nebenwirkungen und besondere Personengruppen

4.1. Inhalte der Aufklärung

Folgende Punkte sind für die Aufklärung von Relevanz (kein Anspruch auf Vollständigkeit)

- mündliche Aufklärung durch ärztliches Personal
- Dokumentation der Aufklärung
- Bedenkzeit einplanen (abhängig von Komplexität des Falls)
- Information nicht nur über Erfolgsaussichten, Ablauf, Risiken und Nebenwirkungen, sondern auch über mögliche Alternativen
- Indikationsstellung durch fachärztliches Personal (Psychiatrie, Nervenheilkunde mit entsprechender Qualifikation im Bereich der Gehirnstimulationsverfahren (entsprechend der Weiterbildungsordnung für Ärzte oder der Vorschläge der Fachgesellschaften DGPPN und/oder DGHP)
- im Falle fehlender Zulassung Dokumentation der Off-Label-Aufklärung
- bei Depressionen mit komorbiden weiteren Störungen sollte die Depression als Indikation betont werden und die Behandlung mit einem Depressionsprotokoll erfolgen

Es wird empfohlen einen Aufklärungsbogen zu verwenden. Einen Textvorschlag für den Aufklärungsbogen finden Sie im Anhang A.

Als Formulierungshilfe für die Dokumentation in der Krankenakte kann folgende Textpassage dienen:

„***Es erfolgte die Vorstellung zur rTMS-Behandlung bei einer vorbeschriebenen Depression und unzureichender Besserung unter der bisherigen multimodalen Therapie. Die/Der Patient/in wurde mündlich und schriftlich über Indikation, Wirkung und Nebenwirkungen aufgeklärt. Kontraindikationen liegen keine vor. Ziel der Behandlung ist die Besserung der depressiven Symptomatik. … Bedenkzeit*** …“

4.2. Kontraindikationen und besondere Personengruppen

Die Indikationsstellung zur Behandlung einer Depression bei vorhandenen Komorbiditäten (beispielsweise epileptischen Anfällen in der Vorgeschichte) oder bei Vorliegen besonderer klinischer Umstände (beispielsweise einer Schwangerschaft) sollte auf Einzelfallniveau unter individueller Nutzen- und Risikoabwägung erfolgen und erfordert eine besonders sorgfältige Aufklärung durch den Arzt. Das erhöhte relative Risiko hinsichtlich eines epileptischen Anfalls sollte beim Vorliegen neurologischer Erkrankungen gewürdigt werden. Die meisten Kontraindikationen sind relativ. Bezogen auf die Kontraindikationen ist über die hier präsentierten Ausführungen auch auf die internationalen Experten-Empfehlungen insbesondere der International Federation of Clinical Neurophysiology (IFCN) zu verweisen (Rossi et al., 2009, Rossi et al., 2021).

Implantate, Tätowierungen, Piercings

Ferromagnetische oder für ihre Aktivität auf elektrische Pulsdetektion und -generation angewiesene Geräte (klassisches Beispiel: Herzschrittmacher) können Kontraindikationen für eine rTMS sein. Die MRT-Tauglichkeit eines Implantats kann Hinweise auf die rTMS-„Tauglichkeit" geben, stellt jedoch keine Garantie dar. Es kann daher nur eine individuelle Risikoabschätzung erfolgen unter Berücksichtigung u.a. der Gerätespezifika (Material, Wirkweise), des Abstandes zur rTMS-Applikation und der Spulengeometrie. Bei einer Risiko-Nutzen-Abwägung ist natürlich auch die Dringlichkeit der Indikation zu berücksichtigen (nur sehr selten ist eine rTMS von hoher oder gar vitaler Dringlichkeit, wie etwa die EKT). Metallhaltige Tätowierungen im Stimulationsbereich stellen auch eine Kontraindikation dar - im Gegensatz zu Zahnimplantaten oder Piercings. Bei letzteren ist materialabhängig ein Ablegen vor der rTMS-Behandlung zu empfehlen.

Zerebrale Vorschädigung und neurologische Vorerkrankungen

Bei „zerebraler Vorschädigung" handelt es sich um einen groben Überbegriff, der alleine keine absolute Kontraindikation für sich darstellt. Zu berücksichtigen ist, ob die individuelle Schädigung in ihrem Ausmaß und ihrer Lokalisation epileptische Anfälle begünstigen kann und/oder eine akkurate Zielidentifikation erschwert (etwa ausgeprägte Atrophie, linksfrontale Prozesse). In derartigen Fällen sollte eine individuelle Nutzen-Risiko-Abwägung unter Berücksichtigung der Therapiealternativen erfolgen und das

Komplikationsrisiko durch entsprechende diagnostische Maßnahmen (z.B. EEG) weiter spezifiziert werden. Die Aufklärung und die Einwilligung sollten in diesen Fällen besonders sorgfältig in der Krankenakte dokumentiert sein.

Ebenso sollte bei bekannten neurologischen Vorerkrankungen geprüft werden, ob die vorliegende wissenschaftliche Evidenz den Einsatz von rTMS-Protokollen z.B. in der Depressionsbehandlung stützt. Es liegen diesbezüglich zwar keine umfassenden Befunde vor, jedoch könnte die Wirksamkeit von rTMS-Protokollen z.B. zur Depressionsbehandlung bei Vorliegen bestimmter neurologischer Erkrankungen wie Morbus Parkinson reduziert sein (Liu et al., 2014; Zhang et al., 2022).

Schwangerschaft

Hierzu existiert mittlerweile robuste wissenschaftliche Evidenz, die mit den entsprechenden Patientinnen diskutiert und zur Grundlage einer gemeinsamen Entscheidungsfindung genutzt werden kann (Hebel, Schecklmann, & Langguth, 2020; Hızlı Sayar et al., 2014; D. R. Kim et al., 2019). Mindestens zwei prospektive Studien und eine Vielzahl von Fallberichten zeigen, dass die rTMS grundsätzlich in der Schwangerschaft ohne Schaden für Mutter und Kind angewendet werden kann. Die insgesamt geringe Fallzahl erlaubt es aber nicht, seltenere Nebenwirkungen verlässlich auszuschließen. In dieser Patientinnengruppe sollte besonderes Augenmerk auf Risiko-Nutzen-Abwägung und Präferenz der Behandelten liegen. Die Aufklärung und die Einwilligung sollten in diesen Fällen besonders sorgfältig in der Krankenakte dokumentiert sein. Ebenso sollte in diesem Zusammenhang im Rahmen der Aufklärung explizit auf eine mögliche Schädigung des Kindes bei einem TMS-induzierten epileptischen Anfall hingewiesen werden.

Ko-Medikation

Aufgrund der Vielzahl von möglichen Substanzen und Wechselwirkungen kann dieses Thema hier nicht erschöpfend abgehandelt werden. Für eine Übersicht sei auf die Arbeit von Hebel, Abdelnaim, et al. (2020) und Deppe et al. (2021). Hauptsächlich diskutiert werden abschwächende Effekte auf den Behandlungserfolg durch Benzodiazepine, Antipsychotika und Antikonvulsiva. Demgegenüber werden förderliche Effekte bei Ko-Medikation mit einem SSRI (Zaidi et al., 2024) oder mit Psychostimulanzien (Hunter et al., 2019) berichtet.

4.3. Nebenwirkungen der rTMS-Behandlung

- **Lokale Reaktionen**

Unangenehme Sensationen an der Stimulationsstelle sowie milde bis moderate Kopfschmerzen sind die häufigsten Nebenwirkungen und treten vorranging zu Behandlungsbeginn auf. Behandlungsabbrüche aufgrund dessen sind der klinischen Erfahrung nach selten, kommen aber vor. Die Toleranz gegenüber und der Umgang mit auftretenden Kopfschmerzen variiert im klinischen Alltag enorm zwischen PatientInnen entsprechend ihrer individuellen Prädisposition und psychischen Gesamtverfassung.

- **Hörschäden und elektromagnetische Feldexposition**

Jede Patientin und jeder Patient sind grundsätzlich dem lauten Klick-Geräusch und dem elektromagnetischen Feld des TMS-Pulses ausgesetzt. Zu diesen zwei grundsätzlichen Expositionen der TMS existieren bislang wenige wissenschaftliche Daten (C. Schönfeldt-Lecuona et al., 2012).

Hinweise auf Hörschäden aus einzelnen Studien und grundlegende Risikoabwägungen legen nahe, dass PatientInnen ein Gehörschutz (Ohrstöpsel) angeraten werden sollte. Zur Relevanz der Langzeit-Exposition gegenüber den durch die rTMS generierten elektromagnetischen Feldern fehlen belastbare Langzeituntersuchungen.

- **Switch-Risiko, Suizidrisiko**

Ein Switch in eine manische Phase (auch falls noch keine bipolare Störung bekannt ist) sowie das Auftreten oder die Verstärkung von Suizidgedanken kann grundsätzlich bei jeder Patientin bzw. jedem Patienten mit Depression bzw. unter Therapie bzw. bei erfolgloser Therapie auftreten. Die Indikationsstellung durch eine Fachärztin oder einen Facharzt für Psychiatrie und ein Abschlussgespräch durch eine qualifizierte Person ist auch aus diesem Grund von Bedeutung. Es handelt sich aber um keine rTMS-spezifischen Risiken.

- **Kognitive Nebenwirkungen**

Erfahrungsgemäß fragen manche PatientInnen nach unerwünschten Wirkungen auf kognitive Fähigkeiten. Es existieren keine Hinweise auf solche Nebenwirkungen (Patel et al., 2020; Rossi et al., 2009). Zu beachten ist, dass „Kognition“ ein weiter Begriff ist und

kognitive Fähigkeiten in der Fremd-, aber auch vor allem Eigenwahrnehmung deutlich durch die eigentlich zu behandelnde Erkrankung, meist die Depression, beeinflusst werden.

- **Epileptischer Anfall und Synkope**

Die Literatur zu dieser Thematik zeigt, dass die Provokation eines epileptischen Anfalles durch die rTMS möglich, aber sehr unwahrscheinlich ist (< 2 von 100.000 Behandlungen; Shafi, 2019). Behandlungsprotokoll, individuelle Anfallsbereitschaft und das Vorhandensein weiterer Provokationsfaktoren spielen ebenso eine Rolle wie die Einnahme anti- und prokonvulsiver Medikamente. Grundsätzlich sollte das anwesende Personal Kenntnisse im Erkennen und der Erstbehandlung eines epileptischen Anfalles haben (z.B. sofortige Beendigung der Behandlung). Bei der Aufklärung ist nicht nur auf dieses mögliche Ereignis hinzuweisen, sondern auch auf mögliche Folgen.

Häufiger treten (prä-)synkopale Ereignisse unter der rTMS auf, insbesondere bei der Erstanwendung im Rahmen der Motorschwelle, bei morgendlichen Behandlungen und bei Protokollen mit langem Verharren in liegender oder zurückgelehnter Position und anschließendem raschen Aufstehen.

Exkurs: Risiken für Anwenderinnen und Anwender

Der Vollständigkeit halber sei erwähnt, dass insbesondere die Punkte Implantate, Schwangerschaft und akustische und elektromagnetische Exposition prinzipiell auch für die Anwenderinnen und Anwender der rTMS zu bedenken sind. Hierzu existieren jedoch so gut wie keine belastbaren Daten. rTMS-spezifischer Risiken sind grundsätzlich bei Gefährdungsbeurteilungen des Arbeitsplatzes zu berücksichtigen. Aktuelle Empfehlungen lauten auf einen Abstand von 70cm (ICNIRP; Rossi et al., 2009) bzw. 24cm (ICNIRP; Rutherford et al., 2020) bzw. 40cm (Rossi et al., 2021) für die Anwenderinnen und Anwender.

5. Dokumentation und Vergütung

Seit der Aufnahme der rTMS in den PEPP-Entgeltkatalog im Jahr 2021 stehen bei (teil-)stationären Behandlungen bewertete spezifische Zusatzentgelte (ZP75) für die Grundleistung (OPS 8-632.0) oder Therapiesitzung (OPS 8-632.1) bei der rTMS zur Verfügung. Für das ambulante Setting bleibt die Vergütungssituation in Deutschland ein Flickenteppich. Bei der Dokumentation und Kostenvoranschlägen ist darauf zu achten, dass die Therapieresistenz und evtl. auch die Schwere der Depression und die Alternativlosigkeit der rTMS erwähnt wird.

- **Stationäres Setting: Zusatzentgelte**

Abgerechnet und dokumentiert werden die rTMS-Grundleistung (ZP75.01, OPS: 8-632.0, 124,17 Euro, Stand: 2024) und die rTMS-Therapiesitzung (ZP75.02, OPS: 8-632.1; 77,54 Euro, Stand: 2024). Zu beachten ist, dass die Ärztin bzw. der Arzt das Aufklärungsgespräch separat dokumentiert und abrechnet. Die abzurechnende Zeit muss unter 25 Minuten bleiben. Die rTMS-Grundleistung enthält die Aufklärung, Motorschwelle und die erste Behandlung und ist immer für den ersten Behandlungstag zu kodieren. Dokumentiert und kodiert wird an dem Tag, an dem die jeweilige Leistung stattgefunden hat. Die rTMS-Therapieleistung ist die einzelne Behandlungssitzung, die u.U. auch mehrmals am Tag erfolgen kann. Da das Zusatzentgelt neu ist, wird sich im Laufe der Zeit durch die MD-Prüfungen zeigen, auf welche Spezifika geachtet werden muss.

- **Privat versicherte und selbstzahlende Personen im ambulanten Setting**

Es empfiehlt sich, einen entsprechenden Kostenvoranschlag/Behandlungsvertrag einreichen zu lassen. Es wird empfohlen die Evidenzlage darzustellen und auf die Therapieresistenz bzw. das Nichtansprechen des Falls einzugehen. Es wird nach GOÄ abgerechnet. Es kommen v.a. die Analogziffern 839a Motorschwelle (eigentlich elektromyographische Untersuchung; 93.84 Euro) und 828 rTMS (eigentlich evozierte Potentiale; 81.11 Euro) zur Anwendung. Zusätzlich sind bei entsprechender Leistungserbringung und Dokumentation die Ziffern 801 (psychiatrische Untersuchung), 804 bzw. 806 (psychiatrische Behandlung), 860 (biographische Anamnese), 1 bzw. 3 (Beratung), 865 (Fallbesprechung) und 857 (psychologische Tests) möglich.

6. Spulenpositionierung und Spulentyp

Bei der Behandlung mit rTMS ist die Positionierung der Magnetspule über dem jeweiligen Zielareal sowie die Ausrichtung und Verkippung der Magnetspule von großer Bedeutung. Es ist sicherzustellen, dass a) der Mittelpunkt der Spule möglichst genau über dem Zielareal positioniert ist und b) die Spulenorientierung korrekt ist. Die Spule soll dem Kopf tangential anliegen und der Spulenmittelpunkt die Auflagefläche auf dem Kopf bilden. Wichtig ist zudem, auf eine entspannte oder halb-liegende Sitzhaltung der Patientin bzw. des Patienten zu achten und die korrekte Spulenpositionierung im Verlauf zu kontrollieren. Ebenso sollte darauf geachtet werden, bei der Positionierung der Spule keinen übermäßigen Druck auf den Kopf der PatientInnen auszuüben, um Schmerzen oder gar Verletzungen zu vermeiden.

Bestimmte Spulenformen sind räumlich weniger präzise bzw. stimulieren größere kortikale Areale im Gegensatz zu der standardmäßig verwendeten Schmetterlingsspule (engl. „figure-8-coil“). Dazu zählen die Rundspule oder Spulen mit größerer Tiefenstimulation wie z.B. die gewinkelte Schmetterlingsspule oder bestimmte Helmspulen. Die Handlungsempfehlungen in diesem Handbuch beziehen sich auf die lokale Stimulation mit Schmetterlingsspulen auf Grund der Gebräuchlichkeit im deutschen Sprachraum. Für die Benutzung von Helmspulen empfehlen wir das Studium der entsprechenden Handbücher der Hersteller.

Generell bestehen zwei Möglichkeiten, ein Zielareal für die rTMS festzulegen. Zum einen kann man sich an Hirnwindungen oder kortikalen Positionen orientieren (**Neuronavigation**). Bei dieser neuroanatomisch basierten Navigation sind vor der Behandlung MRT-Aufnahmen durchzuführen und nur mit Neuronavigation ausgerüstete TMS-Geräte können nachfolgend die Spulenpositionierung durchführen (Carlos Schönfeldt-Lecuona et al., 2005). Diese Methode ist die genaueste und erlaubt über die Behandlungssitzungen hinweg eine sehr reliable Spulenpositionierung, gleichzeitig ist sie aber auch kosten- und zeitaufwändig (C. Schönfeldt-Lecuona et al., 2010). Sie ist möglich, aber nicht nötig. Zu wenig wissenschaftliche Literatur hat sich mit der Frage der Notwendigkeit der Neuronavigation im klinisch-psychiatrischen Setting beschäftigt (Herwig, Padberg, et al., 2001; Herwig, Schönfeldt-Lecuona, et al., 2001). Die zweite und am weitverbreitetste Methode der Zielareal-Lokalisation basiert auf **oberflächen-**

bezogener Bestimmung der Spulenposition (Herwig et al., 2003). Oberflächen-basiert meint die Nutzung von markanten Punkten der Schädelanatomie und von EEG-Punkten. Dazu ist die Verwendung von Hauben hilfreich, die mit einem Marker leicht zu beschriften sind. Zudem sinnvoll ist der Einsatz von flexiblen Maßbändern oder Geodreiecken. Für jede Patientin bzw. jeden Patienten wird aus hygienischen Gründen eine neue Haube verwendet. Bei einer wiederholten Stimulation – z. B. im Rahmen einer Behandlung – ist es wichtig, dass die Kappe immer in gleicher Weise angebracht wird. Dazu sollte man sich an Schädelmarken orientieren. Zum einen kann die Kappennaht immer im gleichen Abstand zum Nasion angebracht werden. Zum anderen kann Überprüfung der EEG-Position Cz hilfreich sein. Hilfreich ist das Einzeichnen der Mittel-/Sagittallinie, die sich mit Blick von vorne mittig über der Nase befinden sollte. Auch Markierungen auf der Kappe vor dem linken und rechten Ohr können hilfreich sein. Im klinischen Alltag bietet sich an, relevante Informationen zur Kappenpositionierung (z.B. Abstand Nasion-Haubennaht) nicht nur auf dem Behandlungsprotokoll, sondern auch auf der Kappe zu vermerken.

Exkurs: EEG-Koordinaten

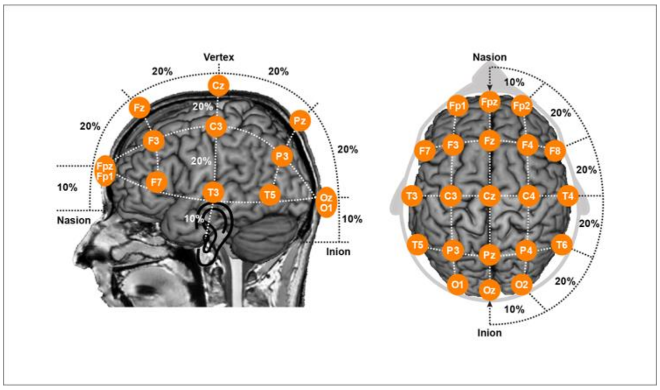

Das 10-20-Elektroden-Koordinatensystem erlaubt die standardisierte Erfassung von Kopfoberflächen-Positionen unabhängig von der Kopfgröße. Die Elektrodenpositionen erlauben eine grobe Zuordnung zu unter dem Schädel liegenden kortikalen Regionen (Koessler et al., 2009). Ausgehend von den festen anatomischen Referenzpunkten des Schädels (Nasion, Inion und präaurikulärer Punkt) werden halbmondförmige Linien über den Schädel gebildet, die wiederum in 10%- oder 20%-Abschnitte eingeteilt werden. Die Benennung der Elektroden-positionen erfolgt nach der Position auf dem Schädel (T=temporal, P=parietal, F=frontal, O=okzipital, C=zentral). Linksseitig gelegene Elektrodenpositionen sind ungerade nummeriert, rechtsseitig gelegene sind mit geraden Zahlen gekennzeichnet.

FPz ist der vordere mittlere Punkt im EEG-System, der als 1/10 der Entfernung Nasion-Inion definiert ist. Cz (Position über dem Vertex) stellt die Schnittstelle der interauralen und der

Mittellinie dar. Man kann sich hier zusätzlich auch von den PatientInnen mit dem Finger den höchsten Punkt des Kopfes zeigen lassen, der Cz entsprechen sollte. Der Punkt Oz liegt 10% über dem Inion. Das Inion ist der Knochenhöcker in der Mittellinie des Hinterkopfes am Ansatz der Nackenmuskeln. Das Nasion ist die Furche über der Nasenwurzel.

Bei längeren Behandlungssitzungen (Ausnahme: Bestimmung der Motorschwelle) sitzt oder liegt die Patientin oder der Patient auf einem der Behandlungsstühle mit abgelegtem Kopf. So können Kopfbewegungen und somit Veränderungen der Position des Kopfes zur Spule minimiert werden. Die PatientInnen sollen nach Möglichkeit Kopfbewegungen unterlassen. Sollte eine Positionsänderung der Patientin oder des Patienten nötig sein, wird sie oder er vorab gebeten, die BehandlerInnen zu informieren, um eine Unterbrechung der laufenden Stimulation und eine erneute Justierung der Spule an der korrekten Lokalisation zu gewährleisten. Die Minimierung unabsichtlicher Kopfbewegungen sowie eine möglichst entspannte Lagerung der/des Patientin/Patienten werden durch die Verwendung von (Vakuum-)Kissen zur Kopfpositionierung erreicht.

7. Relevante Spulenpositionen

Statt einzelne Markierungen zu vermessen, gibt es generell die Möglichkeit, EEG-Leerhauben mit vorgestanzten Markierungen zu benutzen, die bei EEG-Vertriebsfirmen bestellt werden können. Diese Leerhauben werden über die Behandlungskappe gezogen und ermöglichen die Markierungen verschiedener EEG-Punkte.

Eine weitere Empfehlung ist die zusätzliche Markierung des Spulenansatzes. Da viele TMS-Spulen den Blick auf den Stimulationspunkt nicht ermöglichen, hilft es, den Ansatz der Spule auf dem Kopf/ der Haube zu markieren. Auf dem Kopf/ der Haube wird der Abstand der Spulenmitte zum Spulenansatz eingezeichnet. Da häufig die gleichen Standardspulen im Einsatz sind, lohnt es sich den Abstand einmal auszumessen und zu notieren. Zu beachten ist, dass der Abstand Spulenmitte-Spulenansatz planar gemessen wird, der Kopf aber gewölbt ist.

Häufig wird in den Publikationen angegeben, in welche Richtung der Spulengriff zeigt. Diese Information ist alleine ohne die Information der Stromrichtung nicht ausreichend. Leider fehlen diese Angaben häufig. Wir empfehlen deshalb die Verwendung der Default-Einstellungen der Geräte hinsichtlich der Stromflussrichtung.

Die verschiedenen Erkrankungen werden an verschiedenen kortikalen Punkten behandelt. Aufgeführt sind die Spulenpositionierungen für die gängigsten evidenz-basierten Behandlungen. Zusätzlich wird zunächst die Spulenpositionierung über dem Motorkortex und das Vorgehen bei der Motorschwellenbestimmung beschrieben.

7.1. Motorkortex inkl. Motorschwellenbestimmung

Das im Folgenden beschriebene Vorgehen bezieht sich beispielhaft auf die Bestimmung der Motorschwelle für eine geplante antidepressive rTMS-Behandlung über dem linken dorsolateralen präfrontalen Kortex. Aus diesem Grund wird die Motorschwelle über eine Stimulation des linksseitigen motorischen Kortex (aufgrund der kontralateralen Verschaltung demnach eines Zielmuskels der rechten Körperseite) bestimmt. Typischerweise erfolgt diese Untersuchung an einem Muskel der rechten Hand, so dass demnach linkshemisphärisch das Handareal (engl. hand knob) mit TMS-Pulsen zu stimulieren ist. Als Approximation des linken Handareals bietet sich die EEG-Eletrkodenposition C3 an. Das Handareal stellt in der Anatomie eine sehr häufig

vorkommende auffällige Ausweitung oder einen Haken des präzentralen Gyrus in dorsaler Richtung dar. Neben der Motorschwellenbestimmung ist das Areal vereinzelt auch relevant bei der Behandlung bestimmter Schmerzsyndrome. C3 liegt auf halber Strecke zwischen Cz und T3/T7 (10% über präaurikulärem Punkt) bzw. 20% von der Sagittallinie auf der Interaurallinie (vom linken zum rechten präaurikulären Punkt) in Richtung linkes Ohr.

Motorschwellenbestimmung

Zunächst wird der Hotspot der motorischen Schwelle funktionell bestimmt, d.h. es wird diejenige Spulenposition über dem Schädel durch iteratives Testen bestimmt, bei der nach Abgabe von TMS-Einzelpulsen eine maximale und reliable Muskelantwort im Zielmuskel ausgelöst wird. Bei der Motorschwellenbestimmung für eine linksfrontal ausgerichtete antidepressive rTMS-Behandlung dient C3 (H. Kim et al., 2023) nur als erste Orientierung zum Start der Hotspot-Suche. Deshalb genügt es ggf. auch diesen Punkt zunächst nur grob anzunähern, indem man ausgehend vom Punkt Cz auf der interauriculären Linie je nach Fingerdicke zwei oder drei Fingerbreiten von der Sagittallinie in Richtung linkes Ohr abträgt. Entscheidend ist die Spulenorientierung bei der Motorschwellenbestimmung, die im 45-Grad-Winkel zur Sagittallinie sein sollte. Üblicherweise richtet sich der Handgriff der TMS-Spule in Richtung Hinterkopf. Zur Vereinfachung der Spulenausrichtung sollte mit einem flexiblen Geodreieck eine im 45°-Winkel relativ zur Sagittallinie eingezeichnete Hilfslinie verwendet werden, die zugleich auch die Verlängerung der Spule samt Griff mit darstellt (und entsprechend lang eingezeichnet wird). Alternativ kann der 45°-Winkel unter Fortführung der oben beschriebenen Annäherung des C3-Punktes dadurch bestimmt werden, indem auf der Nasion-Inion-Linie zwei oder drei Fingerbreiten in Richtung Nasion (lateral zu Cz) abgetragen werden und anschließend dieser Punkt mit dem angenäherten Punkt C3 verbunden wird.

Die über einer Hemisphäre bestimmte Motorschwelle ist ein Maß der kortikalen Erregbarkeit für eben jene Hemisphäre (bei bilateraler Stimulation ist sie also ggf. für beide Hemisphären zu bestimmen!) und dient als Grundlage zur Bestimmung der individuellen Stimulationsintensität. Die Motorschwelle ist zu Beginn jeder Behandlung zu messen. Die Motorschwelle ist meist über den Verlauf einer Behandlung unverändert (Nordmann et al., 2015), muss also nur einmalig zu Beginn der Behandlung bestimmt werden. Es wird das Monitoring psychotroper Medikation wie Benzodiazepine oder Neuroleptika empfohlen,

die die Motorschwelle oder den Behandlungserfolg beeinflussen können (Deppe et al., 2021; Hebel, Abdelnaim, et al., 2020; Ziemann et al., 2015). Optimalerweise benutzt man ein Elektromyogramm (EMG) und Oberflächenelektroden, um die Aktivität des zu stimulierenden Muskels objektiv zu messen. Zielparameter ist das motorisch evozierte Potential (MEP), eine biphasische Welle, die ca. 20-40ms nach dem TMS-Puls im EMG erkennbar ist. Ist kein EMG verfügbar, kann auf das sichtbare Zucken des Muskels als Zielparameter ausgewichen werden. Es ist zu beachten, dass die Schwelle mit visueller Kontrolle um ca. 10% höher liegt (Westin et al., 2014). Deshalb empfehlen wir – grob geschätzt – eine Reduktion der Behandlungsintensität um einen entsprechenden Faktor bei Bestimmung der Motorschwelle ohne EMG (z.B. anstatt 110% RMT-Intensität eine reduzierte Behandlungsintensität von 100% visuell bestimmte RMT).

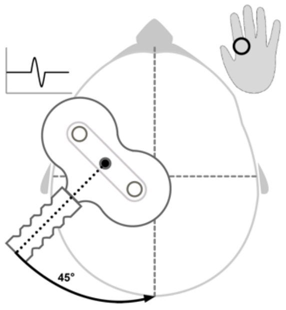

Zur Motorschwellenbestimmung gibt es ein zweistufiges **Vorgehen**:

a) Bestimmung des motorischen Hotspots: Zunächst ist wichtig, dass die/der Patientin/Patient bequem und möglichst entspannt im Untersuchungssessel sitzt. Die rechte Hand liegt locker auf der Armlehne auf. Es ist unbedingt darauf zu achten, dass die Muskulatur nicht vorgespannt ist und die Hand entspannt aufliegt. Bei einer Vorspannung spricht man von der aktiven Motorschwelle (AMT), die im klinischen Kontext selten relevant ist (siehe Abschnitt weiter unten). Die Ableitung der Muskelaktivität erfolgt über Oberflächenelektroden der rechten Hand. Stimuliert wird auf der linken Kopfseite (das motorische System ist kontralateral verschaltet). Üblich ist die Ableitung am Kleinfingerspreizer (ADM), Daumenballen (APB) oder am Zeigefinger (FDI). Die Spule sollte im 45° Winkel zur Mittellinie mit dem Griff nach hinten über dem vorher markierten Startpunkt zur Bestimmung des „Motor-Hotspot“ angelegt werden. Mit deutlich überschwelliger Stimulationsintensität (pragmatisch kann mit 55-60% Stimulatorleistung begonnen werden) wird systematisch an verschiedenen Orten über dem betroffenen Gehirnareal stimuliert, um so den Ort zu bestimmen, bei dem die maximale

Muskelkontraktion ausgelöst werden kann. Dazu soll die Spule in longitudinaler und sagittaler Ebene bewegt werden. Eine andere Möglichkeit ist das Einzeichnen eines Gitters, dessen Punkte systematisch untersucht werden.

b) Eigentliche Bestimmung der Motorschwelle: Ist dieser „Motor-Hotspot" gefunden, wird die Spulenpositionierung auf der Haube markiert, indem markante Punkte der Spule angezeichnet werden (z.B. Rundung zwischen Spulen der Schmetterlingsspule). Im nächsten Schritt wird die Intensität in einer Schwellenprozedur so lange angepasst, bis die Motorschwelle gefunden ist. Schwelle bedeutet, dass in nur 50% der Fälle eine überschwellige Antwort vorhanden ist (sichtbares Muskelzucken am betreffenden Muskel; motorisch evoziertes Potenzial > 50µV). Hierbei bieten sich mehrere mögliche Vorgehensweisen/Bestimmungsalgorithmen an:

- Das übliche Vorgehen ist das Abarbeiten eines bestimmten Algorithmus oder Schwellenverfahrens, bei der die Intensität so lange erhöht und/ oder verringert wird, bis der Schwellenwert gefunden ist (z.B. Rossini-Rothwell-Methode, Mills-Nithi-Methode). Bei korrekter Anwendung sind diese Verfahren recht zeitintensiv und benötigen viele Pulse, so dass es sich in der Praxis bewährt hat, die Stimulationsintensität so lange anzupassen, bis in nur noch 4 von 8 Stimulationen ein motorisch evoziertes Potential von über 50 µV zu finden ist (ohne MEG ein sichtbares Muskelzucken). Erfahrenes Personal kommt innerhalb weniger Minuten zu einem guten Ergebnis.
- Die „Schwellenjagd" (Awiszus, 2003) ermöglicht die Bestimmung der Schwelle mit weniger Pulsen und stellt ein adaptives Schwellenverfahren bekannt aus der Psychophysik dar. Unter Einsatz eines Rechners kann die Schwelle nach dem Maximum-Likelihood-Verfahren geschätzt werden und erlaubt eine schnelle Bestimmung der Schwelle. Hier sind semi-automatische Verfahren zu empfehlen, die erlauben, die Qualität der Antworten auf die einzelnen Stimulationspulse zu beurteilen, um ungültige ausschließen zu können. Ebenso gibt es eine Reihe neuerer Schätzverfahren in diesem Bereich, die nicht auf strengen mathematischen Vorannahmen basieren (Wang et al., 2023). Mögliche Tools zur Umsetzung finden sich unter www.clinicalresearcher.org

(Unterbereich „Software“, z.B. MTAT 2.1) oder https://tms-samt.github.io.

Aktive Motorschwelle und weitere Hinweise

Die vorangegangenen Ausführungen haben sich auf die Erfassung der Ruhemotorschwelle/RMT bezogen. In der klinischen Praxis hat die Erfassung der aktiven Motorschwelle/ AMT zumeist keine große Relevanz.

Jedoch kann sie als *alternatives Maß für die kortikale Erregbarkeit* bei PatientInnen mit pathologisch erhöhtem Muskeltonus dienen (etwa bei Tremor, Spastik, Dystonie oder auch bei krankheitsbedingten Bewegungsstörungen im Rahmen eines Schlaganfalls oder einer Demenz). In solchen Fällen ist eine zuverlässige Bestimmung der RMT häufig erschwert oder gar nicht möglich, weshalb die Erhebung der AMT eine praktikable Option bieten kann. Die AMT wird zudem *häufig in Protokollen der Theta-Burst-Stimulation (TBS) verwendet*, da die Behandlungsintensität häufiger bei der TBS häufiger auf Basis der AMT bestimmt wird. Typischerweise liegt die AMT dabei niedriger als die RMT, bezogen auf die maximale Stimulatorleistung (%MSO), da die kortikospinale Erregbarkeit durch die Muskelvordehnung erhöht ist.

Das Vorgehen zur Bestimmung der AMT ähnelt dem der RMT-Erfassung, allerdings erfolgt die Stimulation bei einem leicht voraktivierten. Zur Schwellenbestimmung kann ein algorithmisches Verfahren angewandt werden, wobei als Reaktionskriterium entweder motorisch evozierte Potenziale (MEPs) mit einer Amplitude von ≥200 µV oder eine sichtbare Muskelzuckung dienen. Ein wichtiger Hinweis betrifft die Standardisierbarkeit der AMT-Erhebung: Ohne objektive Hilfsmittel wie etwa einen Kraftsensor (Dynamometer) zur Quantifizierung der Muskelaktivierung kann es zu Abweichungen und schlechter Reproduzierbarkeit kommen.

Weitere praktische Überlegungen betreffen die Platzierung und Bewegung der TMS-Spule, insbesondere bei der Hotspot-Suche. Hierbei sollte darauf geachtet werden, *keinen übermäßigen Druck durch zu festes Andrücken der Spule auf den Kopf* auszuüben, um Schmerzen und Verletzungen auf Patientenseite zu vermeiden. Für die Durchführung von EMG-basierten Bestimmungen der Motorschwelle sollte ggf. ein Mitglied des Behandlungsteams über *fundierte Kenntnisse in der EMG-Messung* verfügen, um die Elektrodenplatzierung, Signalbeurteilung und Interpretation der

Muskelantworten fachgerecht vorzunehmen. Dies umfasst die Etablierung standardisierter Prozeduren der Hautvorbereitung (z. B. Reinigung mit Alkoholtüchern), die Einschätzung der EMG-Signalqualität zu Beginn der Schwellenbestimmung oder auch die Identifikation möglicher abweichender EMG-Reaktionsmuster aufgrund zugrundeliegender Pathologien (z.B. Ulnarisrinnen-Syndrom, periphere Neuropathie oder Radikulopathie).

7.2. Dorsolateraler präfrontaler Kortex

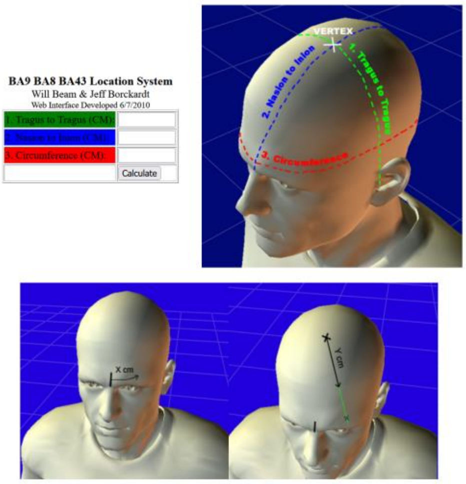

Der dorsolaterale präfrontale Kortex (engl. dorsolateral prefrontal cortex, Abk. DLPFC) ist prominentes Stimulationsziel bei der Behandlung depressiver Syndrome, der Negativsymptomatik im Rahmen der Schizophrenie, von Abhängigkeitssyndromen, der posttraumatischen Belastungsstörung und von Zwangsstörungen. Historisch wurde der DLPFC mittels der 5 cm, später dann der 6 cm-Regel ermittelt, bei der 5 bzw. 6 cm anterior dem Hotspot der Motorschwellenbestimmung und parallel zur Sagittallinie der Stimulationspunkt ermittelt wurde (Herwig, Padberg, et al., 2001). Standard ist inzwischen die Orientierung an den EEG-Punkten F3 auf der linken bzw. F4 auf der rechten Kopfseite. Ein einfaches Tool zur Ermittlung dieses Punktes ist die Beam-F3-Methode, die als Download oder als Online-Kalkulator zur Verfügung steht (http://clinicalresearcher.org/F3/). Dabei wird aus den Abständen Tragus-Tragus, Nasion-Inion und Kopfumfang ermittelt, wie weit der Stimulationspunkt von der Stirnmitte und vom Vertex entfernt liegt. Die Spule sollte im 45-Grad-Winkel zur Sagittallinie positioniert sein, was ausgemessen werden kann. Eine andere pragmatische Möglichkeit ist die Orientierung der Spule in Richtung Nasion oder FPz.

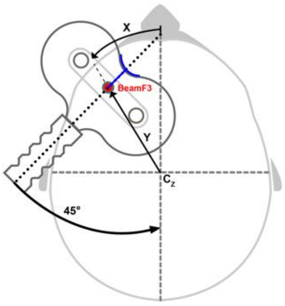

Anatomisch zielt man auf eine Stimulation des Übergangsgebiets der Brodmann Areale BA9 und BA46 bzw. der Grenze zwischen dem vorderen und mittleren Drittel des mittleren frontalen Gyrus ab (Mylius et al., 2013). Wie bereits erwähnt gibt es keine eindeutige Überlegenheit der neuronavigierten rTMS-Behandlung der Depression (Fitzgerald et al., 2009). Viele andere Studien beschäftigen sich nicht mit kontrollierten Vergleichen.

7.3. Temporoparietaler Übergangskortex

Der auditorische Kortex und der temporoparietale Übergangskortex (engl. temporoparietal junction; Abk. TPJ) sind Zielgebiet der Behandlung von akustischen Phantomwahrnehmungen und des Tinnitus. Die Evidenz für diese Protokolle ist gering. Trotzdem finden sich immer wieder therapie-resistente PatientInnen, bei denen ein individueller Heilversuch unternommen werden kann.

Die Markierung der *Position des auditorischen Kortex* richtet sich an Publikationen aus dem Bereich Tinnitus (Langguth et al., 2006). Dabei wird 2,5 cm von T3 aus auf der Linie Richtung C3 und von dort 1,5 cm im 90-Grad-Winkel nach dorsal gemessen. Die Spule wird parallel zur T3-Cz-Linie mit Spulengriff Richtung Cz orientiert.

Zur *Bestimmung der TPJ* ist das Einzeichnen einer Verbindungslinie zwischen zwei Punkten des 10-20-EEG-Systems nötig. Die Position des linken TPJ entspricht hierbei dem Punkt CP5, der rechte TPJ dem Punkt CP6. Der Spulengriff zeigt im 45° Winkel nach hinten oben. CP5/CP6 liegt in der Mitte zwischen T3/T4 und P3/P4. Die Punkte T3-CP5-P3 bzw. T4-CP6-P4 werden verbunden, um die Spulenorientierung (Griff der Spule in Richtung P3/P4) vorzugeben. Als Referenz zur Bestimmung dieser Positionen dienten zwei Arbeiten, die jedoch statt dem Begriff CP5 fälschlicherweise von TP3 sprechen (Herwig et al., 2003; Hoffman et al., 2003).

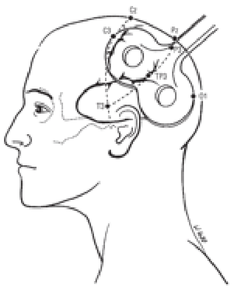

HOFFMAN ET AL., 2003

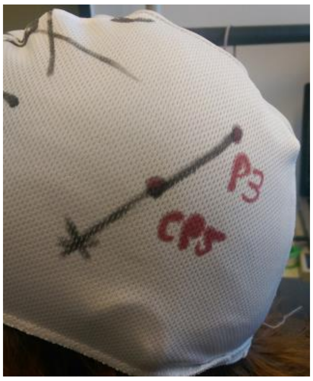

7.4. Supplementär-motorischer Kortex

Der Spulenmittelpunkt liegt 15% der Länge des Nasion-Inion-Abstandes vor Cz auf der Mittellinie. Der Spulengriff zeigt nach hinten in Richtung Inion. Eine übliche Anwendung ist die Behandlung von Zwangsstörungen (Mantovani et al., 2006).

8. Literaturverzeichnis

Aleman, A., Enriquez-Geppert, S., Knegtering, H., & Dlabac-de Lange, J. J. (2018). Moderate effects of noninvasive brain stimulation of the frontal cortex for improving negative symptoms in schizophrenia: Meta-analysis of controlled trials. *Neuroscience & Biobehavioral Reviews*, *89*, 111–118. https://doi.org/10.1016/j.neubiorev.2018.02.009

Awiszus, F. (2003). TMS and threshold hunting. *Supplements to Clinical Neurophysiology*, *56*, 13–23. https://doi.org/10.1016/s1567-424x(09)70205-3

Baeken, C., Brem, A.-K., Arns, M., Brunoni, A. R., Filipčić, I., Ganho-Ávila, A., Langguth, B., Padberg, F., Poulet, E., Rachid, F., Sack, A. T., Vanderhasselt, M.-A., & Bennabi, D. (2019). Repetitive transcranial magnetic stimulation treatment for depressive disorders. *Current Opinion in Psychiatry*, *32*(5), 409–415. https://doi.org/10.1097/YCO.0000000000000533

Blumberger, D. M., Vila-Rodriguez, F., Thorpe, K. E., Feffer, K., Noda, Y., Giacobbe, P., Knyahnytska, Y., Kennedy, S. H., Lam, R. W., Daskalakis, Z. J., & Downar, J. (2018). Effectiveness of theta burst versus high-frequency repetitive transcranial magnetic stimulation in patients with depression (THREE-D): a randomised non-inferiority trial. *The Lancet*, *391*(10131), 1683–1692. https://doi.org/10.1016/S0140-6736(18)30295-2

Cai, D.-B., Qin, Z.-J., Lan, X.-J., Liu, Q.-M., Qin, X.-D., Wang, J.-J., Goya-Maldonado, R., Huang, X.-B., Ungvari, G. S., Ng, C. H., Zheng, W., & Xiang, Y.-T. (2023). Accelerated intermittent theta burst stimulation for major depressive disorder or bipolar depression: A systematic review and meta-analysis. *Asian Journal of Psychiatry*, *85*, 103618. https://doi.org/10.1016/j.ajp.2023.103618

Chang, J., Chu, Y., Ren, Y., Li, C., Wang, Y., & Chu, X.-P. (2020). Maintenance treatment of transcranial magnetic stimulation (TMS) for treatment-resistant depression patients responding to acute TMS treatment. *International Journal of Physiology, Pathophysiology and Pharmacology*, *12*(5), 128–133.

Cole, E. J., Phillips, A. L., Bentzley, B. S., Stimpson, K. H., Nejad, R., Barmak, F., Veerapal, C., Khan, N., Cherian, K., Felber, E., Brown, R., Choi, E., King, S., Pankow, H., Bishop, J. H., Azeez, A., Coetzee, J., Rapier, R., Odenwald, N., . . . Williams, N. R. (2022). Stanford Neuromodulation Therapy (SNT): A Double-Blind Randomized Controlled Trial. *American Journal of Psychiatry*, *179*(2), 132–141. https://doi.org/10.1176/appi.ajp.2021.20101429

Cole, E. J., Stimpson, K. H., Bentzley, B. S., Gulser, M., Cherian, K., Tischler, C., Nejad, R., Pankow, H., Choi, E., Aaron, H., Espil, F. M., Pannu, J., Xiao, X., Duvio, D., Solvason, H. B., Hawkins, J., Guerra, A., Jo, B., Raj, K. S., . . . Williams, N. R. (2020). Stanford Accelerated Intelligent Neuromodulation Therapy for Treatment-Resistant Depression. *American Journal of Psychiatry*, *177*(8), 716–726. https://doi.org/10.1176/appi.ajp.2019.19070720

d'Andrea, G., Mancusi, G., Santovito, M. C., Marrangone, C., Martino, F., Santorelli, M., Miuli, A., Di Carlo, F., Signorelli, M. S., Clerici, M., Pettorruso, M., & Martinotti, G. (2023). Investigating the Role of Maintenance TMS Protocols for Major Depression: Systematic Review and Future Perspectives for Personalized Interventions. *Journal of Personalized Medicine*, *13*(4), 697. https://doi.org/10.3390/jpm13040697

Deppe, M., Abdelnaim, M., Hebel, T., Kreuzer, P. M., Poeppl, T. B., Langguth, B., & Schecklmann, M. (2021). Concomitant lorazepam use and antidepressive efficacy of repetitive transcranial magnetic stimulation in a naturalistic setting. *European Archives of Psychiatry and Clinical Neuroscience*, *271*(1), 61–67. https://doi.org/10.1007/s00406-020-01160-9

Dougall, N., Maayan, N., Soares-Weiser, K., McDermott, L. M., & McIntosh, A. (2015). Transcranial magnetic stimulation (TMS) for schizophrenia. *Cochrane Database of Systematic Reviews*, *2015*(8). https://doi.org/10.1002/14651858.CD006081.pub2

Duprat, R., Desmyter, S., Rudi, D. R., van Heeringen, K., van den Abbeele, D., Tandt, H., Bakic, J., Pourtois, G., Dedoncker, J., Vervaet, M., van Autreve, S., Lemmens, G. M. D., & Baeken, C. (2016). Accelerated intermittent theta burst stimulation treatment in medication-resistant major depression: A fast road to remission? *Journal of Affective Disorders*, *200*, 6–14. https://doi.org/10.1016/j.jad.2016.04.015

Fitzgerald, P. B., Hoy, K., McQueen, S., Maller, J. J., Herring, S., Segrave, R., Bailey, M., Been, G., Kulkarni, J., & Daskalakis, Z. J. (2009). A Randomized Trial of rTMS Targeted with MRI Based Neuro-Navigation in Treatment-Resistant Depression. *Neuropsychopharmacology*, *34*(5), 1255–1262. https://doi.org/10.1038/npp.2008.233

Fitzsimmons, S. M., van der Werf, Y. D., van Campen, A. D., Arns, M., Sack, A. T., Hoogendoorn, A. W., van den Heuvel, O. A., van Balkom, A. J., Batelaan, N. M., van Eijndhoven, P., Hendriks, G.-J., van Oostrom, I., van Oppena, P., Schruers, K. R., Tendolkar, I., & Vriend, C. (2022). Repetitive transcranial magnetic stimulation for

obsessive-compulsive disorder: A systematic review and pairwise/network meta-analysis. *Journal of Affective Disorders*, *302*, 302–312. https://doi.org/10.1016/j.jad.2022.01.048

Folmer, R. L., Theodoroff, S. M., Casiana, L., Shi, Y., Griest, S., & Vachhani, J. (2015). Repetitive Transcranial Magnetic Stimulation Treatment for Chronic Tinnitus. *JAMA Otolaryngology–Head & Neck Surgery*, *141*(8), 716. https://doi.org/10.1001/jamaoto.2015.1219

Gay, A., Cabe, J., Chazeron, I. de, Lambert, C., Defour, M., Bhoowabul, V., Charpeaud, T., Tremey, A., Llorca, P.-M., Pereira, B., & Brousse, G. (2022). Repetitive Transcranial Magnetic Stimulation (rTMS) as a Promising Treatment for Craving in Stimulant Drugs and Behavioral Addiction: A Meta-Analysis. *Journal of Clinical Medicine*, *11*(3). https://doi.org/10.3390/jcm11030624

Giustiniani, A., Vallesi, A., Oliveri, M., Tarantino, V., Ambrosini, E., Bortoletto, M., Masina, F., Busan, P., Siebner, H. R., Fadiga, L., Koch, G., Leocani, L., Lefaucheur, J. P., Rotenberg, A., Zangen, A., Violante, I. R., Moliadze, V., Gamboa, O. L., Ugawa, Y., . . . Burgio, F. (2022). A questionnaire to collect unintended effects of transcranial magnetic stimulation: A consensus based approach. *Clinical Neurophysiology : Official Journal of the International Federation of Clinical Neurophysiology*, *141*, 101–108. https://doi.org/10.1016/j.clinph.2022.06.008

Haesebaert, F., Moirand, R., Schott-Pethelaz, A.-M., Brunelin, J., & Poulet, E. (2018). Usefulness of repetitive transcranial magnetic stimulation as a maintenance treatment in patients with major depression. *The World Journal of Biological Psychiatry*, *19*(1), 74–78. https://doi.org/10.1080/15622975.2016.1255353

He, H., Lu, J., Yang, L., Zheng, J., Gao, F., Zhai, Y., Feng, J., Fan, Y., & Ma, X. (2017). Repetitive transcranial magnetic stimulation for treating the symptoms of schizophrenia: A PRISMA compliant meta-analysis. *Clinical Neurophysiology*, *128*(5), 716–724. https://doi.org/10.1016/j.clinph.2017.02.007

Hebel, T., Abdelnaim, M., Deppe, M., Langguth, B., & Schecklmann, M. (2020). Attenuation of antidepressive effects of transcranial magnetic stimulation in patients whose medication includes drugs for psychosis. *Journal of Psychopharmacology*, *34*(10), 1119–1124. https://doi.org/10.1177/0269881120922965

Hebel, T., Grözinger, M., Landgrebe, M., Padberg, F., Schecklmann, M., Schlaepfer, T., Schönfeldt-Lecuona, C., Ullrich, H., Zwanzger, P., Langguth, B., Bajbouj, M., Bewernick, B.,

Brinkmann, K., Cordes, J., Di Pauli, J., Eichhammer, P., Freundlieb, N., Hajak, G., Höppner-Buchmann, J., . . . Zilles-Wegner, D. (2022). Evidence and expert consensus based German guidelines for the use of repetitive transcranial magnetic stimulation in depression. *The World Journal of Biological Psychiatry*, *23*(5), 327–348. https://doi.org/10.1080/15622975.2021.1995810

Hebel, T., Schecklmann, M., & Langguth, B. (2020). Transcranial magnetic stimulation in the treatment of depression during pregnancy: a review. *Archives of Women's Mental Health*, *23*(4), 469–478. https://doi.org/10.1007/s00737-019-01004-z

Herwig, U., Padberg, F., Unger, J., Spitzer, M., & Schönfeldt-Lecuona, C. (2001). Transcranial magnetic stimulation in therapy studies: examination of the reliability of "standard" coil positioning by neuronavigation. *Biological Psychiatry*, *50*(1), 58–61. https://doi.org/10.1016/S0006-3223(01)01153-2

Herwig, U., Satrapi, P., & Schönfeldt-Lecuona, C. (2003). Using the International 10-20 EEG System for Positioning of Transcranial Magnetic Stimulation. *Brain Topography*, *16*(2), 95–99. https://doi.org/10.1023/B:BRAT.0000006333.93597.9d

Herwig, U., Schönfeldt-Lecuona, C., Wunderlich, A. P., Tiesenhausen, C. von, Thielscher, A., Walter, H., & Spitzer, M. (2001). The navigation of transcranial magnetic stimulation. *Psychiatry Research: Neuroimaging*, *108*(2), 123–131. https://doi.org/10.1016/S0925-4927(01)00121-4

Hızlı Sayar, G., Ozten, E., Tufan, E., Cerit, C., Kağan, G., Dilbaz, N., & Tarhan, N. (2014). Transcranial magnetic stimulation during pregnancy. *Archives of Women's Mental Health*, *17*(4), 311–315. https://doi.org/10.1007/s00737-013-0397-0

Hoffman, R. E., Hawkins, K. A., Gueorguieva, R., Boutros, N. N., Rachid, F., Carroll, K., & Krystal, J. H. (2003). Transcranial magnetic stimulation of left temporoparietal cortex and medication-resistant auditory hallucinations. *Archives of General Psychiatry*, *60*(1), 49–56. https://doi.org/10.1001/archpsyc.60.1.49

Huang, Y.-Z.; Edwards, M. J.; Rounis, E.; Bhatia, K. P.; Rothwell, J. C. (2005). Theta burst stimulation of the human motor cortex. *Neuron, 45* (2), 201–206. https://doi.org/10.1016/j.neuron.2004.12.033

Hunter, A. M., Minzenberg, M. J., Cook, I. A., Krantz, D. E., Levitt, J. G., Rotstein, N. M., Chawla, S. A., & Leuchter, A. F. (2019). Concomitant medication use and clinical outcome of repetitive Transcranial Magnetic Stimulation (rTMS) treatment of Major Depressive Disorder. *Brain and Behavior*, *9*(5), e01275. https://doi.org/10.1002/brb3.1275

Hyde, J., Carr, H., Kelley, N., Seneviratne, R., Reed, C., Parlatini, V., Garner, M., Solmi, M., Rosson, S., Cortese, S., & Brandt, V. (2022). Efficacy of neurostimulation across mental disorders: systematic review and meta-analysis of 208 randomized controlled trials. *Molecular Psychiatry*, *27*(6), 2709–2719. https://doi.org/10.1038/s41380-022-01524-8

Kim, D. R., Wang, E., McGeehan, B., Snell, J., Ewing, G., Iannelli, C., O'Reardon, J. P., Sammel, M. D., & Epperson, C. N. (2019). Randomized controlled trial of transcranial magnetic stimulation in pregnant women with major depressive disorder. *Brain Stimulation*, *12*(1), 96–102. https://doi.org/10.1016/j.brs.2018.09.005

Kim, H., Wright, D. L., Rhee, J., & Kim, T. (2023). C3 in the 10-20 system may not be the best target for the motor hand area. *Brain Research*, *1807*, 148311. https://doi.org/10.1016/j.brainres.2023.148311

Kishi, T., Ikuta, T., Sakuma, K., Hatano, M., Matsuda, Y., Wilkening, J., Goya-Maldonado, R., Tik, M., Williams, N. R., Kito, S., & Iwata, N. (2024). Theta burst stimulation for depression: A systematic review and network and pairwise meta-analysis. *Molecular Psychiatry*, *29*(12), 3893–3899. https://doi.org/10.1038/s41380-024-02630-5

Koessler, L., Maillard, L., Benhadid, A., Vignal, J. P., Felblinger, J., Vespignani, H., & Braun, M. (2009). Automated cortical projection of EEG sensors: Anatomical correlation via the international 10–10 system. *NeuroImage*, *46*(1), 64–72. https://doi.org/10.1016/j.neuroimage.2009.02.006

Langguth, B., Zowe, M., Landgrebe, M., Sand, P., Kleinjung, T., Binder, H., Hajak, G., & Eichhammer, P. (2006). Transcranial magnetic stimulation for the treatment of tinnitus: A new coil positioning method and first results. *Brain Topography*, *18*(4), 241–247. https://doi.org/10.1007/s10548-006-0002-1

Lefaucheur, J.-P., Aleman, A., Baeken, C., Benninger, D. H., Brunelin, J., Di Lazzaro, V., Filipović, S. R., Grefkes, C., Hasan, A., Hummel, F. C., Jääskeläinen, S. K., Langguth, B., Leocani, L., Londero, A., Nardone, R., Nguyen, J.-P., Nyffeler, T., Oliveira-Maia, A. J., Oliviero, A., . . . Ziemann, U. (2020). Evidence-based guidelines on the therapeutic use of

repetitive transcranial magnetic stimulation (rTMS): An update (2014–2018). *Clinical Neurophysiology*, *131*(2), 474–528. https://doi.org/10.1016/j.clinph.2019.11.002

Lefaucheur, J.-P., André-Obadia, N., Antal, A., Ayache, S. S., Baeken, C., Benninger, D. H., Cantello, R. M., Cincotta, M., Carvalho, M. de, Ridder, D. de, Devanne, H., Di Lazzaro, V., Filipović, S. R., Hummel, F. C., Jääskeläinen, S. K., Kimiskidis, V. K., Koch, G., Langguth, B., Nyffeler, T., . . . Garcia-Larrea, L. (2014). Evidence-based guidelines on the therapeutic use of repetitive transcranial magnetic stimulation (rTMS). *Clinical Neurophysiology*, *125*(11), 2150–2206. https://doi.org/10.1016/j.clinph.2014.05.021

Liu, B., Zhang, Y., Zhang, L., & Li, L. (2014). Repetitive transcranial magnetic stimulation as an augmentative strategy for treatment-resistant depression, a meta-analysis of randomized, double-blind and sham-controlled study. *BMC Psychiatry*, *14*(1), 342. https://doi.org/10.1186/s12888-014-0342-4

Liu, H., Wang, X., Gong, T., Xu, S., Zhang, J., Yan, L., Zeng, Y., Yi, M., & Qian, Y. (2024). Neuromodulation treatments for post-traumatic stress disorder: A systematic review and network meta-analysis covering efficacy, acceptability, and follow-up effects. *Journal of Anxiety Disorders*, *106*, 102912. https://doi.org/10.1016/j.janxdis.2024.102912

Mantovani, A., Lisanby, S. H., Pieraccini, F., Ulivelli, M., Castrogiovanni, P., & Rossi, S. (2006). Repetitive transcranial magnetic stimulation (rTMS) in the treatment of obsessive–compulsive disorder (OCD) and Tourette‘s syndrome (TS). *The International Journal of Neuropsychopharmacology*, *9*(01), 95. https://doi.org/10.1017/S1461145705005729

McGirr, A., Devoe, D. J., Raedler, A., Debert, C. T., Ismail, Z., & Berlim, M. T. (2021). Repetitive Transcranial Magnetic Stimulation for the Treatment of Post-traumatic Stress Disorder: A Systematic Review and Network Meta-analysis: La Stimulation Magnétique Transcrânienne Répétitive Pour le Traitement du Trouble de Stress Post-Traumatique : Une Revue Systématique et une Méta-Analyse en Réseau. *Canadian Journal of Psychiatry. Revue Canadienne De Psychiatrie*, *66*(9), 763–773. https://doi.org/10.1177/0706743720982432

Mylius, V., Ayache, S. S., Ahdab, R., Farhat, W. H., Zouari, H. G., Belke, M., Brugières, P., Wehrmann, E., Krakow, K., Timmesfeld, N., Schmidt, S., Oertel, W. H., Knake, S., & Lefaucheur, J. P. (2013). Definition of DLPFC and M1 according to anatomical landmarks for navigated brain stimulation: Inter-rater reliability, accuracy, and influence of gender and age. *NeuroImage*, *78*, 224–232. https://doi.org/10.1016/j.neuroimage.2013.03.061

Osoegawa, C., Gomes, J. S., Grigolon, R. B., Brietzke, E., Gadelha, A., Lacerda, A. L., Dias, Á. M., Cordeiro, Q., Laranjeira, R., Jesus, D. de, Daskalakis, Z. J., Brunelin, Jz., Cordes, J., & Trevizol, A. P. (2018). Non-invasive brain stimulation for negative symptoms in schizophrenia: An updated systematic review and meta-analysis. *Schizophrenia Research*, *197*, 34–44. https://doi.org/10.1016/j.schres.2018.01.010

Patel, R., Silla, F., Pierce, S., Theule, J., & Girard, T. A. (2020). Cognitive functioning before and after repetitive transcranial magnetic stimulation (rTMS): A quantitative meta-analysis in healthy adults. *Neuropsychologia*, *141*, 107395. https://doi.org/10.1016/j.neuropsychologia.2020.107395

Plewnia, C., Brendel, B., Schwippel, T., Martus, P., Cordes, J., Hasan, A., & Fallgatter, A. J. (2018). Treatment of auditory hallucinations with bilateral theta burst stimulation (cTBS): Protocol of a randomized, double-blind, placebo-controlled, multicenter trial. *European Archives of Psychiatry and Clinical Neuroscience*, *268*(7), 663–673. https://doi.org/10.1007/s00406-017-0861-3

Rossi, S., Antal, A., Bestmann, S., Bikson, M., Brewer, C., Brockmöller, J., Carpenter, L. L., Cincotta, M., Chen, R., Daskalakis, J. D., Di Lazzaro, V., Fox, M. D., George, M. S., Gilbert, D., Kimiskidis, V. K., Koch, G., Ilmoniemi, R. J., Lefaucheur, J. P., Leocani, L., . . . Hallett, M. (2021). Safety and recommendations for TMS use in healthy subjects and patient populations, with updates on training, ethical and regulatory issues: Expert Guidelines. *Clinical Neurophysiology*, *132*(1), 269–306. https://doi.org/10.1016/j.clinph.2020.10.003

Rossi, S., Hallett, M., Rossini, P. M., & Pascual-Leone, A. (2009). Safety, ethical considerations, and application guidelines for the use of transcranial magnetic stimulation in clinical practice and research. *Clinical Neurophysiology*, *120*(12), 2008–2039. https://doi.org/10.1016/j.clinph.2009.08.016

Rutherford, G., Lithgow, B., & Moussavi, Z. (2020). Transcranial magnetic stimulation safety from operator exposure perspective. *Medical & Biological Engineering & Computing*, *58*(2), 249–256. https://doi.org/10.1007/s11517-019-02084-w

Schönfeldt-Lecuona, C., Cárdenas-Morales, L., Moreno-Aguirre, A., Dorn, K., Langguth, B., Brühl, A. B., Kammer, T., & Herwig, U. (2012). Effect of 1 Hz Repetitive Transcranial Magnetic Stimulation Over the Auditory Cortex on Audiometry and Otoacustic Emissions. *Brain Topography*, *25*(3), 241–247. https://doi.org/10.1007/s10548-012-0218-1

Schönfeldt-Lecuona, C., Lefaucheur, J.-P., Cardenas-Morales, L., Wolf, R. C., Kammer, T., & Herwig, U. (2010). The value of neuronavigated rTMS for the treatment of depression. *Neurophysiologie Clinique/Clinical Neurophysiology*, *40*(1), 37–43. https://doi.org/10.1016/j.neucli.2009.06.004

Schönfeldt-Lecuona, C., Thielscher, A., Freudenmann, R. W., Kron, M., Spitzer, M., & Herwig, U. (2005). Accuracy of Stereotaxic Positioning of Transcranial Magnetic Stimulation. *Brain Topography*, *17*(4), 253–259. https://doi.org/10.1007/s10548-005-6033-1

Shafi, M. M. (2019). Seizures with TMS: Much ado about (almost) nothing? *Clinical Neurophysiology*, *130*(8), 1397–1398. https://doi.org/10.1016/j.clinph.2019.04.315

Tseng, P.-T., Zeng, B.-S., Hung, C.-M., Liang, C.-S., Stubbs, B., Carvalho, A. F., Brunoni, A. R., Su, K.-P., Tu, Y.-K., Wu, Y.-C., Chen, T.-Y., Li, D.-J., Lin, P.-Y., Hsu, C.-W., Chen, Y.-W., Suen, M.-W., Satogami, K., Takahashi, S., Wu, C.-K., . . . Li, C.-T. (2022). Assessment of Noninvasive Brain Stimulation Interventions for Negative Symptoms of Schizophrenia. *JAMA Psychiatry*, *79*(8), 770. https://doi.org/10.1001/jamapsychiatry.2022.1513

Wang, B., Peterchev, A. V., & Goetz, S. M. (2023). Three novel methods for determining motor threshold with transcranial magnetic stimulation outperform conventional procedures. *Journal of Neural Engineering*, *20*(5). https://doi.org/10.1088/1741-2552/acf1cc

Westin, G. G., Bassi, B. D., Lisanby, S. H., & Luber, B. (2014). Determination of motor threshold using visual observation overestimates transcranial magnetic stimulation dosage: Safety implications. *Clinical Neurophysiology : Official Journal of the International Federation of Clinical Neurophysiology*, *125*(1), 142–147. https://doi.org/10.1016/j.clinph.2013.06.187

Wilson, S., Croarkin, P. E., Aaronson, S. T., Carpenter, L. L., Cochran, M., Stultz, D. J., & Kozel, F. A. (2022). Systematic review of preservation TMS that includes continuation, maintenance, relapse-prevention, and rescue TMS. *Journal of Affective Disorders*, *296*, 79–88. https://doi.org/10.1016/j.jad.2021.09.040

Ye, S.-Y., Chen, C.-N., Wei, B., Zhan, J.-Q., Li, Y.-H., Zhang, C., Huang, J.-J., & Yang, Y.-J. (2024). The efficacy and safety of continuous theta burst stimulation for auditory hallucinations: A systematic review and meta-analysis of randomized controlled trials. *Frontiers in Psychiatry*, *15*, 1446849. https://doi.org/10.3389/fpsyt.2024.1446849

Zaidi, A., Shami, R., Sewell, I. J., Cao, X., Giacobbe, P., Rabin, J. S., Goubran, M., Hamani, C., Swardfager, W., Davidson, B., Lipsman, N., & Nestor, S. M. (2024). Antidepressant class and concurrent rTMS outcomes in major depressive disorder: A systematic review and meta-analysis. *EClinicalMedicine*, *75*, 102760. https://doi.org/10.1016/j.eclinm.2024.102760

Zhang, J. J. Q., Fong, K. N. K., Ouyang, R.-G., Siu, A. M. H., & Kranz, G. S. (2019). Effects of repetitive transcranial magnetic stimulation (rTMS) on craving and substance consumption in patients with substance dependence: A systematic review and meta-analysis. *Addiction (Abingdon, England)*, *114*(12), 2137–2149. https://doi.org/10.1111/add.14753

Zhang, W., Deng, B., Xie, F., Zhou, H., Guo, J.-F., Jiang, H., Sim, A., Tang, B., & Wang, Q. (2022). Efficacy of repetitive transcranial magnetic stimulation in Parkinson's disease: A systematic review and meta-analysis of randomised controlled trials. *EClinicalMedicine*, *52*, 101589. https://doi.org/10.1016/j.eclinm.2022.101589

Ziemann, U., Reis, J., Schwenkreis, P., Rosanova, M., Strafella, A., Badawy, R., & Müller-Dahlhaus, F. (2015). TMS and drugs revisited 2014. *Clinical Neurophysiology*, *126*(10), 1847–1868. https://doi.org/10.1016/j.clinph.2014.08.028

Anhang A: Textvorschlag Aufklärungsbogen zur Behandlung

Aufklärung und Einwilligung: Behandlung mit Magnetstimulation

Sehr geehrte Patientin, sehr geehrter Patient,

zur Behandlung Ihrer Erkrankung ist eine magnetische Hirnstimulation, d.h. der Einsatz der sogenannten repetitiven Transkraniellen Magnetstimulation (rTMS) geplant. Es handelt sich dabei um ein wissenschaftlich anerkanntes Verfahren, dessen Wirksamkeit bei bestimmten neurologischen und psychiatrischen Erkrankungen bekannt ist und gegenwärtig weiter untersucht wird.

Was ist Transkranielle Magnetstimulation?

Bei der Transkraniellen Magnetstimulation (TMS) werden gezielt bestimmte Areale des Gehirns stimuliert (transkraniell = durch den Schädel hindurch). Hierbei wird eine Spule außen an den Kopf angelegt. Über diese Spule werden sehr kurze Magnetpulse erzeugt. Dies ermöglicht die gezielte Beeinflussung von Gehirnaktivität. Ein Anwendungsbeispiel ist in der neurologischen Routinediagnostik das Auslösen eines Muskelzuckens in den Gliedmaßen durch einen einzelnen Magnetimpuls über dem Bewegungsareal des Gehirns. Zur Behandlung mit TMS werden viele solcher Magnetpulse am Stück gegeben. Das nennt sich repetitive TMS (rTMS; repetitiv = wiederholte Impulse) und verursacht länger andauernde Veränderungen der Gehirnaktivität, was zur Symptomlinderung führen kann.

Wie läuft eine Behandlung mit der Magnetstimulation ab?

Vor oder am ersten Behandlungstag findet die Bestimmung der Stimulationsintensität statt. Dazu wird das Bewegungsareal im Gehirn mit einzelnen Pulsen stimuliert. Entstehende Muskelzuckungen können durch Elektroden, die an der entsprechenden Gliedmaße (z.B. am Kleinfinger) abgebracht werden, aufgezeichnet werden. Die eigentliche Behandlung findet täglich werktags statt (also insgesamt 5-Mal pro Woche) und erfolgt in einem Zeitraum von einer oder mehreren Wochen. Jede Behandlungssitzung dauert wenige Minuten. Allerdings ist damit zu rechnen, dass sich die Behandlungswirkung über die Behandlungstage akkumuliert.

Welche Nebenwirkungen sind zu erwarten?

1. Für die Messung der Stimulationsintensität werden Elektroden an den Gliedmaßen angebracht. Dabei werden Hautdesinfektion (meist auf Alkoholbasis) und Elektrodenpaste oder spezielle Klebeelektroden verwendet, welche leichte und vorübergehende Hautirritationen hervorrufen können.
2. Die einzelnen Stimulationspulse können mit Kribbeln auf der Kopfhaut oder Zucken bestimmter Muskeln (z. B. Kiefermuskel) verbunden sein, was leicht schmerzhaft sein kann.
3. Die einzelnen Magnetpulse sind mit einem relativ lauten Geräusch verbunden. Sie sollten deshalb Gehörschutz tragen, den Sie von uns bekommen.

4. Auch können während und nach der Stimulation Kopfschmerzen auftreten, die meist vorübergehend sind und gut mit Kopfschmerzmitteln behandelbar sind.
5. In Einzelfällen kann die Stimulation irritierend erlebt werden, was zu Schwindel oder Kreislaufproblemen und selten zu einer kurzen Ohnmacht führen kann.
6. Es besteht außerdem das Risiko, dass durch die Stimulation mit rTMS ein epileptischer Anfall ausgelöst wird. Dieses Risiko wird allerdings als sehr gering eingeschätzt und ist v.a. bei Personen mit neurologischen Grunderkrankungen wie Epilepsie erhöht.
7. Ebenso wie wir Ihnen keine Besserung versprechen können, können wir eine Zunahme Ihrer Symptome durch die Behandlung auch nicht sicher ausschließen.
8. Implantierte Geräte wie ein Herzschrittmacher können beschädigt werden oder können in Wechselwirkung mit der Magnetstimulation den Körper schädigen.

Welche gesundheitlichen Aspekte sind zu berücksichtigen?

Bei bestimmten Personengruppen sollte die Behandlung nicht erfolgen oder es ist zumindest eine besondere Vorsicht geboten, da das Risiko für Nebenwirkungen erhöht ist. Folgende Kontraindikationen sind relevant:

	Trifft zu		Wenn ja,<br>genaue Angaben und Bewertung:
Elektrische oder metallische Gegenstände im/am Kopf oder Körper, insbesondere:<br>• implantierte Geräte (Herzschrittmacher, Cochlea-Implantat etc.)<br>• Metallsplitter<br>• Gefäßclips<br>• Implantate allgemein	O Nein	O Ja	Ohrringe, Piercings, Zahnfüllungen, -kronen, -implantate stellen kein Problem dar.
Neurologische Bedingungen, insbesondere:<br>• Epilepsie<br>• einmaliger epileptischer Anfall<br>• Schädel-Hirn-Trauma<br>• Schlaganfall<br>• Gehirnschädigung	O Nein	O Ja	
Schwerwiegende, nicht stabil eingestellte Erkrankung.	O Nein	O Ja	
Neigung zu Ohnmacht.	O Nein	O Ja	
Schwangerschaft.	O Nein	O Ja	
Einnahme von Medikation, insbesondere mit Änderung der Krampfschwelle.	O Nein	O Ja	
Vorbehandlung mit rTMS.	O Nein	O Ja	Wenn ja, wie vertragen?
Ich hatte bereits eine Manie.	O Nein	O Ja	

Wie groß sind die Erfolgsaussichten?

Die rTMS ist ein relativ neues Verfahren, deren Wirksamkeit bereits bei vielen Erkrankungen untersucht wird. Insgesamt fehlen aber noch weitere Studien, um gesicherte Aussagen über die Wirkung bei bestimmten Erkrankungen treffen zu können. Der gegenwärtige Forschungsstand basiert auf evidenz-basierten Richtlinien internationaler Experten und ist folgender:

**Erkrankung**	**Wirksamkeit der rTMS**
Depression	nachgewiesen wirksam
Posttraumatische Belastungsstörung	wahrscheinlich wirksam
Schizophrenie	wahrscheinlich wirksam bei akustischen Halluzinationen<br>möglich wirksam bei Negativsymptomatik
Tinnitus, Zwang	möglich wirksam
Substanzmissbrauch und -abhängigkeit	möglich wirksam bei Nikotin
Angststörungen	bisher keine Empfehlung möglich

Wenn Sie Fragen zur Behandlung haben, können Sie sich jederzeit an das Behandlungsteam wenden. Sollten Sie Nebenwirkungen irgendwelcher Art verspüren, teilen Sie uns diese bitte unverzüglich mit. Selbstverständlich dürfen Sie die Behandlung jederzeit abbrechen. Sie wissen, dass es andere Behandlungsmöglichkeiten der Depression gibt.

Geplant ist folgender Behandlungsablauf (Anzahl Sitzungen in welchem Zeitraum): ____

Eine Behandlungssitzung dauert ca. __ Minuten. Neben den Behandlungen werden Befunde durch die Behandler in separaten Gesprächen und in Form von Fragebögen erhoben.

Beschreibung der Behandlung: __________________ Indikation: ______________________________

Bedenkzeit: __________________________ O auf Bedenkzeit verzichtet

Kopie der Aufklärung und Einwilligung

O An Patientin/Patienten ausgehändigt. O Patientin/Patient mit Verzicht auf eine Kopie.

Bemerkungen: ____

Ort: __________________________ Datum: ___.___.______ Uhrzeit: ___.___ Uhr

__________________________	__________________________	__________________________
Name aufklärende Person	Name Patientin/Patient	Name Fachärztin/Facharzt
__________________________	__________________________	__________________________
Unterschrift aufklärende Person	Unterschrift Patientin/Patient	Unterschrift Fachärztin/Facharzt

Anhang B: Textvorschlag Abfrage von Nebenwirkungen

Fragebogen zu Nebenwirkungen der rTMS-Behandlung

Haben Sie während des Behandlungszeitraums eine der folgenden Nebenwirkungen während oder nach den rTMS-Behandlungen wahrgenommen?
Bewerten Sie zunächst für jede Empfindung, ob Sie bei Ihnen im Behandlungszeitraum überhaupt vorgelegen hat (Kreuz in der ersten Spalte setzen).
Falls die Empfindung vorgelegen hat, bitten wir Sie jeweils die folgenden Aspekte zu bewerten:

1. Bewerten Sie bitte das **Ausmaß** der Empfindung auf einer Skala von 1 bis 4:
   - **1** = Gering
   - **2** = Mäßig
   - **3** = Stark
   - **4** = Sehr stark
2. Geben Sie bitte zusätzlich **die Dauer** der Empfindung über den gesamten Behandlungszeitraum hinweg an:
   - **1** = Selten (einmal oder zweimal)
   - **2** = Gelegentlich (an mehreren Tagen)
   - **3** = Häufig (an mehr als der Hälfte der Tage)
   - **4** = Über den gesamten Behandlungszeitraum hinweg

Bitte geben Sie die Empfindungen wie folgt an:

**Empfindung**	**Lag vor?**		**Ausmaß (1-4)** Nur bei Vorliegen				**Dauer (1-4)** Nur bei Vorliegen			
Schmerz an der Kopfhaut	Ja	Nein	1	2	3	4	1	2	3	4
Zahnschmerzen	Ja	Nein	1	2	3	4	1	2	3	4
Kribbeln an der Kopfhaut	Ja	Nein	1	2	3	4	1	2	3	4
Juckreiz	Ja	Nein	1	2	3	4	1	2	3	4
Brennen oder Hitzegefühl	Ja	Nein	1	2	3	4	1	2	3	4
Kopfschmerzen	Ja	Nein	1	2	3	4	1	2	3	4
Geräusche (z. B. Tinnitus)	Ja	Nein	1	2	3	4	1	2	3	4
Muskelkontraktionen	Ja	Nein	1	2	3	4	1	2	3	4
Müdigkeit/Schläfrigkeit	Ja	Nein	1	2	3	4	1	2	3	4
Hörveränderungen	Ja	Nein	1	2	3	4	1	2	3	4
Stimmungsschwankungen (Depression)	Ja	Nein	1	2	3	4	1	2	3	4
Stimmungsschwankungen (Euphorie)	Ja	Nein	1	2	3	4	1	2	3	4
Übelkeit	Ja	Nein	1	2	3	4	1	2	3	4
Schwindelgefühle	Ja	Nein	1	2	3	4	1	2	3	4
Nackensteifigkeit/-schmerzen	Ja	Nein	1	2	3	4	1	2	3	4
Druckgefühl durch die Spule	Ja	Nein	1	2	3	4	1	2	3	4
Angst/Nervosität	Ja	Nein	1	2	3	4	1	2	3	4
Konzentrationsschwierigkeiten	Ja	Nein	1	2	3	4	1	2	3	4
Gedächtnisprobleme	Ja	Nein	1	2	3	4	1	2	3	4
Andere (bitte angeben):	Ja	Nein	1	2	3	4	1	2	3	4

(adaptiert vom TMSens_Q – Fragebogen; siehe Giustiniani et al., 2022)
