# Supplementary material for: rTMS for the treatment of psychiatric disorders: a review about training courses and materials and the presentation of the training materials of the German Society for Brain Stimulation in Psychiatry
Source: Front Psychiatry. 2025 Aug 8;16:1490039. doi: 10.3389/fpsyt.2025.1490039 (PMC12371536; doi:10.3389/fpsyt.2025.1490039)
Supplement: Supplementary file 1 [file SupplementaryFile1.zip › Handbook (English).pdf]

Repetitive Transcranial Magnetic Stimulation (rTMS)

A Handbook from the German Society

for Brain Stimulation in Psychiatry (DGHP; registered association)

Version: April 2025

Submitted by Christiane Licht, Andreas Reissmann, Katrin Sakreida, Wolfgang Strube und Ulrike Vogelmann

(DGHP working group „Clinical Use, Quality Assurance and Certification“)

in collaboration with the DGHP Board of Directors and Martin Schecklmann, Stefanie Dierkes-Möller, Roberto Goya-Maldonado, Bernhard Kis, Michael Landgrebe, Berthold Langguth, Tobias Hebel and Carlos Schönfeldt-Lecuona

This manual expresses the opinion of the authors

and does not claim to be exhaustive.

Abbreviations

- DGHP = German Society for Brain Stimulation in Psychiatry
- DLPFC = dorsolateral prefrontal cortex
- EEG = electroencephalography
- EMG = electromyography
- GCP = Good Clinical Practice
- GOÄ = German fee catalog for physicians
- MDK = German health insurance medical service
- MEP = motor evoked potential
- MRT = magnetic resonance imaging
- OPS = German operations and procedures code
- PEPP = German general fees in psychiatry and psychosomatics
- RMT = resting motor threshold
- AMT = active motor threshold
- SMA = supplementary motor area
- rTMS = repetitive transcranial magnetic stimulation
- TMS = transcranial magnetic stimulation
- TPJ = temporoparietal junction
- ZP = additional charges

Table of Contents

1. **Summary**
2. **Structural and general conditions**
3. **Indications and treatment protocols**

   Excursus: Theta-burst stimulation (TBS)

   Excursus: acceleration of the treatment

   Excursus: maintenance rTMS

   Excursus: indications for rTMS beyond depression

   Table: indications for rTMS in psychiatry with graded levels of evidence
4. **Pre-treatment consultation, contraindications, side effects and special patient groups**

   4.1. Pre-treatment consultation

   4.2. Contraindications and special patient groups

   4.3. Side effects of rTMS treatment

   Excursus: risks for treatment providers
5. **Documentation and billing**
6. **Coil positioning and coil type**

   Excursus: EEG coordinate system
7. **Relevant coil positions**

   7.1. Motor cortex and determining the motor threshold

   7.2. Dorsolateral prefrontal cortex

   7.3. Temporoparietal junction

   7.4. Supplementary motor area
8. **Bibliography**

Appendix A: Suggested pre-treatment informed consent sheet

Appendix B: Suggested questionnaire for TMS side effects assessment

1. Summary

Repetitive transcranial magnetic stimulation (rTMS)…

- … is a non-invasive brain stimulation treatment - based on pulsed magnetic fields to initiate neuroplastic processes
- … is inherently a service of physicians, but can be delegated
- … can be performed in in-patient, out-patient, or day-care treatment settings
- … has a typical duration of treatment encompassing repeated sessions conducted on weekdays over several weeks
- … is preceded by the assessment of resting motor threshold (as a basis to determine stimulation intensity of the respective treatment)
- … can be used therapeutically of a variety of indications using a variety of different treatment protocols
  - high-frequency left DLPFC treatment for depression ("must be considered" recommendation for treatment resistant unipolar depression) and for negative symptoms in schizophrenia ("can be done" recommendation)
  - low-frequency left temporoparietal cortex stimulation for acoustic phantom perceptions and low-frequency supplementary motor area stimulation for obsessive-compulsive disorders ("can be done" recommendation)
- … has (potential) side effects
  - sensations at the stimulation area and noise (mitigation through wearing hearing protection)
  - headaches in rare cases
  - extremely rare: seizures or syncope
- … should eventually not be used in case of (relative) contraindications
  - hardly any absolute contraindication
  - individual risk-benefit assessment
  - electromagnetic implants and pre-existing neurological conditions warrant special and careful consideration

2. Structural and general conditions

Repetitive transcranial magnetic stimulation (rTMS) is a non-invasive brain stimulation method based on strong magnetic fields. It can be administered in in-patient, out-patient, or day-care settings. Treatment consists of repeated sessions conducted over a period of several days to weeks. Typically, stimulation is performed on weekdays; stimulation on weekends is not necessary. Prior to each treatment series, stimulation intensity is determined based on the resting motor threshold (RMT), which serves as the reference for defining individual treatment intensity.
Although rTMS is inherently a medical procedure, it can be delegated to trained non-medical staff. Conditions for implementation and billing are outlined in agreements governing the delegation of medical services to non-medical personnel in out-patient contract medical care. These agreements operate in accordance with Paragraph 1, Sentence 3 of the German Social Code Book V (SGB V). Additionally, guidelines are derived from the manuals provided by device manufacturers, national treatment guidelines, expert consensus articles, and opinions from ethics committees pertinent to relevant studies. It is crucial to ensure compliance with good clinical practice (GCP) standards and to adhere to recommendations from specialist medical societies. The German operations and procedures code (OPS; German: Operationen- und Procedurenschlüssel) for the classification of medical procedures and the German fee catalog for physicians (GOÄ; German Gebührenordnung für Ärzte) are also essential for medical documentation and billing.

In addition to this legal and regulatory frameworks, the following quality assurance measures are recommended in everyday clinical practice:

- regular staff training (at least once a year)
- standardization of procedures
- an emergency plan for dealing with complications such as syncope or induced seizures
- case discussions
- standardized rules for documentation and billing
- compliance with medical confidentiality and data protection

3. Indications and treatment protocols

There are various approaches to evaluating the evidence for specific rTMS protocols in different disorders. Lefaucheur and colleagues provided a comprehensive overview of the current evidence base for the clinical use of rTMS in psychiatric and neurological indications. In this work, international and European experts evaluated the study landscape for each indication based on defined quality levels (Lefaucheur et al., 2014, 2020). The following classes of evidence were defined:

a) "*Definitely effective protocols*" at least two studies of Class I (blinded randomized placebo-controlled clinical trial; n≥25); or at least one Class I study and two Class II studies (same design, but n<25).

b) "*Probably effective protocols*" = two convincing Class II studies, or one study Class II study and two Class III studies (other controlled studies).

c) "*Possibly effective protocols*" = one Class II study or two Class III studies.

In evidence-based medicine, meta-analyses of randomized placebo-controlled trials represent the highest quality level. German clinical guidelines are generally based on the availability of such meta-analyses, but they also take ethical considerations, clinical relevance, and feasibility into account. "High" corresponds to a strong positive recommendation, "moderate" to a weaker positive recommendation and "can" to an open recommendation. The table at the end of this section lists S3 guidelines (https://www.awmf.org/leitlinien) exclusively for the following conditions (unipolar/ bipolar depression, schizophrenia, chronic tinnitus, obsessive-compulsive disorders, smoking and nicotine dependence, and post-traumatic stress disorder). S3 is the highest national medical guideline recommendation according to the Association of the Scientific Medical Societies in Germany (AWMF). In addition, specific rTMS devices are approved for use in certain disorders, either through the FDA in the U.S. or the CE certification in Europe, based on criteria such as safety and efficacy. These approvals are documented in the respective manufacturer’s materials. The table at the end of this section provides an overview of the evidence base for rTMS in psychiatric disorders. Disorders not listed were excluded because no reliable conclusions can yet be drawn due to an insufficient number of studies. This does not imply a lack of efficacy, but rather a lack of data at this point.

rTMS is used across various psychiatric conditions – most commonly, and with the strongest and clearest evidence, in **depression**. In German-speaking countries, depression treatment is typically performed using a high-frequency protocol applied with a figure-8 coil over the left dorsolateral prefrontal cortex (DLPFC). A German expert consensus article recommends the following parameters for this indication (Hebel et al., 2022): a **frequency** of 10Hz or 20Hz, a **stimulation intensity** of 100-120% relative to the individually measured resting motor threshold (RMT), a **number of pulses per individual session** ranging from 1500 to 3000 pulses per session and a **treatment duration** of 15-30 sessions (corresponding to 3-6 weeks of weekday sessions). These recommendations align with the German national treatment guideline (NVL, Nationale Versorgungsleitlinie), which classifies rTMS as a second-line or third-line intervention. The same protocol is also recommended for treatment-resistant depression (TRD), particularly in combination with psychotherapy, according to more recent findings.

- 7-16 (new): "For patients who do not respond to monotherapy with antidepressants, augmentation with repetitive transcranial magnetic stimulation (rTMS) can be considered."
- 7-29 (modified): "Repetitive transcranial magnetic stimulation (rTMS) should be considered for treatment-resistant depressive episodes."
- 7-30 (new): "The selection of the rTMS method (stimulation location and type) should be done by a specialized center."

The European guideline (Lefaucheur et al., 2014; 2020) also considers right-frontal low-frequency stimulation or bilateral stimulation, including theta-burst protocols (iTBS or cTBS), as probably effective. These alternatives may be equally effective as the standard high-frequency protocol and are particularly relevant in cases of poor tolerability or neurological contraindications. The protocols vary widely (e.g., 120–1600 pulses per session, 10–20 sessions), though the overall stimulation intensity is comparable. What stands out is their shorter duration and lower number of pulses per session.

In the S3 guideline on bipolar disorder, the same high-frequency DLPFC protocol is recommended as a potential option for treating bipolar depression (especially in bipolar II disorder), although the evidence base is weaker due to fewer publications. The potential risk of switching into hypomania or mania should be considered in the risk-benefit assessment and

discussed during the informed consent process. Overall, the treatment effects appear to be independent of age.

Excursus – Theta-Burst Stimulation (TBS)

In contrast to conventional rTMS, theta-burst stimulation (TBS) is based on short, high-frequency pulse bursts administered in a rhythmic pattern that mimics the brain's natural theta frequency (~5 Hz) (Huang et al., 2005). The stimulation is delivered in so-called bursts, each consisting of three TMS pulses at 50 Hz. These bursts are then repeated at a theta frequency of 5 Hz.

The two main forms of TBS are intermittent TBS (iTBS) and continuous TBS (cTBS). In iTBS, bursts are delivered in trains lasting approximately two seconds (on-phase), followed by short breaks of around eight seconds (off-phase). These breaks are assumed to promote excitatory neuroplastic effects. In contrast, cTBS is administered as a continuous series of bursts without breaks, typically over a period of 40 seconds. This uninterrupted delivery leads to inhibitory effects on the neuronal activity of the targeted brain area and is therefore used, for example, to suppress overactive networks in persistent auditory hallucinations. The application of cTBS to the right DLPFC requires particular consideration, as the inhibitory effect is intended to generate an antidepressant effect based on the lateralization hypothesis of depression.

TBS protocols are often considered advantageous due to their significantly shorter treatment times. While conventional rTMS sessions last between 17 and 40 minutes, TBS can typically be administered in just a few minutes. Recent studies raise the question of whether shorter session durations might also result in better tolerability, possibly due to the use of lower stimulation intensities, typically around 80% of the resting motor threshold (RMT).

Following the successful evaluation of TBS for depression—showing at least equivalent antidepressant effects compared to conventional rTMS (Blumberger et al., 2018)—this stimulation modality has gained growing international popularity. In a pivotal non-inferiority trial, iTBS at 120% RMT was found not inferior to standard 10 Hz rTMS and led to similarly robust reductions in depressive symptoms.

The application of intermittent TBS (iTBS) using the 600-pulse standard protocol enables a 6–7 fold reduction in daily treatment duration. Compared to conventional rTMS, iTBS is typically applied at a slightly lower intensity over the same number of treatment days (Kishi et al.,

2024). The ideal number of pulses per treatment session (600, 1200, or 1800) remains a topic of ongoing debate. During the COVID-19 pandemic, the method gained popularity due to its time efficiency, and it continues to be of high interest in resource-limited clinical environments, given its considerable time-saving potential (e.g., 3.5 minutes vs. ~20 minutes per session).

Excursus: acceleration of the treatment

In recent years, the concept of accelerating antidepressant TMS protocols has been increasingly discussed in the scientific community. The idea is to intensify rTMS/TBS treatment by delivering multiple sessions per day (accelerated TMS, aTMS), aiming to achieve a faster therapeutic response and shorten the total treatment duration.

The interest in aTMS has been particularly stimulated by the publication of the SAINT/SNT protocol (Stanford Accelerated Intelligent Neuromodulation Therapy), which demonstrated strong antidepressant effects in initial studies (Cole et al., 2020; Cole et al., 2022). However, it remains unclear which specific components of the SAINT/SNT protocol are responsible for its effectiveness—whether it is the use of neuronavigation (based on functional anticorrelation with the subgenual anterior cingulate cortex), the high number of 10 daily sessions, or the application of 1800 pulses of prolonged iTBS per session (resulting in a total of 90,000 pulses over five days). Due to the intensive resource requirements, implementation of the SAINT/SNT protocol in routine clinical settings seems feasible only in highly specialized centers.

Nevertheless, a recent meta-analysis (Cai et al., 2023) summarized the available evidence for aTMS protocols and concluded that accelerated treatment approaches, particularly accelerated iTBS (aiTBS), show promising antidepressant efficacy beyond the SAINT protocol. For clinical use, more flexible and feasible adaptations of these accelerated protocols are recommended—e.g., protocols with 4–5 sessions per day, which are considerably easier to implement.

For example, Duprat et al. (2016) demonstrated that a four-day aTMS protocol using five daily iTBS sessions (1620 pulses per session, over the left DLPFC, with 15-minute intersession intervals) produced positive antidepressant effects, which in some cases emerged fully only with a delay of 1–2 weeks following treatment completion.

When planning accelerated protocols, it is critical to observe the intersession interval between treatments administered on the same day. A minimum pause of 15 minutes between sessions is necessary, but ideally around 50 minutes should be allowed (see Cai et al., 2023). If this is not taken into account, the intended accumulation of neuroplastic TMS effects may not occur, and the outcome of multiple sessions per day may potentially not differ from that of a single session. Due to the possibility of delayed therapeutic effects, this should be addressed during patient education about accelerated TMS (aTMS), and ideally, follow-up appointments for state assessment (e.g., 2–4 weeks after the end of treatment) should be scheduled.

Any deviation from standard protocols must be clearly justified, particularly because less is known about the safety and tolerability of intensified approaches. Careful safety management and substantial clinical experience with TMS are required when implementing aTMS. Overall, further research is needed to clarify the safety and efficacy of accelerated protocols.

Excursus: maintenance rTMS

There are hardly any controlled studies on relapse prevention in depression therapy and on maintenance treatment with rTMS in patients with a positive initial response (Baeken et al., 2019; Chang et al., 2020; Haesebaert et al., 2018; Wilson et al., 2022). **Based on clinical experience**, it has been shown that a positive response to rTMS treatment predicts future response to renewed treatment. Thus, as a general recommendation, patients who responded to rTMS can be advised to undergo rTMS treatment again in the event of a new depressive episode.

Regarding ongoing maintenance treatment with rTMS following a successful antidepressant rTMS series, the current evidence—although weak due to being based primarily on open-label studies—is nonetheless predominantly positive. The findings clearly indicate that maintenance rTMS is safe and effective. However, no standardized protocol or treatment scheme has yet been widely studied or published. A recent systematic review by d'Andrea et al. (2023) showed that maintenance effects were not achieved with too few rTMS sessions (two or fewer per month). In addition, available studies cautiously suggest that a time interval of up to four weeks between the end of the acute treatment phase and the start of maintenance rTMS may be possible without increasing the risk of relapse (d'Andrea et al.,

2023). This consideration should also be taken into account when planning a suitable maintenance rTMS protocol, especially with respect to available resources.

From a clinical perspective, several variants of maintenance treatment with rTMS are conceivable for patients with recurrent depressive episodes:

- Variant (a): Gradual tapering of rTMS sessions over the course of several weeks. The number of sessions per week is progressively reduced according to a reduction scheme. This form of maintenance treatment usually begins directly after the acute treatment phase, typically starting with 1 or 2 sessions per week.
- Variant (b): Use of booster treatment weeks (i.e., one week of daily rTMS sessions) with intervals of several weeks between them (initially 3–6 weeks), possibly with gradual extension of the treatment-free intervals over time.

It is recommended to discuss the patient's recurrent depressive symptoms in each individual case and to plan and adjust maintenance rTMS accordingly on a case-by-case basis.

Excursus: rTMS Indications Beyond Depression

The **negative symptoms of schizophrenia** are treated in a similar manner to depression. However, the level of evidence is lower, so that only a "can" recommendation and "possibly effective" classification can be made. This corresponds to the findings reported in published meta-analyses, which are based on a relatively small number of studies. In addition, the number of available meta-analyses is low, some are relatively old, and they do not exclusively focus on rTMS for negative symptoms (Aleman et al., 2018; Dougall et al., 2015; Hyde et al., 2022; Osoegawa et al., 2018; Tseng et al., 2022). The German S3 guideline emphasizes that patients must be informed about the expected low response rates.

The assessment of the efficacy of low-frequency rTMS for treatment-resistant **auditory hallucinations in schizophrenia** is somewhat more favorable in consensus-based guidelines, although negative meta-analyses have also been presented in this context (He et al., 2017). According to the current S3 guideline, rTMS "should" be administered in the presence of treatment-resistant auditory hallucinations in the context of a schizophrenic psychosis, since—despite the heterogeneity of the findings—a slight positive therapeutic effect is considered probable. In summary, in the case of treatment resistance, a trial of low-frequency

(inhibitory) 1 Hz rTMS over the left temporoparietal junction cortex (TPJ) is recommended (stimulation intensity: 80–100% RMT, 10 sessions, 1000 or 1200 pulses).

In the case of **chronic tinnitus**, the current S3 guideline explicitly does not recommend treatment with rTMS, even though a number of positive meta-analyses have been published and the European guideline considers rTMS possibly effective (Lefaucheur et al., 2020; Lefaucheur et al., 2014). Treatment follows the same approach as for auditory hallucinations.

For **obsessive-compulsive disorder**, the S3 guideline states that rTMS treatment may be considered in patients who do not respond sufficiently to first-line treatments (recommendation grade 0). A range of different stimulation protocols is available (Fitzsimmons et al., 2022), targeting different brain regions (dorsolateral prefrontal cortex, supplementary motor area, medial prefrontal cortex/anterior cingulate cortex, orbitofrontal cortex). Despite the existence of approvals (e.g., FDA-approved deep rTMS with H1 coil, NeuroStar system) and guideline recommendations, the evidence remains heterogeneous. In particular, systematic studies differentiating between syndromal subtypes (e.g., primarily obsessive thoughts vs. compulsive actions) are lacking, so that no clear preference for a specific protocol can currently be expressed. A more recent development is the use of deep rTMS, which enables greater penetration depth through special coil geometries (target area: medial PFC, anterior cingulate cortex). The studies leading to FDA approval combined the typical six-week deep rTMS protocol with a behavioral exposure intervention, which should be considered when planning and designing this form of treatment.

For **substance use disorders and craving**, the evidence is very heterogeneous. Guidelines and approvals rarely align. Based on neurobiological findings, rTMS over the left dlPFC (high-frequency stimulation protocol) is recommended, with the aim of strengthening cognitive control networks and reducing craving (Gay et al., 2022; Zhang et al., 2019). The choice of stimulation protocol could depend on the specific substance dependence and treatment goal, and—given the need for further research and ongoing studies in this area—a current literature review should be conducted before making protocol decisions.

For **post-traumatic stress disorder**, high-frequency stimulation of the right DLPFC is probably effective, although only short-term treatment effects have been demonstrated so far for different TMS treatment approaches (Liu et al., 2024). However, it should be noted that TMS is not mentioned in the guidelines and no approvals exist.

The table on the following page summarizes the current evidence and the most commonly used rTMS protocols for the treatment of the psychiatric disorders discussed.

Table: Psychiatric indications of rTMS, prominent protocols and levels of evidence

	Behandlungsprotokoll	Deutsche Leitlinien	Europä-ische Leitlinie[a]	Zulas-sungen	Studienlage
Unipolar / Bipolar Depression	F3, 10/20 Hz, 100–120%, 1500–3000 pulses, 20–30 sessions (or F3, iTBS, 80%, 600 pulses, 20–30 sessions) (Hebel et al., 2022)	"Should" in treatment resistance "Can" after failed monotherapy and in bipolar depression	definitely effective	FDA, CE	Numerous positive meta-analyses available
Negative Symptoms in Schizophrenia	Protocol analogous to depression (Lefaucheur et al., 2014; 2020)	"Can" in treatment resistance<br><br>Inform about low expected response	possibly effective	None	Further evidence required
Auditory Hallucinations in Schizophrenia	CP5, 1 Hz, 80–100%, 1000/1200 pulses, 10 sessions (or CP5 & CP6, cTBS, 80%, 600 pulses each, 15–20 sessions) (Lefaucheur et al., 2014; 2020; Plewnia et al., 2018; Ye et al., 2024)	"Should" in treatment resistance as part of an overall treatment plan	possibly effective	None	Further evidence required
Chronic Tinnitus	CP5, 1 Hz, 110%, 2000 pulses, 10 sessions (Folmer et al., 2015; Lefaucheur et al., 2014; 2020)	"Should not" be used (DGPPN dissent: "may be considered")	possibly effective	None	Further evidence required
Obsessive-Compulsive Disorder	SMA, 1 Hz, 100% (leg), ≥1200 pulses, 15–30 sessions, possibly with angled or double-cone coil or F4, 1 Hz, 110%, ≥1200 pulses, 15–30 sessions (Fitzsimmons et al., 2022)	"Can" for short-term symptom relief in treatment resistance	possibly effective	FDA, CE	Protocols inconsistent; further evidence required
Addiction / Craving	Protocol analogous to depression (Lefaucheur et al., 2014; 2020)	No recommendation possible	possibly effective for nicotine	FDA, CE (substa nce-specific)	Further evidence required
Post-Traumatic Stress Disorder (PTSD)	F4, 1–20 Hz, 80–120%, 100–4000 pulses, 10–30 sessions (McGirr et al., 2021)	Not mentioned in guidelines	probably effective	None	Further evidence required

Notes: F3-corresponds to the left dlPFC (10-20 EEG-system coordinate), F4-corresponds to the right dlPFC (10-20 EEG-system coordinate), CP5/CP6-corresponds to the left/right temporoparietal junction (TPJ, 10-20 EEG-system coordinate, SMA-supplementary-motor area; [a] refers to Lefaucheur et al. (2014; 2020)

4. Pre-treatment consultation, contraindications, side effects and special patient groups

4.1. Contents of the pre-treatment consultation

The following points are relevant for pre-treatment consultation (non-exhaustive) and informed consent:

- verbal consultation via physicians
- documentation of the consultation
- allowing time to consider (depending on the complexity of the case)
- information about possible treatment alternatives in addition to treatment efficacy, procedures, risks, and side effects
- indication should be determined by specialized physicians (psychiatrist/neurologist with qualifications in brain stimulation procedures as per training regulations or medical societies such as DGPPN and/or DGHP)
- in the absence of FDA/CE approval, there should be documentation of off-label use
- in the case of depression with comorbid disorders, the emphasis should be on depression as an indication, and treatment should follow a depression protocol

It is recommended to use an informed consent sheet. A sample text is provided in the appendix.

As a formulation aid for medical record documentation, the following text passage can be used:

"The patient sought rTMS treatment following prior diagnosis of depression with inadequate improvement despite current multimodal therapy. The patient received comprehensive information, both verbally and written, about the treatment's indication, effects and side effects. There are no contraindications. The primary objective of the treatment is to alleviate depressive symptoms… Time to consider…"

4.2. Contraindications and special patient groups

The indication for the treatment of depression in the presence of comorbidities (e.g., a history of epileptic seizures) or under special clinical circumstances (e.g., pregnancy) should be made on an individual basis after weighing the benefits and risks, and requires especially careful medical counseling. The increased relative risk of epileptic seizures should be taken into account in the presence of neurological conditions. Most contraindications are relative. Regarding contraindications, reference should also be made to the international expert recommendations, in particular those of the International Federation of Clinical Neurophysiology (IFCN) (Rossi et al., 2009; Rossi et al., 2021).

- **Implants, Tattoos, Piercings**

Ferromagnetic devices or implants that rely on the detection or generation of electrical pulses for their function (a classic example: pacemakers) can be contraindications for rTMS. MRI compatibility of an implant can provide a hint as to whether rTMS is feasible, but it does not guarantee safety. Therefore, only an individual risk assessment is possible, considering factors such as device specifications (material, mechanism of action), the distance to the stimulation site, and coil geometry. When weighing risks and benefits, the urgency of the indication must of course also be considered (rTMS is rarely urgent or vital, unlike ECT). Metal-containing tattoos in the stimulation area also represent a contraindication—unlike dental implants or piercings. In the latter case, it is recommended to remove them before rTMS treatment depending on the material.

- **Pre-existing brain injury and neurological conditions**

“Pre-existing brain injury” is a broad umbrella term that does not, in itself, constitute an absolute contraindication. It is important to assess whether the individual lesion, in its extent and location, may promote epileptic seizures and/or impair accurate target identification (e.g., severe atrophy, left frontal processes). In such cases, an individual risk-benefit analysis should be conducted, taking therapy alternatives into account, and the risk of complications should be further specified through appropriate diagnostics (e.g., EEG). In these cases, patient information and consent should be documented particularly thoroughly in the medical record.

Likewise, in known neurological preconditions, it should be evaluated whether the available scientific evidence supports the use of rTMS protocols (e.g., in the treatment of depression). Although comprehensive findings are lacking, the efficacy of rTMS protocols for depression may be reduced in certain neurological conditions such as Parkinson's disease (Liu et al., 2014; Zhang et al., 2022).

- **Pregnancy**

Robust scientific evidence is now available and can be discussed with the patient to support shared decision-making (Hebel, Schecklmann, & Langguth, 2020; Hızlı Sayar et al., 2014; D. R. Kim et al., 2019). At least two prospective studies and numerous case reports show that rTMS can generally be administered during pregnancy without harm to mother or child. However, due to the overall small number of cases, rare side effects cannot be reliably ruled out. In this patient group, particular attention should be paid to the benefit-risk assessment and the patient's preference. Informed consent should be documented with particular care in the medical record. During this counseling, explicit reference should also be made to the possibility of harm to the child in the event of a TMS-induced seizure.

- **Co-medication**

Due to the wide range of potential substances and interactions, this topic cannot be exhaustively addressed here. For an overview, see Hebel, Abdelnaim et al. (2020) and Deppe et al. (2021). Primarily discussed are attenuating effects on treatment success from benzodiazepines, antipsychotics, and anticonvulsants. On the other hand, beneficial effects have been reported when combined with SSRIs (Zaidi et al., 2024) or psychostimulants (Hunter et al., 2019).

4.3 Side Effects of rTMS Treatment

- **Local Reactions**

Unpleasant sensations at the stimulation site and mild to moderate headaches are the most common side effects and primarily occur at the beginning of treatment. In clinical experience, treatment discontinuations due to these effects are rare but do occur. Tolerance and coping

with headaches vary greatly in clinical practice depending on the individual predisposition and overall psychological state of the patient.

- **Hearing Damage and Electromagnetic Field Exposure**

All patients are inherently exposed to the loud clicking sound and the electromagnetic field generated by the TMS pulse. For these two fundamental aspects of exposure, there are still few scientific data available (C. Schönfeldt-Lecuona et al., 2012).

Findings from individual studies and basic risk considerations suggest that patients should be advised to wear hearing protection (earplugs). There is a lack of robust long-term studies on the relevance of long-term exposure to the electromagnetic fields generated by rTMS.

- **Risk of manic switch and suicidality**

A switch into a manic phase (even in the absence of a prior diagnosis of bipolar disorder), as well as the occurrence or worsening of suicidal thoughts, can in principle occur in any patient with depression, during therapy, or in the context of ineffective treatment. For this reason, the indication should be established by a specialist in psychiatry, and a final interview should be conducted by a qualified person. However, these are not risks specific to rTMS.

- **Cognitive side effects**

It is often the case that patients inquire about potential cognitive side effects. According to current knowledge, there is no evidence of such side effects (Patel et al., 2020; Rossi et al., 2009). It must be noted that "cognition" is a broad term, and cognitive abilities—both in self-perception and third-party assessment—are significantly influenced by the underlying illness being treated, most commonly depression.

- **Epileptic seizure and syncope**

The literature shows that while the induction of a seizure by rTMS is possible, it is highly unlikely (< 2 in 100,000 treatments; Shafi, 2019). The stimulation protocol, individual seizure susceptibility, and the presence of additional provoking factors all play a role, as does the use of anti- or proconvulsant medications. In general, staff present should be trained to recognize and manage seizures (e.g., immediate discontinuation of stimulation). During the informed

consent process, patients should be informed not only of the possibility of such an event, but also of potential consequences.

Presyncopal or syncopal events occur more frequently during rTMS treatment—especially during initial sessions for motor threshold determination, morning treatments, and protocols involving prolonged lying or reclined positions followed by rapid standing.

Excursus: Risks for rTMS Operators

For the sake of completeness, it should be mentioned that the issues of implants, pregnancy, and acoustic/electromagnetic exposure are in principle also relevant for rTMS operators. However, there is a near-complete lack of reliable data on this topic. rTMS-specific risks should be addressed within occupational risk assessments. Current recommendations for operator distance vary: 70 cm (ICNIRP; Rossi et al., 2009), 24 cm (ICNIRP; Rutherford et al., 2020), or 40 cm (Rossi et al., 2021).

5. Documentation and billing

Since the inclusion of rTMS in the German PEPP fee catalog in 2021, specific additional fees (ZP75) for basic services (OPS 8-632.0) or therapy sessions (OPS 8-632.1) have been available for in-patient and day-care treatments. Billing arrangements for out-patient care in Germany remain inconsistent. When documenting and providing cost estimates, it is important to mention therapy resistance, the severity of depression, and the necessity of TMS.

- **In-patient care: additional charges**

The basic rTMS service (ZP75.01, OPS: 8-632.0, EUR 124.17, as of 2024) and the rTMS therapy session (ZP75.02, OPS: 8-632.1; EUR 74.54, as of 2024) are billed and documented. It should be noted that the physician documents and bills the pre-treatment consultation separately. The billed time must be under 25 minutes. The rTMS basic service includes consultation, motor threshold assessment, and the first treatment. The basic service must always be coded on the first day of treatment. Documentation and coding are done on same the day the service takes place. The rTMS therapy service is the individual treatment session. This may take place several times a day. As these additional charges are new, German health insurance medical service (MDK) inspections over time will reveal further aspects to consider.

- **Privately insured out-patients and self-Payers: German fee catalog for physicians**

Submitting a cost estimate or treatment contract is advisable. Additionally, providing supporting evidence and addressing therapy resistance or the absence of treatment response in the patient’s case is recommended. Billing is done in accordance with the German fee catalog for physicians (GOÄ). In particular, the codes 839a motor threshold (electromyographic examination; 93.84 euros) and 828 rTMS (evoked potentials; 81.11 euros) are used. Additionally, depending on the service provision and documentation, codes 801 (psychiatric examination), 804 or 806 (psychiatric treatment), 860 (biographical case history), 1 or 3 (pre-treatment consultation), 865 (case discussion) and 857 (psychological tests) are possible.

6. Coil positioning and coil type

Correct positioning of the magnetic coil over the target area, as well as its orientation and tilt, is of central importance in rTMS treatment. It must be ensured that (a) the center of the coil is positioned as precisely as possible over the intended target area and that (b) the coil orientation is correct. The coil should lie tangentially on the head, with its center forming the contact point with the scalp. Attention should also be paid to a relaxed or semi-recumbent position of the patient and to verifying correct coil positioning throughout the course of treatment. Moreover, care should be taken not to apply excessive pressure to the patient's head when positioning the coil in order to prevent discomfort or injury.

Some coil types are less spatially precise or stimulate larger cortical areas compared to the standard figure-8 coil. These include the round coil and coils designed for deeper stimulation, such as the angled figure-8 coil or helmet-style coils. The recommendations in this manual are based on the use of standard figure-8 coils, which are the most common in the German-speaking clinical context. For the use of deep coils or helmet systems, we recommend consulting the respective manufacturer’s manuals.

In general, there are two options to determine a target area for rTMS. Firstly, there is **anatomy-based neuronavigation** using structural MRI. This method requires individual MRI scans prior to treatment and a neuronavigation-compatible TMS system. It is the most precise technique and enables high spatial reproducibility across sessions (Carlos Schönfeldt-Lecuona et al., 2005; C. Schönfeldt-Lecuona et al., 2010). However, it is also resource-intensive and not mandatory for clinical application. To date, there is insufficient literature addressing the necessity of neuronavigation in routine psychiatric treatment settings (Herwig, Padberg, et al., 2001; Herwig, Schönfeldt-Lecuona, et al., 2001). **Scalp-based localization** based on external landmarks and EEG reference points (Herwig et al., 2003). This is currently the most common method. It involves identifying standard anatomical reference points on the skull and using caps with visible markers, flexible measuring tapes, or protractors. For hygiene reasons, each patient receives a new cap. When stimulation is repeated (e.g., during a treatment course), it is important that the cap is placed in the same way each time. This should be based on skull markings. As a guide, the cap seam can be placed always at the same distance from the nasion and verification of the EEG position Cz can be helpful. It is helpful to draw the midline/sagittal line, which should be centered above the nose when looking from the front. Marking the cap

in front of the ears can also be helpful. In clinical practice, it makes sense to note relevant information about cap positioning (e.g., nasion-cap seam distance) not only on the treatment protocol but also on the cap itself.

Excursus: EEG coordinates

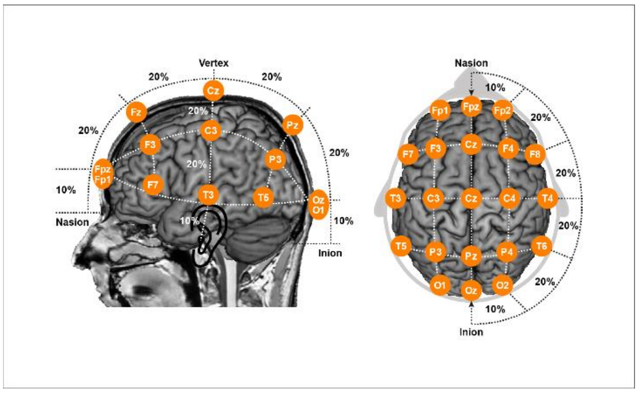

The 10-20-electrode coordinate system allows for the standardized recording of head surface positions regardless of head size. The electrode positions roughly correspond to cortical regions located under the skull (Koessler et al., 2009). Based on fixed anatomical locations of the skull (nasion, inion and preauricular point), crescent-shaped lines are formed across the skull, which in turn are divided into 10% or 20% sections. The electrode positions are named according to their position on the skull (T=temporal, P=parietal, F=frontal, O=occipital, C=central). Left-sided electrode positions are odd-numbered, and right-sided positions are marked with even numbers.

FPz is the anterior midpoint in the EEG system, which is defined as 1/10 of the nasion-inion distance. Cz (position above the vertex) represents the intersection of the interaural line and the midline. Patients can also be asked to point to the highest point of their head with their finger, which should correspond to Cz. The point Oz is 10% above the inion. The inion is the bony hump in the midline of the back of the head at the attachment of the neck muscles. The nasion is the furrow above the root of the nose.

During longer treatment sessions (exception: motor threshold determination), the patient is seated or reclined in a treatment chair with the head supported. This minimizes head movements and ensures stable coil positioning. Patients should avoid moving their heads during stimulation. If repositioning is necessary, patients should inform the staff beforehand so that stimulation can be paused and the coil realigned to the correct location. Minimizing unintentional head movements and ensuring the most relaxed positioning of the patient can be achieved through the use of (vacuum) cushions for head stabilization.

7. Relevant coil positions

Instead of manually measuring each mark, it is also possible to use EEG cap shells with pre-punched standard EEG positions, which can be ordered from EEG equipment suppliers. These shells are placed over the treatment cap and allow for reproducible markings of various EEG points.

Another recommendation is to mark the coil handle position on the cap. Many TMS coils obscure the direct view of the stimulation center, so marking the coil edge or handle on the cap can help ensure consistent placement. This involves measuring and marking the distance from the center of the coil to its edge (the point of contact on the cap), which is usually consistent for standard coils. Note that this distance should be measured on a flat plane, while the human head is curved.

Often, publications report the direction of the coil handle. However, without the associated current flow direction, this information is insufficient. As this is often not explicitly stated, we recommend using the default current direction settings provided by the manufacturer.

Different disorders require targeting different cortical areas. The following section describes the coil positioning for the most common evidence-based rTMS treatments. We begin by outlining the coil positioning and procedure for motor threshold determination over the motor cortex.

7.1. Motor cortex and determining the motor threshold

The following procedure refers to the determination of the motor threshold in the context of a planned antidepressant rTMS treatment over the left dorsolateral prefrontal cortex. Therefore, the motor threshold is determined by stimulating the left motor cortex, which controls muscles on the right side of the body due to contralateral innervation. This is typically done using a muscle of the right hand, meaning that the hand area ("hand knob") of the left hemisphere is targeted with TMS pulses. As an anatomical approximation of the left hand area, the EEG position C3 can be used. Anatomically, the hand knob often appears as a dorsally protruding hook in the precentral gyrus. Besides its role in motor threshold determination, this area is occasionally relevant for the treatment of certain pain syndromes. C3 lies halfway between Cz and T3/T7 (10% above the preauricular point), or about 20% from the sagittal midline along the interaural line toward the left ear.

Determination of the motor threshold

Motor threshold determination begins with functional identification of the motor hotspot. That is, the coil position over the scalp is identified—through iterative testing—where a single TMS pulse reliably elicits a maximum and reliable response in the target muscle. For motor threshold determination in the context of left-frontal antidepressant rTMS, C3 (H. Kim et al., 2023) serves merely as a starting point. A coarse approximation of this point may suffice at first, for example by measuring two to three finger widths laterally from Cz along the interaural line depending on finger width. Crucial is the coil orientation, which should be at a 45° angle to the sagittal plane. Typically, the handle of the TMS coil points posteriorly. To simplify this orientation, a flexible protractor can be used to draw a 45° reference line relative to the sagittal plane that represents the extension of the coil and handle. Alternatively, the 45° angle can also be defined by taking two to three finger widths anterior from Cz along the nasion-inion line and connecting this point to the approximated C3 location.

The motor threshold determined over one hemisphere is a measure of cortical excitability for that hemisphere (for bilateral stimulation, the threshold may need to be determined for both hemispheres) and serves as the basis for defining individual stimulation intensity. The motor threshold must be measured at the beginning of every treatment. It generally remains stable over the course of treatment (Nordmann et al., 2015) and thus usually only needs to be determined once. Monitoring of psychotropic medication such as benzodiazepines or antipsychotics is recommended, as these can affect the motor threshold or treatment outcome (Deppe et al., 2021; Hebel, Abdelnaim, et al., 2020; Ziemann et al., 2015).

Ideally, an electromyogram (EMG) and surface electrodes are used to objectively measure muscle activity. The target parameter is the motor evoked potential (MEP), a biphasic waveform visible in the EMG approximately 20–40 ms after the TMS pulse. If EMG is not available, visible muscle twitches can be used instead. It is important to note that visually determined thresholds are about 10% higher than EMG-based thresholds (Westin et al., 2014). Therefore, we recommend roughly reducing the treatment intensity accordingly when the motor threshold is determined without EMG (e.g., using 100% of the visually determined threshold instead of 110% RMT).

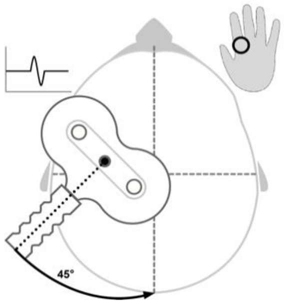

To determine the motor threshold, a two-stage **approach** is followed:

a) **Determining the motor hotspot**: The patient should sit comfortably and as relaxed as possible in the examination chair. The right hand should rest loosely on the armrest, ensuring that the muscles are not pre-activated. If tension is present, the resulting value may reflect the active motor threshold (AMT), which is typically not relevant in this clinical context (see section below). Muscle activity is recorded via surface electrodes placed on the right hand. Stimulation occurs on the left side of the head (due to contralateral organization of the motor system). Commonly used muscles include ADM, APB, or FDI. The coil should be positioned at a 45° angle to the midline, with the handle pointing posteriorly, over the previously approximated starting point for hotspot localization. Using clearly suprathreshold stimulation intensity (pragmatically 55–60% of stimulator output), the coil is moved systematically over the cortical area in longitudinal and sagittal directions to find the spot that evokes the strongest and most reliable muscle response. Alternatively, a grid can be drawn and each point tested.

b) **Actual determination of the motor threshold:** Once the motor hotspot is found, the coil position is marked on the cap by outlining notable features of the coil (e.g., the junction of the figure-8 windings). Then, a threshold procedure is conducted in which stimulation intensity is adjusted until the threshold is found. Threshold means that a response exceeding the criterion (visible twitch or MEP > 50 µV) occurs in 50% of trials. There are several options:

   - Standard algorithmic approaches (e.g., Rossini-Rothwell, Mills-Nithi), which involve increasing/decreasing intensity until the threshold is reached. These methods are time-consuming and require many pulses. A pragmatic approach is to adjust the stimulation intensity until a motor response > 50 µV (or visible twitch) is seen in 4

out of 8 trials. Experienced personnel can typically identify the threshold within a few minutes.

- "Threshold hunting" (Awiszus, 2003) is an adaptive method derived from psychophysics that requires fewer pulses. With computer support, the threshold can be estimated via maximum likelihood procedures. Semi-automated software tools are recommended, as they allow evaluation of individual pulse responses and exclusion of invalid trials. There are also newer estimation methods that do not rely on strict mathematical assumptions (Wang et al., 2023). Tools can be found at www.clinicalresearcher.org (under “Software”, e.g., MTAT 2.1) or https://tms-samt.github.io.

Active Motor Threshold and Additional Considerations

The previous sections focused on the determination of the resting motor threshold (RMT). In clinical practice, the determination of the active motor threshold (AMT) is generally of limited relevance. However, it can serve as an *alternative measure of cortical excitability* in patients with pathologically increased muscle tone (e.g., tremor, spasticity, dystonia, or motor disturbances due to stroke or dementia). In such cases, reliable determination of the RMT may be difficult or impossible, making AMT a feasible option. AMT is also *frequently used in theta-burst stimulation* (TBS) protocols, where treatment intensity is more often based on AMT. Typically, AMT is lower than RMT in terms of the maximum stimulator output (%MSO), since corticospinal excitability is increased during slight muscle pre-activation.

The procedure for determining AMT is similar to that of RMT, except that the stimulation is performed while the target muscle is slightly pre-activated. The threshold can be determined using an algorithmic procedure, where the response criterion is either an MEP with an amplitude ≥200 µV or a visible muscle twitch. A critical note concerns the standardizability of AMT measurement: without objective aids such as a dynamometer to quantify the level of muscle activation, variation and poor reproducibility may result.

Additional practical considerations apply to the positioning and movement of the coil, especially during hotspot identification. Care must be taken *not to apply excessive pressure with the coil onto the head* to avoid pain or injury. For EMG-based threshold procedures, a team member should ideally have *sound knowledge of EMG measurement*,

including electrode placement, signal assessment, and interpretation of muscle responses. This includes the establishment of standardized skin preparation procedures (e.g., cleaning with alcohol wipes), the assessment of EMG signal quality at the beginning of threshold determination, as well as the identification of potentially abnormal EMG response patterns due to underlying pathologies (e.g., ulnar groove syndrome, peripheral neuropathy, or radiculopathy).

7.2. Dorsolateral prefrontal cortex

The dorsolateral prefrontal cortex (DLPFC) is the target stimulation area for treating depressive syndromes, negative symptoms in schizophrenia, addiction syndromes, and obsessive-compulsive disorders. Historically, the DLPFC was determined using the 5 cm rule and later the 6 cm rule, in which the stimulation point was determined 5 or 6 cm anterior to the hot spot of motor threshold determination and parallel to the midline (Herwig, Padberg, et al., 2001). The standard approach now involves referring to the F3 EEG point on the left side of the head and F4 on the right. A simple tool for determining this point is the Beam-F3 method, available as a download or as an online calculator (http://clinicalresearcher.org/F3/). The tragus-tragus and nasion-inion distance as well as the head circumference are used to determine how far the stimulation point is from the center of the forehead and from the vertex. The coil should be positioned at a measured 45-degree angle to the midline. Another helpful option is to orient the coil towards the nasion or FPz.

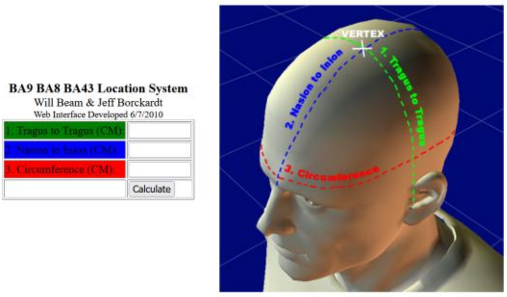

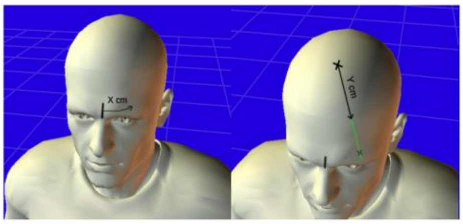

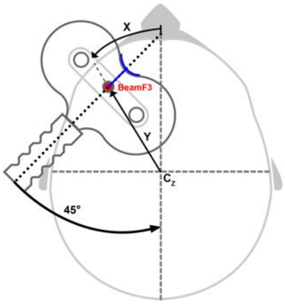

Anatomically, the aim is to stimulate the transition area of the Brodmann areas BA9 and BA46 or the border between the anterior and middle third of the middle frontal gyrus (Mylius et al., 2013). As already mentioned, there is no clear superiority of neuro-navigated rTMS treatment for depression (Fitzgerald et al., 2009). Many other studies were not done as controlled studies.

7.3. Temporoparietal cortex

The auditory cortex and the temporoparietal junction (TPJ) are the target areas for treating auditory hallucinations. The evidence for these protocols is limited. Nevertheless, therapy-resistant patients are found again and again, which make an individualized attempt for treatment worthwhile.

The position of the auditory cortex is marked analogous to tinnitus publications (Langguth et al., 2006). Firstly, starting at T3, we move 2.5 cm on a line towards C3. Secondly, from that position, we move 1.5 cm dorsally at a 90-degree angle. The coil should be applied in parallel to the T3-Cz line, with the coil handle pointing towards Cz.

The left TPJ is determined via CP5, the right via CP6. The coil handle points at a 45-degree angle backward and upward. CP5/CP6 is in the middle between T3/T4 and P3/P4. The points T3-CP5-P3 and T4-CP6-P4 are connected in order to specify the coil orientation (handle direction towards P3/P4). Two papers, that mistakenly use the term TP3 instead of CP5, were used to determine these positions originally (Herwig et al., 2003; Hoffman et al., 2003).

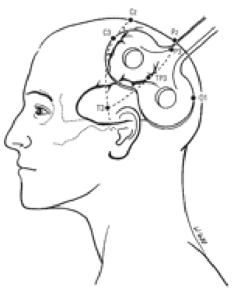

HOFFMAN ET AL, 2003

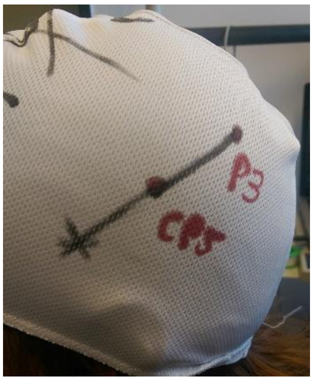

7.4. Supplementary Motor Cortex

To position the center of the coil, starting at Cz move 15% of the length of the nasion-inion distance forward on the midline. The coil handle should point backwards towards the inion. This positioning is commonly applied in the treatment of obsessive-compulsive disorder (Mantovani et al., 2005).

Bibliography

Aleman, A., Enriquez-Geppert, S., Knegtering, H., & Dlabac-de Lange, J. J. (2018). Moderate effects of noninvasive brain stimulation of the frontal cortex for improving negative symptoms in schizophrenia: Meta-analysis of controlled trials. Neuroscience & Biobehavioral Reviews, 89, 111–118. https://doi.org/10.1016/j.neubiorev.2018.02.009

Awiszus, F. (2003). TMS and threshold hunting. Supplements to Clinical Neurophysiology, 56, 13–23. https://doi.org/10.1016/s1567-424x(09)70205-3

Baeken, C., Brem, A.-K., Arns, M., Brunoni, A. R., Filipčić, I., Ganho-Ávila, A., Langguth, B., Padberg, F., Poulet, E., Rachid, F., Sack, A. T., Vanderhasselt, M.-A., & Bennabi, D. (2019). Repetitive transcranial magnetic stimulation treatment for depressive disorders. Current Opinion in Psychiatry, 32(5), 409–415. https://doi.org/10.1097/YCO.0000000000000533

Blumberger, D. M., Vila-Rodriguez, F., Thorpe, K. E., Feffer, K., Noda, Y., Giacobbe, P., Knyahnytska, Y., Kennedy, S. H., Lam, R. W., Daskalakis, Z. J., & Downar, J. (2018). Effectiveness of theta burst versus high-frequency repetitive transcranial magnetic stimulation in patients with depression (THREE-D): a randomised non-inferiority trial. The Lancet, 391(10131), 1683–1692. https://doi.org/10.1016/S0140-6736(18)30295-2

Cai, D.-B., Qin, Z.-J., Lan, X.-J., Liu, Q.-M., Qin, X.-D., Wang, J.-J., Goya-Maldonado, R., Huang, X.-B., Ungvari, G. S., Ng, C. H., Zheng, W., & Xiang, Y.-T. (2023). Accelerated intermittent theta burst stimulation for major depressive disorder or bipolar depression: A systematic review and meta-analysis. Asian Journal of Psychiatry, 85, 103618. https://doi.org/10.1016/j.ajp.2023.103618

Chang, J., Chu, Y., Ren, Y., Li, C., Wang, Y., & Chu, X.-P. (2020). Maintenance treatment of transcranial magnetic stimulation (TMS) for treatment-resistant depression patients responding to acute TMS treatment. International Journal of Physiology, Pathophysiology and Pharmacology, 12(5), 128–133.

Cole, E. J., Phillips, A. L., Bentzley, B. S., Stimpson, K. H., Nejad, R., Barmak, F., Veerapal, C., Khan, N., Cherian, K., Felber, E., Brown, R., Choi, E., King, S., Pankow, H., Bishop, J. H., Azeez, A., Coetzee, J., Rapier, R., Odenwald, N., . . . Williams, N. R. (2022). Stanford Neuromodulation Therapy (SNT): A Double-Blind Randomized Controlled Trial. American Journal of Psychiatry, 179(2), 132–141. https://doi.org/10.1176/appi.ajp.2021.20101429

Cole, E. J., Stimpson, K. H., Bentzley, B. S., Gulser, M., Cherian, K., Tischler, C., Nejad, R., Pankow, H., Choi, E., Aaron, H., Espil, F. M., Pannu, J., Xiao, X., Duvio, D., Solvason, H. B., Hawkins, J., Guerra, A., Jo, B., Raj, K. S., . . . Williams, N. R. (2020). Stanford Accelerated Intelligent Neuromodulation Therapy for Treatment-Resistant Depression. American Journal of Psychiatry, 177(8), 716–726. https://doi.org/10.1176/appi.ajp.2019.19070720

d'Andrea, G., Mancusi, G., Santovito, M. C., Marrangone, C., Martino, F., Santorelli, M., Miuli, A., Di Carlo, F., Signorelli, M. S., Clerici, M., Pettorruso, M., & Martinotti, G. (2023). Investigating the Role of Maintenance TMS Protocols for Major Depression: Systematic Review and Future Perspectives for Personalized Interventions. Journal of Personalized Medicine, 13(4), 697. https://doi.org/10.3390/jpm13040697

Deppe, M., Abdelnaim, M., Hebel, T., Kreuzer, P. M., Poeppl, T. B., Langguth, B., & Schecklmann, M. (2021). Concomitant lorazepam use and antidepressive efficacy of repetitive transcranial magnetic stimulation in a naturalistic setting. European Archives of Psychiatry and Clinical Neuroscience, 271(1), 61–67. https://doi.org/10.1007/s00406-020-01160-9

Dougall, N., Maayan, N., Soares-Weiser, K., McDermott, L. M., & McIntosh, A. (2015). Transcranial magnetic stimulation (TMS) for schizophrenia. Cochrane Database of Systematic Reviews, 2015(8). https://doi.org/10.1002/14651858.CD006081.pub2

Duprat, R., Desmyter, S., Rudi, D. R., van Heeringen, K., van den Abbeele, D., Tandt, H., Bakic, J., Pourtois, G., Dedoncker, J., Vervaet, M., van Autreve, S., Lemmens, G. M. D., & Baeken, C. (2016). Accelerated intermittent theta burst stimulation treatment in medication-resistant major depression: A fast road to remission? Journal of Affective Disorders, 200, 6–14. https://doi.org/10.1016/j.jad.2016.04.015

Fitzgerald, P. B., Hoy, K., McQueen, S., Maller, J. J., Herring, S., Segrave, R., Bailey, M., Been, G., Kulkarni, J., & Daskalakis, Z. J. (2009). A Randomized Trial of rTMS Targeted with MRI Based Neuro-Navigation in Treatment-Resistant Depression. Neuropsychopharmacology, 34(5), 1255–1262. https://doi.org/10.1038/npp.2008.233

Fitzsimmons, S. M., van der Werf, Y. D., van Campen, A. D., Arns, M., Sack, A. T., Hoogendoorn, A. W., van den Heuvel, O. A., van Balkom, A. J., Batelaan, N. M., van Eijndhoven, P., Hendriks, G.-J., van Oostrom, I., van Oppena, P., Schruers, K. R., Tendolkar, I., & Vriend, C. (2022). Repetitive transcranial magnetic stimulation for obsessive-compulsive disorder: A systematic review and pairwise/network meta-analysis. Journal of Affective Disorders, 302, 302–312. https://doi.org/10.1016/j.jad.2022.01.048

Folmer, R. L., Theodoroff, S. M., Casiana, L., Shi, Y., Griest, S., & Vachhani, J. (2015). Repetitive Transcranial Magnetic Stimulation Treatment for Chronic Tinnitus. JAMA Otolaryngology–Head & Neck Surgery, 141(8), 716. https://doi.org/10.1001/jamaoto.2015.1219

Gay, A., Cabe, J., Chazeron, I. de, Lambert, C., Defour, M., Bhoowabul, V., Charpeaud, T., Tremey, A., Llorca, P.-M., Pereira, B., & Brousse, G. (2022). Repetitive Transcranial Magnetic Stimulation (rTMS) as a Promising Treatment for Craving in Stimulant Drugs and Behavioral Addiction: A Meta-Analysis. Journal of Clinical Medicine, 11(3). https://doi.org/10.3390/jcm11030624

Giustiniani, A., Vallesi, A., Oliveri, M., Tarantino, V., Ambrosini, E., Bortoletto, M., Masina, F., Busan, P., Siebner, H. R., Fadiga, L., Koch, G., Leocani, L., Lefaucheur, J. P., Rotenberg, A., Zangen, A., Violante, I. R., Moliadze, V., Gamboa, O. L., Ugawa, Y., . . . Burgio, F. (2022). A questionnaire to collect unintended effects of transcranial magnetic stimulation: A consensus based approach. Clinical Neurophysiology : Official Journal of the International Federation of Clinical Neurophysiology, 141, 101–108. https://doi.org/10.1016/j.clinph.2022.06.008

Haesebaert, F., Moirand, R., Schott-Pethelaz, A.-M., Brunelin, J., & Poulet, E. (2018). Usefulness of repetitive transcranial magnetic stimulation as a maintenance treatment in patients with major depression. The World Journal of Biological Psychiatry, 19(1), 74–78. https://doi.org/10.1080/15622975.2016.1255353

He, H., Lu, J., Yang, L., Zheng, J., Gao, F., Zhai, Y., Feng, J., Fan, Y., & Ma, X. (2017). Repetitive transcranial magnetic stimulation for treating the symptoms of schizophrenia: A PRISMA compliant meta-analysis. Clinical Neurophysiology, 128(5), 716–724. https://doi.org/10.1016/j.clinph.2017.02.007

Hebel, T., Abdelnaim, M., Deppe, M., Langguth, B., & Schecklmann, M. (2020). Attenuation of antidepressive effects of transcranial magnetic stimulation in patients whose medication includes drugs for psychosis. Journal of Psychopharmacology, 34(10), 1119–1124. https://doi.org/10.1177/0269881120922965

Hebel, T., Grözinger, M., Landgrebe, M., Padberg, F., Schecklmann, M., Schlaepfer, T., Schönfeldt-Lecuona, C., Ullrich, H., Zwanzger, P., Langguth, B., Bajbouj, M., Bewernick, B., Brinkmann, K., Cordes, J., Di Pauli, J., Eichhammer, P., Freundlieb, N., Hajak, G., Höppner-Buchmann, J., . . . Zilles-Wegner, D. (2022). Evidence and expert consensus based German guidelines for the use of repetitive transcranial magnetic stimulation in depression. The World Journal of Biological Psychiatry, 23(5), 327–348. https://doi.org/10.1080/15622975.2021.1995810

Hebel, T., Schecklmann, M., & Langguth, B. (2020). Transcranial magnetic stimulation in the treatment of depression during pregnancy: a review. Archives of Women‘s Mental Health, 23(4), 469–478. https://doi.org/10.1007/s00737-019-01004-z

Herwig, U., Padberg, F., Unger, J., Spitzer, M., & Schönfeldt-Lecuona, C. (2001). Transcranial magnetic stimulation in therapy studies: examination of the reliability of “standard” coil positioning by neuronavigation. Biological Psychiatry, 50(1), 58–61. https://doi.org/10.1016/S0006-3223(01)01153-2

Herwig, U., Satrapi, P., & Schönfeldt-Lecuona, C. (2003). Using the International 10-20 EEG System for Positioning of Transcranial Magnetic Stimulation. Brain Topography, 16(2), 95–99. https://doi.org/10.1023/B:BRAT.0000006333.93597.9d

Herwig, U., Schönfeldt-Lecuona, C., Wunderlich, A. P., Tiesenhausen, C. von, Thielscher, A., Walter, H., & Spitzer, M. (2001). The navigation of transcranial magnetic stimulation. Psychiatry Research: Neuroimaging, 108(2), 123–131. https://doi.org/10.1016/S0925-4927(01)00121-4

Hızlı Sayar, G., Ozten, E., Tufan, E., Cerit, C., Kağan, G., Dilbaz, N., & Tarhan, N. (2014). Transcranial magnetic stimulation during pregnancy. Archives of Women‘s Mental Health, 17(4), 311–315. https://doi.org/10.1007/s00737-013-0397-0

Hoffman, R. E., Hawkins, K. A., Gueorguieva, R., Boutros, N. N., Rachid, F., Carroll, K., & Krystal, J. H. (2003). Transcranial magnetic stimulation of left temporoparietal cortex and medication-resistant auditory hallucinations. Archives of General Psychiatry, 60(1), 49–56. https://doi.org/10.1001/archpsyc.60.1.49

Huang, Y.-Z.; Edwards, M. J.; Rounis, E.; Bhatia, K. P.; Rothwell, J. C. (2005). Theta burst stimulation of the human motor cortex. Neuron, 45 (2), 201–206. https://doi.org/10.1016/j.neuron.2004.12.033

Hunter, A. M., Minzenberg, M. J., Cook, I. A., Krantz, D. E., Levitt, J. G., Rotstein, N. M., Chawla, S. A., & Leuchter, A. F. (2019). Concomitant medication use and clinical outcome of repetitive Transcranial Magnetic Stimulation (rTMS) treatment of Major Depressive Disorder. Brain and Behavior, 9(5), e01275. https://doi.org/10.1002/brb3.1275

Hyde, J., Carr, H., Kelley, N., Seneviratne, R., Reed, C., Parlatini, V., Garner, M., Solmi, M., Rosson, S., Cortese, S., & Brandt, V. (2022). Efficacy of neurostimulation across mental disorders: systematic review and meta-analysis of 208 randomized controlled trials. Molecular Psychiatry, 27(6), 2709–2719. https://doi.org/10.1038/s41380-022-01524-8

Kim, D. R., Wang, E., McGeehan, B., Snell, J., Ewing, G., Iannelli, C., O'Reardon, J. P., Sammel, M. D., & Epperson, C. N. (2019). Randomized controlled trial of transcranial magnetic stimulation in pregnant women with major depressive disorder. Brain Stimulation, 12(1), 96–102. https://doi.org/10.1016/j.brs.2018.09.005

Kim, H., Wright, D. L., Rhee, J., & Kim, T. (2023). C3 in the 10-20 system may not be the best target for the motor hand area. Brain Research, 1807, 148311. https://doi.org/10.1016/j.brainres.2023.148311

Kishi, T., Ikuta, T., Sakuma, K., Hatano, M., Matsuda, Y., Wilkening, J., Goya-Maldonado, R., Tik, M., Williams, N. R., Kito, S., & Iwata, N. (2024). Theta burst stimulation for depression: A systematic review and network and pairwise meta-analysis. Molecular Psychiatry, 29(12), 3893–3899. https://doi.org/10.1038/s41380-024-02630-5

Koessler, L., Maillard, L., Benhadid, A., Vignal, J. P., Felblinger, J., Vespignani, H., & Braun, M. (2009). Automated cortical projection of EEG sensors: Anatomical correlation via the international 10–10 system. NeuroImage, 46(1), 64–72. https://doi.org/10.1016/j.neuroimage.2009.02.006

Langguth, B., Zowe, M., Landgrebe, M., Sand, P., Kleinjung, T., Binder, H., Hajak, G., & Eichhammer, P. (2006). Transcranial magnetic stimulation for the treatment of tinnitus: A new coil positioning method and first results. Brain Topography, 18(4), 241–247. https://doi.org/10.1007/s10548-006-0002-1

Lefaucheur, J.-P., Aleman, A., Baeken, C., Benninger, D. H., Brunelin, J., Di Lazzaro, V., Filipović, S. R., Grefkes, C., Hasan, A., Hummel, F. C., Jääskeläinen, S. K., Langguth, B., Leocani, L., Londero, A., Nardone, R., Nguyen, J.-P., Nyffeler, T., Oliveira-Maia, A. J., Oliviero, A., . . . Ziemann, U. (2020). Evidence-based guidelines on the therapeutic use of repetitive transcranial magnetic stimulation (rTMS): An update (2014–2018). Clinical Neurophysiology, 131(2), 474–528. https://doi.org/10.1016/j.clinph.2019.11.002

Lefaucheur, J.-P., André-Obadia, N., Antal, A., Ayache, S. S., Baeken, C., Benninger, D. H., Cantello, R. M., Cincotta, M., Carvalho, M. de, Ridder, D. de, Devanne, H., Di Lazzaro, V., Filipović, S. R., Hummel, F. C., Jääskeläinen, S. K., Kimiskidis, V. K., Koch, G., Langguth, B., Nyffeler, T., . . . Garcia-Larrea, L. (2014). Evidence-based guidelines on the therapeutic use of repetitive transcranial magnetic stimulation (rTMS). Clinical Neurophysiology, 125(11), 2150–2206. https://doi.org/10.1016/j.clinph.2014.05.021

Liu, B., Zhang, Y., Zhang, L., & Li, L. (2014). Repetitive transcranial magnetic stimulation as an augmentative strategy for treatment-resistant depression, a meta-analysis of randomized, double-blind and sham-controlled study. BMC Psychiatry, 14(1), 342. https://doi.org/10.1186/s12888-014-0342-4

Liu, H., Wang, X., Gong, T., Xu, S., Zhang, J., Yan, L., Zeng, Y., Yi, M., & Qian, Y. (2024). Neuromodulation treatments for post-traumatic stress disorder: A systematic review and network meta-analysis covering efficacy, acceptability, and follow-up effects. Journal of Anxiety Disorders, 106, 102912. https://doi.org/10.1016/j.janxdis.2024.102912

Mantovani, A., Lisanby, S. H., Pieraccini, F., Ulivelli, M., Castrogiovanni, P., & Rossi, S. (2006). Repetitive transcranial magnetic stimulation (rTMS) in the treatment of obsessive–compulsive disorder (OCD) and Tourette's syndrome (TS). The International Journal of Neuropsychopharmacology, 9(01), 95. https://doi.org/10.1017/S1461145705005729

McGirr, A., Devoe, D. J., Raedler, A., Debert, C. T., Ismail, Z., & Berlim, M. T. (2021). Repetitive Transcranial Magnetic Stimulation for the Treatment of Post-traumatic Stress Disorder: A Systematic Review and Network Meta-analysis: La Stimulation Magnétique Transcrânienne Répétitive Pour le Traitement du Trouble de Stress Post-Traumatique : Une Revue Systématique et une Méta-Analyse en Réseau. Canadian Journal of Psychiatry. Revue Canadienne De Psychiatrie, 66(9), 763–773. https://doi.org/10.1177/0706743720982432

Mylius, V., Ayache, S. S., Ahdab, R., Farhat, W. H., Zouari, H. G., Belke, M., Brugières, P., Wehrmann, E., Krakow, K., Timmesfeld, N., Schmidt, S., Oertel, W. H., Knake, S., & Lefaucheur, J. P. (2013). Definition of DLPFC and M1 according to anatomical landmarks for navigated brain stimulation: Inter-rater reliability, accuracy, and influence of gender and age. NeuroImage, 78, 224–232. https://doi.org/10.1016/j.neuroimage.2013.03.061

Osoegawa, C., Gomes, J. S., Grigolon, R. B., Brietzke, E., Gadelha, A., Lacerda, A. L., Dias, Á. M., Cordeiro, Q., Laranjeira, R., Jesus, D. de, Daskalakis, Z. J., Brunelin, Jz., Cordes, J., & Trevizol, A. P. (2018). Non-invasive brain stimulation for negative symptoms in schizophrenia: An updated systematic review and meta-analysis. Schizophrenia Research, 197, 34–44. https://doi.org/10.1016/j.schres.2018.01.010

Patel, R., Silla, F., Pierce, S., Theule, J., & Girard, T. A. (2020). Cognitive functioning before and after repetitive transcranial magnetic stimulation (rTMS): A quantitative meta-analysis in healthy adults. Neuropsychologia, 141, 107395. https://doi.org/10.1016/j.neuropsychologia.2020.107395

Plewnia, C., Brendel, B., Schwippel, T., Martus, P., Cordes, J., Hasan, A., & Fallgatter, A. J. (2018). Treatment of auditory hallucinations with bilateral theta burst stimulation (cTBS): Protocol of a randomized, double-blind, placebo-controlled, multicenter trial. European Archives of Psychiatry and Clinical Neuroscience, 268(7), 663–673. https://doi.org/10.1007/s00406-017-0861-3

Rossi, S., Antal, A., Bestmann, S., Bikson, M., Brewer, C., Brockmöller, J., Carpenter, L. L., Cincotta, M., Chen, R., Daskalakis, J. D., Di Lazzaro, V., Fox, M. D., George, M. S., Gilbert, D., Kimiskidis, V. K., Koch, G., Ilmoniemi, R. J., Lefaucheur, J. P., Leocani, L., . . . Hallett, M. (2021). Safety and recommendations for TMS use in healthy subjects and patient populations, with updates on training, ethical and regulatory issues: Expert Guidelines. Clinical Neurophysiology, 132(1), 269–306. https://doi.org/10.1016/j.clinph.2020.10.003

Rossi, S., Hallett, M., Rossini, P. M., & Pascual-Leone, A. (2009). Safety, ethical considerations, and application guidelines for the use of transcranial magnetic stimulation in clinical practice and research. Clinical Neurophysiology, 120(12), 2008–2039. https://doi.org/10.1016/j.clinph.2009.08.016

Rutherford, G., Lithgow, B., & Moussavi, Z. (2020). Transcranial magnetic stimulation safety from operator exposure perspective. Medical & Biological Engineering & Computing, 58(2), 249–256. https://doi.org/10.1007/s11517-019-02084-w

Schönfeldt-Lecuona, C., Cárdenas-Morales, L., Moreno-Aguirre, A., Dorn, K., Langguth, B., Brühl, A. B., Kammer, T., & Herwig, U. (2012). Effect of 1 Hz Repetitive Transcranial Magnetic Stimulation Over the Auditory Cortex on Audiometry and Otoacustic Emissions. Brain Topography, 25(3), 241–247. https://doi.org/10.1007/s10548-012-0218-1

Schönfeldt-Lecuona, C., Lefaucheur, J.-P., Cardenas-Morales, L., Wolf, R. C., Kammer, T., & Herwig, U. (2010). The value of neuronavigated rTMS for the treatment of depression. Neurophysiologie Clinique/Clinical Neurophysiology, 40(1), 37–43. https://doi.org/10.1016/j.neucli.2009.06.004

Schönfeldt-Lecuona, C., Thielscher, A., Freudenmann, R. W., Kron, M., Spitzer, M., & Herwig, U. (2005). Accuracy of Stereotaxic Positioning of Transcranial Magnetic Stimulation. Brain Topography, 17(4), 253–259. https://doi.org/10.1007/s10548-005-6033-1

Shafi, M. M. (2019). Seizures with TMS: Much ado about (almost) nothing? Clinical Neurophysiology, 130(8), 1397–1398. https://doi.org/10.1016/j.clinph.2019.04.315

Tseng, P.-T., Zeng, B.-S., Hung, C.-M., Liang, C.-S., Stubbs, B., Carvalho, A. F., Brunoni, A. R., Su, K.-P., Tu, Y.-K., Wu, Y.-C., Chen, T.-Y., Li, D.-J., Lin, P.-Y., Hsu, C.-W., Chen, Y.-W., Suen, M.-W., Satogami, K., Takahashi, S., Wu, C.-K., . . . Li, C.-T. (2022). Assessment of Noninvasive Brain Stimulation Interventions for Negative Symptoms of Schizophrenia. JAMA Psychiatry, 79(8), 770. https://doi.org/10.1001/jamapsychiatry.2022.1513

Wang, B., Peterchev, A. V., & Goetz, S. M. (2023). Three novel methods for determining motor threshold with transcranial magnetic stimulation outperform conventional procedures. Journal of Neural Engineering, 20(5). https://doi.org/10.1088/1741-2552/acf1cc

Westin, G. G., Bassi, B. D., Lisanby, S. H., & Luber, B. (2014). Determination of motor threshold using visual observation overestimates transcranial magnetic stimulation dosage: Safety implications. Clinical Neurophysiology : Official Journal of the International Federation of Clinical Neurophysiology, 125(1), 142–147. https://doi.org/10.1016/j.clinph.2013.06.187

Wilson, S., Croarkin, P. E., Aaronson, S. T., Carpenter, L. L., Cochran, M., Stultz, D. J., & Kozel, F. A. (2022). Systematic review of preservation TMS that includes continuation, maintenance, relapse-prevention, and rescue TMS. Journal of Affective Disorders, 296, 79–88. https://doi.org/10.1016/j.jad.2021.09.040

Ye, S.-Y., Chen, C.-N., Wei, B., Zhan, J.-Q., Li, Y.-H., Zhang, C., Huang, J.-J., & Yang, Y.-J. (2024). The efficacy and safety of continuous theta burst stimulation for auditory hallucinations: A systematic review and meta-analysis of randomized controlled trials. Frontiers in Psychiatry, 15, 1446849. https://doi.org/10.3389/fpsyt.2024.1446849

Zaidi, A., Shami, R., Sewell, I. J., Cao, X., Giacobbe, P., Rabin, J. S., Goubran, M., Hamani, C., Swardfager, W., Davidson, B., Lipsman, N., & Nestor, S. M. (2024). Antidepressant class and concurrent rTMS outcomes in major depressive disorder: A systematic review and meta-analysis. EClinicalMedicine, 75, 102760. https://doi.org/10.1016/j.eclinm.2024.102760

Zhang, J. J. Q., Fong, K. N. K., Ouyang, R.-G., Siu, A. M. H., & Kranz, G. S. (2019). Effects of repetitive transcranial magnetic stimulation (rTMS) on craving and substance consumption in patients with substance

dependence: A systematic review and meta-analysis. Addiction (Abingdon, England), 114(12), 2137–2149. https://doi.org/10.1111/add.14753

Zhang, W., Deng, B., Xie, F., Zhou, H., Guo, J.-F., Jiang, H., Sim, A., Tang, B., & Wang, Q. (2022). Efficacy of repetitive transcranial magnetic stimulation in Parkinson's disease: A systematic review and meta-analysis of randomised controlled trials. EClinicalMedicine, 52, 101589. https://doi.org/10.1016/j.eclinm.2022.101589

Ziemann, U., Reis, J., Schwenkreis, P., Rosanova, M., Strafella, A., Badawy, R., & Müller-Dahlhaus, F. (2015). TMS and drugs revisited 2014. Clinical Neurophysiology, 126(10), 1847–1868. https://doi.org/10.1016/j.clinph.2014.08.028

Appendix A: Suggested pre-treatment informed consent sheet

Informed consent: treatment with transcranial magnetic stimulation

Dear patient,

for the treatment of your symptoms, we are planning to utilize a magnetic brain stimulation technique known as repetitive Transcranial Magnetic Stimulation (rTMS). This procedure is scientifically recognized, with known effectiveness in certain neurological and psychiatric medical conditions.

What is transcranial magnetic stimulation?

Transcranial magnetic stimulation (TMS) involves stimulating specific areas of the brain (transcranial = through the skull). A figure-8-shaped-coil is placed on the outside of the head. Very short magnetic pulses are generated via this coil that enable targeted influencing of brain activity. An example of its application in routine neurological diagnostics involves inducing a muscle twitch in the limbs through a single magnetic pulse. This targets the area of the brain responsible for movement, the motor cortex. For treatment with TMS, other brain areas, such as the frontal cortex, are targeted for therapy and many such magnetic pulses are given in repeated succession. This form of TMS works as a treatment and is called repetitive TMS (rTMS; repetitive = repeated pulses), which causes longer-lasting changes in brain activity that can lead to symptom relief.

How does treatment with magnetic stimulation work?

The stimulation intensity is determined before or on the first day of treatment. For this purpose, the motor cortex of the brain is stimulated with single pulses. The resulting muscle twitches are recorded by electrodes attached to the corresponding limb (e.g. the little finger). The actual treatment takes place every weekday for a total of 5 times a week. It is carried out over a period of one or more weeks. Each treatment session lasts a few minutes. It is to be expected that the therapeutic effect will accumulate progressively over the course of treatments.

What side effects are to be expected?

1. Electrodes are attached to the limbs to measure stimulation intensity. Skin disinfectant (usually alcohol-based) and electrode gel or special adhesive electrodes are used. This can cause slight temporary skin irritation.
2. The single stimulation pulses can be accompanied by tingling on the scalp or muscle twitching (e.g. jaw muscle), which can be slightly painful.
3. The single magnetic pulses are accompanied by a relatively loud noise. Hence, it is advisable to wear the provided hearing protection during the treatment.
4. Headaches can also occur during and after stimulation. These are usually temporary and can be easily treated with headache medication.

5. In individual cases, the stimulation can be irritating. This can lead to dizziness, circulatory problems or in rare cases to a brief fainting spell.
6. Stimulation with rTMS may trigger an epileptic seizure. However, this risk is considered to be very low and is only increased in people with pre-existing neurological conditions such as epilepsy.
7. Just as we cannot promise any improvement, we also cannot rule out a worsening of symptoms as a result of the treatment.
8. Implanted devices, such as a pacemaker, have the potential to be damaged or cause harm to the body through interactions with magnetic stimulation.

What health aspects need to be considered?

Treatment should be avoided in certain groups of people, or at the very least, special care should be taken, as these individuals may face an elevated risk of experiencing side effects. The following contraindications are relevant:

	Yes or No		If yes,<br>details and evaluation:
electrical or metallic objects in/on the head or body, in particular:<br>• implanted devices (pacemakers, cochlear implants, etc.)<br>• metal splinters<br>• vascular clips<br>• implants in general	O No	O Yes	Earrings, piercings, dental fillings, crowns and implants are no problem.
neurological conditions, in particular:<br>• epilepsy<br>• single epileptic seizure<br>• craniocerebral trauma<br>• stroke<br>• brain damage	O No	O Yes	
severe, non-stable medical condition	O No	O Yes	
tendency to faint	O No	O Yes	
pregnancy	O No	O Yes	
medication, especially seizure threshold relevant.	O No	O Yes	
prior rTMS treatment	O No	O Yes	If so, how was it tolerated?

history of mania	O No	O Yes	

What are the chances of success?

rTMS is a relatively novel treatment method that is actively being explored for its effectiveness in addressing various medical conditions. However, additional studies are still needed to establish reliable conclusions regarding its effectiveness. The current state of research, based on evidence-based guidelines from international experts, is as follows:

**medical condition**	**effectiveness of rTMS**
depression	proven effective
post-traumatic stress disorder	probably effective
schizophrenia	effective for negative symptoms in some cases<br>possibly effective for acoustic hallucinations
tinnitus, compulsive behavior	possibly effective
substance abuse and addiction	possibly effective with nicotine
anxiety disorders	currently no recommendation possible

If you have any questions about the treatment, please contact the treatment team freely. If you experience side effects of any kind, please let us know immediately. Of course, you may discontinue treatment at any time. You are aware that there are other treatment options for depression.

The planned treatment schedule involves (number of sessions in which period of time):

____

Each treatment session lasts approximately ____ minutes. In addition to the treatments, findings are collected by the practitioners during interviews and in the form of questionnaires.

Description of the treatment: ______________________ Indication: ____________________________

Time given to consider: ________________________ O abstained from time to consider

Copy of the information sheet and written consent:

O Given to the patient O Patient waives copy

Remarks: ____

Location: ________________________ Date: ___.___.______ Time: ___.___ AM/PM

________________________	________________________	________________________
Name of consultation partner	Patient‘s name	Name of specialised physician
________________________	________________________	________________________
Signature of consultation partner	Patient's signature	Signature of specialised physician

Appendix B: Suggested questionnaire for TMS side effect assessment

Questionnaire on Side Effects of rTMS Treatment

Have you experienced any of the following side effects during or after rTMS sessions throughout the treatment period?
For each sensation, first indicate whether you experienced it at all during the treatment period (check the box in the first column).
If the sensation was present, please assess the following aspects:

1. Please rate the **intensity** of the sensation on a scale from 1 to 4:
   • 1 = Mild
   • 2 = Moderate
   • 3 = Strong
   • 4 = Very strong
2. Please also indicate the **duration** of the sensation over the entire treatment period:
   • 1 = Rare (once or twice)
   • 2 = Occasional (on several days)
   • 3 = Frequent (on more than half of the days)
   • 4 = Throughout the entire treatment period

<u>Please indicate the sensations as follows:</u>

Sensation	Present?		Intensity (1-4)<br>Only if present				Duration (1-4)<br>Only if present			
Scalp pain	Yes	No	1	2	3	4	1	2	3	4
Toothache	Yes	No	1	2	3	4	1	2	3	4
Tingling sensation on the scalp	Yes	No	1	2	3	4	1	2	3	4
Itching	Yes	No	1	2	3	4	1	2	3	4
Burning or heat sensation	Yes	No	1	2	3	4	1	2	3	4
Headache	Yes	No	1	2	3	4	1	2	3	4
Noises (e.g., tinnitus)	Yes	No	1	2	3	4	1	2	3	4
Muscle contractions	Yes	No	1	2	3	4	1	2	3	4
Fatigue/drowsiness	Yes	No	1	2	3	4	1	2	3	4
Changes in hearing	Yes	No	1	2	3	4	1	2	3	4
Mood changes (depression)	Yes	No	1	2	3	4	1	2	3	4
Mood changes (euphoria)	Yes	No	1	2	3	4	1	2	3	4
Nausea	Yes	No	1	2	3	4	1	2	3	4
Dizziness	Yes	No	1	2	3	4	1	2	3	4
Neck stiffness/pain	Yes	No	1	2	3	4	1	2	3	4
Pressure sensation from the coil	Yes	No	1	2	3	4	1	2	3	4
Anxiety/nervousness	Yes	No	1	2	3	4	1	2	3	4
Difficulty concentrating	Yes	No	1	2	3	4	1	2	3	4
Memory problems	Yes	No	1	2	3	4	1	2	3	4
Other (please specify):	Yes	No	1	2	3	4	1	2	3	4

(adaptiert vom TMSens_Q – Fragebogen; siehe Giustiniani et al., 2022)
