# Supplementary material for: rTMS for the treatment of psychiatric disorders: a review about training courses and materials and the presentation of the training materials of the German Society for Brain Stimulation in Psychiatry
Source: Front Psychiatry. 2025 Aug 8;16:1490039. doi: 10.3389/fpsyt.2025.1490039 (PMC12371536; doi:10.3389/fpsyt.2025.1490039)
Supplement: Supplementary file 1 [file SupplementaryFile1.zip › Exam Questions (German).PDF]

Transkranielle Magnetstimulation

Deutsche Gesellschaft für Hirnstimulation in der Psychiatrie e.V. (DGHP)

Hands-On-Workshop Beginner Juni 2024

Deutsche Gesellschaft für
Hirnstimulation
in der Psychiatrie e. V.

Prüfungsfragen (richtige Antwort = fett)

1. Wie wirkt rTMS technisch-physiologisch?

A) Es werden kortikale Neurone durch die positive Wirkung magnetischer Stoßwellen beeinflusst.

B) Die Vibration der magnetischen Impulse führt zu einer vermehrten Ausschüttung neurotropher Wachstumsfaktoren.

C) Durch das physikalische Prinzip der elektromagnetischen Induktion werden durch einen Stromfluss im leitfähigen Hirngewebe unter der Magnetspule kortikale Neurone depolarisiert.

D) Während der Stimulation wird die linke Hemisphäre kurzzeitig magnetisiert. Freie Radikale werden dadurch magnetisch angezogen und anschließend besser verstoffwechselt.

2. Wie wirkt die rTMS therapeutisch?

A) Sie kann nur kurzzeitige Effekte induzieren. Diese sind auf die Dauer der Therapiesitzungen begrenzt.

B) Sie wirkt neuroplastisch und kann dabei nur aktivierend auf neuronale Netzwerke wirken.

C) Sie kann die Hirnfunktion über Wochen und Monate hinweg verändern.

D) Sie wirkt mit einem 1Hz-Protokoll ausschließlich hemmend und mit einem 10Hz-Protokoll streng aktivierend.

3. Welche Aussage bezüglich der Eindringtiefe der rTMS-Pulse stimmt?

A) Da rTMS Magnetfelder nicht durch Knochen oder andere Strukturen “aufgehalten” werden, können sie ungehindert bis in den Hirnstamm eindringen.

B) Das Magnetfeld hat eine direkte Stimulationswirkung im Bereich der Hirnrinde.

C) Bestimmte Spulen erlauben eine größere Eindringtiefe. Bei diesen speziellen Spulen spielen Effekte, die in Hirnarealen zwischen diesen tieferen Zielregionen und der Spule sattfinden, keine Rolle.

D) Die Motorschwelle ist ein Maß für die Erregbarkeit des Motorkortex und auch anderer kortikaler Hirnareale.

4. Wieso wird zur korrekten Bestimmung der RMT (Ruhe-Motorschwelle) eine Elektromyographie (EMG) empfohlen?

A) Die Patienten fühlen sich durch die technische Versorgung besser.

B) Mit Hilfe des EMG kann das individuelle Risiko für einen induzierten Anfall genauer bestimmt und damit vermieden werden.

C) Eine Bestimmung der Motorschwelle ist mit EMG unter Umständen genauer, weil auch nicht sichtbare Muskelaktivität gemessen werden kann.

D) Die EMG sorgt dafür, dass die TMS-Pulse bis in die kleinen Handmuskeln fortgeleitet werden.

5. Welche Aussage zum Risikomanagement ist richtig?

A) Zum besseren Risikoscreening sollte ein EEG vor dem Behandlungsstart durchgeführt werden, da induzierte Anfälle häufig unter rTMS zu beobachten sind.

B) Synkopen als Nebenwirkung sind völlig unproblematisch, weil sie nur unter hoher Anspannung auftreten und durch Beruhigungsstrategien immer leicht zu vermeiden sind.

C) Eine Schwangerschaft ist eine absolute Kontraindikation für die rTMS.

D) Ein Hörschutz während der Behandlung sollte empfohlen werden.

6. Welche Aussage bezüglich der Aufklärung ist richtig.

A) Ein unterschriebenes schriftliches Aufklärungsdokument reicht.

B) Die Aufklärung muss mündlich erfolgen und muss dokumentiert werden.

C) Behandlungsalternativen müssen nicht genannt werden.

D) Da rTMS wenig Nebenwirkungen hat, ist eine Bedenkzeit nicht notwendig.

7. Welches der folgenden Behandlungsprotokolle ist nach vorliegender Evidenz bei unipolarer depressiver Störung das Effektivste?

A) Stimulation über dem linken DLPFC und einem hochfrequenten Protokoll

B) Stimulation über dem rechten DLPFC und einem hochfrequenten Protokoll

C) Stimulation über dem linken DLPFC und einem niedrigfrequenten Protokoll

D) Stimulation über dem rechten DLPFC und einem niedrigfrequenten Protokoll

8. Welche Aussage zur Depressionsbehandlung mit rTMS ist richtig?

A) Menschen mit bipolaren Depressionen profitieren nach neuen Studienerkenntnissen nicht von einer Behandlung mit rTMS.

B) Aufgrund zu hoher Risiken darf die rTMS bei peripartalen Depressionen nicht als individueller Heilversuch angeboten werden.

C) Bei Menschen mit einer Depression über dem 65. Lebensjahr besteht keine Evidenz für eine Wirksamkeit neuroplastischer Therapien.

D) Die rTMS kann zusätzlich zu den meisten Medikamenten verwendet werden.

9. Wie hoch ist die durchschnittliche Ansprechrate bei Menschen mit Depressionen auf eine reguläre Behandlung mit rTMS?

A) Nach vier Wochen sind ungefähr 90% der Patienten in Remission.

B) Ungefähr 10-50% der Patientinnen und Patienten sprechen auf die Behandlung an.

C) In Kombination mit Moclobemid kann die Remissionsrate auf bis zu 70 % gesteigert werden.

D) Die Ansprechrate nach vier Wochen liegt bei 5%.

10. Sie haben aktuell nur einen Behandlungsplatz für die rTMS zur Verfügung. Welchen der genannten Patienten behandeln sie am ehesten?

A) 27-jährige schwangere Patientin mit zweiter depressiver Episode seit 3 Wochen, die keine Medikamente einnehmen will und schon einmal von rTMS profitiert hat.

B) 68-jähriger Patient mit therapieresistenter Depression und somatischen Komorbiditäten inkl. zahlreicher somatischer und psychiatrischer Medikamente.

C) 41-jähriger Patient mit depressivem Stupor.

D) 25-jährige Patientin mit Borderline-Persönlichkeitsstörung, ADHS und PTBS, die hohen Leidensdruck hat, weil viele Medikamente in der Vergangenheit nicht geholfen haben.

11. Wie kann die Stimulationsintensität am effektivsten bestimmt werden?

A) Indem man die Patientinnen und Patienten während der Stimulation nach der Stärke des empfundenen Schmerzgefühls fragt und ggf. die Stimulationsstärke anpasst.

B) Indem man einen Gruppen-Referenzwert aus einer wissenschaftlichen Veröffentlichung verwendet.

C) Indem man die individuelle Motorschwelle als Maß für die kortikale Erregbarkeit bestimmt.

D) Am besten nimmt man 60% des Stimulator-Outputs.

12. Welche Aussage zur Neuronavigation beim Einsatz der rTMS für die Behandlung der Depression am DLPFC (dorso-lateralen Präfrontal-Cortex) trifft zu?

A) Eine Neuronavigation ist allen anderen Methoden immer vorzuziehen, weil sie immer stärkere Effekte bewirkt.

B) Eine Neuronavigation stellt eine Methode dar, um Hirnareale für die Stimulation genau zu definieren. Die Lokalisation mittels EEG-Punkten wie z.B. der Beam-F3-Methode führt allerdings zu einer ähnlichen anatomischen Genauigkeit.

C) Die 5cm-Regel stellt bis heute den Goldstandard der rTMS in der klinischen Versorgung dar.

D) Die Neuronavigation ist v.a. bei Tinnitus anzuwenden, weil hier die Präzision der Spulenlokalisation besonders wichtig ist.
